# Efficient synthesis of chiral 2,3-dihydro-benzo[*b*]thiophene 1,1-dioxides *via* Rh-catalyzed hydrogenation†
†Electronic supplementary information (ESI) available. See DOI: 10.1039/c8sc05397a


**DOI:** 10.1039/c8sc05397a

**Published:** 2019-01-15

**Authors:** Gongyi Liu, Heng Zhang, Yi Huang, Zhengyu Han, Gang Liu, Yuanhua Liu, Xiu-Qin Dong, Xumu Zhang

**Affiliations:** a Key Laboratory of Biomedical Polymers , Engineering Research Center of Organosilicon Compounds & Materials , Ministry of Education , College of Chemistry and Molecular Sciences , Wuhan University , Wuhan , Hubei 430072 , P. R. China . Email: xiuqindong@whu.edu.cn; b Department of Chemistry , Shenzhen Grubbs Institute , Southern University of Science and Technology , Shenzhen , Guangdong 518055 , P. R. China . Email: zhangxm@sustc.edu.cn

## Abstract

Rh-catalyzed asymmetric hydrogenation of prochiral substituted benzo[*b*]thiophene 1,1-dioxides was successfully developed, affording various chiral 2,3-dihydrobenzo[*b*]thiophene 1,1-dioxides with high yields and excellent enantioselectivities (up to 99% yield and >99% ee). In particular, for challenging substrates, such as aryl substituted substrates with sterically hindered groups and alkyl substituted substrates, the reaction proceeded smoothly in our catalytic system with excellent results. The gram-scale asymmetric hydrogenation proceeded well with 99% yield and 99% ee in the presence of 0.02 mol% (S/C = 5000) catalyst loading. The possible hydrogen-bonding interaction between the substrate and the ligand may play an important role in achieving high reactivity and excellent enantioselectivity.

## Abstract 2

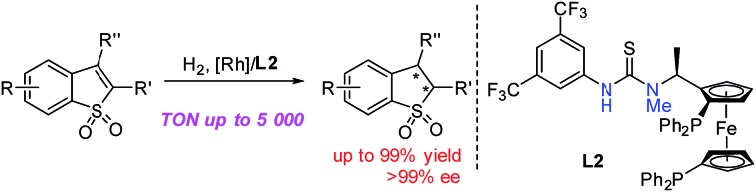
Rh-Catalyzed asymmetric hydrogenation of substituted benzo[*b*]thiophene 1,1-dioxides was successfully developed. Both aromatic and alkyl substituted benzo[*b*]thiophene 1,1-dioxide substrates worked well with high yields and excellent enantioselectivities.

## Introduction

The 2,3-dihydro-benzo[*b*]thiophene 1,1-dioxides and derivative motifs are widely distributed with significant applications in many biologically active compounds,^1^–^6^ such as the inhibitor of tumour necrosis factor-α converting enzyme (TACE),^2^ antidiabetics^3^ and HIF-2a inhibitors.^4^ Other examples include benzothiophene scaffolds, such as 2,3-dihydroraloxifene as raloxifene's analogue with selective estrogen receptor modulator activity^5^ and a potential HIV-1 reverse transcriptase inhibitor (NSC-380292).^6^ In addition, they are important synthetic intermediates in the field of organic synthesis.

**Scheme 1:**
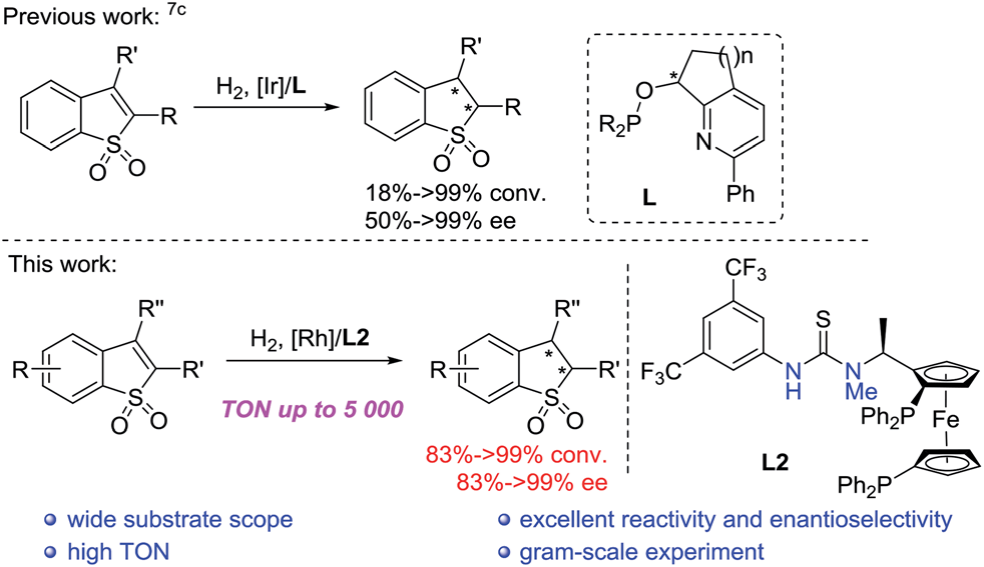
Asymmetric hydrogenation of prochiral benzo[*b*]thiophene 1,1-dioxides.

Although chiral 2,3-dihydro-benzo[*b*]thiophene 1,1-dioxides and their derivatives showed great potential, the development of highly efficient asymmetric synthetic methodologies to construct these compounds still remains very challenging.^7^,^8^ In 2017, Pfaltz and co-workers developed the asymmetric hydrogenation of prochiral benzo[*b*]thiophene 1,1-dioxides by using the Ir/pyridyl phosphinite ligand complex with moderate to excellent enantioselectivities, whereas for some aryl substituted substrates with slightly sterically hindered groups and alkyl substituted substrates it remained difficult to achieve both high reactivity and excellent enantioselectivity (Scheme 1).7*c* Although some progress was achieved, it is extremely necessary to develop highly efficient asymmetric catalytic systems to prepare chiral 2,3-dihydro-benzo[*b*]thiophene 1,1-dioxides and their derivatives. Transition metal-catalyzed asymmetric hydrogenation of prochiral unsaturated heterocyclic compounds is a powerful and important method to synthesize chiral heterocyclic compounds.^9^,^10^ Meanwhile, chiral ferrocenyl phosphine ligands have emerged as a class of important and privileged ligands, which exhibited excellent performance in asymmetric catalytic reactions.^11^ Recently, our group successfully developed a series of bifunctional ferrocenyl bisphosphine-thiourea ligands, which were applied in some Rh-catalyzed asymmetric hydrogenation of unsaturated functionalized substrates.^12^ We envisaged that the asymmetric hydrogenation of prochiral substituted benzo[*b*]thiophene 1,1-dioxides could proceed well with high reactivity and excellent enantioselective control with the aid of the possible hydrogen-bonding interaction between the sulfonyl group of the substrate and the thiourea motif of the ligand. Herein, we realized Rh-catalyzed asymmetric hydrogenation of prochiral benzo[*b*]ene 1,1-dioxides with *N*-methylated bisphosphine-thiourea ZhaoPhos **L2** as the ligand, affording various chiral 2,3-dihydro-benzo[*b*]thiophene 1,1-dioxides with up to >99% conversion, >99% ee and 5000 TON (Scheme 1). Challenging substrates, such as aryl substituted substrates with sterically hindered groups and alkyl substituted substrates, also performed well in our catalytic system with excellent results.

## Results and discussion

The initial investigation of the Rh-catalyzed asymmetric hydrogenation of 2-phenylbenzo[*b*]thiophene 1,1-dioxide (**1a**)^13^ as a model substrate was conducted with different metal sources using ligand ZhaoPhos **L1** (S/C = 100) under 50 atm H_2_ in CH_2_Cl_2_ at 50 °C for 40 h (Table 1, entries 1–4). The Rh(NBD)_2_BF_4_ afforded the best result with high conversion and excellent enantioselectivity (94% conversion, 93% ee, Table 1, entry 1). The conversion can be improved to 99%, when the reaction temperature is increased from 50 °C to 70 °C (Table 1, entry 5). In order to achieve good solubility of the Rh(NBD)_2_BF_4_/ZhaoPhos **L1** catalytic system, the catalyst was generated *in stiu* by mixing Rh(NBD)_2_BF_4_/ZhaoPhos **L1** in CH_2_Cl_2_. The solvent effect of this asymmetric hydrogenation was investigated in various solvents. Excellent enantioselectivities can be obtained in mixed solvents of dichloroethane, CHCl_3_, MeOH, EtOH, ^i^PrOH or tetrahydrofuran in CH_2_Cl_2_ with the volume ratio of 10 : 1, but the reactivities were very poor (15–51% conversion, 90–99% ee, Table 1, entries 6–7, 9–11, and 14). Good to excellent reactivities and enantioselectivities were observed in the mixed solvents of CF_3_CH_2_OH, ethyl acetate or toluene in CH_2_Cl_2_ (87–>99% conversion, 87–>99% ee, Table 1, entries 8 and 12–13). And the mixed solvent CF_3_CH_2_OH/CH_2_Cl_2_ (10 : 1) was chosen as the best reaction solvent with full conversion and >99% ee (Table 1, entry 8).

**Table 1:** Screening metal sources and solvents for asymmetric hydrogenation of 2-phenylbenzo[*b*]thiophene 1,1-dioxide **1a**^*a*^

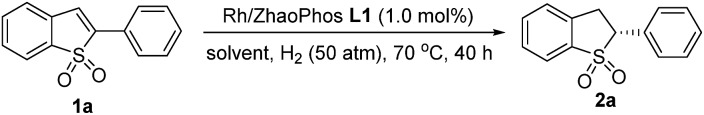				
Entry	Metal source	Solvent	Conv.^*b*^ [%]	ee^*c*^ [%]
1^*d*^	Rh(NBD)_2_BF_4_	CH_2_Cl_2_	94	93
2^*d*^	Rh(COD)_2_BF_4_	CH_2_Cl_2_	92	87
3^*d*^	[Rh(COD)Cl]_2_	CH_2_Cl_2_	44	95
4^*d*^	Rh(COD)_2_CF_3_SO_3_	CH_2_Cl_2_	NR	NA
5	Rh(NBD)_2_BF_4_	CH_2_Cl_2_	99	93
6	Rh(NBD)_2_BF_4_	DCE : CH_2_Cl_2_ = 10 : 1	23	95
7	Rh(NBD)_2_BF_4_	CHCl_3_ : CH_2_Cl_2_ = 10 : 1	15	95
8	Rh(NBD)_2_BF_4_	TFE : CH_2_Cl_2_ = 10 : 1	>99	>99
9	Rh(NBD)_2_BF_4_	MeOH : CH_2_Cl_2_ = 10 : 1	34	95
10	Rh(NBD)_2_BF_4_	EtOH : CH_2_Cl_2_ = 10 : 1	44	90
11	Rh(NBD)_2_BF_4_	^i^PrOH : CH_2_Cl_2_ = 10 : 1	39	98
12	Rh(NBD)_2_BF_4_	EA : CH_2_Cl_2_ = 10 : 1	87	87
13	Rh(NBD)_2_BF_4_	Toluene : CH_2_Cl_2_ = 10 : 1	99	93
14	Rh(NBD)_2_BF_4_	THF : CH_2_Cl_2_ = 10 : 1	51	99

^*a*^Unless otherwise noted, all reactions were carried out with a [Rh]/ligand **L1**/substrate **1a** (0.1 mmol) ratio of 1 : 1.1 : 100 at 70 °C in 1.0 mL solvent under 50 atm H_2_ for 40 h, and the catalyst was pre-complexed in CH_2_Cl_2_ (0.1 mL for each reaction vial).

^*b*^Determined by ^1^H NMR analysis.

^*c*^Determined by HPLC on a chiral phase.

^*d*^Reaction temperature is 50 °C. NR = no reaction, NA = not available. DCE is dichloroethane. TFE is CF_3_CH_2_OH. EA is ethyl acetate. THF is tetrahydrofuran.

**Fig. 1:**
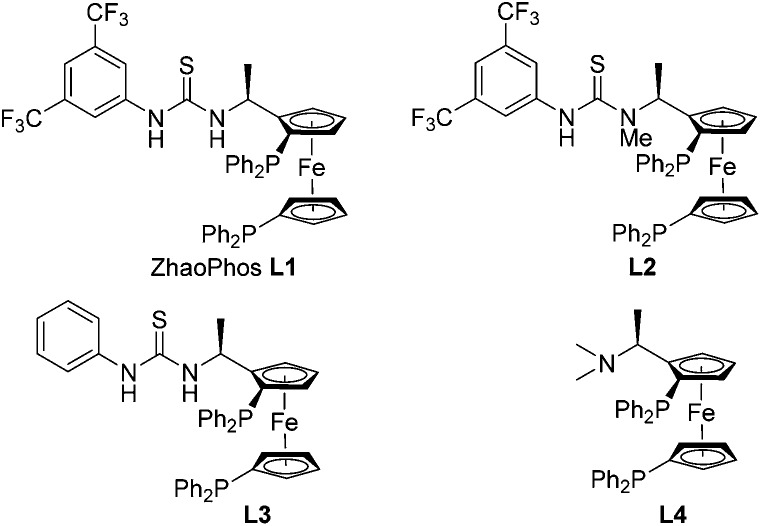
The structure of bisphosphine ligands.

A series of bisphosphine-thiourea ligands were then investigated in this Rh-catalyzed asymmetric hydrogenation (Fig. 1). As shown in Table 2, ZhaoPhos ligand **L1** and *N*-methylated ZhaoPhos ligand **L2** provided the same result with >99% conversion and >99% ee (Table 2, entries 1 and 2), which indicates that one hydrogen bond is sufficient to obtain high reactivity and excellent enantioselectivity in this asymmetric transformation. The ligand **L3** without the CF_3_ group on the phenyl ring provided poor results (73% conversion, 56% ee, Table 2, entry 3). In addition, no reaction was observed using ligand **L4** without the thiourea group, which showed that the possible hydrogen bonding interaction between the ligand and the sulfonyl group of the substrate was essential to achieve high reactivity and excellent enantioselectivity. In order to obtain the optimal ligand, this Rh-catalyzed asymmetric hydrogenation was conducted in the presence of ZhaoPhos ligand **L1** and *N*-methylated ZhaoPhos ligand **L2** with a lower catalyst loading (0.5 mol%). We found that ligand **L2** provided better results than ligand **L1** (95% conversion, 98% ee, Table 2, entry 6).

**Table 2:** Screening bisphosphine ligands for asymmetric hydrogenation of 2-phenylbenzo[*b*]thiophene 1,1-dioxide **1a**^*a*^

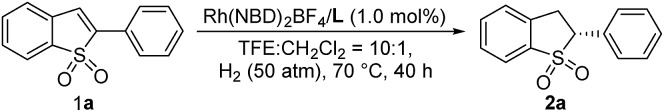			
Entry	Ligand	Conv.^*b*^ [%]	ee^*c*^ [%]
1	ZhaoPhos **L1**	>99	>99
2	**L2**	>99	>99
3	**L3**	73	56
4	**L4**	NR	NA
5^*d*^	ZhaoPhos **L1**	81	96
6^*d*^	**L2**	95	98

^*a*^Unless otherwise mentioned, all reactions were carried out with a [Rh(NBD)_2_]BF_4_/ligand/substrate **1a** (0.1 mmol) ratio of 1 : 1.1 : 100 in 1.0 mL CF_3_CH_2_OH under 50 atm H_2_ at 70 °C for 40 h, and the catalyst was pre-complexed in CH_2_Cl_2_ (0.1 mL for each reaction vial).

^*b*^Determined by ^1^H NMR analysis.

^*c*^The ee value was determined by HPLC on a chiral phase.

^*d*^Catalyst loading is 0.5 mol%, 12 h. NR = no reaction, NA = not available.

Under the optimized reaction conditions, the substrate scope of Rh-catalyzed asymmetric hydrogenation of prochiral substituted benzo[*b*]thiophene 1,1-dioxides was explored, and the results are summarized in Table 3. A wide range of 2-substituted benzo[*b*]thiophene 1,1-dioxides were hydrogenated smoothly catalyzed by Rh(NBD)_2_BF_4_/**L2**. When the 2-substituted benzo[*b*]thiophene 1,1-dioxides bearing the electron-donating group (**1b** and **1d–1f**) or electron-withdrawing group on the phenyl ring (**1c** and **1g**) were used, the corresponding hydrogenation products chiral 2-substituted 2,3-dihydro-benzo[*b*]thiophene 1,1-dioxides (**2b–2g**) were obtained with full conversions, high yields and excellent enantioselectivities (>99% conversion, 98–99% yields, 96–>99% ee). And the position of the substituent on the phenyl ring had little effect on the reactivity and enantioselectivity. To our delight, 2-substituted benzo[*b*]thiophene 1,1-dioxides with an *ortho*- (**1d**) or *meta*- (**1e** and **1g**) substituted group on the phenyl ring with steric hindrance were hydrogenated smoothly with excellent results (>99% conversion, 98% yield and 97–98% ee). The asymmetric hydrogenation of the substrate with a bulky 2-naphthyl group also proceeded efficiently to afford the product (**2h**) with >99% conversion, 98% yield and 95% ee. Noticeably, the alkyl substituted benzo[*b*]thiophene 1,1-dioxides (**1i–1l**) were also hydrogenated well in our catalytic system, providing the desired products (**2i–2l**) with >99% conversion, 98–99% yields and 83–92% ee.

**Table 3:** Scope study of the Rh-catalyzed asymmetric hydrogenation of 2-substituted benzo[*b*]thiophene 1,1-dioxides^*a*^

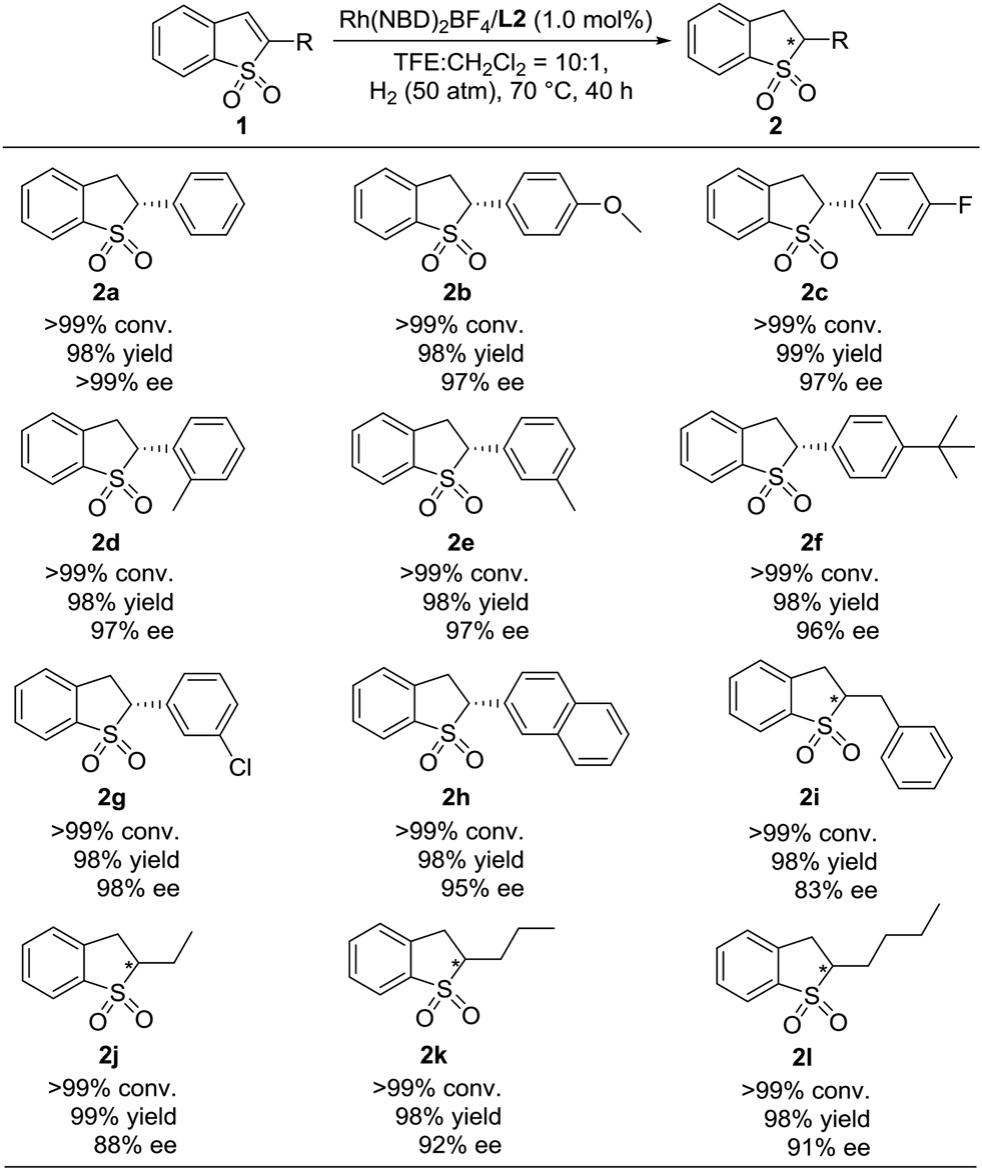

^*a*^0.1 mmol substrate **1**, substrate **1**/Rh(NBD)_2_BF_4_/**L2** = 1/0.01/0.011 at 70 °C under 50 atm H_2_ in 1.0 mL CF_3_CH_2_OH for 40 h, and the catalyst was pre-complexed in CH_2_Cl_2_ (0.1 mL for each reaction vial). Conversion was determined by ^1^H NMR analysis. Yield is isolated yield. The ee value was determined by HPLC on a chiral column. The absolute configuration of **2a** was determined as (*R*) according to previous work.7*c*

Encouraged by the success in the highly enantioselective hydrogenation of various 2-substituted benzo[*b*]thiophene 1,1-dioxides catalyzed by Rh(NBD)_2_BF_4_/**L2**, we turned our attention to investigate the substrate generality of 3-substituted benzo[*b*]thiophene 1,1-dioxides. As shown in Table 4, a variety of 3-substituted benzo[*b*]thiophene 1,1-dioxides were reduced efficiently, providing the desired hydrogenation products (**2m–2x**) with excellent results (>99% conversion, 97–99% yields, and 94–>99% ee). We found that the electronic properties and position of the substituted group on the phenyl ring of 3-aromatic substituted benzo[*b*]thiophene 1,1-dioxides have little influence on the reactivity and enantioselectivity. In addition, the substrate (**1u**) with a bulky 2-naphthyl group also worked well to afford the product (**2u**) with >99% conversion, 99% yield and >99% ee. Furthermore, when the 3-substituted aromatic group was changed to an alkyl group, the Rh-catalyzed asymmetric hydrogenation of 3-alkyl substituted benzo[*b*]thiophene 1,1-dioxides (**1v–1x**) proceeded smoothly with excellent results (>99% conversion, 97–98% yields, and 96–98% ee).

**Table 4:** Scope study of the Rh-catalyzed asymmetric hydrogenation of 3-substituted benzo[*b*]thiophene 1,1-dioxides^*a*^

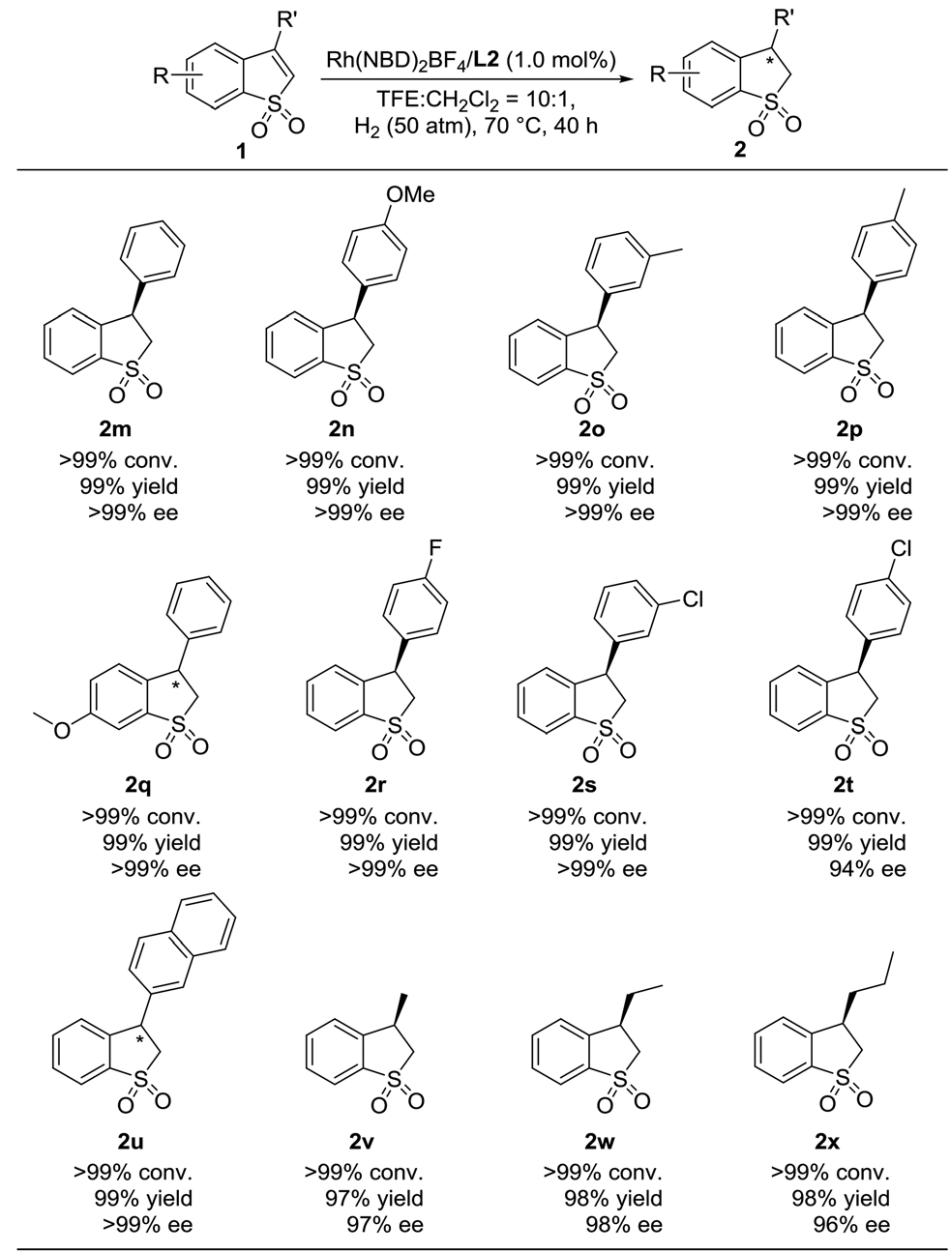

^*a*^0.1 mmol substrate **1**, substrate **1**/Rh(NBD)_2_BF_4_/**L2** = 1/0.01/0.011 at 70 °C under 50 atm H_2_ in 1.0 mL CF_3_CH_2_OH for 40 h, and the catalyst was pre-complexed in CH_2_Cl_2_ (0.1 mL for each reaction vial). Conversion was determined by ^1^H NMR analysis. Yield is isolated yield. The ee value was determined by HPLC on a chiral column. The absolute configurations of **2n** and **2v** were determined as (*R*) according to previous work.7*c*

**Scheme 2:**
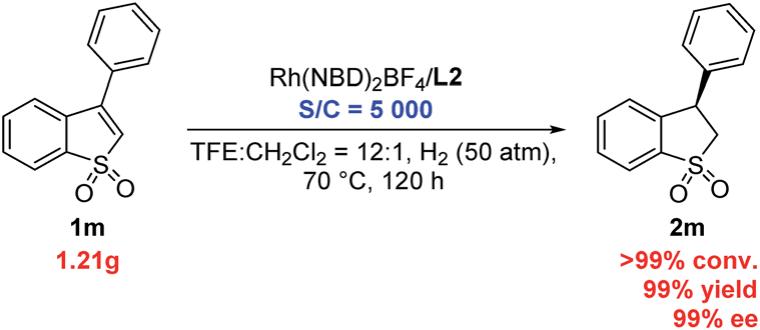
Gram-scale asymmetric hydrogenation with high TON.

In addition, the gram-scale asymmetric hydrogenation of 3-phenyl benzo[*b*]thiophene 1,1-dioxide (**1m**) proceeded efficiently with only 0.02 mol% (S/C = 5000) catalyst, affording the desired product (**2m**) with >99% conversion, 99% yield and 99% ee (Scheme 2). This result showed that this Rh/ligand **L2** catalytic system possessed very high activity in this reaction.

**Scheme 3:**
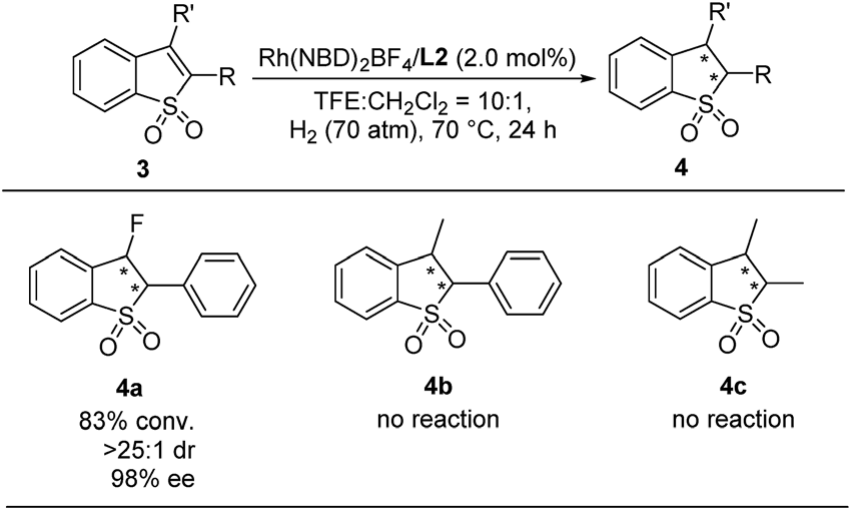
Rh-catalyzed asymmetric hydrogenation of 2,3-disubstituted benzo[*b*]thiophene 1,1-dioxides.

It is very challenging to realize the asymmetric hydrogenation of tetrasubstituted cyclic olefins owing to their unfavorable bulky steric hindrance. The tetrasubstituted cyclic olefins 2,3-disubstituted benzo[*b*]thiophene 1,1-dioxides were applied in this Rh-catalyzed asymmetric hydrogenation to further investigate the substrate generality. As shown in Scheme 3, the desired product 3-fluoro-2-phenyl-2,3-dihydrobenzo[*b*]thiophene 1,1-dioxide (**4a**) can be obtained with good conversion, high diastereoselectivity and excellent enantioselectivity (83% conversion, >25 : 1 dr, and 98% ee). In addition, no reaction was detected with more challenging substrates, 3-methyl-2-phenylbenzo[*b*]thiophene 1,1-dioxide (**3b**) and 2,3-dimethylbenzo[*b*]thiophene 1,1-dioxide (**3c**).

A nonlinear effect suggests that the potential dimerization or high-order aggregation of catalysts should exist in catalytic asymmetric reactions.^14^ In order to verify the possible catalytic model, the asymmetric hydrogenation of substrate **1m** was performed in the presence of ligand **L2** with different ee values. And no nonlinear effect was observed in this transformation, which revealed that there should be no catalyst self-aggregation or ligand–substrate agglomeration in this catalytic system. Furthermore, a Job plot was drawn and the curve suggests a 1 : 1 binding pattern between ligand **L2** and substrate **1m**. On the basis of these observations and the reaction results, 3D catalytic models for the asymmetric hydrogenation of substrates **1a** and **1m** were built through DFT calculations to account for the possible hydrogen bonding interaction between the Rh-catalyst and the substrate (summarized in the ESI†).

## Conclusions

In summary, a highly efficient synthetic methodology for the construction of various chiral 2,3-dihydro-benzo[*b*]thiophene 1,1-dioxides was successfully developed through Rh/*N*-methylated ZhaoPhos ligand **L2**-catalyzed asymmetric hydrogenation. Our catalytic system possessed wide tolerance of substrate scope, both aromatic and alkyl substituted groups at the 2-position or the 3-position of prochiral benzo[*b*]thiophene 1,1-dioxides worked well in this asymmetric hydrogenation to provide the desired products with high yields and excellent enantioselectivities (up to 99% yield and >99% ee). In addition, our catalytic system showed very high activity, and the gram-scale asymmetric hydrogenation of 3-phenyl benzo[*b*]thiophene 1,1-dioxide proceeded well catalyzed by only 0.02 mol% (S/C = 5000) Rh/ligand **L2** catalyst loading with >99% conversion, 99% yield and 99% ee. The possible hydrogen-bonding interaction between the substrate and the thiourea motif of the ligand may make an important contribution to achieving high reactivity and excellent enantioselectivity in this reaction. Further investigations toward a catalytic asymmetric variant of this reaction process are under way.

## Conflicts of interest

There are no conflicts to declare.

## Supplementary Material

Supplementary information
