# Supplementary material for: Efficient synthesis of chiral 2,3-dihydro-benzo[b]thiophene 1,1-dioxides via Rh-catalyzed hydrogenation
Source: Chem Sci. 2019 Jan 15;10(8):2507–12. doi: 10.1039/c8sc05397a (PMC6385876; doi:10.1039/c8sc05397a)
Supplement: Supplementary file 1 [file SC-010-C8SC05397A-s001.pdf]

Electronic Supplementary Material (ESI) for Chemical Science.
This journal is © The Royal Society of Chemistry 2019

Supporting information

Efficient Synthesis of Chiral 2,3-Dihydro-benzo[*b*]thiophene 1,1-Dioxides *via* Rh-Catalyzed Hydrogenation

Gongyi Liu,† Heng Zhang,† Yi Huang,† Zhengyu Han,† Gang Liu,† Yuanhua Liu,† Xiu-Qin Dong,*† and Xumu Zhang*§,†

† Key Laboratory of Biomedical Polymers, Engineering Research Center of Organosilicon Compounds & Materials, Ministry of Education, College of Chemistry and Molecular Sciences, Wuhan University, Wuhan, Hubei, 430072, P. R. China.

§ Department of Chemistry and Shenzhen Grubbs Institute, Southern University of Science and Technology, Shenzhen, Guangdong, 518055, P. R. China.

Contents

1. General remarks……………………………………………………………..2

2. General procedure for the synthesis of substrate……….……………………...2

3. General procedure for asymmetric hydrogenation……………………..……..17

4. General procedure for the high TON experiment…….……………………..……...31

5. General procedure for Job plot…….…………………..……..............................31

6. General computational calculation (DFT) details…………..……......................33

7. Reference…………………………………………………………………..…….52

8. NMR spectra…………………….………...…..……………………………….54

9. HPLC spectra……………….…………………………..………………...106

1. General remarks

Unless otherwise noted, all reagents and solvents were purchased from commercial suppliers and used without further purification. Anhydrous solvents were purchased from Sigma-Aldrich, J&K Chemical Technology company, and transferred by syringe. ^1H NMR and ^{13}C NMR spectra were recorded on a Bruker ADVANCE III (400 MHz) spectrometer with $CDCl_3$ as the solvent and tetramethylsilane (TMS) as the internal standard. Chemical shifts are reported in parts per million (ppm, δ scale) downfield from TMS at 0.00 ppm and referenced to the $CDCl_3$ at 7.26 ppm (for ^1H NMR) or 77.0 ppm (for ^{13}C NMR). Data are reported as: multiplicity (s = singlet, d = doublet, t = triplet, q = quartet, m = multiplet), coupling constant in hertz (Hz) and signal area integration in natural numbers. ^{13}C NMR analyses were run with decoupling. Enantiomeric excess values were determined by Daicel chiral column on an Agilent 1260 Series HPLC instrument. Optical rotations $[\alpha]_D$ were measured on a PERKIN ELMER polarimeter 343 instrucment. Column Chromatography was performed with silica gel (300-400 mesh).

2. General procedure for the synthesis of substrate

The substrates of aryl-substituted benzothiophene 1,1-dioxides according were synthesized to the method A:

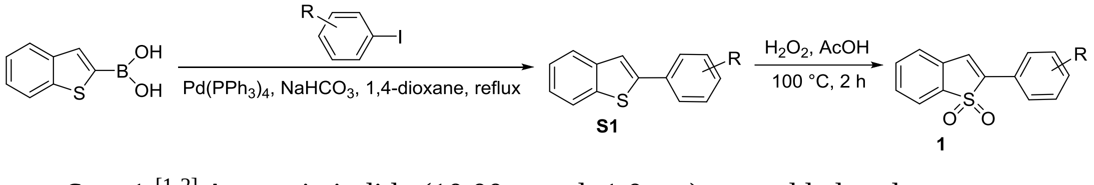

Step 1:[1-2] Aromatic iodide (10.00 mmol, 1.0 eq.) was added under argon at r.t. to a solution of $Pd(Ph_3P)_4$ (58 mg, 0.05 mmol, 0.005 eq.) in dry 1,4-dioxane (50 mL) and stirred for 20 min. After this time saturated $NaHCO_3$ (aq., 14.0 mL) and commercially available 1-benzothiophene-2-boronic acid (979 mg, 11.0 mmol, 1.1 eq.) were added and the resulting reaction mixture was refluxed overnight. The reaction mixture was then allowed to cool to r.t. and diluted with H_2O (80 mL) and extracted with CH_2Cl_2 (3×60 mL). The combined organic extracts were washed with NaOH (aq. 1 M, 40 mL)

and then with brine, dried over $MgSO_4$ and concentrated in vacuo to give a brown crystalline solid that was purified by flash chromatography to give the corresponding product **S1**.

Step 2:[3] **S1** (1.00 eq.) was suspended in glacial AcOH (concentration ≈ 0.9 M) and H_2O_2 (30% aq. solution, 6.5 eq.) was slowly added. The mixture was heated to 100 °C and, at this temperature, it became a clear solution. Stirring at 100 °C was continued for 3 h and GC-MS analysis showed full conversion. The mixture was allowed to cool to r.t. and partitioned between CH_2Cl_2 and saturated $NaHCO_3$ (aq. so to bring the pH of the aq. phase to ≈ 9), the layers were separated and the aq. phase was extracted three times with CH_2Cl_2. The combined organic extracts were dried over $MgSO_4$ and concentrated in vacuo to give the crude product that was purified by flash chromatography (silica gel, petroleum ether–EtOAc).

2-phenylbenzo[*b*]thiophene 1,1-dioxide **1a**

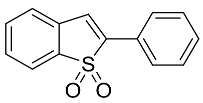

White crystalline solid; ^{1}H NMR (400 MHz, $CDCl_3$) δ 7.85-7.83 (m, 2H), 7.77 (d, *J* = 7.6 Hz, 1H), 7.57 (t, *J* = 7.6 Hz, 1H), 7.52-7.45 (m, 4H), 7.44-7.40 (m, 1H), 7.29 (s, 1H); ^{13}C NMR (100 MHz, $CDCl_3$) δ 142.54, 137.02, 133.76, 131.10, 130.34, 129.91, 129.22, 127.10, 126.52, 125.02, 123.66, 121.48. The characterization data of compound **1a** is in accordance with the reported data in the literature.[2]

2-(4-methoxyphenyl)benzo[*b*]thiophene 1,1-dioxide **1b**

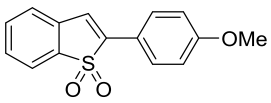

Yellow crystalline solid; ^{1}H NMR (400 MHz, $CDCl_3$) δ (ppm) 7.79-7.77 (m, 2H), 7.74 (d, *J* = 7.6 Hz, 1H), 7.54 (t, *J* = 7.6 Hz, 1H), 7.45 (t, *J* = 7.6 Hz, 1H), 7.36 (d, *J* = 7.6 Hz, 1H), 7.15 (s, 1H), 7.00-6.97 (m, 2H), 3.85 (s, 3H); ^{13}C NMR (100 MHz, $CDCl_3$) δ 161.19, 142.23, 136.70, 133.72, 131.53, 129.36, 128.10, 124.66, 121.38, 121.25, 119.54, 114.72, 55.42. The characterization data of compound **1b** is in

accordance with the reported data in the literature.[2]

2-(4-fluorophenyl)benzo[*b*]thiophene 1,1-dioxide **1c**

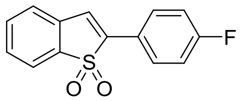

Pale yellow crystalline solid; ^{1}H NMR (400 MHz, $CDCl_3$) δ 7.85-7.81 (m, 2H), 7.76 (d, *J* = 7.6 Hz, 1H), 7.60-7.56 (m, 1H), 7.51 (t, *J* = 7.6 Hz, 1H), 7.40 (d, *J* = 7.6 Hz, 1H), 7.23 (s, 1H), 7.20-7.15 (m, 2H); ^{13}C NMR (100 MHz, $CDCl_3$) δ 163.77 (d, *J* = 251.0 Hz), 141.54, 136.76, 133.85, 131.00, 129.97, 128.65 (d, *J* = 9.0 Hz), 125.03, 123.51 (d, *J* = 2.0 Hz), 123.36 (d, *J* = 4.0 Hz), 121.54, 116.52 (d, *J* = 22.0 Hz). The characterization data of compound **1c** is in accordance with the reported data in the literature.[2]

2-(*o*-tolyl)benzo[*b*]thiophene 1,1-dioxide **1d**

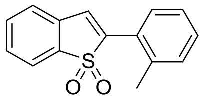

White crystalline solid; ^{1}H NMR (400 MHz, $CDCl_3$) δ 7.77 (d, *J* = 7.2 Hz, 1H), 7.69 (d, *J* = 7.6 Hz, 1H), 7.60-7.56 (m, 1H), 7.53-7.49 (m, 1H), 7.41-7.39 (m, 1H), 7.36-7.27 (m, 3H), 7.04 (s, 1H), 2.45 (s, 3H); ^{13}C NMR (100 MHz, $CDCl_3$) δ 142.59, 138.06, 136.29, 133.66, 131.06, 130.09, 130.06, 129.47, 128.18, 126.40, 126.19, 125.00, 121.70, 20.67. The characterization data of compound **1d** is in accordance with the reported data in the literature.[2]

2-(*m*-tolyl)benzo[*b*]thiophene 1,1-dioxide **1e**

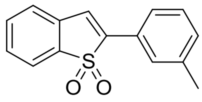

White crystalline solid; ^{1}H NMR (400 MHz, $CDCl_3$) δ 7.76 (d, *J* = 7.6 Hz, 1H), 7.65-7.62 (m, 2H), 7.58-7.51 (m, 1H), 7.49-7.47 (m, 1H), 7.40-7.34 (m, 2H), 7.27 (s, 2H), 2.41 (s, 3H); ^{13}C NMR (100 MHz, $CDCl_3$) δ 142.69, 139.02, 137.04, 133.72, 131.22, 129.81, 129.10, 127.00, 124.94, 123.71, 123.50, 121.46, 21.46. The

characterization data of compound **1e** is in accordance with the reported data in the literature.[2]

2-(4-(tert-butyl)phenyl)benzo[*b*]thiophene 1,1-dioxide **1f**

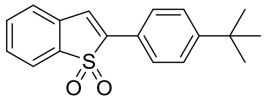

White crystalline solid; ^{1}H NMR (400 MHz, $CDCl_3$) δ 7.79-7.75 (m, 3H), 7.59-7.55 (m, 1H), 7.51-7.47 (m, 3H), 7.40 (d, *J* = 7.2 Hz, 1H), 7.25 (s, 1H), 1.35 (s, 9H); ^{13}C NMR (100 MHz, $CDCl_3$) δ 153.85, 142.60, 136.98, 133.72, 131.37, 129.67, 126.30, 126.26, 124.86, 124.21, 122.64, 121.48, 34.93, 31.09. HRMS calculated for $C_{18}H_{18}NaO_2S^+$ [$(M+Na)^+$] = 321.0922, found: 321.0919.

2-(3-chlorophenyl)benzo[*b*]thiophene 1,1-dioxide **1g**

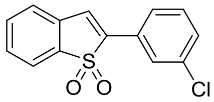

White crystalline solid; ^{1}H NMR (400 MHz, $CDCl_3$) δ 7.78-7.73 (m, 3H), 7.61-7.57 (m, 1H), 7.57-7.51 (m, 1H), 7.44-7.42 (m, 3H), 7.32 (s, 1H); ^{13}C NMR (100 MHz, $CDCl_3$) δ 141.24, 136.97, 135.24, 133.89, 130.70, 130.49, 130.37, 130.33, 128.80, 126.46, 125.30, 124.99, 124.66, 121.59. HRMS calculated for $C_{14}H_9ClNaO_2S^+$ [$(M+Na)^+$] = 298.9907, found: 298.9903.

2-(naphthalen-2-yl)benzo[*b*]thiophene 1,1-dioxide **1h**

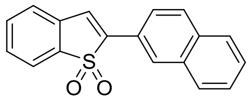

White crystalline solid; ^{1}H NMR (400 MHz, $CDCl_3$) δ 8.15 (s, 1H), 7.91-7.80 (m, 6H), 7.68 (s, 1H), 7.54-7.47 (m, 2H), 7.39-7.31 (m, 2H); ^{13}C NMR (100 MHz, $CDCl_3$) δ 142.56, 137.08, 133.81, 133.10, 131.24, 129.89, 129.18, 128.97, 127.72, 127.56, 126.96, 126.73, 125.03, 124.24, 123.65, 123.05, 121.52. HRMS calculated for $C_{18}H_{12}NaO_2S^+$ [$(M+Na)^+$] = 315.0456, found: 315.0450.

3-phenylbenzo[*b*]thiophene 1,1-dioxide **1m**

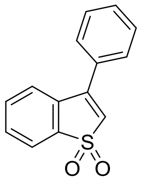

White crystalline solid; ^{1}H NMR (400 MHz, $CDCl_3$) δ 7.83-7.79 (m, 1H), 7.60-7.48 (m, 8H), 6.65 (s, 1H); ^{13}C NMR (100 MHz, $CDCl_3$) δ 145.95, 138.08, 133.29, 131.90, 130.99, 130.59, 130.47, 129.16, 127.95, 125.60, 124.28, 121.62. The characterization data of compound **1m** is in accordance with the reported data in the literature.[2]

3-(4-methoxyphenyl)benzo[*b*]thiophene 1,1-dioxide **1n**

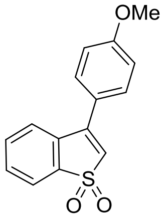

White crystalline solid; ^{1}H NMR (400 MHz, $CDCl_3$) δ 7.81-7.79 (m, 1H), 7.59-7.53 (m, 3H), 7.50-7.47 (m, 2H), 7.05-7.03 (m, 2H), 6.58 (s, 1H), 3.89 (s, 3H); ^{13}C NMR (100 MHz, $CDCl_3$) δ 161.35, 145.60, 138.24, 133.17, 132.06, 130.44, 129.47, 124.25, 124.22, 123.14, 121.50, 114.55, 55.43. The characterization data of compound **1n** is in accordance with the reported data in the literature.[2]

3-(*m*-tolyl)benzo[*b*]thiophene 1,1-dioxide **1o**

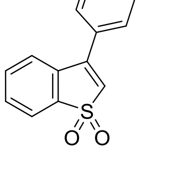

White crystalline solid; ^{1}H NMR (400 MHz, $CDCl_3$) δ 7.82-7.79 (m, 1H), 7.59-7.55 (m, 2H), 7.53-7.49 (m, 1H), 7.42 (t, *J* = 7.6 Hz, 1H), 7.33 (t, *J* = 7.2 Hz, 3H), 6.63 (s, 1H), 2.44 (s, 3H); ^{13}C NMR (100 MHz, $CDCl_3$) δ 146.12, 139.07, 138.10, 133.26, 132.01, 131.23, 130.94, 130.53, 129.01, 128.51, 125.39, 125.03,

124.34, 121.57, 21.45. The characterization data of compound **1o** is in accordance with the reported data in the literature.[2]

3-(*p*-tolyl)benzo[*b*]thiophene 1,1-dioxide **1p**

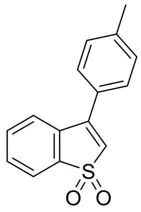

White crystalline solid; ^{1}H NMR (400 MHz, $CDCl_3$) δ 7.81-7.79 (m, 1H), 7.58-7.56 (m, 2H), 7.53-7.50 (m, 1H), 7.42 (d, *J* = 7.6 Hz, 2H), 7.33 (d, *J* = 8.0 Hz, 2H), 6.62 (s, 1H), 2.45 (s, 3H); ^{13}C NMR (100 MHz, $CDCl_3$) δ 145.98, 140.86, 138.16, 133.22, 132.03, 130.49, 129.82, 128.06, 127.88, 124.96, 124.29, 121.54, 21.44. The characterization data of compound **1p** is in accordance with the reported data in the literature.[2]

3-(4-fluorophenyl)benzo[*b*]thiophene 1,1-dioxide **1r**

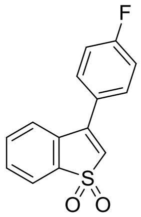

White crystalline solid; ^{1}H NMR (400 MHz, $CDCl_3$) δ 7.82-7.80 (m, 1H), 7.60–7.58 (m, 2H), 7.54-7.46 (m, 2H), 7.47-7.46 (m, 1H), 7.25–7.21 (m, 2H), 6.64 (s, 1H); ^{13}C NMR (100 MHz, $CDCl_3$) δ 163.91 (d, *J* = 250.0 Hz), 144.91, 138.07, 133.35, 131.74, 130.72, 129.98 (d, *J*= 8.0 Hz), 127.03 (d, *J* = 3.0 Hz), 125.77, 124.06, 121.71, 116.44 (d, *J* = 21.0 Hz). The characterization data of compound **1r** is in accordance with the reported data in the literature.[2]

3-(3-chlorophenyl)benzo[*b*]thiophene 1,1-dioxide **1s**

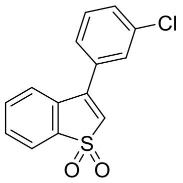

White crystalline solid; ^{1}H NMR (400 MHz, $CDCl_3$) δ (ppm) 7.81-7.80 (m, 1H), 7.61-7.59 (m, 2H), 7.52-7.40 (m, 5H), 6.68 (s, 1H); ^{13}C NMR (100 MHz, $CDCl_3$) δ (ppm) 144.51, 137.93, 135.23, 133.44, 132.72, 131.39, 130.83, 130.55, 130.53, 127.97, 126.53, 126.12, 124.03, 121.79. HRMS calculated for $C_{14}H_9ClNaO_2S^+$ [(M+Na) $^+$] = 298.9907, found: 298.9903.

3-(4-chlorophenyl)benzo[*b*]thiophene 1,1-dioxide **1t**

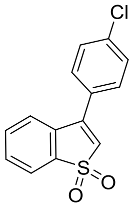

White crystalline solid; ^{1}H NMR (400 MHz, $CDCl_3$) δ (ppm) 7.82-7.80 (m, 1H), 7.60-7.58 (m, 2H), 7.53-7.44 (m, 5H), 6.66 (s, 1H); ^{13}C NMR (100 MHz, $CDCl_3$) δ (ppm) 144.79, 138.02, 136.68, 133.38, 131.53, 130.78, 129.52, 129.40, 129.28, 126.04, 124.02, 121.76. HRMS calculated for $C_{14}H_9ClNaO_2S^+$ [(M+Na)$^+$] = 298.9908, found: 298.9903.

3-(naphthalen-2-yl)benzo[*b*]thiophene 1,1-dioxide **1u**

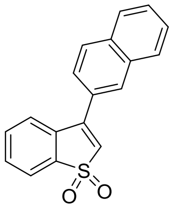

White crystalline solid; ^{1}H NMR (400 MHz, $CDCl_3$) δ (ppm) 8.03 (s, 1H), 7.98 (d, *J* = 4.2 Hz, 1H), 7.93-7.91 (m, 2H), 7.85-7.81 (m, 1H), 7.61-7.55 (m, 6H), 6.74 (s, 1H); ^{13}C NMR (100 MHz, $CDCl_3$) δ (ppm) 145.97, 138.16, 133.93, 133.30, 132.96, 131.99, 130.60, 129.03, 128.39, 128.34, 127.87, 127.56, 127.11, 125.82, 124.81, 124.37, 121.62. HRMS calculated for $C_{18}H_{12}NaO_2S^+$ [(M+Na)$^+$] = 315.0455, found: 315.0450.

The synthesis of substrate 6-methoxy-3-phenylbenzo[*b*]thiophene 1,1-dioxide according to the method B:

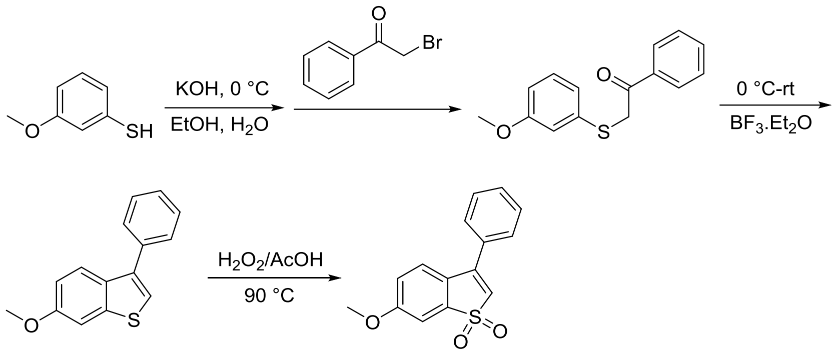

1q

Step 1:[4] Methoxybenzenethiol (1.12 g, 8.0 mmol) was added to a round-bottom flask charged with a freshly prepared solution containing 6 mL ethanol, 14 mL water, and KOH (0.51 g, 9.0 mmol). The solution was cooled in an ice-water bath, and a solution of 2-bromo-1-phenylethan-1-one (1.59 g, 8.0 mmol) in 10 mL ethyl acetate was slowly added. The reaction mixture was monitored by TLC until completion. The solvents were evaporated under reduced pressure, and the residue mixture was partitioned between water and ethyl acetate. The combined organic phase was washed with brine, and dried by Na_2SO_4 to give 2-((4-methoxyphenyl)thio)-1-phenylethan-1-one, which was used in the next step without further purification.

Step 2:[5] $BF_3{\cdot}OEt_2$ (70 mL) was slowly added to a flask charged with 2-((4-methoxyphenyl)thio)-1-phenylethan-1-one (2.0 g) under argon atmosphere in an ice bath. The reaction mixture was stirred until starting material was consumed as monitored by TLC. The reaction mixture was poured into saturated $NaHCO_3$/ice-water, stirred 30 min, and extracted with dichloromethane. The crude product was purified by silica gel chromatography (5% dichloromethane in petroleum ether). The combined fractions from the column were concentrated and recrystallized in ethanol to give pale yellow solid (0.5 g).

Step 3: 5-methoxy-3-phenylbenzo[*b*]thiophene (1.00 eq.) was suspended in glacial AcOH (concentration ≈ 0.9 M) and H_2O_2 (30% aq. solution, 6.5 eq.) was

slowly added. The mixture was heated to 90 °C and, at this temperature, it became a clear solution. Stirring at 90 °C was continued for 3 h and GC-MS analysis showed full conversion. The mixture was allowed to cool to r.t. and partitioned between CH_2Cl_2 and saturated $NaHCO_3$ (aq. so to bring the pH of the aq. phase to ≈ 9), the layers were separated and the aq. phase was extracted three times with CH_2Cl_2. The combined organic extracts were dried over $MgSO_4$ and concentrated in vacuo to give the crude product that was purified by flash chromatography (silica gel, petroleum ether–EtOAc).

6-methoxy-3-phenylbenzo[*b*]thiophene 1,1-dioxide **1q**

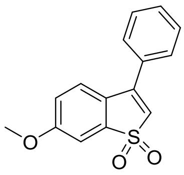

White crystalline solid; ^{1}H NMR (400 MHz, $CDCl_3$) δ (ppm) 7.52 (s, 5H), 7.39 (d, *J* = 8.0 Hz, 1H), 7.34 (d, *J* = 1.2 Hz, 1H), 7.04-7.01 (m, 1H), 6.54 (s, 1H), 3.91 (s, 3H); ^{13}C NMR (100 MHz, $CDCl_3$) δ (ppm) 161.93, 146.24, 140.00, 131.32, 130.40, 129.10, 127.89, 125.42, 123.93, 123.82, 118.48, 107.51, 56.05. HRMS calculated for $C_{15}H_{12}NaO_3S^+$ [$(M+Na)^+$] = 295.0402, found: 295.0399.

The substrates of 2-alkyl-substituted benzothiophene 1,1-dioxides 1i-l according were synthesized to the method C:

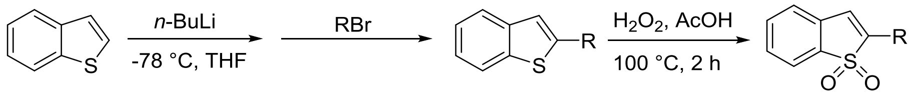

Step 1:[6] To a cold (-78 °C) solution of benzo[*b*]thiophene (2.00 mL, 17.1 mmol, 1.00 eq.) in THF (40 mL) was added *n*-BuLi (1.6 M in hexane, 21.4 mL, 34.3 mmol, 2.00 eq.) dropwise via dropping funnel (over 30 min) under Ar. The dropping funnel was rinsed twice with 5 mL Et_2O. Stirring was continued at -78 °C for 45 min and then the mixture was allowed to slowly warm to r.t. and stirred at this temperature for another 45 min (at r.t. the reaction mixture turned from colorless to yellow). After

this time the reaction mixture was cooled again to -78 °C and bromoalkanes (51.4 mmol, 3.00 eq.) was slowly added *via* dropping funnel. The mixture was stirred for 30 min at -78 °C (formation of a lump of white solid was observed) and then the temperature was slowly raised to 0 °C (vigorous gas evolution and formation of a white suspension). After 20 min of stirring at r.t., an aqueous solution of NaOH (66 mmol in 15 mL of H_2O) was added, the layers were separated and the organic phase was washed with water (10 mL). The combined organic extracts were dried over Na_2SO_4 and concentrated *in vacuo* to give a colorless oil that was purified by flash chromatography (silica gel, petroleum ether) to give 2-alkyl benzo[*b*]thiophene as white crystalline solid.

Step 2: 2-alkyl benzo[*b*]thiophene (1.00 eq.) was suspended in glacial AcOH (concentration ≈ 0.9 M) and H_2O_2 (30% aq. solution, 6.5 eq.) was slowly added. The mixture was heated to 100 °C and, at this temperature, it became a clear solution. Stirring at 100 °C was continued for 3 h and after this time GC-MS analysis showed full conversion. The mixture was allowed to cool to r.t. and partitioned between CH_2Cl_2 and $NaHCO_3$ (aq. sat. so to bring the pH of the aq. phase to ≈ 9), the layers were separated and the aq. phase was extracted three times with CH_2Cl_2. The combined organic extracts were dried over $MgSO_4$ and concentrated in vacuo to give the crude product that was purified by flash chromatography (silica gel, petroleum ether–ethyl acetate).

2-benzylbenzo[*b*]thiophene 1,1-dioxide **1i**

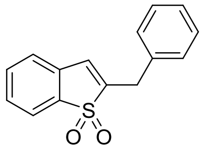

White crystalline solid; ^{1}H NMR (400 MHz, $CDCl_3$) δ 7.72 (d, *J* = 8.0 Hz, 1H), 7.49-7.31 (m, 7H), 7.19 (d, *J* = 8.0 Hz, 1H), 6.41-6.40 (m, 1H), 3.85 (s, 2H); ^{13}C NMR (100 MHz, $CDCl_3$) δ 145.21, 136.84, 134.99, 133.63, 131.42, 129.45, 129.41, 128.95, 127.37, 126.31, 124.52, 121.55, 29.61. The characterization data of compound **1i** is in accordance with the reported data in the literature.[2]

2-ethylbenzo[*b*]thiophene 1,1-dioxide **1j**

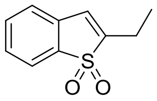

White crystalline solid; ^{1}H NMR (400 MHz, $CDCl_3$) δ 7.70 (d, *J* = 7.6 Hz, 1H), 7.54-7.50 (m, 1H), 7.45-7.41 (m, 1H), 7.29 (d, *J* = 7.6 Hz, 1H), 6.76-6.75 (m, 1H), 2.65-2.59 (m, 2H), 1.35 (t, *J* = 7.6 Hz, 3H); ^{13}C NMR (100 MHz, $CDCl_3$) δ 146.71, 136.77, 133.57, 131.61, 129.30, 124.31, 124.27, 121.43, 17.14, 11.14. The characterization data of compound **1j** is in accordance with the reported data in the literature.[2]

2-propylbenzo[*b*]thiophene 1,1-dioxide **1k**

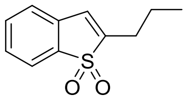

White crystalline solid; ^{1}H NMR (400 MHz, $CDCl_3$) δ 7.69 (d, *J* = 7.6 Hz, 1H), 7.53-7.49 (m, 1H), 7.43 (t, *J* = 7.6 Hz, 1H), 7.28 (d, *J* = 7.6 Hz, 1H), 6.76-6.75 (m, 1H), 2.58-2.53 (m, 2H), 1.81-1.74 (m, 2H), 1.05 (t, *J* = 7.6 Hz, 3H); ^{13}C NMR (100 MHz, $CDCl_3$) δ 145.00, 136.57, 133.51, 131.55, 129.25, 125.07, 124.28, 121.27, 25.82, 20.21, 13.61. The characterization data of compound **1k** is in accordance with the reported data in the literature.[2]

2-butylbenzo[*b*]thiophene 1,1-dioxide **1l**

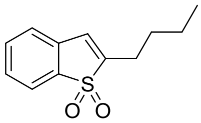

White crystalline solid; ^{1}H NMR (400 MHz, $CDCl_3$) δ 7.68 (d, *J* = 7.2 Hz, 1H), 7.50 (td, *J* = 7.6 Hz, 1.2Hz, 1H), 7.42 (m, *J* = 7.6 Hz, 0.8 Hz, 1H), 7.27 (d, *J* = 5.2 Hz 1H), 6.75 (d, *J* = 0.8 Hz, 1H), 2.59-2.55 (m, 2H), 1.76-1.68 (m, 2H), 1.50-1.41 (m, 2H), 0.96 (t, *J* = 7.6 Hz, 3H); ^{13}C NMR (100 MHz, $CDCl_3$) δ 145.38, 136.65, 133.54, 131.63, 129.27, 124.89, 124.26, 121.38, 28.83, 23.56, 22.25, 13.67. HRMS calculated for $C_{12}H_{14}NaO_2S^+$ [$(M+Na)^+$] = 245.0608, found: 245.0606.

The synthesis of substrates 3-alkyl-substituted benzothiophene 1,1-dioxides 1v-x according to the method D:

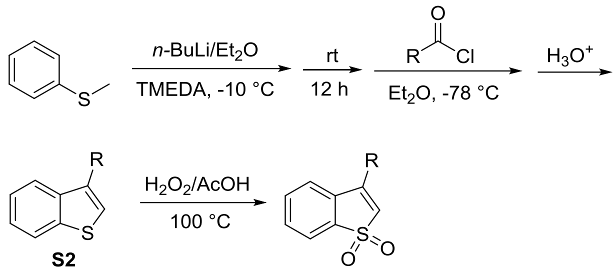

Step 1:[7] To a vigorously stirred solution of methyl(phenyl)sulfane (2.48 g, 20.0 mmol), anhydrous TMEDA (2.28 g, 22.0 mmol), and anhydrous Et_2O (50 mL) cooled to -10 °C, 2.4 M solution of *n*-BuLi in hexane (60 mL) is gradually added under N_2, and stirring is continued at the room temperature for 12 h. The mixture is then cooled to -78 °C and a solution of the acyl chloride (19.0 mmol) in anhydrous Et_2O (10 mL) is gradually (30 min) added under N_2. The resultant mixture is stirred at -78 °C for 1 h, then allowed to warm to room temperature, stirred for 16 h, and poured into H_2O (50 mL). The pH is adjusted to 4-5 by addition of 10% aqueous HCl. The organic layer is separated, and the aqueous layer extracted with Et_2O (2×50 mL). The organic phases are combined, dried by Na_2SO_4, and concentrated. The crude product was purified by silica gel chromatography to obtain **S2**.

Step 2: **S2** (1.00 eq.) was suspended in glacial AcOH (concentration ≈ 0.9 M) and H_2O_2 (30% aq. solution, 6.5 eq.) was slowly added. The mixture was heated to 100 °C and, at this temperature, it became a clear solution. Stirring at 100 °C was continued for 3 h and after this time GC-MS analysis showed full conversion. The mixture was allowed to cool to r.t. and partitioned between CH_2Cl_2 and $NaHCO_3$ (aq. sat. so to bring the pH of the aq. phase to ≈ 9), the layers were separated and the aq. phase was extracted three times with CH_2Cl_2. The combined organic extracts were dried over $MgSO_4$ and concentrated in vacuo to give the crude product that was purified by flash chromatography (silica gel, petroleum ether–EtOAc).

3-methylbenzo[*b*]thiophene 1,1-dioxide **1v**

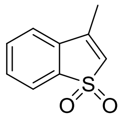

White crystalline solid; ^{1}H NMR (400 MHz, $CDCl_3$) δ 7.73–7.71 (m, 1H), 7.62-7.58 (m, 1H), 7.56-752 (m, 1H), 7.41 (d, J = 7.6 Hz, 1H), 6.48-6.47 (m, 1H), 2.28 (d, J = 1.2 Hz, 3H); ^{13}C NMR (100 MHz, $CDCl_3$) δ 142.83, 137.48, 133.45, 133.07, 130.41, 125.71, 122.23, 120.90, 13.84. The characterization data of compound **1v** is in accordance with the reported data in the literature.[2]

3-ethylbenzo[*b*]thiophene 1,1-dioxide **1w**

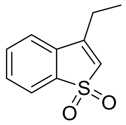

White crystalline solid; ^{1}H NMR (400 MHz, $CDCl_3$) δ (ppm) 7.72-7.70 (m, 1H), 7.60-7.52 (m, 2H), 7.43-7.41 (m, 1H), 6.45-6.44 (m, 1H), 2.67-2.61 (m, 2H), 1.31 (t, J = 7.2 Hz, 3H); ^{13}C NMR (100 MHz, $CDCl_3$) δ (ppm) 148.72, 137.77, 133.39, 132.71, 130.43, 124.04, 122.07, 121.00, 21.15, 10.95. HRMS calculated for $C_{10}H_{10}NaO_2S^+$ [$(M+Na)^+$] = 217.0294, found: 217.0293.

3-propylbenzo[*b*]thiophene 1,1-dioxide **1x**

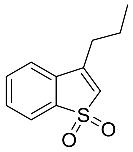

White crystalline solid; ^{1}H NMR (400 MHz, $CDCl_3$) δ (ppm) 7.72-7.70 (m, 1H), 7.60-7.51 (m, 2H), 7.43-7.41 (m, 1H), 6.44-6.43 (m, 1H), 2.60-2.56 (m, 2H), 1.75-1.70 (m, 2H), 1.07 (t, J = 7.2 Hz, 3H); ^{13}C NMR (100 MHz, $CDCl_3$) δ (ppm) 147.04, 137.72, 133.36, 132.76, 130.40, 124.61, 122.19, 121.03, 29.79, 20.12, 13.82. HRMS calculated for $C_{11}H_{12}NaO_2S^+$ [$(M+Na)^+$] = 231.0458, found: 231.0450.

The synthesis of substrate 3-fluoro-2-phenylbenzo[b]thiophene 1,1-dioxide 3a according to the method E:

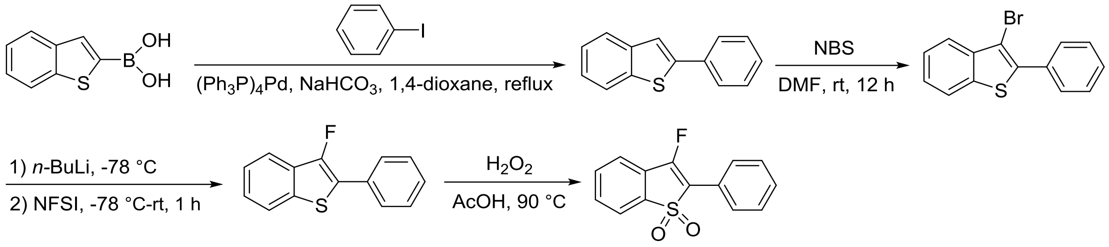

3a

Step 1: The 2-phenylbenzo[*b*]thiophene was synthesized through the procedure according to the method A.

Step 2:[8] 2-phenylbenzo[*b*]thiophene (1.00 g, 4.76 mmol, 1.00 eq.), *N*-bromosuccinimide (933 mg, 5.24 mmol, 1.10 eq.), and DMF (10 mL) were combined in a 50 mL round-bottom flask and allowed to stir at room temperature for 12 h. Purification of the crude reaction mixture by filtration through a silica gel plug, eluting with ether, followed by recrystallization of the resulting solid from MeOH, provided 3-bromo-2-phenylbenzo[*b*]thiophene as a pale yellow solid.

Step 3:[8] 3-bromo-2-phenylbenzo[*b*]thiophene (578 mg, 2 mmol, 1.0 eq.), *n*-BuLi (2.5 M in hexanes, 0.9 mL, 2.2 mmol, 1.10 eq.), NFSI (758 mg, 2.4 mmol, 1.20 eq.), and THF (15 mL) were combined and allowed to stir at room temperature for 1 h. The 3-fluoro-2-phenylbenzo[*b*]thiophene was purified by flash chromatography (340 mg, 74% yield) as a white solid. ^{1}H NMR (400 MHz, $CDCl_3$) δ (ppm) 7.78-7.74 (m, 4H), 7.47-7.34 (m, 5H). The characterization data of compound is in accordance with the reported data in the literature. [8]

Step 4：The substrate **3a** was synthesized through oxidation reaction in the method A: White crystalline solid; ^{1}H NMR (400 MHz, $CDCl_3$) δ (ppm) 7.91-7.89 (m, 2H), 7.83-7.81 (m, 1H), 7.69-7.66 (m, 1H), 7.63-7.59 (m, 2H), 7.53-7.43 (m, 3H); ^{13}C NMR (100 MHz, $CDCl_3$) δ (ppm) 155.96 (J = 290.0 Hz), 137.50 (J = 3.0 Hz), 133.72, 131.15, 129.96 (J = 2.0 Hz), 129.20, 127.54 (J = 5.0 Hz), 126.86 (J = 28.0 Hz), 124.47 (J = 4.0 Hz), 121.18 (J = 3.0 Hz), 120.81 (J = 6.0 Hz), 120.53. HRMS calculated for $C_{14}H_9FNaO_2S^+$ [$(M+Na)^+$] = 283.0197, found:281.0199.

The synthesis of substrate 3-methyl-2-phenylbenzo[*b*]thiophene 1,1-dioxide 3b:

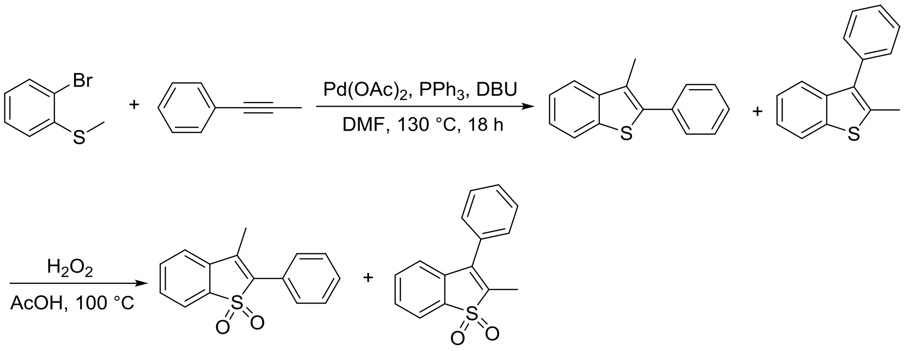

3b **3b'**

Step 1:[9] An oven-dried 50 mL screw-capped vial was charged with (2-bromophenyl)(methyl)sulfane (1.217 g, 6.0 mmol), prop-1-yn-1-ylbenzene (1.043 g, 9.0 mmol), $Pd(OAc)_2$ (133.89 mg, 0.6 mmol), PPh_3 (480 mg, 1.8 mmol), DBU (2.736 g, 18 mmol), and DMF (18 mL) under a gentle stream of nitrogen. The vessel was then sealed and heated at 130 °C for 18 h. The mixture was cooled to rt and filtered through a short pad of silica gel, eluting with EtOAc.

Step 2: The substrate **3b** was synthesized through oxidation reaction according to the method A: The mixtures of compounds **3b** and **3b'** were recrystallized several times in ethanol to get pure compound **3b**. White crystalline solid; ^{1}H NMR (400 MHz, $CDCl_3$) δ (ppm) 7.78 (d, J = 7.4 Hz, 1H), 7.66-7.60 (m, 3H), 7.55-7.46 (m, 5H), 2.28 (s, 3H); ^{13}C NMR (100 MHz, $CDCl_3$) δ (ppm) 137.42, 136.01, 135.38, 133.56, 133.37, 129.91, 129.75, 129.36, 128.97, 127.03, 122.26, 121.04, 12.01. HRMS calculated for $C_{15}H_{13}O_2S^+$ [$(M+H)^+$] = 257.0635, found: 257.0630.

The synthesis of substrate 2,3-dimethylbenzo[*b*]thiophene 1,1-dioxide 3c:

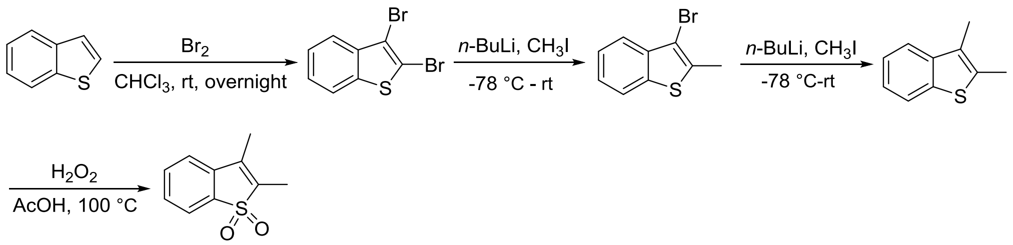

3c

Step 1:[10] To a stirred solution of of 2,3-benzo[*b*]thiophene (1.01 g, 7.5 mmol, 1.0 eq.) in 20 mL $CHCl_3$, bromine (0.8 mL, 15 mmol, 2.0 eq.) in 20 mL $CHCl_3$ was added dropwise at room temperature over 1 h. After stirring for 18 h in the dark, solid

$NaHCO_3$ was added to neutralize the hydrobromic acid until gas evolution ceased. The organic phase was washed with water and 10% aqueous $Na_2S_2O_3$ solution, extracted with $CHCl_3$, and dried over $MgSO_4$. After evaporation of the solvent, crystallization from methanol gave 2,3-dibromobenzo[*b*]thiophene (2.03 g, 91% yield) as a white solid.

Step 2:[10] 2,3-dibromobenzo[*b*]thiophene (2.03 g, 7.0 mmol, 1.0 eq.) was dissolved in 10 mL dry degassed THF under argon. The solution was cooled to -78°C before *n*-BuLi (4.8 mL, 7.7 mmol, 1.6 M in hexane, 1.1 eq.) was added, and the mixture was stirred for 30 min before iodomethane (0.5 mL, 7.9 mmol, 1.1 eq.) was added. The mixture was allowed to warm to room temperature, washed with water, extracted with ethyl acetate and dried over $MgSO_4$. A short column chromatography (silica, petroleum ether 100%) gave the product as white solid.

Step 3:[11] 3-Bromo-2-methylbenzothiophene (1.0 g, 4.4 mmol) was dissolved in THF (25 mL). 3 mL *n*-BuLi (1.6 M in hexane, 4.8 mmol) was added slowly with stirring at -78 °C. Reaction mixture was stirred for 30 min and then methyl iodide (4.8 mmol) was added. After stirring for 1h, the mixture was warmed to room temperature and stirred for 2 h at room temperature. After completion of reaction, H_2O was added. The mixture was extracted with CH_2Cl_2 (2 x 25 mL), dried over $MgSO_4$, filtered and the solvents were removed. The residue was purified by chromatography on silica gel to give 2 ,3-dimethylbenzo[*b*]thiophene (642 mg, 90 % yield).

Step 4: The substrate **3c** according was synthesized through oxidation reaction according to the method A: White crystalline solid; ^{1}H NMR (400 MHz, $CDCl_3$) δ (ppm) 7.72 (d, *J* = 7.4 Hz, 1H), 7.58-7.54 (m, 1H), 7.48-7.44 (m, 1H), 7.36 (d, *J* = 7.6 Hz, 1H), 2.14 (s, 6H); ^{13}C NMR (100 MHz, $CDCl_3$) δ (ppm) 135.90, 134.09, 133.91, 133.47, 129.12, 121.50, 120.90, 10.92, 6.83. HRMS calculated for $C_{10}H_{11}O_2S^+$ $[(M+H)^+]$ = 195.0476, found: 195.0474.

3. General procedure for asymmetric hydrogenation

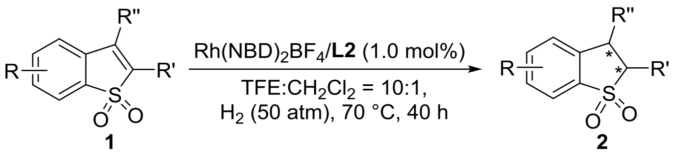

A stock solution was made by mixing $Rh(NBD)_2BF_4$ with *N*-methylated ZhaoPhos **L2** in a 1:1.1 molar ratio in CH_2Cl_2 at room temperature for 40 min in a argon-filled glovebox. An aliquot of the catalyst solution (0.1 mL, 0.001 mmol) was transferred by syringe into the vials charged with different substrates (0.1 mmol for each) in anhydrous CF_3CH_2OH (1.0 mL). The vials were subsequently transferred into an autoclave into which hydrogen gas was then charged. The reaction was then stirred under H_2 (50 atm) at 70°C for 40 h. After completed, the hydrogen gas was released slowly and carefully. The solution was concentrated and passed through a short column of silica gel (eluant: EA) to remove the metal complex. The ee values of all compounds were determined by HPLC analysis on a chiral stationary phase.

The absolute configurations of products **2a**, **2n** and **2v** were determined by comparison of analytical data with the literatures (optical rotation data). [2] The absolute configuration of others was assigned by analogy.

(*R*)-2-phenyl-2,3-dihydrobenzo[*b*]thiophene 1,1-dioxide **2a**

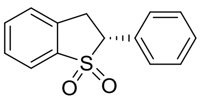

Colorless crystalline solid; >99% conv., 98% yield, 24.0 mg, >99% ee; $[\alpha]_D^{20}$ = -75.0 (c = 0.5, $CHCl_3$); The enantiomeric excess was determined by HPLC on Chiralpak AS-H column, hexane: isopropanol = 80:20; flow rate = 1.0 mL/min; UV detection at 220 nm; t_R = 54.6 min (major), 74.7 min (minor). ^1H NMR (400 MHz, $CDCl_3$) δ (ppm) 7.80 (d, *J* = 8.0 Hz, 1H), 7.64-7.60 (m, 1H), 7.53-7.50 (m, 1H), 7.49-7.42 (m, 6H), 4.68 (t, *J* = 8.4 Hz, 1H), 3.65 (d, *J* = 8.4 Hz, 2H); ^{13}C NMR (100 MHz, $CDCl_3$) δ (ppm) 138.30, 136.30, 133.42, 129.99, 129.39, 129.31, 129.05, 128.97, 127.01, 122.32, 67.33, 32.78. The characterization data of compound **2a** is in accordance with the reported data in the literature.[2]

(R)-2-(4-methoxyphenyl)-2,3-dihydrobenzo[*b*]thiophene 1,1-dioxide **2b**

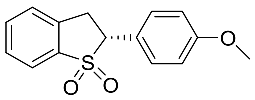

Colorless crystalline solid; >99% conv., 98% yield, 26.8 mg, 97% ee; $[\alpha]_D^{20}$ = -57.8 (c = 0.6, $CHCl_3$); The enantiomeric excess was determined by HPLC on Chiralpak AD-H column, hexane: isopropanol = 80:20; flow rate = 1.0 mL/min; UV detection at 220 nm; t_R = 36.6 min (minor), 54.5 min (major). ^1H NMR (400 MHz, $CDCl_3$) δ (ppm) 7.79 (d, J = 7.7 Hz, 1H), 7.63-7.59 (m, 1H), 7.50 (t, J = 7.4 Hz, 1H), 7.44 (d, J = 7.6 Hz, 1H), 7.41-7.38 (m, 2H), 6.98-6.94 (m, 2H), 4.63 (t, J = 8.5 Hz, 1H), 3.83 (s, 3H), 3.61-3.59 (m, 2H); ^{13}C NMR (100 MHz, $CDCl_3$) δ (ppm) 160.39, 138.25, 136.36, 133.34, 130.50, 128.99, 126.97, 122.33, 121.57, 114.40, 66.82, 55.30, 32.82. The characterization data of compound **2b** is in accordance with the reported data in the literature.[2]

(R)-2-(4-fluorophenyl)-2,3-dihydrobenzo[*b*]thiophene 1,1-dioxide **2c**

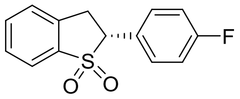

Colorless crystalline solid; >99% conv., 99% yield, 25.9 mg, 97% ee; $[\alpha]_D^{20}$ = -57.9 (c = 0.62, $CHCl_3$); The enantiomeric excess was determined by HPLC on Chiralpak AD-H column, hexane: isopropanol = 90:10; flow rate = 1.0 mL/min; UV detection at 220 nm; t_R = 47.9 min (minor), 55.2 min (major). ^1H NMR (400 MHz, $CDCl_3$) δ (ppm) 7.80 (d, J = 7.7 Hz, 1H), 7.65-7.61 (m, 1H), 7.52 (t, J = 7.6 Hz, 1H), 7.48-7.44 (m, 3H), 7.16-7.11 (m, 2H), 4.68-4.64 (m, 1H), 3.69-3.55 (m, 2H); ^{13}C NMR (100 MHz, $CDCl_3$) δ (ppm) 163.35 (d, J = 248.0 Hz), 138.10, 136.06, 133.51, 131.12 (d, J = 8.0 Hz), 129.16, 127.01, 125.76 (d, J = 3.0 Hz), 122.36, 116.07 (d, J = 21.0 Hz), 66.54, 32.97. The characterization data of compound **2c** is in accordance with the reported data in the literature.[2]

(R)-2-(o-tolyl)-2,3-dihydrobenzo[*b*]thiophene 1,1-dioxide **2d**

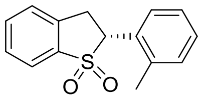

Colorless crystalline solid; >99% conv., 98% yield, 25.3 mg, 97% ee; $[\alpha]_D^{20}$ = -191.8 (c = 0.44, $CHCl_3$); The enantiomeric excess was determined by HPLC on Chiralpak AD-H column, hexane: isopropanol = 90:10; flow rate = 1.0 mL/min; UV detection at 220 nm; t_R = 30.0 min (minor), 35.0 min (major). ^{1}H NMR (400 MHz, $CDCl_3$) δ (ppm) 7.78 (d, *J* = 7.7 Hz, 1H), 7.64-7.60 (m, 1H), 7.52 (t, *J* = 7.6 Hz, 1H), 7.46 (d, *J* = 7.7 Hz, 1H), 7.35 (d, *J* = 7.3 Hz, 1H), 7.30-7.22 (m, 3H), 5.06 (t, *J* = 7.9 Hz, 1H), 3.65 (d, *J* = 7.5 Hz, 2H), 2.55 (s, 3H); ^{13}C NMR (100 MHz, $CDCl_3$) δ (ppm) 138.54, 138.32, 136.69, 133.40, 130.92, 129.04, 129.00, 128.85, 127.84, 126.91, 126.47, 122.29, 62.93, 33.10, 20.24. The characterization data of compound **2d** is in accordance with the reported data in the literature.[2]

(*R*)-2-(*m*-tolyl)-2,3-dihydrobenzo[*b*]thiophene 1,1-dioxide **2e**

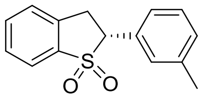

Colorless crystalline solid; >99% conv., 98% yield, 25.3 mg, 97% ee; $[\alpha]_D^{20}$ = -71.7 (c = 0.67, $CHCl_3$); The enantiomeric excess was determined by HPLC on Chiralpak AD-H column, hexane: isopropanol = 90:10; flow rate = 1.0 mL/min; UV detection at 220 nm; t_R = 40.4 min (major), 56.9 min (minor). ^{1}H NMR (400 MHz, $CDCl_3$) δ (ppm) 7.80 (d, *J* = 7.7 Hz, 1H), 7.63-7.59 (m, 1H), 7.51 (t, *J* = 7.3 Hz, 1H), 7.44 (d, *J* = 7.6 Hz, 1H), 7.33 (t, *J* = 7.5 Hz , 1H), 7.28-7.23 (m, 3H), 4.65 (t, *J* = 8.5 Hz, 1H), 3.64 (d, *J* = 8.1 Hz, 2H), 2.39 (s, 3H); ^{13}C NMR (100 MHz, $CDCl_3$) δ (ppm) 138.67, 138.31, 136.33, 133.37, 130.19, 130.04, 129.75, 128.99, 128.82, 126.99, 126.24, 122.28, 67.29, 32.65, 21.41. The characterization data of compound **2e** is in accordance with the reported data in the literature.[2]

(*R*)-2-(4-(tert-butyl)phenyl)-2,3-dihydrobenzo[*b*]thiophene 1,1-dioxide **2f**

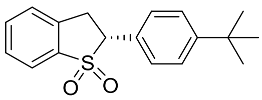

Colorless crystalline solid; >99% conv., 98% yield, 29.4 mg, 96% ee; $[\alpha]_D^{20}$ = -26.7 (c = 0.80, $CHCl_3$); The enantiomeric excess was determined by HPLC on Chiralpak AD-H column, hexane: isopropanol = 90:10; flow rate = 1.0 mL/min; UV detection at 220 nm; t_R = 29.1 min (minor), 31.1 min (major). ^1H NMR (400 MHz, $CDCl_3$) δ (ppm) 7.79 (d, J = 7.6 Hz, 1H), 7.62-7.58 (m, 1H), 7.50 (t, J = 7.3 Hz, 1H), 7.47-7.39 (m, 5H), 4.66 (t, J = 8.5 Hz, 1H), 3.62 (d, J = 8.4 Hz, 2H), 1.33 (m, 9H); ^{13}C NMR (100 MHz, $CDCl_3$) δ (ppm) 152.42, 138.35, 136.42, 133.34, 129.01, 128.98, 127.00, 126.74, 125.96, 122.30, 67.09, 34.66, 32.78, 31.20. HRMS calculated for $C_{18}H_{20}NaO_2S^+$ $[(M+Na)^+]$ = 323.1078, found: 323.1076.

(*R*)-2-(3-chlorophenyl)-2,3-dihydrobenzo[*b*]thiophene 1,1-dioxide **2g**

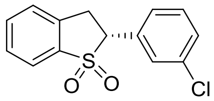

Colorless crystalline solid; >99% conv., 98% yield, 27.2 mg, 98% ee; $[\alpha]_D^{20}$ = -58.8 (c = 0.53, $CHCl_3$); The enantiomeric excess was determined by HPLC on Chiralpak AD-H column, hexane: isopropanol = 90:10; flow rate = 1.0 mL/min; UV detection at 220 nm; t_R = 42.3 min (major), 48.3 min (minor). ^1H NMR (400 MHz, $CDCl_3$) δ (ppm) 7.80 (d, J = 7.7 Hz, 1H), 7.65-7.61 (m, 1H), 7.53 (t, J = 7.5 Hz, 1H), 7.46-7.44 (m, 2H), 7.42-7.36 (m, 3H), 4.64 (t, J = 8.0 Hz, 1H), 3.69-3.56 (m, 2H); ^{13}C NMR (100 MHz, $CDCl_3$) δ (ppm) 138.04, 135.89, 134.83, 133.60, 132.03, 130.17, 129.59, 129.39, 129.21, 127.56, 127.04, 122.32, 66.67, 32.70. HRMS calculated for $C_{14}H_{11}ClNaO_2S^+$ $[(M+Na)^+]$ = 301.0059, found: 301.0060.

(*R*)-2-(naphthalen-2-yl)-2,3-dihydrobenzo[*b*]thiophene 1,1-dioxide **2h**

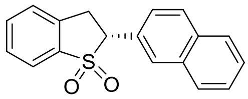

Colorless crystalline solid; >99% conv., 98% yield, 28.8 mg, 95% ee; $[\alpha]_D^{20}$ = -79.1 (c = 0.67, $CHCl_3$); The enantiomeric excess was determined by HPLC on Chiralcel OD-H column, hexane: isopropanol = 85:15; flow rate = 1.0 mL/min; UV detection at 220 nm; t_R = 35.4 min (major), 52.5 min (minor). ^1H NMR (400 MHz,

$CDCl_3$) δ (ppm) 7.94-7.81 (m, 5H), 7.64 (t, *J* = 7.1 Hz, 1H), 7.58-7.47 (m, 5H), 4.85 (t, *J* = 8.4 Hz, 1H), 3.82-3.69 (m, 2H); ^{13}C NMR (100 MHz, $CDCl_3$) δ (ppm) 138.33, 136.29, 133.57, 133.47, 133.13, 129.23, 129.08, 128.78, 128.08, 127.71, 127.41, 127.04, 126.79, 126.54, 126.03, 122.33, 67.45, 32.78. HRMS calculated for $C_{18}H_{14}NaO_2S^+$ [$(M+Na)^+$] = 317.0610, found: 317.0606.

(+)-2-benzyl-2,3-dihydrobenzo[*b*]thiophene 1,1-dioxide **2i**

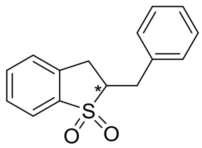

Colorless crystalline solid; >99% conv., 98% yield, 25.3 mg, 83% ee; $[\alpha]_D^{20}$ = +27.2 (c = 0.66, $CHCl_3$); The enantiomeric excess was determined by HPLC on Chiralpak AS-H column, hexane: isopropanol = 80:20; flow rate = 1.0 mL/min; UV detection at 220 nm; t_R = 53.8 min (major), 68.0 min (minor). ^{1}H NMR (400 MHz, $CDCl_3$) δ (ppm) 7.77 (d, *J* = 7.7 Hz, 1H), 7.57-7.53 (m, 1H), 7.47 (t, *J* = 7.4 Hz, 1H), 7.38-7.35 (m, 2H), 7.31-7.27 (m, 4H), 3.77-3.69 (m, 1H), 3.54 (dd, *J* = 14.0, 4.7 Hz 1H), 3.22 (dd, *J* = 16.3, 7.5 Hz 1H), 3.05 (dd, *J* = 16.3, 8.1 Hz, 1H), 2.90 (dd, *J* = 14.0, 10.7 Hz, 1H); ^{13}C NMR (100 MHz, $CDCl_3$) δ (ppm) 138.85, 136.53, 136.31, 133.41, 128.98, 128.93, 128.79, 127.17, 127.10, 121.93, 62.37, 33.15, 31.83. The characterization data of compound **2i** is in accordance with the reported data in the literature.[2]

(+)-2-ethyl-2,3-dihydrobenzo[*b*]thiophene 1,1-dioxide **2j**

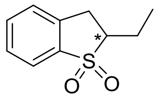

White solid; >99% conv., 99% yield, 19.4 mg, 88% ee; $[\alpha]_D^{20}$ = +20.0 (c =0.58, $CHCl_3$); The enantiomeric excess was determined by HPLC on Chiralpak AS-H column, hexane: isopropanol = 80:20; flow rate = 1.0 mL/min; UV detection at 220 nm; t_R = 28.9 min (major), 65.0 min (minor). ^{1}H NMR (400 MHz, $CDCl_3$) δ (ppm) 7.74 (d, *J* = 7.7 Hz, 1H), 7.55 (td, *J* = 7.5, 1.0 Hz, 1H), 7.45 (t, *J* = 7.5 Hz, 1H), 7.34

(d, J = 7.7 Hz, 1H), 3.45-3.35 (m, 2H), 3.00-2.93 (m, 1H), 2.23-2.12 (m, 1H), 1.85-1.74 (m, 1H), 1.19 (t, J = 7.5 Hz, 3H); ^{13}C NMR (100 MHz, $CDCl_3$) δ (ppm) 139.17, 136.41, 133.26, 128.69, 126.98, 121.79, 62.94, 32.19, 21.08, 11.60. The characterization data of compound **2j** is in accordance with the reported data in the literature.[2]

(+)-2-propyl-2,3-dihydrobenzo[*b*]thiophene 1,1-dioxide **2k**

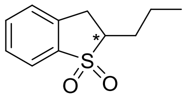

White solid; >99% conv., 98% yield, 20.6 mg, 92% ee; $[\alpha]_D^{20}$ = +19.2 (c =0.5, $CHCl_3$); The enantiomeric excess was determined by HPLC on Chiralpak AS-H column, hexane: isopropanol = 80:20; flow rate = 1.0 mL/min; UV detection at 220 nm; t_R = 27.2 min (major), 50.6 min (minor). ^{1}H NMR (400 MHz, $CDCl_3$) δ 7.76 (d, J = 7.6 Hz, 1H), 7.58-7.54 (m, 1H), 7.46 (t, J = 7.5 Hz, 1H), 7.34 (d, J = 7.7 Hz, 1H), 3.51-3.38 (m, 2H), 3.02–2.96 (m, 1H), 2.17-2.08 (m, 1H), 1.78–1.71 (m, 1H), 1.64-1.60 (m, 2H), 1.04 (t, J = 7.3 Hz, 3H); ^{13}C NMR (100 MHz, $CDCl_3$) δ (ppm) 139.10, 136.46, 133.22, 128.64, 126.97, 121.74, 61.24, 32.37, 29.48, 20.27, 13.88. The characterization data of compound **2k** is in accordance with the reported data in the literature.[2]

(+)-2-butyl-2,3-dihydrobenzo[*b*]thiophene 1,1-dioxide **2l**

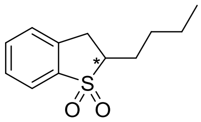

White solid; >99% conv., 98% yield, 22.0 mg, 91% ee; $[\alpha]_D^{20}$ = +20.8 (c =0.72, $CHCl_3$); The enantiomeric excess was determined by HPLC on Chiralpak AS-H column, hexane: isopropanol = 80:20; flow rate = 1.0 mL/min; UV detection at 220 nm; t_R = 30.2 min (major), 47.7 min (minor). ^{1}H NMR (400 MHz, $CDCl_3$) δ (ppm) 7.74 (d, J = 7.7 Hz, 1H), 7.57-7.52 (m, 1H), 7.45 (t, J = 7.5 Hz, 1H), 7.33 (d, J = 7.7 Hz, 1H), 3.46-3.38 (m, 2H), 3.02–2.94 (m, 1H), 2.16-2.10 (m, 1H), 1.76-1.71 (m, 1H), 1.61-1.51 (m, 2H), 1.45-1.39 (m, 2H), 0.95 (t, J = 7.3 Hz, 3H); ^{13}C NMR (100 MHz,

$CDCl_3$) δ (ppm) 139.14, 136.47, 133.23, 128.66, 126.97, 121.79, 61.57, 32.44, 29.07, 27.20, 22.54, 13.78. HRMS calculated for $C_{12}H_{16}NaO_2S^+$ [$(M+Na)^+$] = 247.0766, found: 247.0763.

(*R*)-3-phenyl-2,3-dihydrobenzo[*b*]thiophene 1,1-dioxide **2m**

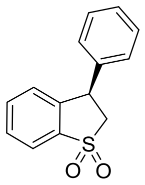

Colorless crystalline solid; >99% conv., 99% yield, 24.2 mg, >99% ee; $[\alpha]_D^{20}$ = +9.4 (c = 0.5, $CHCl_3$); The enantiomeric excess was determined by HPLC on Chiralpak AS-H column, hexane: isopropanol = 80:20; flow rate = 1.0 mL/min; UV detection at 220 nm; t_R = 58.9 min (major), t_R = 39.6 min (minor). ^{1}H NMR (400 MHz, $CDCl_3$) δ (ppm) 7.82-7.80 (m, 1H), 7.57-7.50 (m, 2H), 7.41-7.32 (m, 3H), 7.26-7.24 (m, 2H), 7.10 (d, *J* = 7.4 Hz, 1H), 4.79 (t, *J* = 7.8 Hz, 1H), 3.93 (dd, *J* = 13.5, 8.0 Hz, 1H), 3.51 (dd, *J* = 13.5, 7.8 Hz, 1H); ^{13}C NMR (100 MHz, $CDCl_3$) δ (ppm) 141.00, 139.70, 139.02, 133.71, 129.29, 129.11, 128.21, 128.10, 127.21, 121.15, 58.93, 43.96. The characterization data of compound **2m** is in accordance with the reported data in the literature.[2]

(*R*)-3-(4-methoxyphenyl)-2,3-dihydrobenzo[*b*]thiophene 1,1-dioxide **2n**

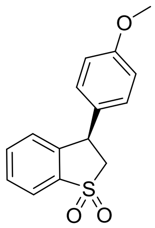

Colorless crystalline solid; >99% conv., 99% yield, 27.2 mg, >99% ee; $[\alpha]_D^{20}$ = +6.7 (c = 0.7, $CHCl_3$); The enantiomeric excess was determined by HPLC on Chiralpak AS-H column, hexane: isopropanol = 80:20; flow rate = 1.0 mL/min; UV detection at 220 nm; t_R = 82.7 min (major), t_R = 55.4 min (minor). ^{1}H NMR (400 MHz, $CDCl_3$) δ (ppm) 7.81-7.78 (m, 1H), 7.56-7.48 (m, 2H), 7.17-7.14 (m, 2H), 7.11-7.10

(m, 1H), 6.92-6.88 (m, 2H), 4.75 (t, J = 7.9 Hz, 1H), 3.89 (dd, J = 13.5, 7.9 Hz, 1H), 3.82 (s, 3H), 3.46 (dd, J = 13.5, 7.9 Hz, 1H); ^{13}C NMR (100 MHz, $CDCl_3$) δ (ppm) 159.28, 141.39, 138.97, 133.66, 131.59, 129.28, 129.01, 127.14, 121.08, 114.58, 59.06, 55.31, 43.22. The characterization data of compound **2n** is in accordance with the reported data in the literature.[2]

(*R*)-3-(*m*-tolyl)-2,3-dihydrobenzo[*b*]thiophene 1,1-dioxide **2o**

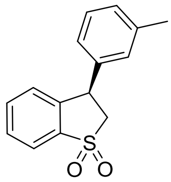

Colorless crystalline solid; >99% conv., 99% yield, 25.6 mg, >99% ee; $[\alpha]_D^{20}$ = +6.26 (c = 0.33 $CHCl_3$); The enantiomeric excess was determined by HPLC on Chiralpak AS-H column, hexane: isopropanol = 80:20; flow rate = 1.0 mL/min; UV detection at 220 nm; t_R = 41.4 min (major), t_R = 28.0 min (minor). ^{1}H NMR (400 MHz, $CDCl_3$) δ (ppm) 7.81-7.79 (m, 1H), 7.56-7.49 (m, 2H), 7.28-7.25 (m, 1H), 7.15 (d, J = 7.5 Hz, 1H), 7.10 (d, J = 7.5 Hz, 1H), 7.04-7.02 (m, 2H), 4.75 (t, J = 7.9 Hz, 1H), 3.91 (dd, J =13.5, 8.0 Hz, 1H), 3.50 (dd, J = 13.5, 7.9 Hz, 1H), 2.34 (s, 3H); ^{13}C NMR (100 MHz, $CDCl_3$) δ (ppm) 141.13, 139.61, 139.11, 138.99, 133.69, 129.14, 129.04, 128.84, 127.23, 125.27, 121.10, 58.94, 43.90, 21.38. The characterization data of compound **2o** is in accordance with the reported data in the literature.[2]

(*R*)-3-(*p*-tolyl)-2,3-dihydrobenzo[*b*]thiophene 1,1-dioxide **2p**

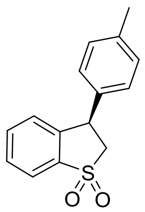

Colorless crystalline solid; >99% conv., 99% yield, 25.6 mg, >99% ee; $[\alpha]_D^{20}$ = +8.5 (c = 0.75, $CHCl_3$); The enantiomeric excess was determined by HPLC on Chiralpak AS-H column, hexane: isopropanol = 80:20; flow rate = 1.0 mL/min; UV

detection at 220 nm; t_R = 40.3 min (major), t_R = 33.9 min (minor). ^{1}H NMR (400 MHz, $CDCl_3$) δ (ppm) 7.80-7.78 (m, 1H), 7.56-7.48 (m, 2H), 7.19 (d, *J* = 7.9 Hz, 2H), 7.13-7.09 (m, 3H), 4.75 (t, *J* = 7.9 Hz, 1H), 3.90 (dd, *J* =13.5, 7.9 Hz, 1H), 3.48 (dd, *J* = 13.5, 7.9 Hz, 1H), 2.36 (s, 3H); ^{13}C NMR (100 MHz, $CDCl_3$) δ (ppm) 141.27, 139.01, 137.93, 136.64, 133.66, 129.93, 129.02, 128.07, 127.17, 121.11, 59.02, 43.60, 21.07. The characterization data of compound **2p** is in accordance with the reported data in the literature.[2]

(-)-6-methoxy-3-phenyl-2,3-dihydrobenzo[*b*]thiophene 1,1-dioxide **2q**

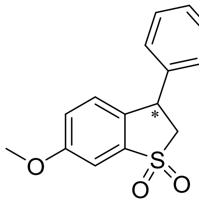

Colorless crystalline solid; >99% conv., 99% yield, 27.1 mg, >99% ee; $[\alpha]_D{}^{20}$ = -10.56 (c =3.00, CH_3OH); The enantiomeric excess was determined by HPLC on Chiralpak AS-H column, hexane: isopropanol = 80:20; flow rate = 1.0 mL/min; UV detection at 220 nm; t_R = 36.8 min (minor), t_R = 39.5 min (major). ^{1}H NMR (400 MHz, $CDCl_3$) δ (ppm) 7.39-7.30 (m, 3H), 7.24-7.22 (m, 3H), 7.08 (dd, *J* = 8.6, 2.5 Hz, 1H), 6.98 (d, *J* = 8.6 Hz, 1H), 4.72 (t, *J* = 7.7 Hz, 1H), 3.93 (dd, *J* = 13.5, 8.0 Hz, 1H), 3.87 (s, 3H), 3.50 (dd, *J* = 13.5, 7.4 Hz, 1H); ^{13}C NMR (100 MHz, $CDCl_3$) δ (ppm) 160.31, 140.11, 139.98, 132.74, 129.23, 128.13, 128.07, 128.00, 122.24, 103.37, 59.60, 55.83, 43.22. HRMS calculated for $C_{15}H_{14}NaO_3S^+$ [$(M+Na)^+$] = 297.0558, found: 297.0555.

(*R*)-3-(4-fluorophenyl)-2,3-dihydrobenzo[*b*]thiophene 1,1-dioxide **2r**

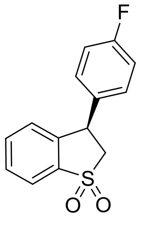

Colorless crystalline solid; >99% conv., 99% yield, 26.0 mg, >99% ee; $[\alpha]_D{}^{20}$ =

+7.7 (c = 0.7, $CHCl_3$); The enantiomeric excess was determined by HPLC on Chiralpak AS-H column, hexane: isopropanol = 80:20; flow rate = 1.0 mL/min; UV detection at 220 nm; t_R = 43.8 min (minor), t_R = 63.9 min (major). ^{1}H NMR (400 MHz, $CDCl_3$) δ (ppm) 7.82-7.80 (m, 1H), 7.59-7.51 (m, 2H), 7.24-7.20 (m, 2H), 7.11-7.05 (m, 3H), 4.79 (t, *J* = 7.7 Hz, 1H), 3.92 (dd, *J* = 13.5, 8.0 Hz, 1H), 3.46 (dd, *J* =13.5, 7.5 Hz, 1H); ^{13}C NMR (100 MHz, $CDCl_3$) δ (ppm) 162.35 (d, *J* = 246.0 Hz), 140.71, 139.03, 135.60 (d, *J* = 3.0 Hz), 133.81, 129.84 (d, *J* = 8.0 Hz), 129.27, 127.11, 121.24, 116.25 (d, *J* = 21.0 Hz), 58.88, 43.23. The characterization data of compound **2r** is in accordance with the reported data in the literature.[2]

(*R*)-3-(3-chlorophenyl)-2,3-dihydrobenzo[*b*]thiophene 1,1-dioxide **2s**

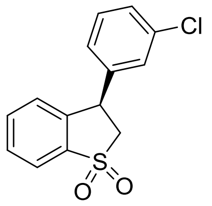

Colorless crystalline solid; >99% conv., 99% yield, 27.5 mg, >99% ee; $[\alpha]_D^{20}$ = +2.03 (c = 0.59, $CHCl_3$); The enantiomeric excess was determined by HPLC on Chiralpak AS-H column, hexane: isopropanol = 80:20; flow rate = 1.0 mL/min; UV detection at 220 nm; t_R = 35.5 min (minor), t_R = 61.5 min (major); ^{1}H NMR (400 MHz, $CDCl_3$) δ (ppm) 7.83-7.80 (m, 1H), 7.60-7.52 (m, 2H), 7.34-7.30 (m, 2H), 7.25 (d, *J* = 0.8 Hz, 1H), 7.15-7.11 (m, 2H), 4.77 (t, *J* = 7.8 Hz, 1H), 3.92 (dd, *J* = 13.5, 8.1 Hz, 1H), 3.48 (dd, *J* = 13.5, 7.5 Hz, 1H); ^{13}C NMR (100 MHz, $CDCl_3$) δ (ppm) 141.81, 140.08, 139.09, 135.13, 133.90, 130.63, 129.43, 128.40, 128.37, 127.15, 126.39, 121.34, 58.62, 43.59. HRMS calculated for $C_{14}H_{11}ClNaO_2S^+$ $[(M+Na)^+]$ = 301.0063, found: 301.0060.

(*R*)-3-(4-chlorophenyl)-2,3-dihydrobenzo[*b*]thiophene 1,1-dioxide **2t**

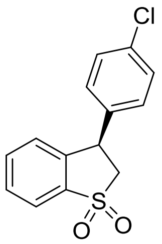

Colorless crystalline solid; >99% conv., 98% yield, 27.2 mg, 94% ee; $[\alpha]_D^{20}$ = +1.60 (c = 0.56, $CHCl_3$); The enantiomeric excess was determined by HPLC on Chiralpak AS-H column, hexane: isopropanol = 80:20; flow rate = 1.0 mL/min; UV detection at 220 nm; t_R = 54.8 min (minor), t_R = 59.5 min (major). ^1H NMR (400 MHz, $CDCl_3$) δ (ppm) 7.81-7.79 (m, 1H), 7.58-7.50 (m, 2H), 7.37-7.33 (m, 2H), 7.20-7.17 (m, 2H), 7.10-7.08 (m, 1H), 4.77 (t, J = 7.7 Hz, 1H), 3.91 (dd, J = 13.5, 8.1 Hz, 1H), 3.45 (dd, J = 13.5, 7.4 Hz, 1H); ^{13}C NMR (100 MHz, $CDCl_3$) δ (ppm) 140.37, 139.04, 138.33, 134.06, 133.84, 129.54, 129.48, 129.34, 127.10, 121.28, 58.69, 43.33. HRMS calculated for $C_{14}H_{11}ClNaO_2S^+$ [$(M+Na)^+$] = 301.0062, found: 301.0060.

(-)-3-(naphthalen-2-yl)-2,3-dihydrobenzo[*b*]thiophene 1,1-dioxide **2u**

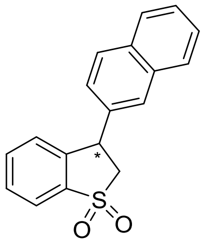

Colorless crystalline solid; >99% conv., 99% yield, 29.1 mg, >99% ee; $[\alpha]_D^{20}$ = -49.64 (c = 0.56, $CHCl_3$); The enantiomeric excess was determined by HPLC on Chiralpak AS-H column, hexane: isopropanol = 80:20; flow rate = 1.0 mL/min; UV detection at 220 nm; t_R = 37.5 min (minor), t_R = 56.2 min (major); ^1H NMR (400 MHz, $CDCl_3$) δ (ppm) 7.87-7.82 (m, 4H), 7.77 (d, J = 1.3 Hz, 1H), 7.55-7.51 (m, 4H), 7.27-7.25 (m, 1H), 7.12-7.10 (m, 1H), 4.96 (t, J = 7.9 Hz, 1H), 3.98 (dd, J = 13.6, 8.0 Hz, 1H), 3.61 (dd, J = 13.6, 7.8 Hz, 1H); ^{13}C NMR (100 MHz, $CDCl_3$) δ (ppm) 140.89, 139.12, 136.85, 133.75, 133.31, 132.83, 129.48, 129.19, 127.75, 127.73, 127.70, 127.31, 126.75, 126.51, 125.16, 121.21, 58.75, 44.14. HRMS calculated for $C_{18}H_{14}NaO_2S^+$ [$(M+Na)^+$] = 317.0609, found: 317.0606.

(*R*)-3-methyl-2,3-dihydrobenzo[*b*]thiophene 1,1-dioxide **2v**

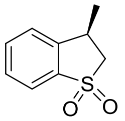

Colorless crystalline solid; >99% conv., 97% yield, 17.7 mg, 97% ee; $[\alpha]_D^{20}$ = +30.7 (c = 0.41, $CHCl_3$); The enantiomeric excess was determined by HPLC on Chiralpak AD-H column, hexane: isopropanol = 95:5; flow rate = 1.0 mL/min; UV detection at 220 nm; t_R = 26.1 min (major), 27.7 min (minor). ^{1}H NMR (400 MHz, $CDCl_3$) δ (ppm) 7.73 (d, *J*= 7.8 Hz, 1H), 7.64-7.60 (m, 1H), 7.49 (t, *J* = 7.5 Hz, 1H), 7.44 (d, *J* = 7.8 Hz, 1H), 3.74-3.65 (m, 2H), 3.17-3.10 (m, 1H), 1.54 (d, *J* = 6.8 Hz, 3H); ^{13}C NMR (100 MHz, $CDCl_3$) δ (ppm) 142.34, 138.63, 133.64, 128.79, 125.52, 121.28, 57.90, 32.57, 19.83. The characterization data of compound **2v** is in accordance with the reported data in the literature.[2]

(*R*)-3-ethyl-2,3-dihydrobenzo[*b*]thiophene 1,1-dioxide **2w**

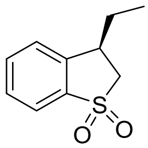

Colorless crystalline solid; >99% conv., 98% yield, 19.0 mg, 98% ee; $[\alpha]_D^{20}$ = +81.73 (c = 0.46, $CHCl_3$); The enantiomeric excess was determined by HPLC on Chiralpak AD-H column, hexane: isopropanol = 95:5; flow rate = 0.3 mL/min; UV detection at 220 nm; t_R = 83.0 min (major), t_R = 87.2 min (minor); ^{1}H NMR (400 MHz, $CDCl_3$) δ (ppm) 7.72 (d, *J* = 7.8 Hz, 1H), 7.62-7.58 (m, 1H), 7.48 (t, *J* = 7.5 Hz, 1H), 7.43-7.41 (m, 1H), 3.62 (dd, *J* = 13.0, 7.8 Hz, 1H), 3.56-3.50 (m, 1H), 3.20 (dd, *J* = 13.0, 5.3 Hz, 1H), 2.08-2.05 (m, 1H), 1.76-1.74 (m, 1H), 1.04 (t, *J*= 7.4 Hz, 3H); ^{13}C NMR (100 MHz, $CDCl_3$) δ (ppm) 141.17, 138.99, 133.45, 128.89, 125.99, 121.41, 55.47, 39.40, 27.32, 11.55. HRMS calculated for $C_{10}H_{12}NaO_2S^+$ $[(M+Na)^+]$ = 219.0450, found: 219.0450.

(*R*)-3-propyl-2,3-dihydrobenzo[*b*]thiophene 1,1-dioxide **2x**

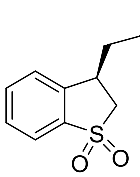

Colorless crystalline solid; >99% conv., 98% yield, 20.6 mg, 96% ee; $[\alpha]_D^{20}$ = +36.87 (c = 1.33, $CHCl_3$); The enantiomeric excess was determined by HPLC on Chiralpak AD-H column, hexane: isopropanol = 95:5; flow rate = 0.3 mL/min; UV detection at 220 nm; t_R = 69.1 min (major), t_R = 73.2 min (minor). ^{1}H NMR (400 MHz, $CDCl_3$) δ (ppm) 7.72 (d, J = 7.8 Hz, 1H), 7.62-7.58 (m, 1H), 7.47 (t, J = 7.5 Hz, 1H), 7.42 (d, J = 7.8 Hz, 1H), 3.64-3.56 (m, 2H), 3.22-3.17 (m, 1H), 2.03-1.95 (m, 1H), 1.71-1.63 (m, 1H), 1.50-1.41 (m, 2H), 1.00 (t, J =7.3 Hz, 3H); ^{13}C NMR (100 MHz, $CDCl_3$) δ (ppm) 141.48, 138.85, 133.45, 128.86, 125.98, 121.42, 55.88, 37.80, 36.67, 20.57, 13.85. HRMS calculated for $C_{11}H_{14}NaO_2S^+$ [(M+Na)$^+$] = 233.0609, found: 233.0606.

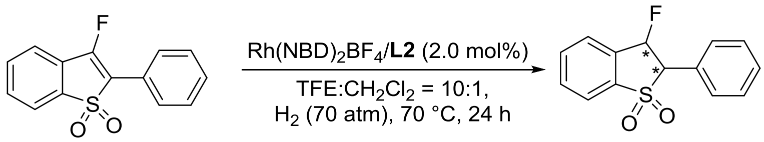

Colorless crystalline solid; 83% conv., >25:1 dr, 98% ee; $[\alpha]_D^{20}$ = +56.00 (c = 0.35, $CHCl_3$); The dr value was analyzed by ^{1}H NMR spectroscopy; The enantiomeric excess was determined by HPLC on Chiralcel OD-H column, hexane: isopropanol = 90:10; flow rate = 1.0 mL/min; UV detection at 220 nm; t_R = 35.0 min (major), t_R = 71.3 min (minor). ^{1}H NMR (400 MHz, $CDCl_3$) δ (ppm) 7.93 (d, J = 7.5 Hz, 1H), 7.79-7.73 (m, 3H), 7.58-7.55 (m, 2H), 7.50-7.43 (m, 3H), 6.11 (dd, J = 53.3, 5.1Hz, 1H), 4.70 (dd, J = 24.0, 5.1Hz, 1H); ^{13}C NMR (100 MHz, $CDCl_3$) δ (ppm) 139.80 (d, J = 3.0 Hz), 134.00 (d, J = 3.0 Hz), 133.60 (d, J = 18.0 Hz), 132.43 (d, J = 3.0 Hz), 131.78 (d, J = 3.0 Hz), 129.75, 128.72, 128.15, 126.32, 122.45, 87.11 (d, J = 190.0 Hz), 69.87 (d, J = 20.0 Hz). HRMS calculated for $C_{14}H_{11}FNaO_2S^+$ [(M+Na)$^+$] = 285.0356, found: 285.0355.

4. General procedure for the high TON experiment

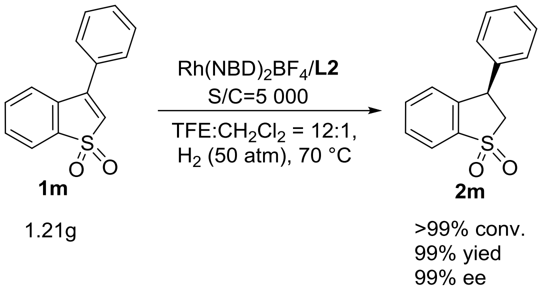

A stock solution was made by mixing [Rh(NBD)$_2$]BF$_4$ with *N*-methylated ZhaoPhos **L2** in a 1:1.1 molar ratio in CH_2Cl_2 at room temperature for 40 min in a nitrogen-filled glovebox. An aliquot of the catalyst solution (0.001 mmol) was transferred by syringe into a hydrogenated vial charged with **1m** (5.0 mmol, 1.21 g) in anhydrous CF_3CH_2OH :CH_2Cl_2 = 12:1 (8 mL). The cup was subsequently transferred into an autoclave into which hydrogen gas was charged. The reaction was then stirred under H_2 (50 atm) at 70 ^{o}C for 120 h. After completed, the hydrogen gas was released slowly and carefully. The product **2m** was obtained by a column of silica gel (petroleum ether–EtOAc).

5. General procedure for Job plot

In order to inspect the possible catalytic model, the experiments for the investigation of the nonlinear effect and ^{1}H NMR titration were conducted. The asymmetric hydrogenation of substrate **1m** was performed in the presence of ligand **L2** with different ee values (Table S1). As shown in Figure S1, no nonlinear effect was observed in this Rh-catalyzed asymmetric hydrogenation, which displayed that it should be no catalyst self-aggregation or ligand-substrate agglomeration in this catalytic system.

Table S1. Nonlinear effect for Rh/ligand **L2**-catalyzed asymmetric hydrogenation of **1m**.

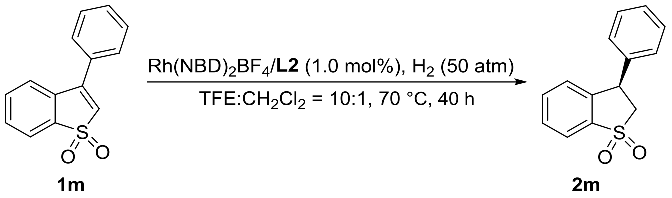

entry	ee of **L2** (%)	conv. (%)	ee (%)
1	0	>99	0
2	10	>99	11
3	20	>99	20
4	40	>99	40
5	60	>99	60
6	80	>99	80
7	90	>99	91
8	100	>99	>99

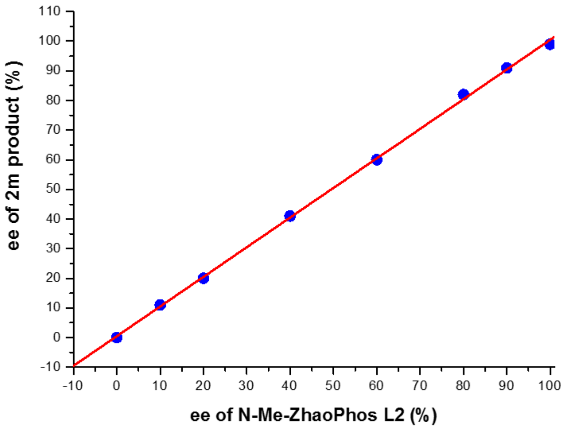

Figure S1. Nonlinear effect of the hydrogenation of substrate **1m** using ligand **L2** with different ee values

A series of ^{1}H NMR titration experiments were conducted in chloroform-d, which was dried with potassium carbonate and degassed. The total concentration of the host (**1m**) and the guest (ligand **L2**) was 0.02 M. The proportion of the concentration of the host *vs* the total concentration varied, the chemical shifts of the proton on the 2-position of **1m** were recorded (Table S2). A Job plot was drawn (Figure S2) and the curve suggests a 1:1 binding pattern between ligand **L2** and substrate **1m**.

Table S2. Data for Job plot.

NO.	c_1 (**1m**)	c_2 (**L2**)	δ (H-**1m**)	Δδ (H-**1m**)	$\Delta\delta*10^4*c_1/0.02$
1	0.02	0	6.6569	0	0
2	0.016	0.004	6.6556	0.0013	10.4
3	0.014	0.006	6.6551	0.0018	12.6
4	0.012	0.008	6.6547	0.0022	13.2
5	0.01	0.01	6.6542	0.0027	13.5
6	0.008	0.012	6.6538	0.0031	12.4
7	0.006	0.014	6.6532	0.0037	11.1
8	0.004	0.016	6.6527	0.0042	8.4

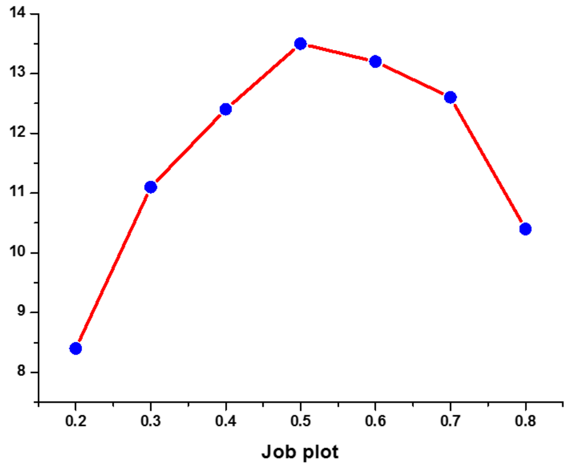

Figure S2. Job plot of ligand **L2** and **1m**, x = c (**1m**)/[c (ligand **L2**)+c (**1m**)], y = $\Delta\delta*10^4*c$ (**1m**)/[c (ligand **L2**)+c (**1m**)]

6. General computational calculation (DFT) details

DFT calculations were performed using the M06-L method [12] with the Gaussian09 program [13]. The 6-31G(d) basis set was used for the C, H, N, O, F, S and P, and the SDD basis set was used for the Rh and Fe. Solvent effect and Grimme's dispersion correction [14] were applied during geometry optimization. The parameter of int=ultrafine was used. Mixture of 2,2,2-trifluoroethanol and dichloromethane (10:1) was employed as the solvents. The solvent effects were considered using SMD continuum solvation model [15]. Frequency calculations at the same level of theory

have been performed to identify all of the stationary points as minima (zero imaginary frequencies) and to provide free energies at 298.15 K.

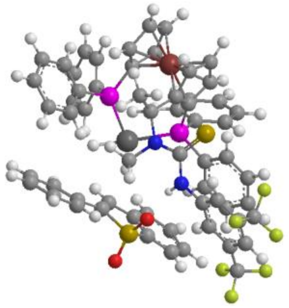

Rh(III)HH-**1a**-pro-(*S*) **I**:

Thermal correction to Gibbs Free Energy= 0.852493

Sum of electronic and thermal Free Energies= -4887.807460

Fe	2.42478900	-3.29284100	-0.46493600
S	-2.37544200	-3.34083100	1.17685000
N	-0.71879400	-1.31952700	1.79913300
N	-2.84536300	-0.67979800	1.31749800
F	-5.53705100	2.33804900	-1.45188100
F	-7.11988100	1.02373800	-2.14819800
F	-7.44382800	2.27296300	-0.41196400
F	-7.53634000	-2.55549300	2.91741500
F	-6.88382000	-3.89657400	1.35078400
F	-8.58921800	-2.59159300	1.01899300
C	0.49554100	-2.16166100	1.69788600
C	-1.96880800	-1.73402200	1.44467700
C	-4.20819800	-0.69686200	0.99626000
C	-6.01663600	0.41187800	-0.16062100
C	-6.44170900	-1.59913200	1.06322200
C	-6.52050300	1.51364800	-1.03853800
C	-7.36751300	-2.65649400	1.57946100
C	0.63905200	-2.91215700	0.39735600
C	0.84556500	-4.32081000	0.29792900

C	1.03949300	-4.66873900	-1.06493700
C	0.95086200	-3.48158800	-1.83797300
C	0.70964300	-2.37537300	-0.95188800
C	3.86182600	-1.92386400	-0.05943900
C	3.87529600	-2.99731700	0.90439300
C	4.10787400	-4.21708600	0.20999500
C	4.22294800	-3.92460000	-1.17938700
C	4.05692800	-2.52205500	-1.35910400
H	-2.41516600	0.22941900	1.15669800
H	1.27855200	-5.65653700	-1.44347000
H	1.10386200	-3.40545700	-2.90916100
H	3.73179300	-2.88663700	1.97430900
H	4.12963000	-5.20360100	0.65991200
H	4.35020100	-4.65010100	-1.97539800
H	4.08551600	-1.99608600	-2.30744600
C	0.66496000	-3.03749400	2.93133700
H	0.62358300	-2.42492700	3.83874600
H	-0.12802700	-3.78963600	2.98464200
H	1.63526000	-3.54645400	2.91432600
H	1.30941900	-1.41783000	1.72053400
C	-0.52604700	-0.07928200	2.53926400
H	-0.26182000	0.76795900	1.88518300
H	-1.43404600	0.19049800	3.08481800
H	0.27906100	-0.21997800	3.26849700
H	0.89349300	-5.00495400	1.13822000
P	0.61641600	-0.64642400	-1.49618600
P	3.73328200	-0.15979700	0.34140500
C	1.35413100	-0.69326200	-3.18369900
C	0.62698900	-1.21344700	-4.26466200
C	2.64680200	-0.20996400	-3.41988300

C	1.18611100	-1.26096200	-5.53827300
H	-0.38600500	-1.58429300	-4.11045700
C	3.20771100	-0.25775400	-4.69418600
H	3.23028300	0.20886400	-2.60070500
C	2.47773500	-0.78390600	-5.75680900
H	0.60702700	-1.66949300	-6.36375500
H	4.21494900	0.12347300	-4.85125500
H	2.91058800	-0.81687500	-6.75435100
C	5.16731900	0.58636400	-0.50949100
C	6.26758700	-0.20425100	-0.87307400
C	5.20855700	1.96636700	-0.75317900
C	7.38033000	0.37724400	-1.47479300
H	6.25920600	-1.27702200	-0.69087600
C	6.32392800	2.54110200	-1.35350000
H	4.36304900	2.59221000	-0.47231900
C	7.40974200	1.74775000	-1.71928700
H	8.22456500	-0.24845700	-1.75549700
H	6.34030500	3.61255000	-1.54015400
H	8.27772000	2.19784900	-2.19612000
C	-1.15345000	-0.44162700	-1.92756200
C	-1.58592000	0.78373900	-2.45360800
C	-2.04946600	-1.51595100	-1.87726000
C	-2.88283400	0.91907000	-2.94106500
H	-0.89458800	1.62841900	-2.50635800
C	-3.35259400	-1.36958900	-2.34754100
H	-1.73167300	-2.47376600	-1.47038900
C	-3.76707400	-0.15777200	-2.89282400
H	-3.20679000	1.86923200	-3.36096200
H	-4.04326800	-2.20969000	-2.28503300
H	-4.78366100	-0.04487100	-3.26565800

C	4.14329500	-0.16577100	2.12460400
C	3.26277800	0.33739800	3.08745500
C	5.34934400	-0.75145800	2.53742600
C	3.56349000	0.22073300	4.44362600
H	2.34815100	0.84912200	2.78556600
C	5.65681100	-0.84370000	3.88963000
H	6.04689200	-1.14504900	1.79810700
C	4.75709800	-0.37082000	4.84558000
H	2.86279400	0.60541200	5.18204100
H	6.59780400	-1.29381400	4.19779600
H	4.99309000	-0.45710400	5.90395200
Rh	1.81463300	0.89782600	-0.03977500
H	2.70665000	1.94566700	0.79395800
H	2.50620200	1.53102100	-1.24652500
C	-1.36644600	3.96053900	-0.96667000
C	0.00094100	4.02747500	-1.26446500
C	0.20926400	3.63843000	1.07740200
C	0.85375900	3.86545900	-0.09614900
S	-1.56893800	3.59952200	0.75950800
O	-2.24412600	4.67971700	1.48276600
O	-2.09541600	2.23140400	0.92856700
C	-6.91262900	-0.57412500	0.25298500
H	-7.95482200	-0.53976100	-0.05156300
C	-4.68154200	0.35988700	0.20729400
H	-3.98804600	1.12193200	-0.13993200
C	-5.10269000	-1.66630300	1.44874300
H	-4.76129900	-2.46991100	2.09137200
C	-2.35746400	4.12064100	-1.91398500
H	-3.41111000	4.06708500	-1.64841000
C	-1.95277500	4.35173500	-3.23396100

H	-2.70409700	4.48968400	-4.00768400
C	-0.59783100	4.39861000	-3.56267400
H	-0.30362600	4.56817100	-4.59552400
C	0.38852400	4.24051200	-2.58677900
H	1.44421000	4.28679900	-2.84754400
C	0.71816700	3.51264800	2.42902300
C	-0.10486900	3.07790800	3.48283300
C	2.06260500	3.82574200	2.70801800
C	0.40440200	2.94791900	4.77020600
H	-1.14788600	2.82750600	3.29571000
C	2.56337400	3.69848600	3.99482400
H	2.71135100	4.17878300	1.90835600
C	1.73685700	3.25915700	5.03132000
H	-0.24614700	2.60567200	5.57188700
H	3.60462900	3.94352500	4.19275300
H	2.13302200	3.16235000	6.04000400
H	1.93361300	3.97413600	-0.16895000

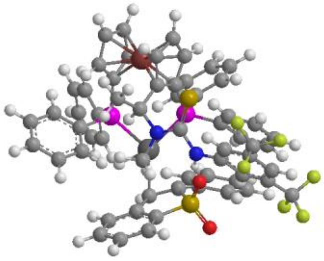

Rh(III)HH-**1a**-pro-(*R*) **II**:

Thermal correction to Gibbs Free Energy= 0.860058

Sum of electronic and thermal Free Energies= -4887.800670

Fe	2.07779300	-2.57936200	-2.16863700
S	-2.83940600	-3.14830900	-1.01451700
N	-1.13541300	-2.07720900	0.77621900
N	-3.19818000	-1.13528100	0.75469500
F	-5.68749000	3.08071500	0.28774400

F	-6.90541500	2.48062600	-1.40461200
F	-7.77036500	2.52611500	0.57910700
F	-8.43638700	-2.87969700	1.07338600
F	-7.30997300	-3.77804300	-0.54437300
F	-8.82764600	-2.28138000	-0.97161900
C	0.06670200	-2.71555400	0.19022600
C	-2.37741400	-2.10292800	0.21495100
C	-4.54295000	-0.87847000	0.46690100
C	-6.24880700	0.78964100	0.09237700
C	-6.81460200	-1.53673700	0.01200300
C	-6.64625700	2.21942000	-0.10075700
C	-7.84304900	-2.61790500	-0.11404900
C	0.27130400	-2.47971400	-1.28663600
C	0.28305500	-3.52856000	-2.25715900
C	0.60495200	-2.99071200	-3.52862500
C	0.79698700	-1.59371500	-3.37674300
C	0.60831000	-1.24699700	-1.99148800
C	3.57886400	-2.02752700	-0.95149900
C	3.36396200	-3.45047700	-0.87863700
C	3.54552000	-3.99680900	-2.17904100
C	3.87133800	-2.93287800	-3.06801900
C	3.88696000	-1.71711100	-2.32750300
H	-2.69937000	-0.31938400	1.11045300
H	0.74588900	-3.55396400	-4.44454800
H	1.10744200	-0.90951700	-4.15856600
H	3.10121700	-4.00298000	0.01660900
H	3.40501000	-5.03750800	-2.44975100
H	4.02324300	-3.01890100	-4.13835300
H	4.09452100	-0.73193000	-2.73100500
C	0.12667000	-4.18785100	0.58030500

H	0.09754500	-4.29558600	1.67005500
H	-0.72616200	-4.72850400	0.15839300
H	1.04922600	-4.65465800	0.22145600
H	0.88936300	-2.19407500	0.70930900
C	-0.95421100	-1.67479000	2.16499100
H	-0.49889300	-0.68339100	2.24901200
H	-1.91254000	-1.66119200	2.68774500
H	-0.30304300	-2.39987800	2.66786500
H	0.11346000	-4.57682500	-2.03803600
P	0.77824100	0.47415700	-1.41141000
P	3.53161300	-0.87636400	0.44835800
C	1.82550300	1.28160300	-2.69365600
C	1.27720200	1.62283300	-3.94032000
C	3.17265900	1.58416600	-2.45969000
C	2.05388500	2.24225500	-4.91463000
H	0.23089100	1.40784800	-4.15060900
C	3.95164300	2.20466200	-3.43317100
H	3.63891100	1.32894400	-1.50950600
C	3.39249900	2.53834900	-4.66354600
H	1.60685000	2.49786100	-5.87280700
H	4.99525500	2.42842300	-3.22063800
H	3.99569200	3.03041500	-5.42336500
C	5.17997700	-0.07932800	0.34803300
C	6.22927500	-0.72036000	-0.32819400
C	5.43310700	1.14888900	0.97505400
C	7.49331800	-0.14005100	-0.38007200
H	6.06246000	-1.67462400	-0.82345800
C	6.69800800	1.72622800	0.91901200
H	4.64161900	1.66220600	1.51686300
C	7.72994400	1.08544300	0.23767600

H	8.29381500	-0.64991900	-0.91134100
H	6.87216500	2.68216200	1.40770100
H	8.71663900	1.54057100	0.18793200
C	-0.86900200	1.15440900	-1.81883700
C	-1.02733900	2.53683300	-1.96783800
C	-1.97521400	0.31890400	-1.99549900
C	-2.28108800	3.07798700	-2.23415700
H	-0.16170300	3.19194000	-1.89993600
C	-3.22526100	0.86178500	-2.28287000
H	-1.86629300	-0.76211400	-1.93292200
C	-3.38745000	2.24209600	-2.38130400
H	-2.38938200	4.15651900	-2.33812800
H	-4.07585400	0.19651200	-2.42719100
H	-4.36967000	2.66354400	-2.58687000
C	3.61570400	-2.00285600	1.88582300
C	2.65636400	-1.93483200	2.90108000
C	4.64065200	-2.95508800	1.97711600
C	2.70108900	-2.81544600	3.97943200
H	1.86952700	-1.18100100	2.86116000
C	4.69103500	-3.82597400	3.05975700
H	5.39972300	-3.01737500	1.19743000
C	3.71779700	-3.76262100	4.05800900
H	1.94176700	-2.75385900	4.75678400
H	5.49250300	-4.55845200	3.12337200
H	3.75782900	-4.45037000	4.89974200
Rh	1.83493300	0.74917400	0.80345600
H	2.88618700	0.66674500	2.00927600
H	2.83078900	1.69601600	0.17387400
C	-0.39214800	1.50837700	3.52036300
C	0.99821200	1.56872000	3.60044100

C	0.71897100	2.67372100	1.44159800
C	1.62517600	2.23353200	2.44297100
S	-0.96848700	2.32081900	2.05376200
O	-1.69719100	1.37446700	1.18973200
O	-1.65536400	3.56647700	2.41623300
C	-7.20328600	-0.20573000	-0.10643000
H	-8.23063900	0.05170100	-0.35076900
C	-4.93156500	0.46347800	0.38457400
H	-4.18750900	1.24556600	0.51594100
C	-5.49773900	-1.88384300	0.30344400
H	-5.21458100	-2.92581300	0.39753900
C	-1.18193500	0.89026900	4.47790100
H	-2.26538200	0.85724700	4.38181600
C	-0.53218700	0.30954600	5.56611400
H	-1.11559000	-0.18422400	6.33897300
C	0.86116500	0.36341400	5.67283600
H	1.35016700	-0.09534900	6.52894600
C	1.63532700	0.98610300	4.69668600
H	2.72150600	1.00404000	4.77262000
C	0.95562700	3.86819400	0.60089500
C	-0.08926500	4.66602800	0.11029400
C	2.27349200	4.24706000	0.28447000
C	0.17488400	5.78602700	-0.67372100
H	-1.12191700	4.41452800	0.32956600
C	2.53312100	5.35725000	-0.50724100
H	3.11461600	3.66080100	0.65363000
C	1.48252300	6.13483400	-0.99451400
H	-0.65784900	6.38244900	-1.04133800
H	3.56359000	5.61440300	-0.74322000
H	1.68491800	7.00366100	-1.61667900

H	2.56490600	2.76248700	2.59413000

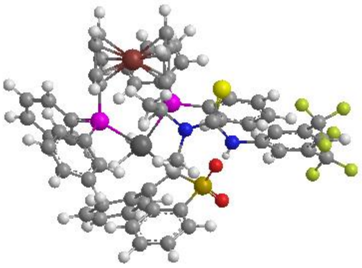

Rh(III)HH-**1m**-pro-(*S*) **III**:

Thermal correction to Gibbs Free Energy= 0.856984

Sum of electronic and thermal Free Energies= -4887.806304

Fe	-1.54599200	-3.56938500	0.59663800
S	3.29097800	-2.88152200	-0.63516500
N	1.41556100	-1.19075800	-1.58445300
N	3.44443700	-0.24485500	-1.17125200
F	5.83109800	3.23665800	1.41969400
F	6.75696800	1.74299800	2.68931100
F	7.95208900	2.79611800	1.22042400
F	8.78870900	-1.09874500	-2.44351600
F	7.73892900	-2.84753100	-1.71084100
F	9.17042300	-1.82013600	-0.43753900
C	0.32259700	-2.18110800	-1.43675700
C	2.69477100	-1.40134300	-1.15900200
C	4.78582500	-0.10197600	-0.79454200
C	6.42820900	1.17204600	0.43340100
C	7.10588400	-0.74740400	-0.83014900
C	6.74126700	2.24403800	1.42845600
C	8.19563200	-1.63248200	-1.35094800
C	0.18995600	-2.80460800	-0.06899100
C	0.33369200	-4.20457700	0.17832500
C	0.02886700	-4.47678600	1.53579000
C	-0.30978600	-3.25058600	2.16308000

C	-0.22358000	-2.19310600	1.18798200
C	-3.16194400	-2.62996800	-0.14458400
C	-2.78217100	-3.72872000	-0.99280200
C	-2.82965200	-4.92438500	-0.22349600
C	-3.23868400	-4.58735900	1.09883400
C	-3.44065000	-3.17977700	1.16064100
H	2.89397100	0.60163300	-1.03102400
H	-0.00607900	-5.45642900	1.99954100
H	-0.64103400	-3.13361300	3.18890500
H	-2.49975800	-3.64900400	-2.03679300
H	-2.54875900	-5.91093600	-0.57574800
H	-3.32649900	-5.27331600	1.93420700
H	-3.74897500	-2.62141800	2.03776400
C	0.39030900	-3.20977800	-2.56087900
H	0.33295600	-2.71584100	-3.53680800
H	1.33113400	-3.76662100	-2.50938000
H	-0.43807500	-3.92133800	-2.49680300
H	-0.58094400	-1.56657600	-1.59359800
C	1.10243500	-0.14938400	-2.55791700
H	0.49470800	0.65440100	-2.12568500
H	2.01589500	0.28662100	-2.96492700
H	0.53942800	-0.59217600	-3.38867400
H	0.59459300	-4.94176100	-0.57265700
P	-0.61844600	-0.46894800	1.61214400
P	-3.34255800	-0.89687800	-0.65293600
C	-1.63160800	-0.61205300	3.13954300
C	-1.01201600	-0.88599800	4.36849500
C	-3.02268100	-0.45895300	3.11417700
C	-1.76732300	-1.01164000	5.53051000
H	0.06973900	-1.00162500	4.41814200

C	-3.77956900	-0.58379700	4.27652000
H	-3.53638200	-0.24602900	2.17805500
C	-3.15249000	-0.86028100	5.48850400
H	-1.26836100	-1.22537100	6.47319100
H	-4.85969100	-0.45930900	4.22726300
H	-3.73989500	-0.95271900	6.39937100
C	-5.05170700	-0.54719100	-0.09591700
C	-6.00103200	-1.58134600	-0.07141800
C	-5.44285500	0.73774700	0.29758400
C	-7.30976600	-1.32530600	0.32379100
H	-5.71708300	-2.59538000	-0.34752900
C	-6.75213100	0.98944400	0.69828000
H	-4.72249200	1.55289900	0.29880800
C	-7.68844300	-0.04078800	0.71041000
H	-8.03352800	-2.13712800	0.33677300
H	-7.03396200	1.99459300	1.00571800
H	-8.71073500	0.15371200	1.02737100
C	0.95218700	0.17586700	2.28274500
C	0.94139100	1.37113200	3.01538100
C	2.16208500	-0.49260700	2.07986200
C	2.12742200	1.90441200	3.50727600
H	-0.00226400	1.87258200	3.23041300
C	3.34702100	0.04014900	2.58429800
H	2.18659100	-1.43570700	1.53418200
C	3.33581000	1.24452300	3.28372600
H	2.10693700	2.83391500	4.07249300
H	4.28456500	-0.49176300	2.42493400
H	4.26452100	1.66282300	3.66587700
C	-3.40213200	-1.02078200	-2.47827400
C	-2.55702600	-0.23322900	-3.26902900

C	-4.28043200	-1.91517100	-3.10598800
C	-2.57313400	-0.34586100	-4.65725900
H	-1.88444200	0.48903300	-2.80460600
C	-4.30427200	-2.01851200	-4.49240800
H	-4.94670600	-2.53789200	-2.51046000
C	-3.44672800	-1.23959000	-5.26989600
H	-1.90410400	0.27158900	-5.25376600
H	-4.99386000	-2.71265300	-4.96725800
H	-3.46441400	-1.32843600	-6.35381900
Rh	-1.82541200	0.83678200	-0.05643700
H	-2.95372500	1.36260200	-1.07714700
H	-2.79832900	1.21803600	1.04594500
C	0.28684300	3.24546900	-1.78136100
C	-1.10983500	3.24177200	-1.82074000
C	-0.77801900	2.66267200	0.50000700
C	-1.73857800	3.05006900	-0.47857800
H	-0.89755900	2.93286600	1.54592400
C	-2.95328900	3.83265200	-0.11472300
C	-4.16432400	3.71824200	-0.81028800
C	-2.85790300	4.77896800	0.91417700
C	-5.25011700	4.52252800	-0.48463400
H	-4.27404200	2.96058700	-1.58633500
C	-3.94748300	5.58133600	1.24301800
H	-1.91452900	4.90809700	1.44242700
C	-5.14601700	5.45571700	0.54624000
H	-6.18522700	4.40686900	-1.02862500
H	-3.85314300	6.31000900	2.04505900
H	-5.99839200	6.07950200	0.80609000
S	0.86777400	2.98270800	-0.12548600
O	1.77238900	1.82235100	-0.12028000

O	1.35013200	4.21788700	0.50383900
C	7.43660500	0.30818700	0.01708100
H	8.46175200	0.45331500	0.34683700
C	5.11289200	0.98006600	0.02641400
H	4.32659000	1.64881300	0.36936800
C	5.79422500	-0.95865600	-1.24317700
H	5.55250400	-1.78198900	-1.90587300
C	1.08078100	3.38250200	-2.91009700
H	2.16654800	3.36868800	-2.83838900
C	0.43696600	3.52732800	-4.13711300
H	1.02467400	3.63528600	-5.04504900
C	-0.95873700	3.53649600	-4.20393500
H	-1.44864800	3.64861900	-5.16816400
C	-1.73799600	3.39981500	-3.05745700
H	-2.82258700	3.39984200	-3.13563300

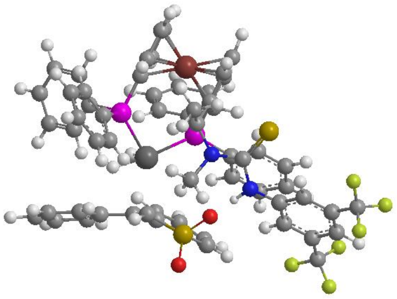

Rh(III)HH-**1m**-pro-(*R*) **IV**:

Thermal correction to Gibbs Free Energy= 0.855831

Sum of electronic and thermal Free Energies= -4887.790620

Fe	1.63346300	3.29458000	1.49130300
S	-3.15750800	3.29098400	-0.19192500
N	-1.26996100	1.84388300	-1.45788900
N	-3.29064200	0.82758800	-1.29871500
F	-5.52707900	-3.30789400	0.12406900
F	-6.99279100	-2.43980500	1.47105700

F	-7.58264100	-3.02817600	-0.52537500
F	-8.74962000	1.89518300	-2.17613400
F	-7.63826600	3.38835800	-1.06275100
F	-8.98742600	2.03369900	-0.02920200
C	-0.15492800	2.70773200	-1.00214900
C	-2.55579100	1.95624600	-1.01370900
C	-4.63324400	0.55742400	-1.01117700
C	-6.25102400	-1.09447600	-0.31224400
C	-6.96512600	1.13115300	-0.83884600
C	-6.57912000	-2.46837100	0.18212800
C	-8.07775100	2.11649400	-1.02282100
C	-0.07366900	2.89755800	0.49142400
C	-0.17167500	4.16220300	1.14715400
C	0.02145500	3.98056600	2.54091200
C	0.24253400	2.59803700	2.77617300
C	0.20172700	1.90215300	1.51766600
C	3.27823100	2.53456500	0.61061400
C	3.05063800	3.89267900	0.18380400
C	3.07178900	4.73043800	1.33309900
C	3.30396200	3.91311800	2.47653500
C	3.42095300	2.56089700	2.04639800
H	-2.73234000	-0.01918000	-1.40647200
H	0.06177800	4.76566100	3.28814800
H	0.47961200	2.14991400	3.73477900
H	2.88744300	4.21378400	-0.83935100
H	2.88633600	5.79898700	1.33747000
H	3.32500800	4.24947800	3.50742700
H	3.58628200	1.70136300	2.68762200
C	-0.11467100	4.01039000	-1.78959900
H	-0.07851000	3.80375400	-2.86499000

H	-1.00522400	4.61190300	-1.58394600
H	0.77468700	4.59427000	-1.52921900
H	0.73699300	2.12562700	-1.28996000
C	-0.96300100	1.04068800	-2.63620600
H	-0.57491200	0.04398700	-2.38322000
H	-1.85681600	0.90886800	-3.25028900
H	-0.21430700	1.56699100	-3.23777300
H	-0.32897800	5.11146400	0.64669500
P	0.45497800	0.10132800	1.35499100
P	3.40545000	1.09208600	-0.48064900
C	1.38210600	-0.33945300	2.88878700
C	0.73599800	-0.34842500	4.13517900
C	2.74025400	-0.67707300	2.84689000
C	1.43220800	-0.66888400	5.29671600
H	-0.32383900	-0.10562300	4.19863800
C	3.43943500	-0.99894800	4.00827700
H	3.27419800	-0.68153400	1.89847900
C	2.78639600	-0.99521500	5.23765600
H	0.91119600	-0.66608500	6.25185100
H	4.49571500	-1.25424300	3.94311200
H	3.32754200	-1.25032800	6.14630200
C	5.01396700	0.35689600	0.00434800
C	5.97382200	1.12713800	0.67725800
C	5.32083600	-0.97010000	-0.32607200
C	7.20516100	0.57434700	1.01975000
H	5.76205000	2.16057700	0.94361600
C	6.55340600	-1.51719500	0.01665200
H	4.59253700	-1.58094900	-0.85723800
C	7.49625000	-0.74831200	0.69577700
H	7.93617000	1.18342100	1.54689700

H	6.77192000	-2.55082300	-0.24419300
H	8.45569800	-1.17975300	0.97294100
C	-1.21322300	-0.51382700	1.81211000
C	-1.37145600	-1.84907700	2.19962800
C	-2.32360300	0.33547900	1.84058200
C	-2.61496600	-2.32240200	2.60653200
H	-0.50979700	-2.51876800	2.20923000
C	-3.56949600	-0.14236400	2.24182600
H	-2.22243400	1.38355800	1.56616100
C	-3.71941100	-1.47177600	2.62741000
H	-2.72068900	-3.36069300	2.91687100
H	-4.42396500	0.53392000	2.25177100
H	-4.69137400	-1.84508500	2.94394400
C	3.70200600	1.89327900	-2.10025200
C	2.85849800	1.65829400	-3.19006300
C	4.76918100	2.79088100	-2.24323500
C	3.05913100	2.32448300	-4.39720300
H	2.04736000	0.93566000	-3.10250600
C	4.97916300	3.44177400	-3.45380600
H	5.43577500	2.98580000	-1.40312000
C	4.11975500	3.21613000	-4.52961900
H	2.38872500	2.13735400	-5.23339900
H	5.81461700	4.13077400	-3.55608500
H	4.28242100	3.73326100	-5.47272600
Rh	1.76048700	-0.47984300	-0.65681600
H	2.78645400	-0.76269000	-1.84369900
H	2.70199900	-1.33921300	0.17829500
C	-0.58809400	-3.89395000	-0.31794800
C	0.79686000	-3.91995500	-0.09537300
C	0.75162400	-2.34867500	-1.88807200

C	1.54908900	-3.25300500	-1.18367400
H	0.96411100	-2.04599000	-2.91061200
C	2.88446100	-3.66302600	-1.62191900
C	3.57463000	-4.72556100	-1.00837800
C	3.48318800	-3.06175900	-2.75077700
C	4.82534700	-5.12745800	-1.46341900
H	3.12274200	-5.28332900	-0.19753600
C	4.72759700	-3.46523400	-3.20419700
H	2.97425300	-2.25753500	-3.27523800
C	5.41385700	-4.49288600	-2.55250700
H	5.33263900	-5.95153100	-0.96796000
H	5.16579700	-2.97732500	-4.07135800
H	6.39376800	-4.80559100	-2.90618000
S	-0.96205900	-2.77402600	-1.64097000
O	-1.44194900	-3.43808300	-2.85813400
O	-1.78790600	-1.68451700	-1.09323800
C	-7.27615600	-0.15674400	-0.41162100
H	-8.29969200	-0.42583500	-0.16164600
C	-4.94055900	-0.74812200	-0.61046400
H	-4.14091700	-1.47858500	-0.50969000
C	-5.65792300	1.49661100	-1.14871100
H	-5.43693700	2.50025300	-1.49119500
C	-1.50636900	-4.54107400	0.48690900
H	-2.57306200	-4.48966500	0.27736800
C	-1.01561000	-5.23939100	1.59162600
H	-1.70200700	-5.78519800	2.23427500
C	0.34247500	-5.18837100	1.90491200
H	0.70344500	-5.66920200	2.81035900
C	1.25227100	-4.52607500	1.08012900
H	2.29429600	-4.45918700	1.37884700

7. Reference

(1) Kuhn, M.; Falk, F. C.; Paradies, J. *Org. Lett.* **2011**, *13*, 4100.

(2) Tosatti, P.; Pfaltz, A. *Angew. Chem. Int. Ed.* **2017**, *56*, 4579.

(3) Antonow, D.; Marrafa, T.; Dawood, I.; Ahmed, T.; Haque, M. R.; Thurston, D. E.; Zinzalla, G. *Chem. Commun.*, **2010**, *46*, 2289.

(4) Xiong, R.; Patel, H. K.; Gutgesell, L. M.; Zhao, J.; Delgado-Rivera, L.; Pham, T. N. D.; Zhao, H.; Carlson, K.; Martin, T.; Katzenellenbogen, J. A.; Moore, T. W.; Tonetti, D. A.; Thatcher, G. R. J. *J. Med. Chem.* **2016**, *59*, 219.

(5) Kim, S.; Yang, J.; Dininno, F. *Tetrahedron Lett.* **1999**, *40*, 2909.

(6) Urban, S.; Beiring, B.; Ortega, N.; Paul, D.; Glorius, F. *J. Am. Chem. Soc.* **2012**, *134*, 15241.

(7) Cabiddu, S.; Cancellu, D.; Floris, G.; Gelli, G.; Melis, S. *Synthesis* **1988**, 888.

(8) Milner, P. J.; Yang, Y.; Buchwald, S. L. *Organometallics* **2015**, *34*, 4775.

(9) Masuya, Y.; Tobisu, M.; Chatani, N. *Org. Lett.* **2016**, *18*, 4312.

(10) Fredrich, S.; A.; Bonasera, V.; Valderrey, Hecht, S. *J. Am. Chem. Soc.* **2018**, *140*, 6432.

(11) Jeong, Y.-C.; Park, D. G.; Kim, E.; Yang, S. I.; Ahn, K.-H. *Macromolecules* **2006**, *39*, 3106.

(12) Zhao, Y.; Truhlar, D. G. *J. Chem. Phys.*, **2006**, *125*, 194101.

(13) Gaussian 09, Revision D.01, Frisch, M. J.; Trucks, G. W.; Schlegel, H. B.; Scuseria, G. E.; Robb, M. A.; Cheeseman, J. R.; Scalmani, G.; Barone, V.; Mennucci, B.; Petersson, G. A.; Nakatsuji, H.; Caricato, M.; Li, X.; Hratchian, H. P.; Izmaylov, A. F.; Bloino, J.; Zheng, G.; Sonnenberg, J. L.; Hada, M.; Ehara, M.; Toyota, K.; Fukuda, R.; Hasegawa, J.; Ishida, M.; Nakajima, T.; Honda, Y.; Kitao, O.; Nakai, H.; Vreven, T.; Montgomery, J. A.; Peralta, Jr., J. E.; Ogliaro, F.; Bearpark, M.; Heyd, J. J.; Brothers, E.; Kudin, K. N.; Staroverov, V. N.; Keith, T.; Kobayashi, R.; Normand, J.; Raghavachari, K.; Rendell, A.; Burant, J. C.; Iyengar, S. S.; Tomasi, J.; Cossi, M.; Rega, N.; Millam, J. M.; Klene, M.; Knox, J. E.; Cross, J. B.; Bakken, V.; Adamo, C.; Jaramillo, J.; Gomperts, R.; Stratmann, R. E.; Yazyev, O.; Austin, A. J.; Cammi, R.; Pomelli, C.; Ochterski, J. W.; Martin, R. L.;

Morokuma, K.; Zakrzewski, V. G.; Voth, G. A.; Salvador, P.; Dannenberg, J. J.; Dapprich, S.; Daniels, A. D.; Farkas, O.; Foresman, J. B.; Ortiz, J. V.; Cioslowski, J.; Fox, D. J.; Gaussian, Inc., Wallingford CT, 2013.

(14) Grimme, S.; Antony, J.; Ehrlich, S.; Krieg, H. *J. Chem. Phys.* **2010**, *132*, 154104.

(15) Marenich, A. V.; Cramer, C. J.; Truhlar, D. G. *J. Phys. Chem. B* **2009**, *113*, 6378.

8. NMR spectra

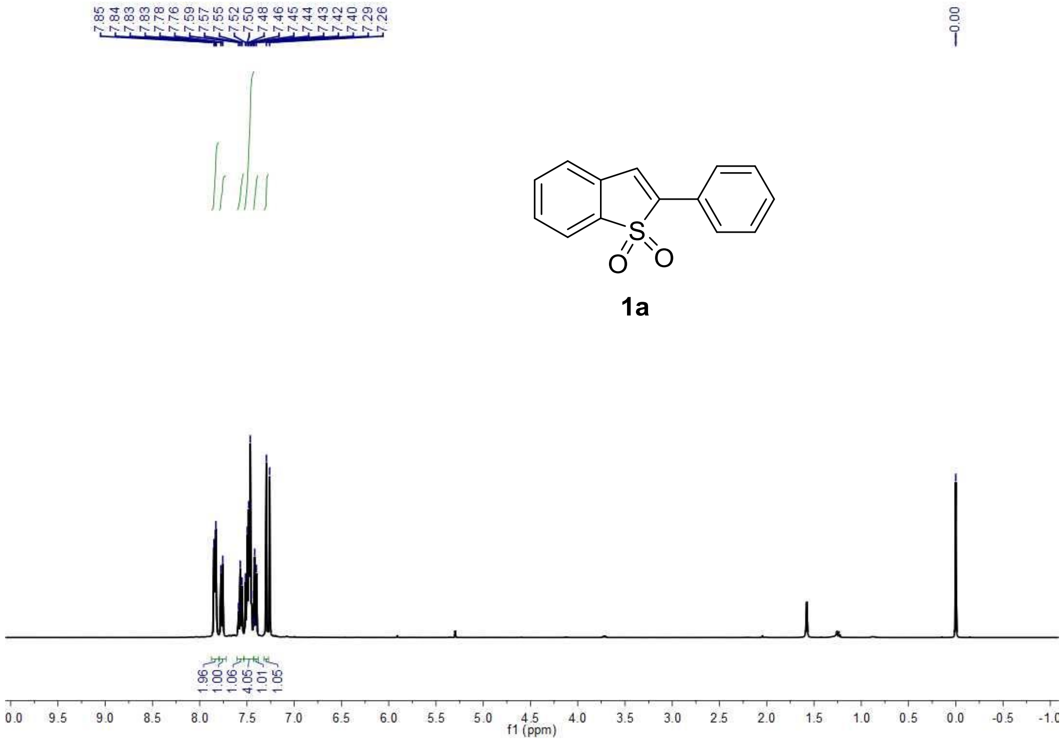

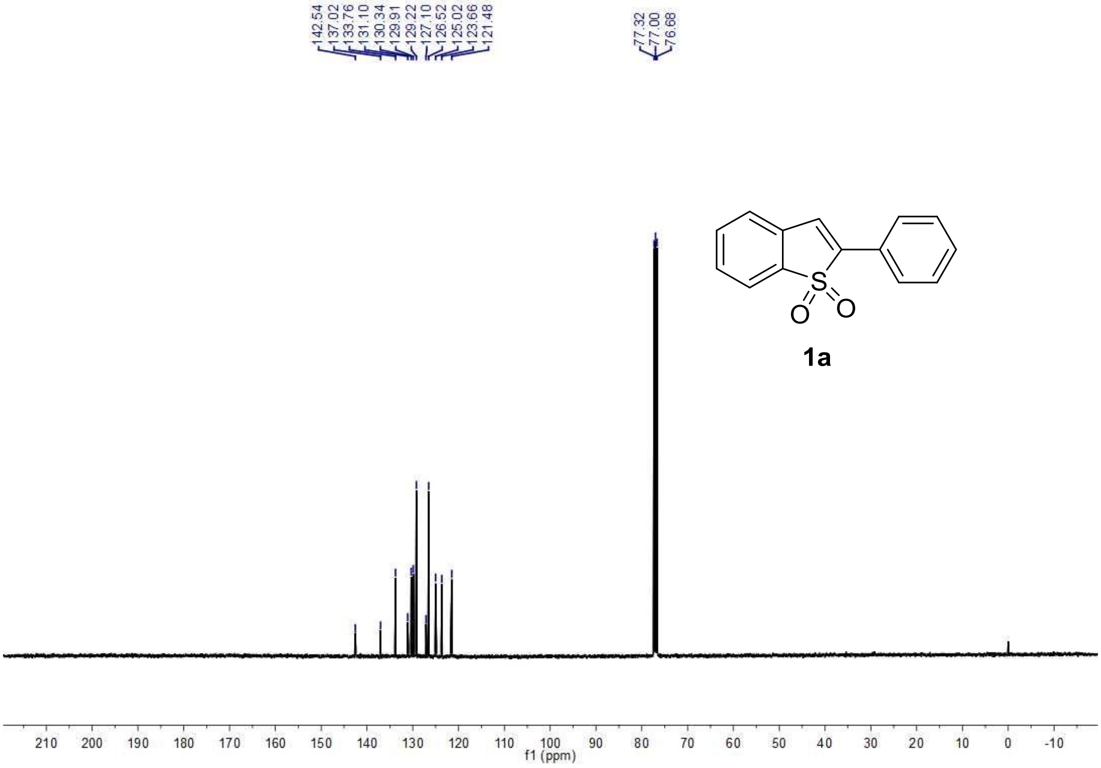

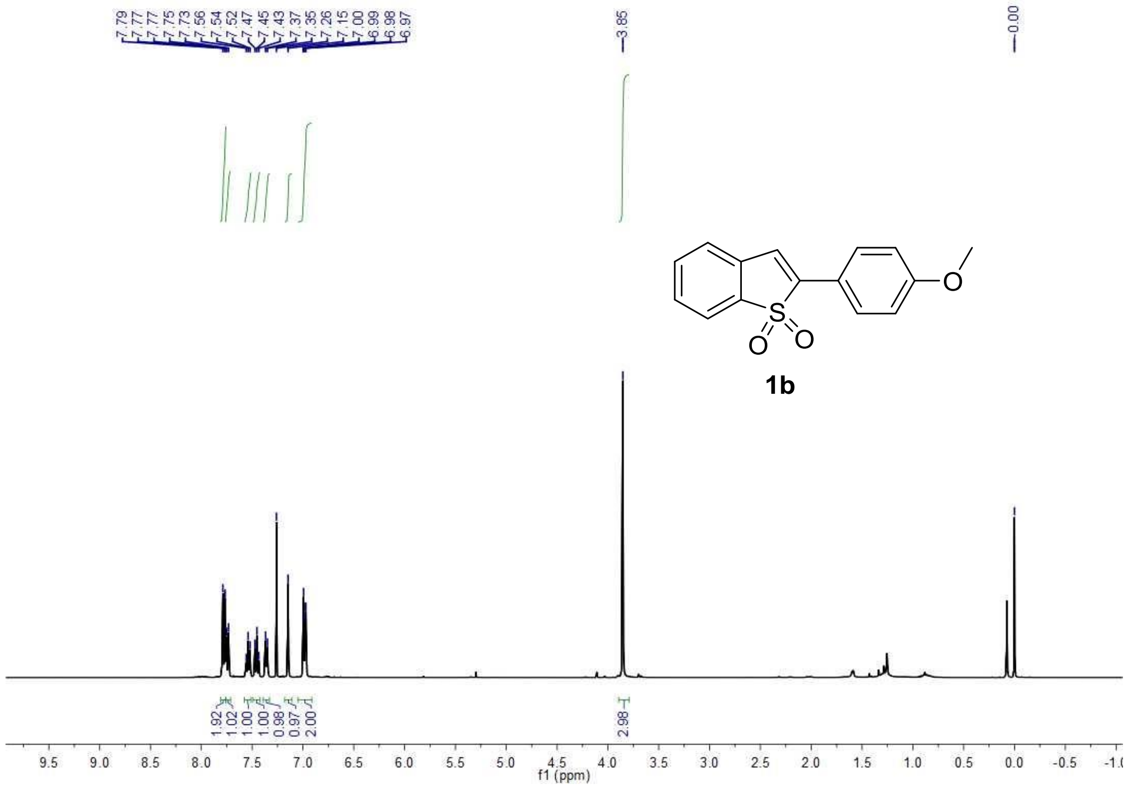

1b

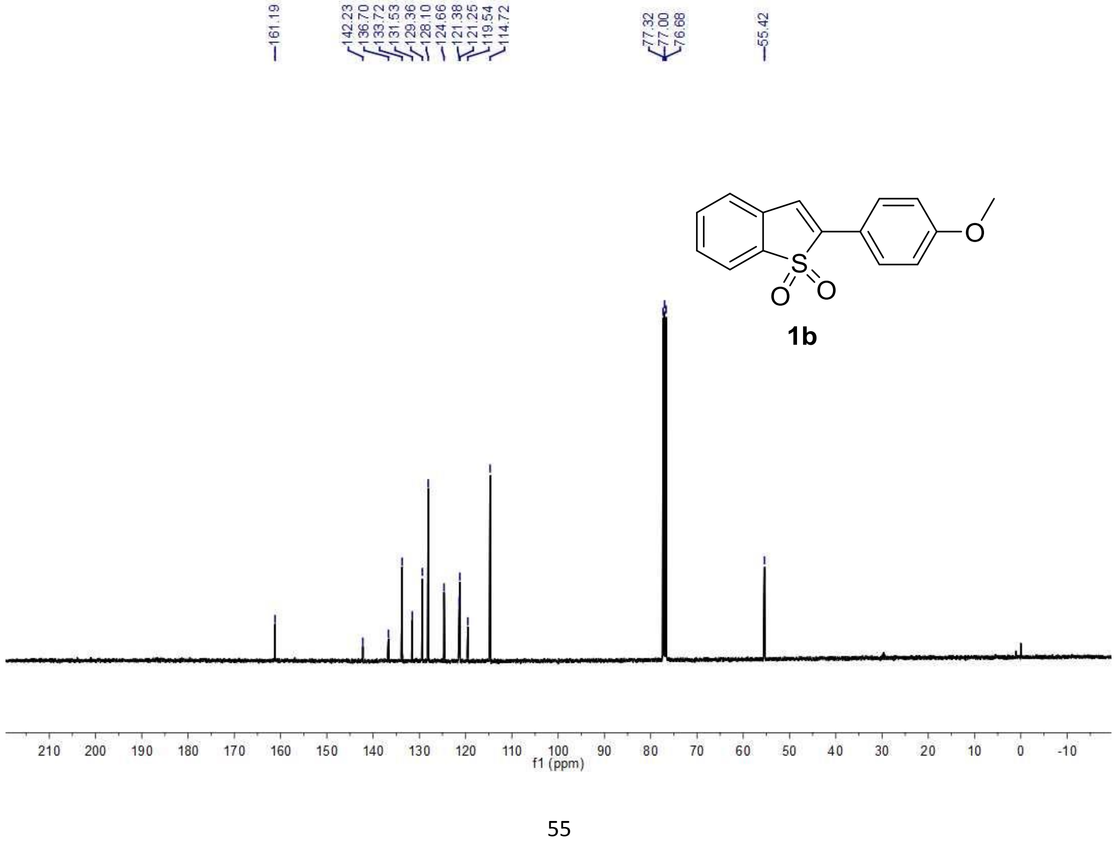

1b

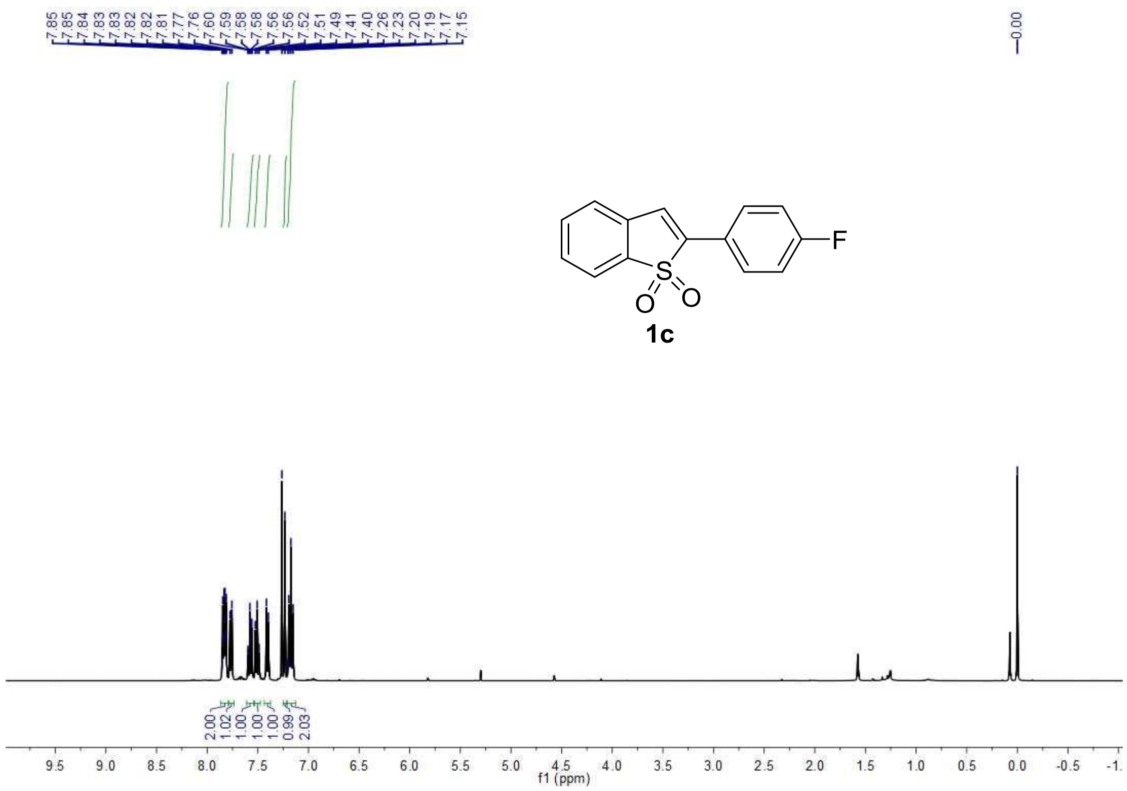
7.85
7.85
7.84
7.83
7.83
7.82
7.82
7.81
7.77
7.76
7.60
7.59
7.58
7.58
7.56
7.56
7.52
7.51
7.49
7.41
7.40
7.26
7.23
7.20
7.19
7.17
7.15
0.00
F
S
O
O
1c
2.00
1.02
1.00
1.00
1.00
0.99
2.03
f1 (ppm)

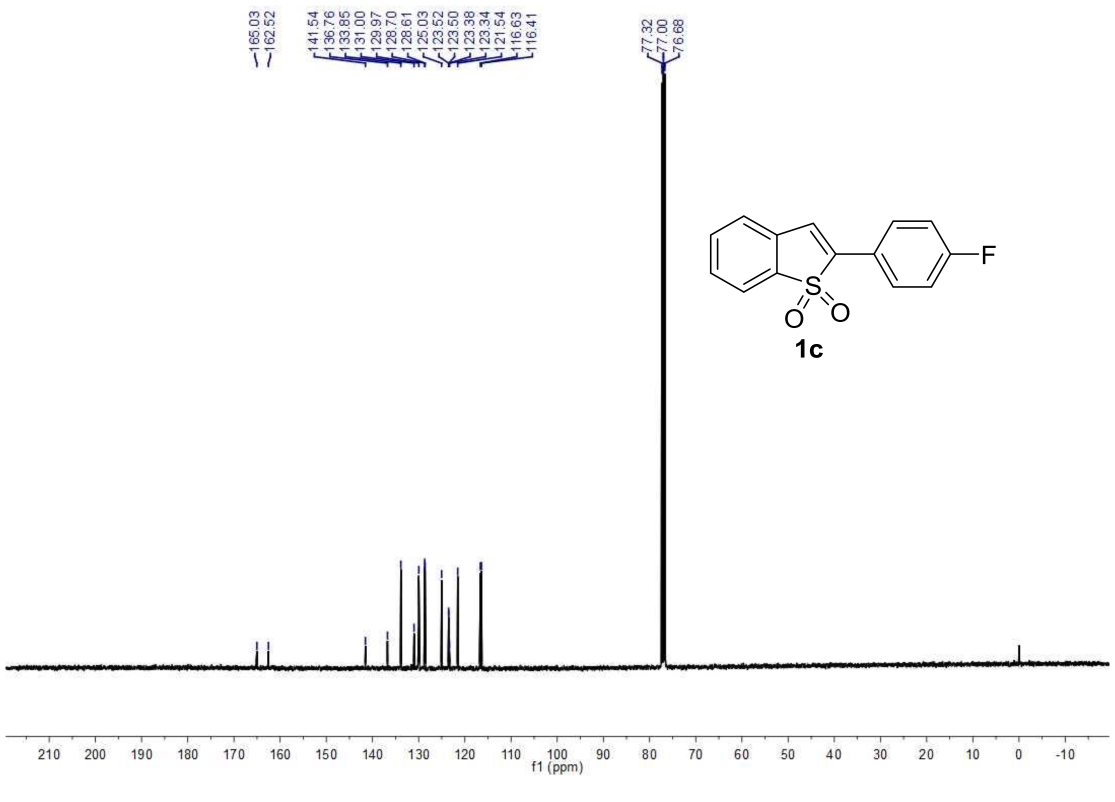
165.03
162.52
141.54
136.76
133.85
131.00
129.97
128.70
128.61
125.03
123.52
123.50
123.38
123.34
121.54
116.63
116.41
77.32
77.00
76.68
F
S
O
O
1c
f1 (ppm)

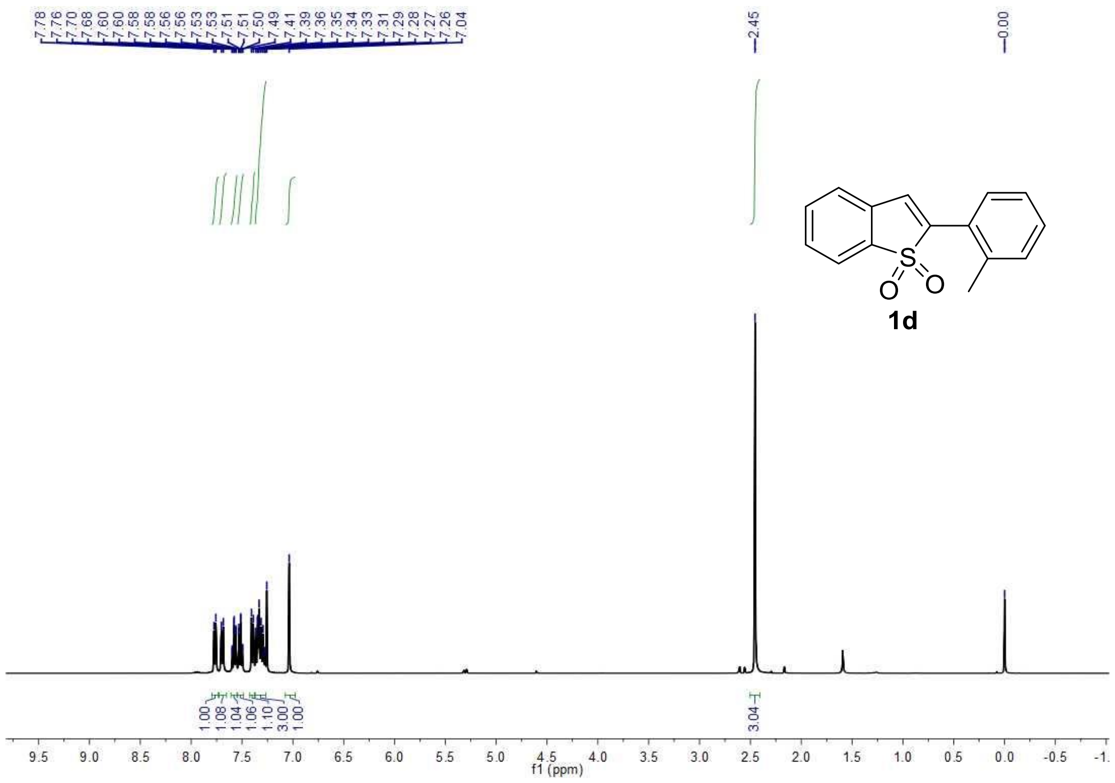
7.78
7.76
7.70
7.68
7.60
7.58
7.58
7.56
7.56
7.53
7.53
7.51
7.51
7.50
7.49
7.41
7.39
7.36
7.35
7.34
7.33
7.31
7.29
7.28
7.27
7.26
7.04
2.45
0.00
1d
1.00
1.08
1.04
1.06
1.10
3.00
1.00
3.04
f1 (ppm)

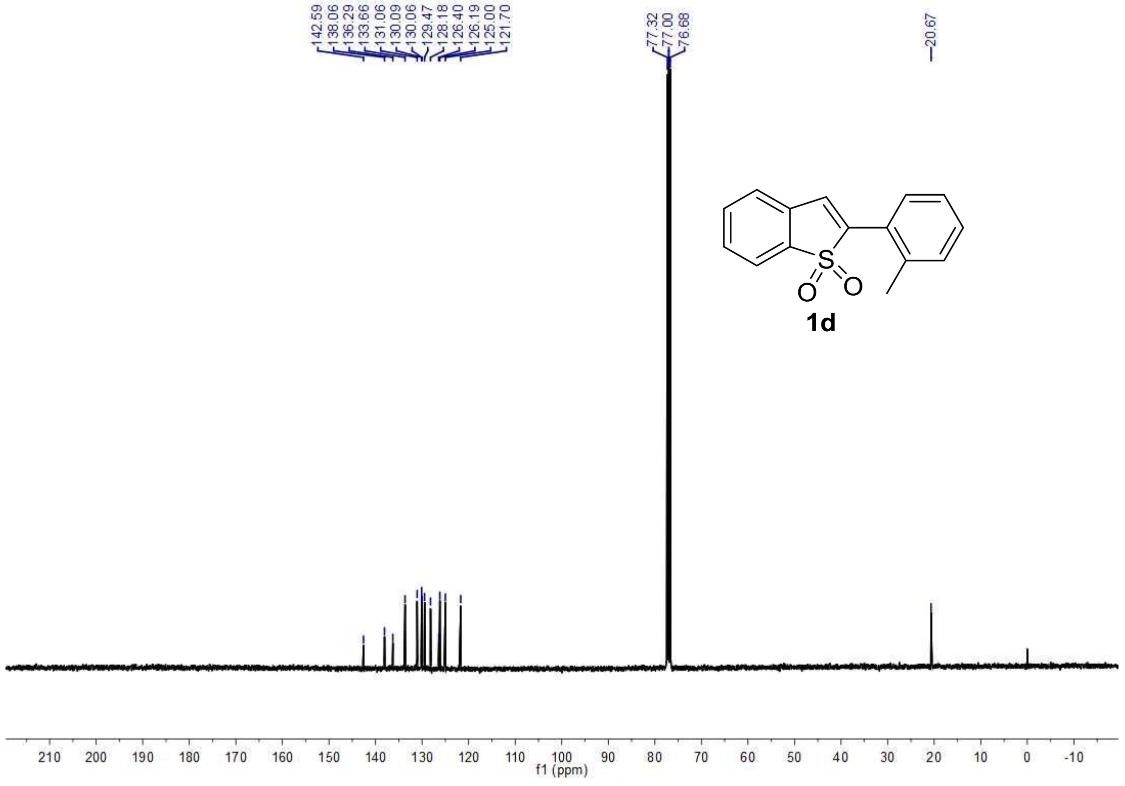
142.59
138.06
136.29
133.66
131.06
130.09
130.06
129.47
128.18
126.40
126.19
125.00
121.70
77.32
77.00
76.68
20.67
1d
f1 (ppm)

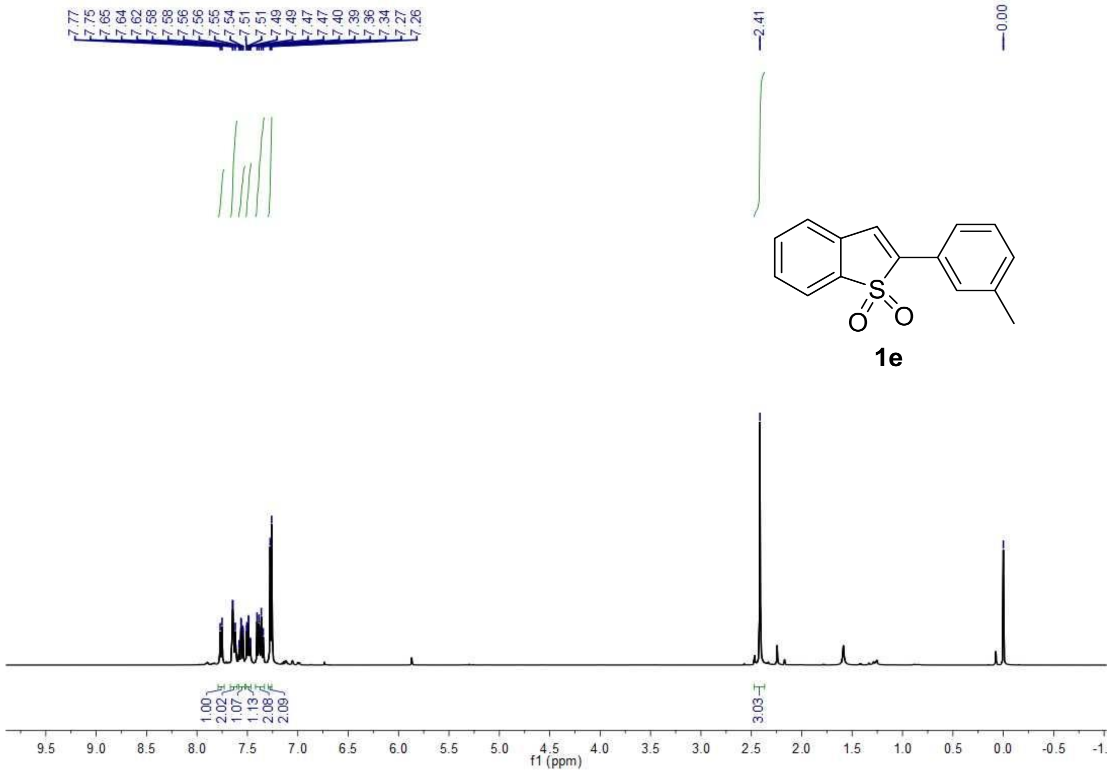

7.77 7.75 7.65 7.64 7.62 7.58 7.58 7.56 7.55 7.54 7.51 7.51 7.49 7.49 7.47 7.47 7.40 7.39 7.36 7.34 7.27 7.26
2.41
0.00
1e
1.00 2.02 1.07 1.13 2.08 2.09
3.03
f1 (ppm)

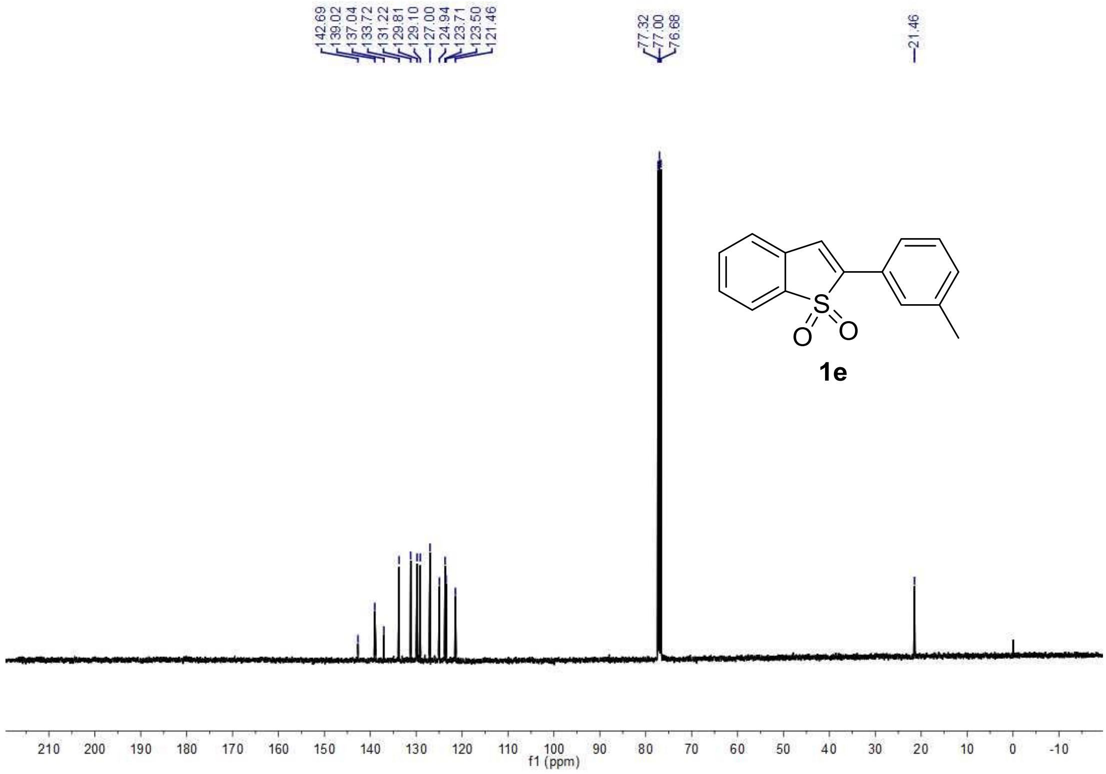

142.69 139.02 137.04 133.72 131.22 129.81 129.10 127.00 124.94 123.71 123.50 121.46
77.32 77.00 76.68
21.46
1e
f1 (ppm)

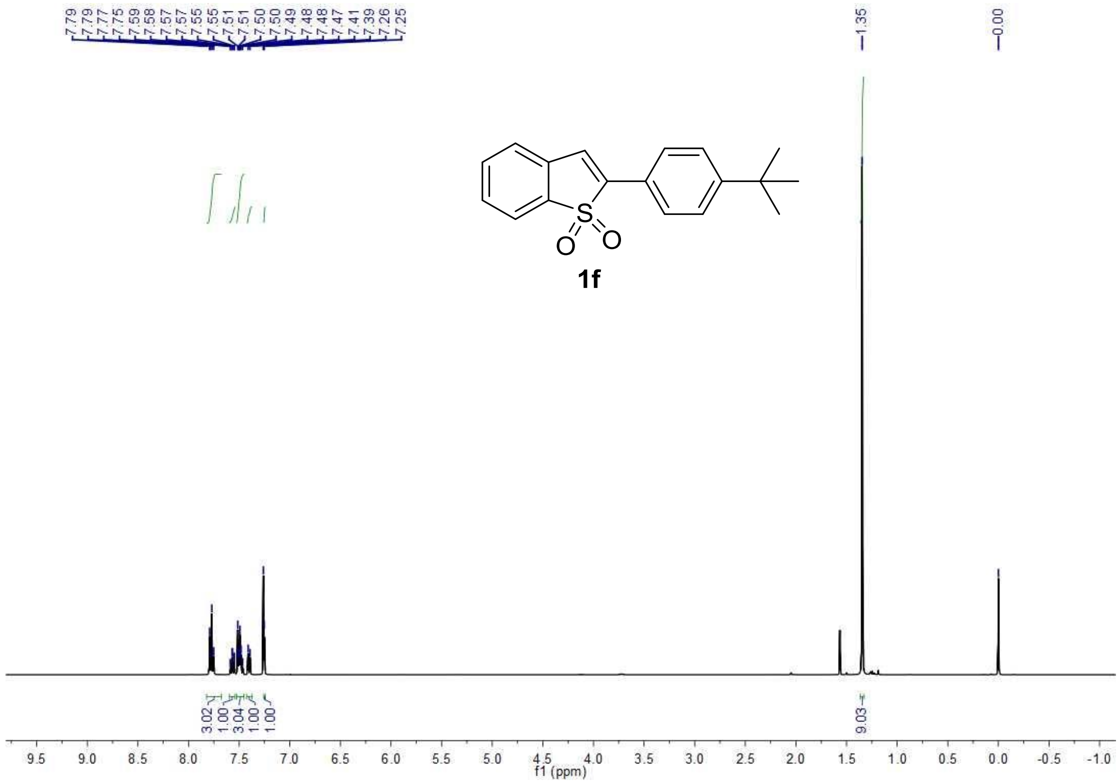
7.79 7.79 7.77 7.75 7.59 7.58 7.57 7.57 7.55 7.55 7.51 7.51 7.50 7.50 7.49 7.48 7.47 7.41 7.39 7.26 7.25
1.35
0.00
1f
3.02 1.00 3.04 1.00 1.00
9.03
f1 (ppm)

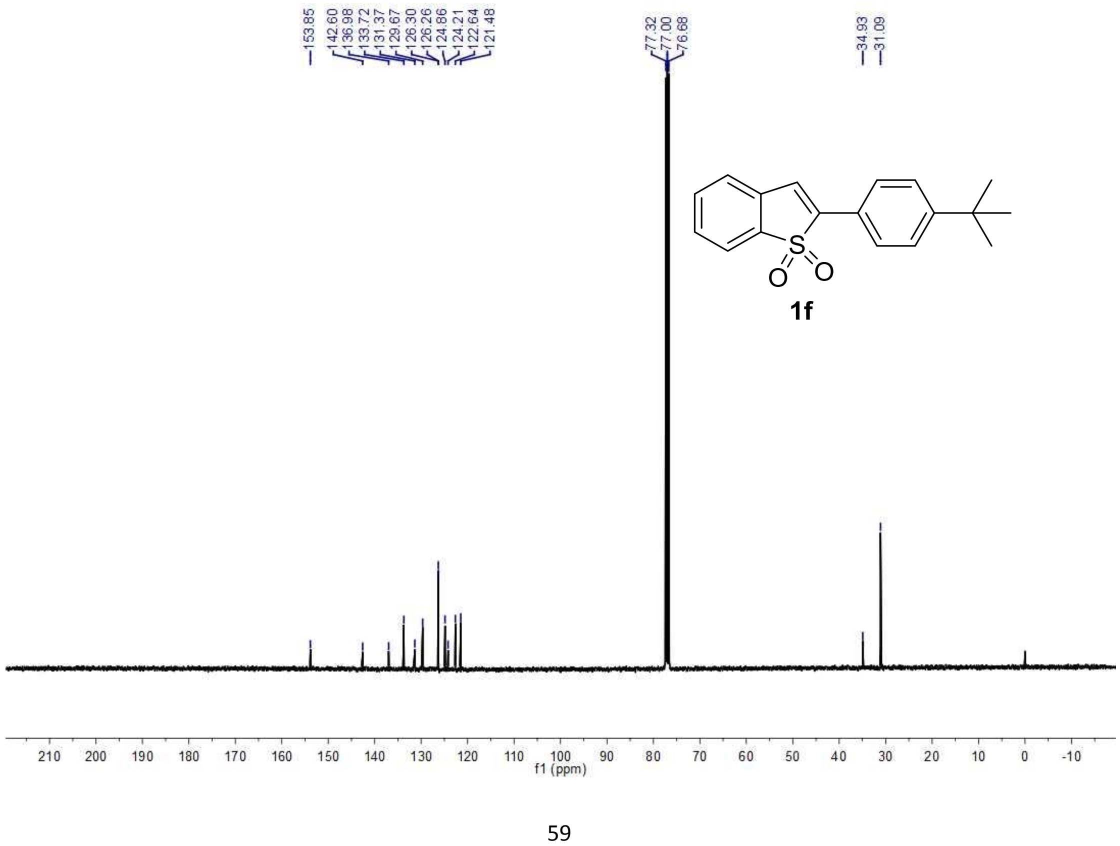
153.85
142.60 136.98 133.72 131.37 129.67 126.30 126.26 124.86 124.21 122.64 121.48
77.32 77.00 76.68
34.93 31.09
1f
f1 (ppm)

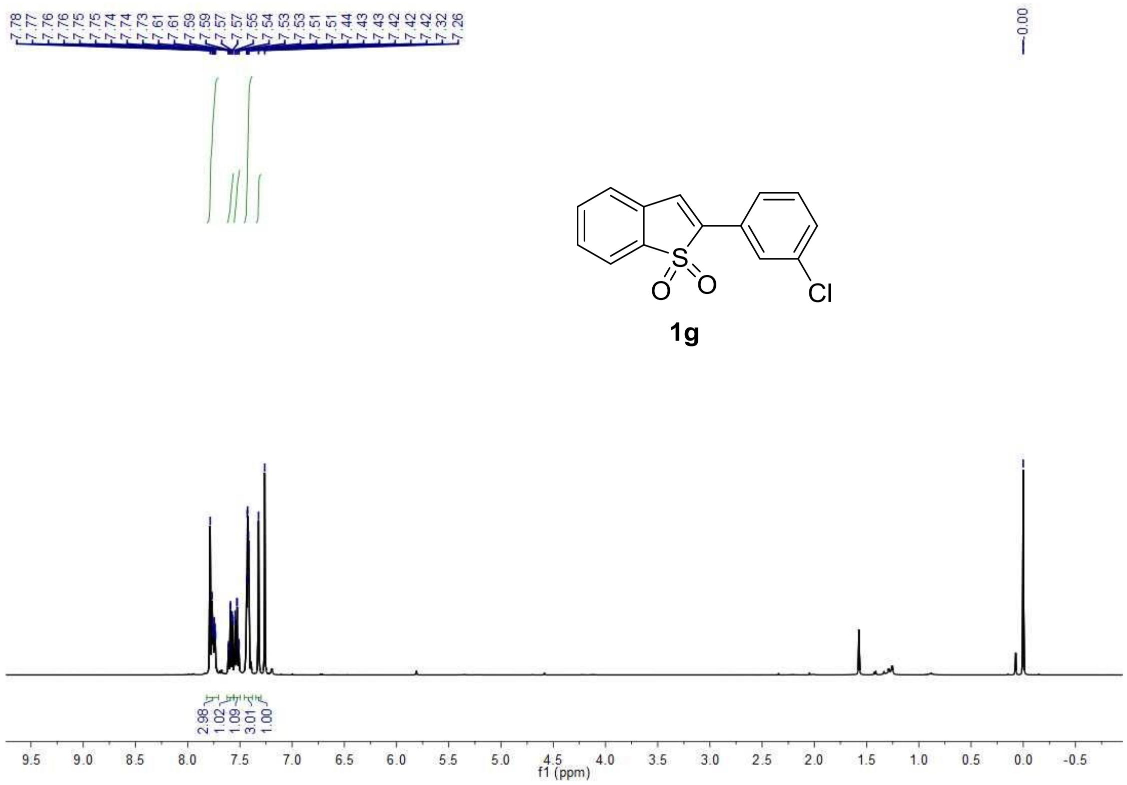
7.78 7.77 7.76 7.75 7.75 7.74 7.74 7.73 7.61 7.59 7.59 7.57 7.57 7.55 7.54 7.53 7.53 7.51 7.51 7.44 7.43 7.43 7.42 7.42 7.42 7.32 7.26
0.00
Cl
1g
2.98 1.02 1.09 3.01 1.00
f1 (ppm)

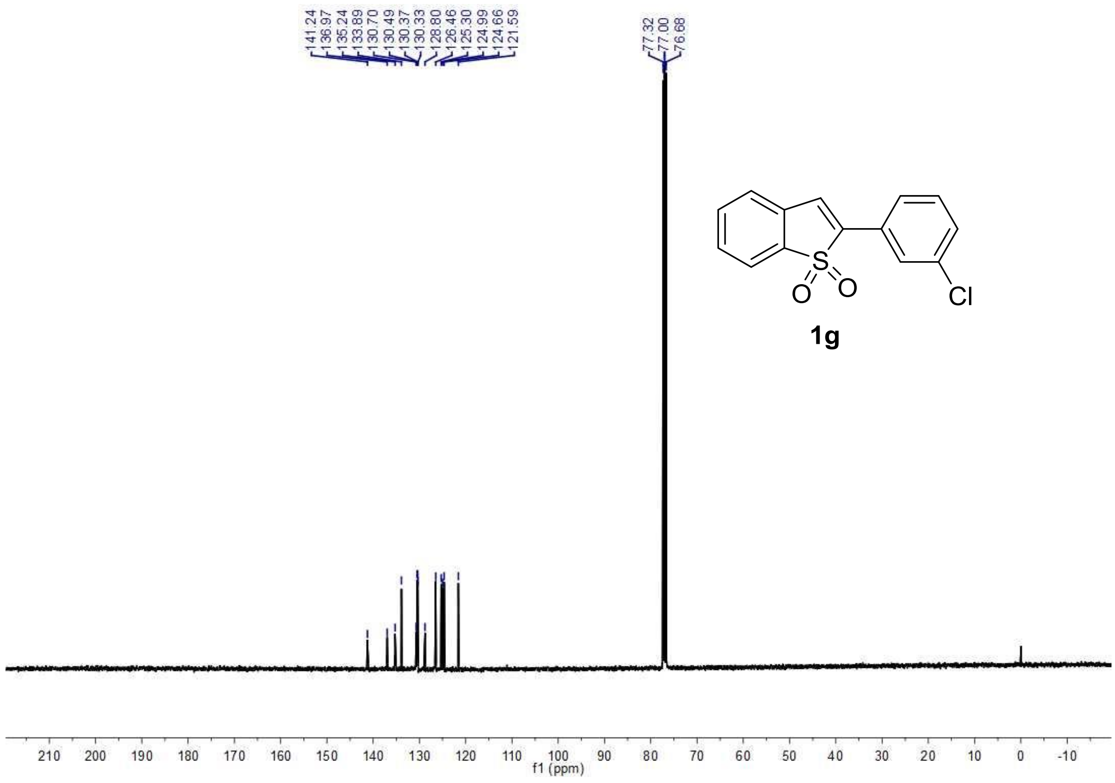
141.24 136.97 135.24 133.89 130.70 130.49 130.37 130.33 128.80 126.46 125.30 124.99 124.66 121.59
77.32 77.00 76.68
Cl
1g
f1 (ppm)

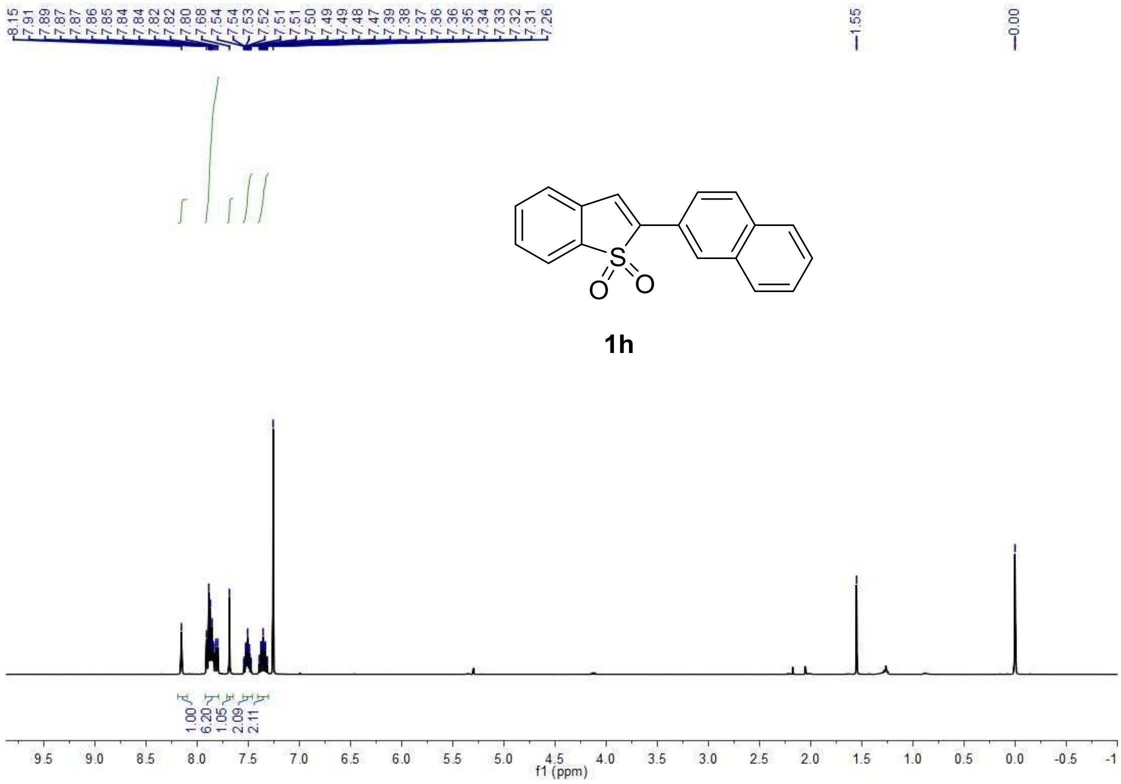
8.15 7.91 7.89 7.87 7.86 7.85 7.84 7.84 7.82 7.80 7.68 7.54 7.54 7.53 7.52 7.51 7.50 7.49 7.49 7.48 7.47 7.39 7.38 7.37 7.36 7.36 7.35 7.34 7.33 7.32 7.31 7.26
1.55
0.00
1h
1.00 6.20 1.05 2.09 2.11
f1 (ppm)

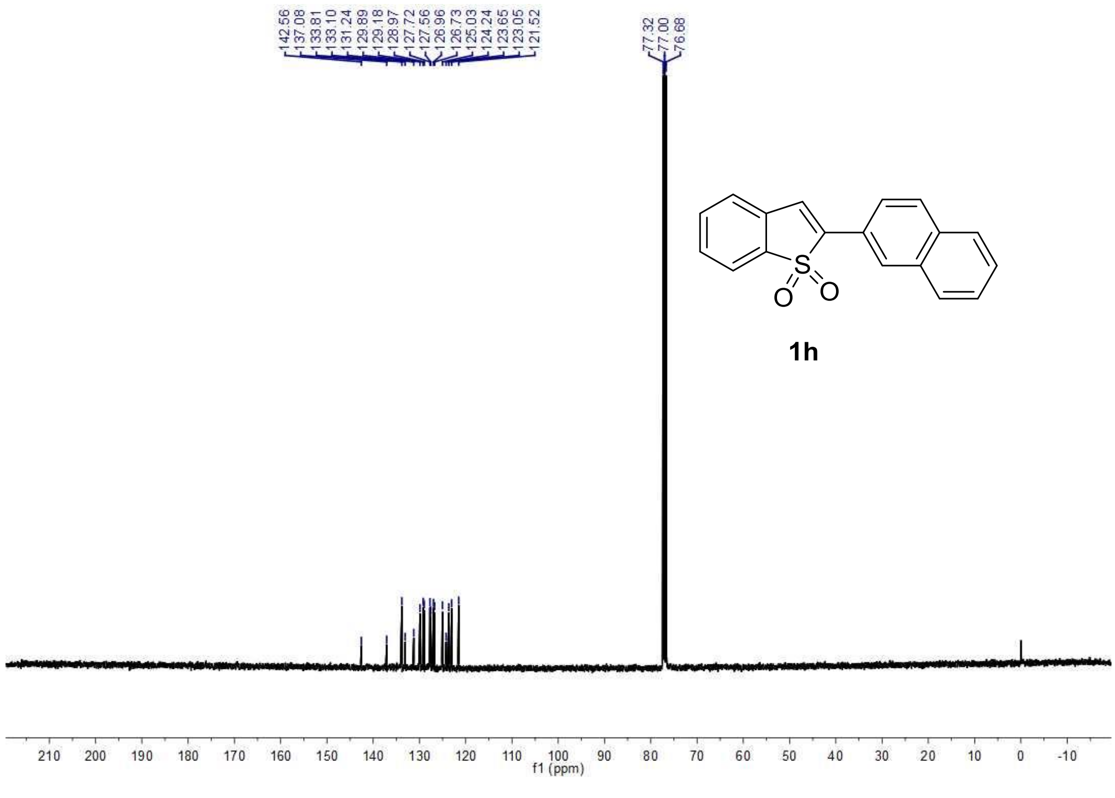
142.56 137.08 133.81 133.10 131.24 129.89 129.18 128.97 127.72 127.56 126.96 126.73 125.03 124.24 123.65 123.05 121.52
77.32 77.00 76.68
1h
f1 (ppm)

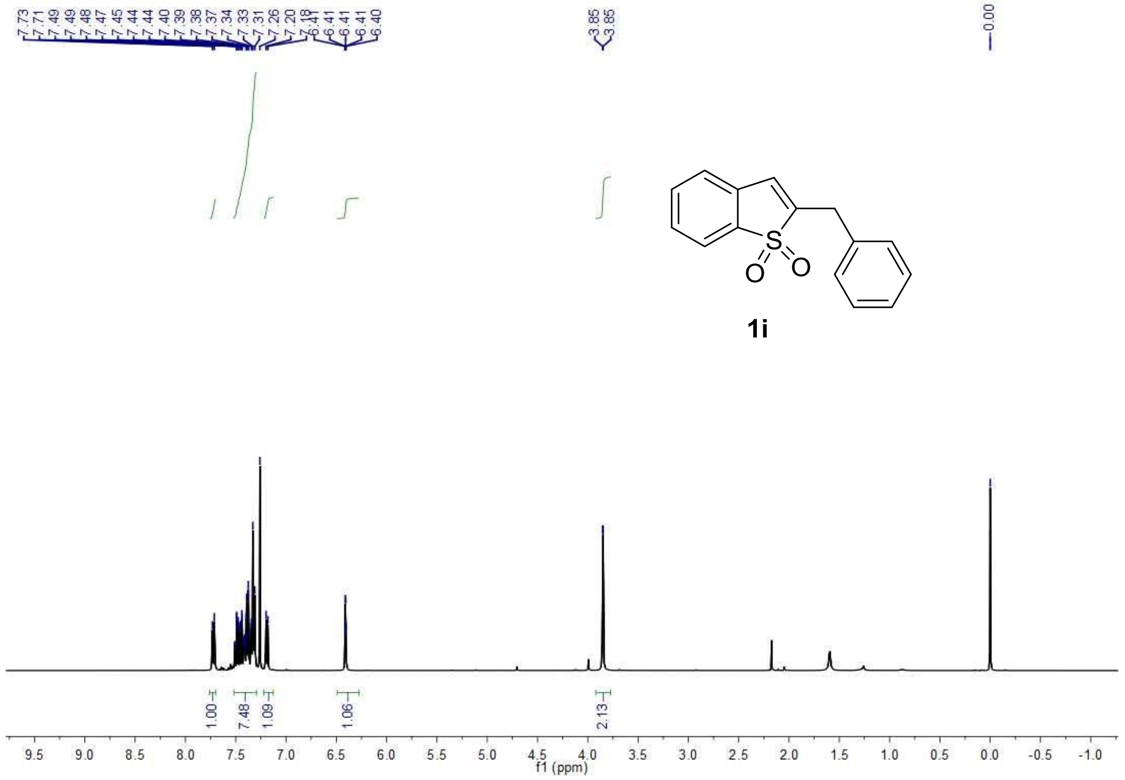
7.73 7.71 7.49 7.49 7.48 7.47 7.45 7.44 7.44 7.40 7.39 7.38 7.37 7.34 7.33 7.31 7.26 7.20 7.18 6.41 6.41 6.41 6.40
3.85 3.85
0.00
1i
1.00 7.48 1.09 1.06 2.13
9.5 9.0 8.5 8.0 7.5 7.0 6.5 6.0 5.5 5.0 4.5 4.0 3.5 3.0 2.5 2.0 1.5 1.0 0.5 0.0 -0.5 -1.0
f1 (ppm)

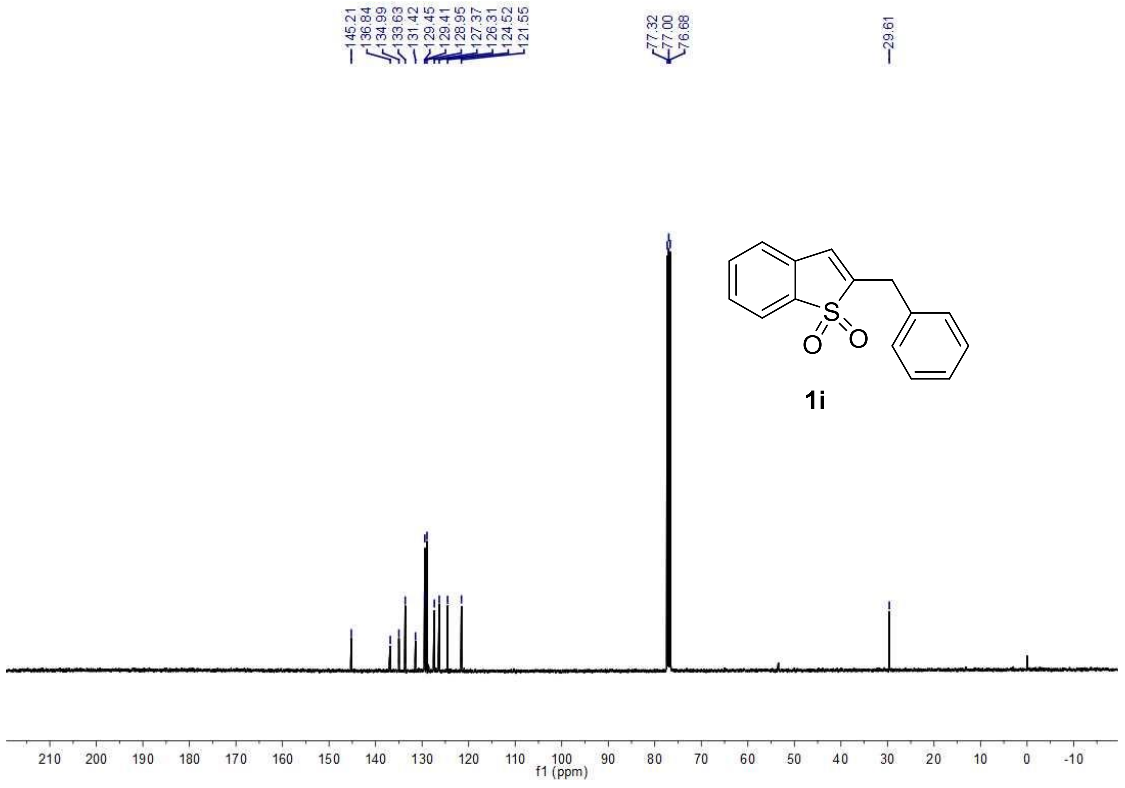
145.21 136.84 134.99 133.63 131.42 129.45 129.41 128.95 127.37 126.31 124.52 121.55
77.32 77.00 76.68
29.61
1i
210 200 190 180 170 160 150 140 130 120 110 100 90 80 70 60 50 40 30 20 10 0 -10
f1 (ppm)

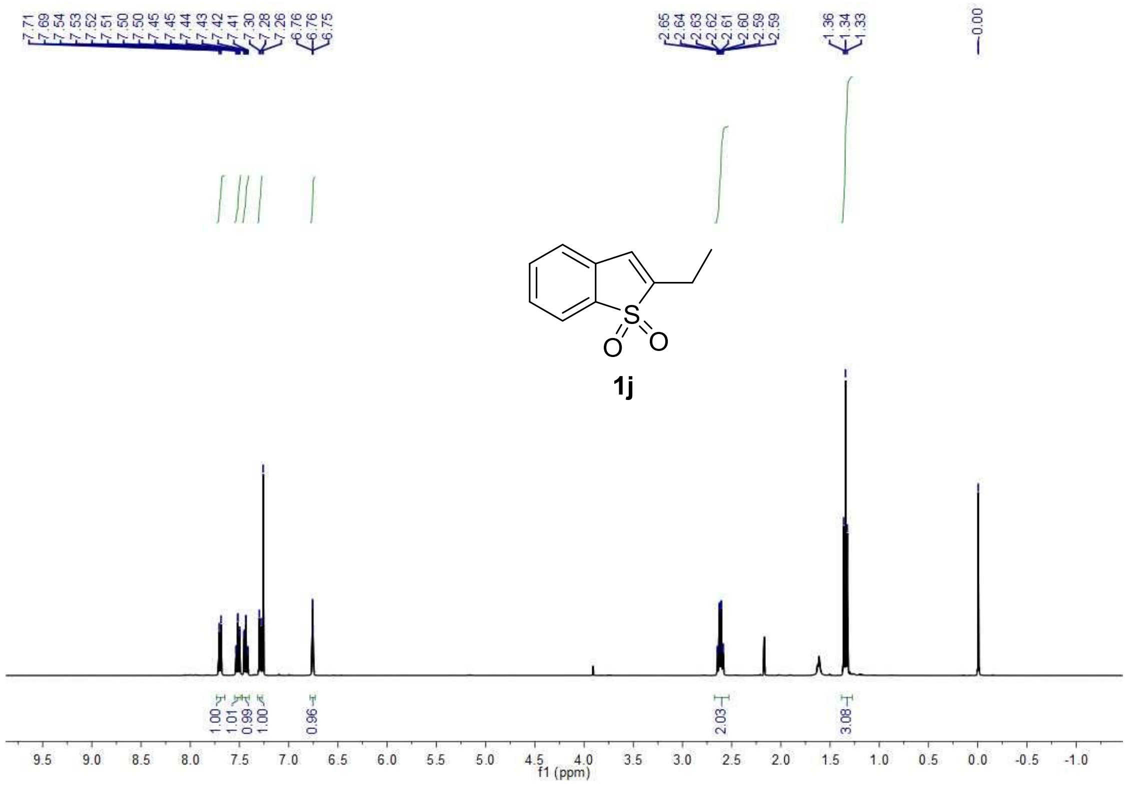
7.71 7.69 7.54 7.53 7.52 7.51 7.50 7.50 7.45 7.44 7.43 7.42 7.41 7.30 7.28 7.26 6.76 6.76 6.75
2.65 2.64 2.63 2.62 2.61 2.60 2.59 2.59
1.36 1.34 1.33
0.00
1j
1.00 1.01 0.99 1.00 0.96 2.03 3.08
f1 (ppm)

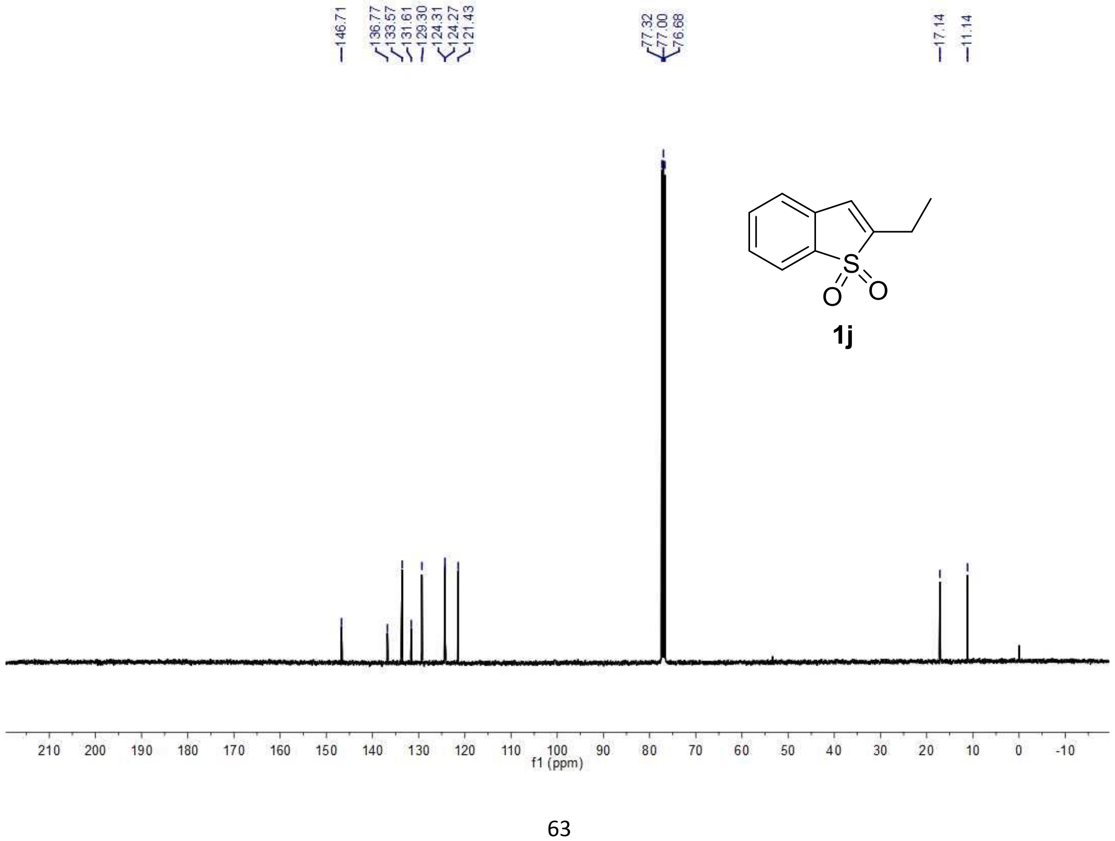
146.71
136.77 133.57 131.61 129.30 124.31 124.27 121.43
77.32 77.00 76.68
17.14
11.14
1j
f1 (ppm)

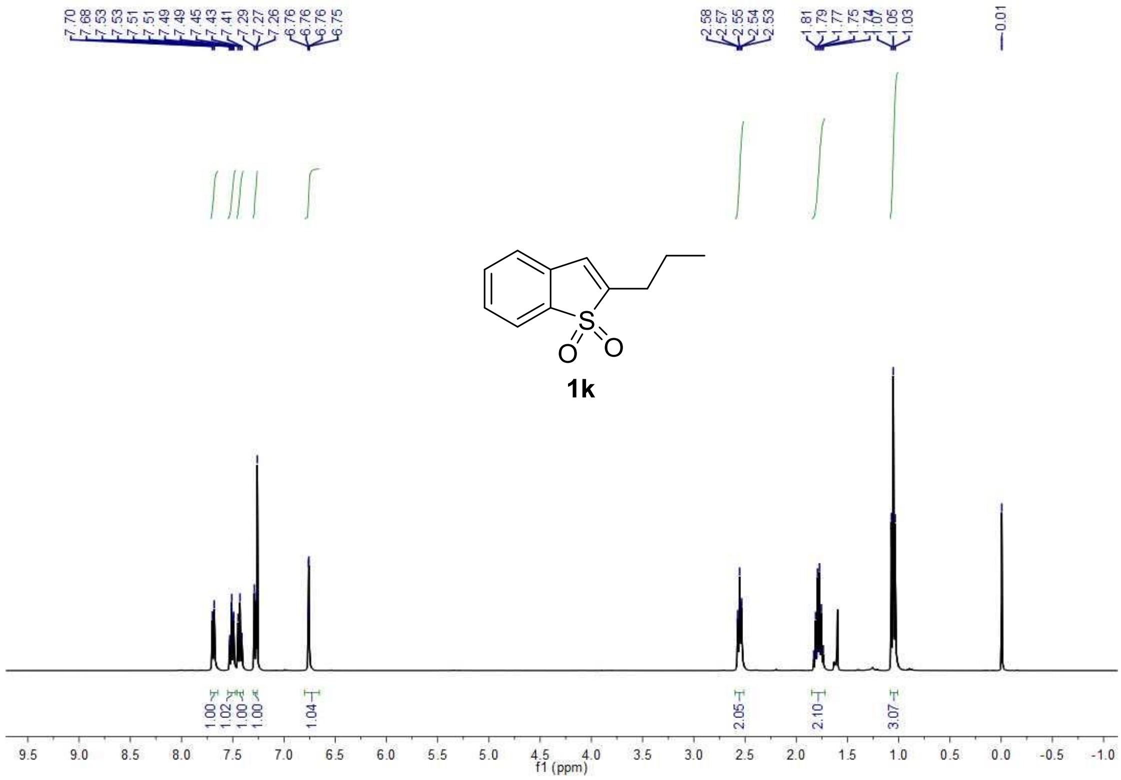
7.70 7.68 7.53 7.51 7.51 7.49 7.45 7.43 7.41 7.29 7.27 7.26 6.76 6.76 6.76 6.75
2.58 2.57 2.55 2.54 2.53
1.81 1.79 1.77 1.75 1.74 1.07 1.05 1.03
0.01
1k
1.00 1.02 1.00 1.00 1.04 2.05 2.10 3.07
f1 (ppm)

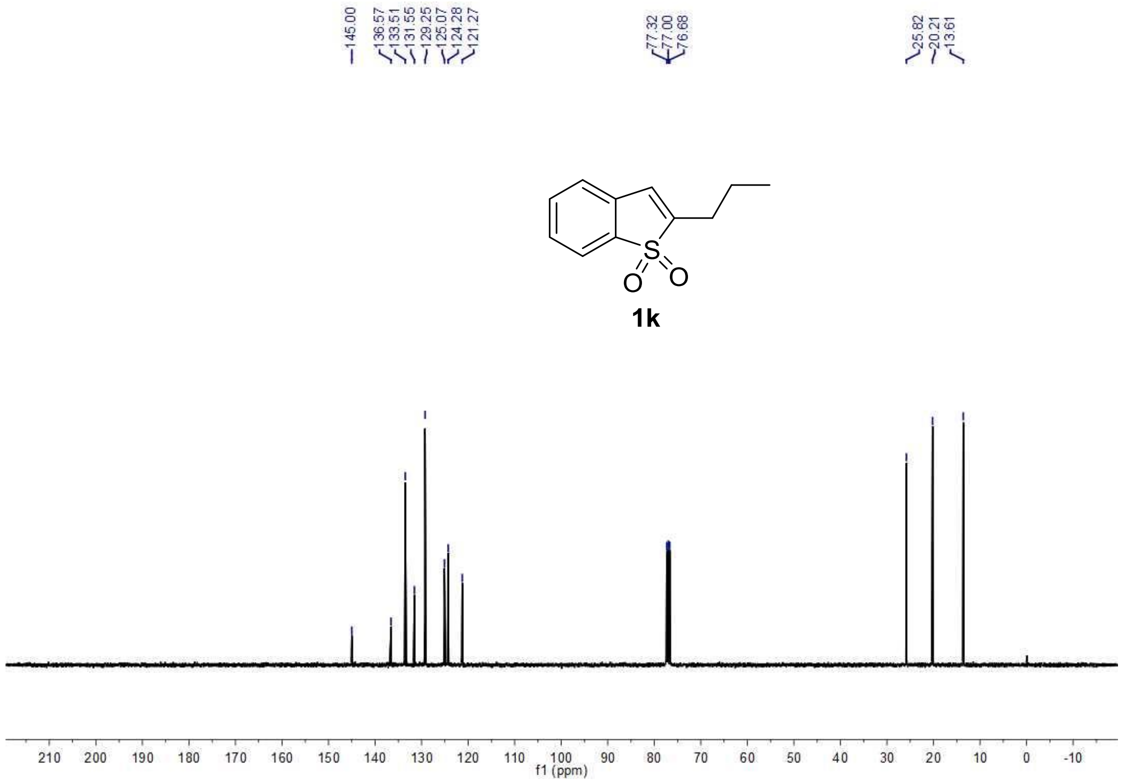
145.00
136.57 133.51 131.55 129.25 125.07 124.28 121.27
77.32 77.00 76.68
25.82 20.21 13.61
1k
f1 (ppm)

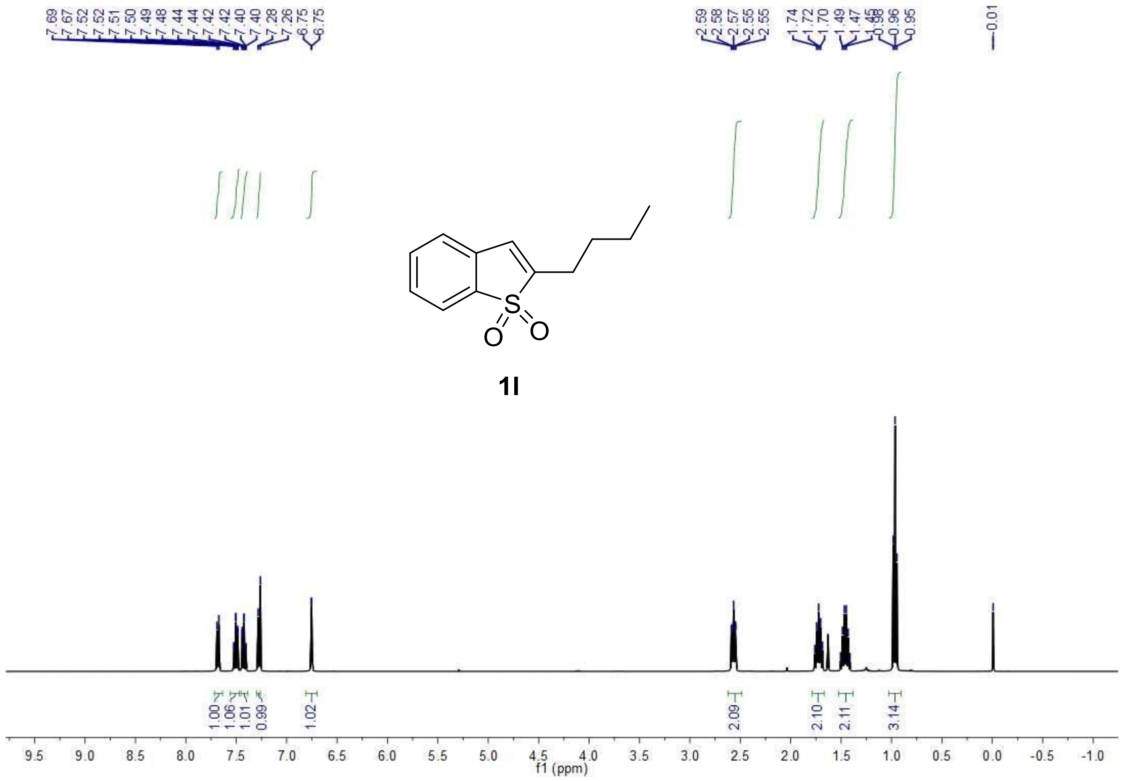
7.69 7.67 7.52 7.51 7.50 7.49 7.48 7.44 7.42 7.42 7.40 7.40 7.28 7.26 6.75 6.75
2.59 2.58 2.57 2.55 2.55 1.74 1.72 1.70 1.49 1.47 1.45 0.98 0.96 0.95
-0.01
1l
1.00 1.06 1.01 0.99 1.02 2.09 2.10 2.11 3.14
f1 (ppm)

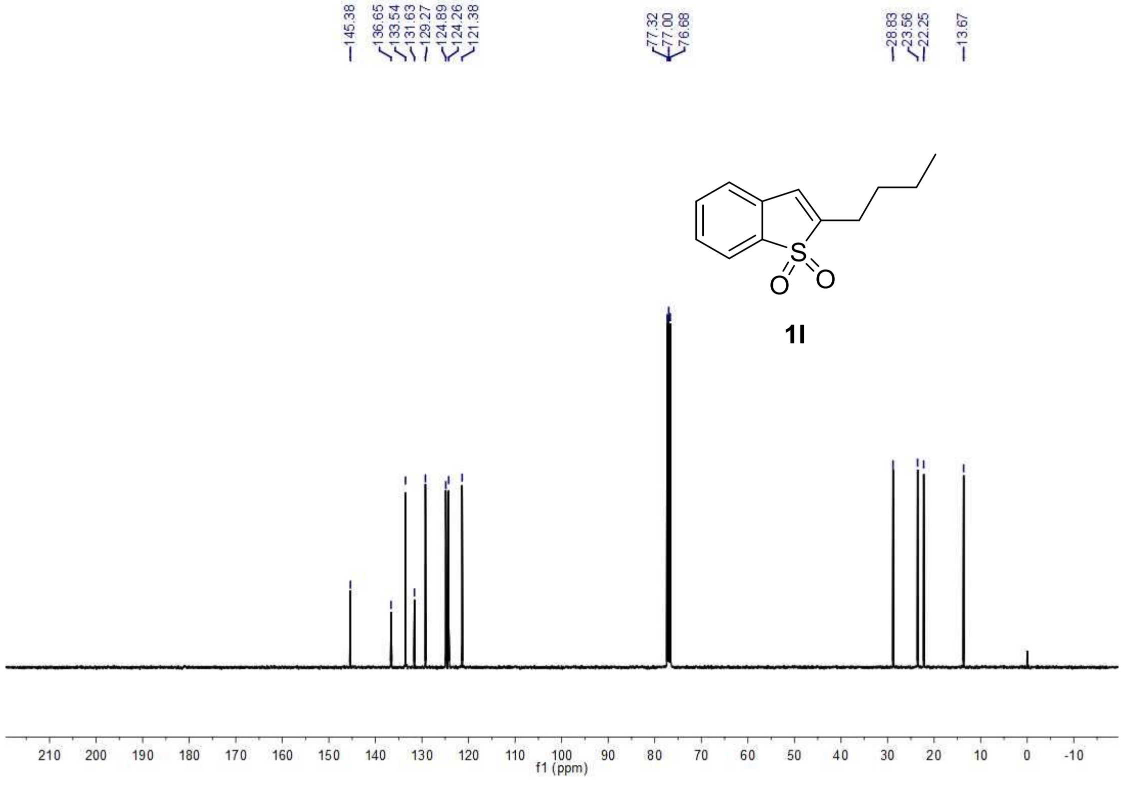
145.38 136.65 133.54 131.63 129.27 124.89 124.26 121.38
77.32 77.00 76.68
28.83 23.56 22.25 13.67
1l
f1 (ppm)

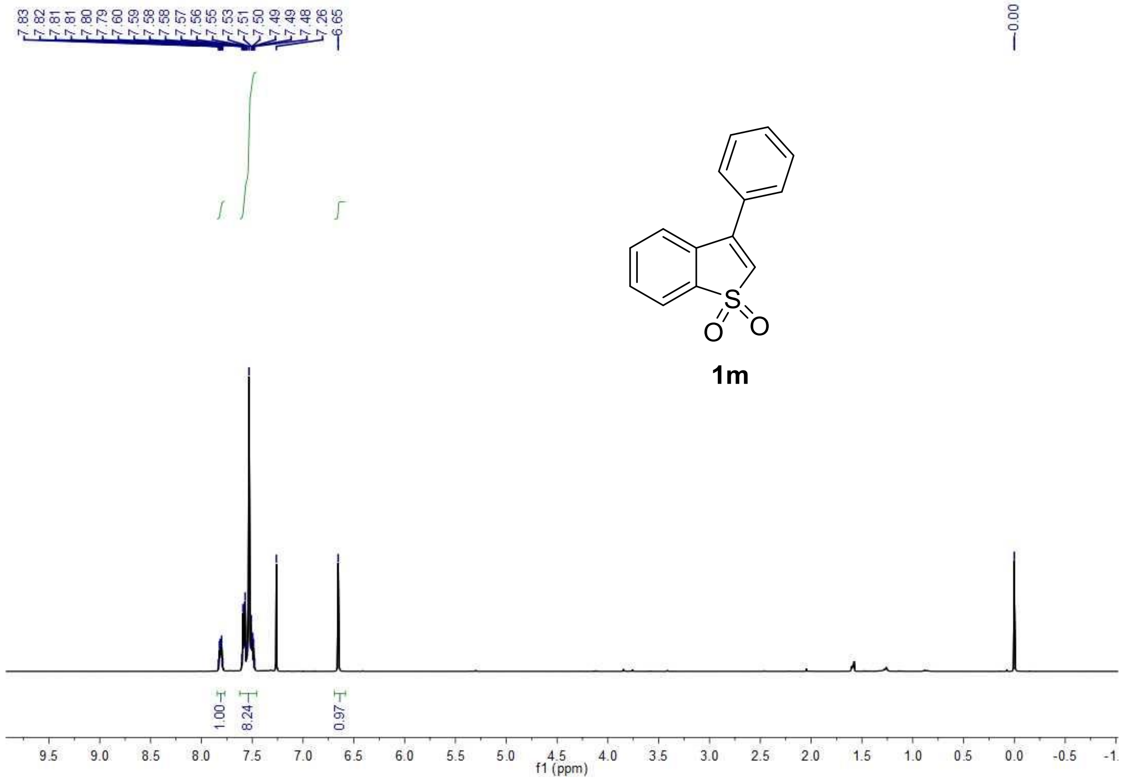
7.83 7.82 7.81 7.81 7.80 7.79 7.60 7.59 7.58 7.58 7.57 7.56 7.55 7.53 7.51 7.50 7.49 7.48 7.26 6.65
0.00
1m
1.00 8.24 0.97
f1 (ppm)

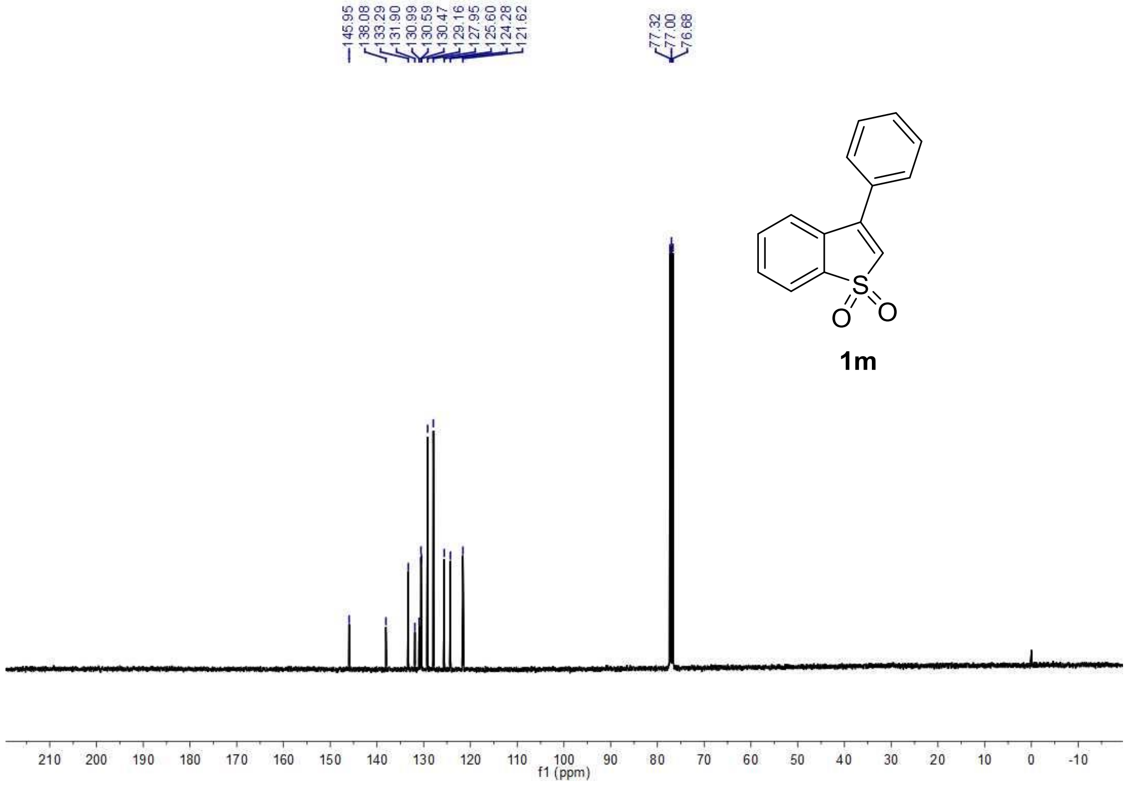
145.95 138.08 133.29 131.90 130.99 130.59 130.47 129.16 127.95 125.60 124.28 121.62
77.32 77.00 76.68
1m
f1 (ppm)

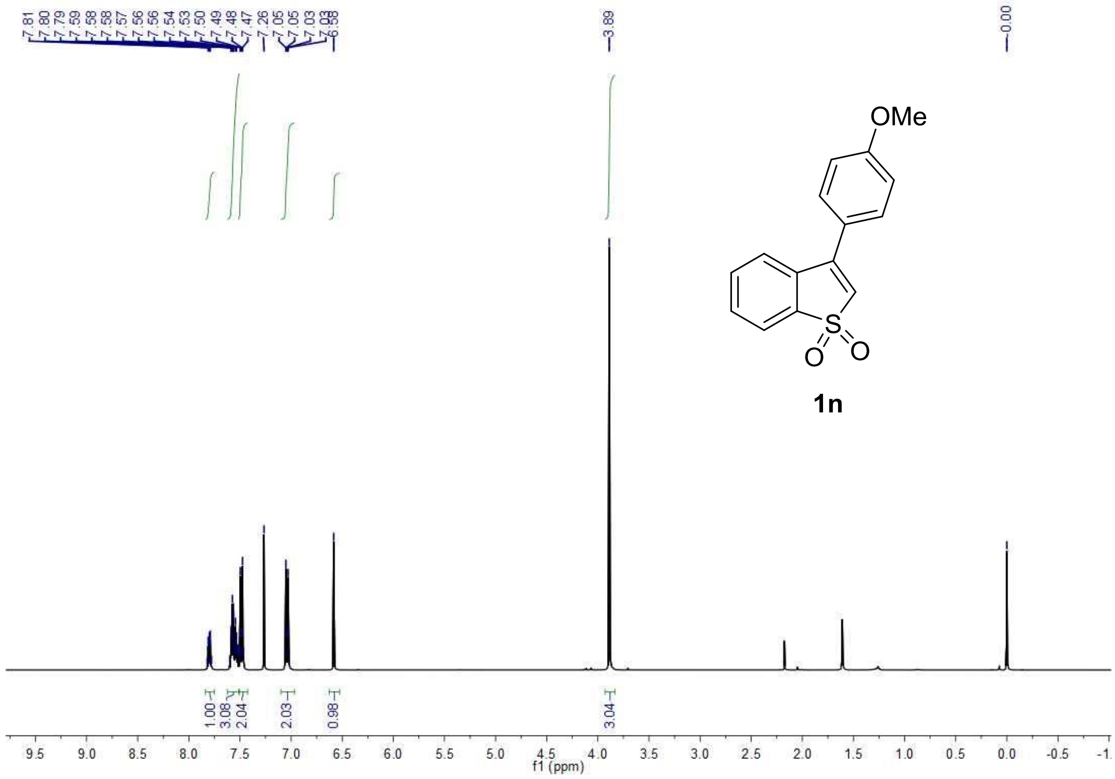
OMe
1n
f1 (ppm)

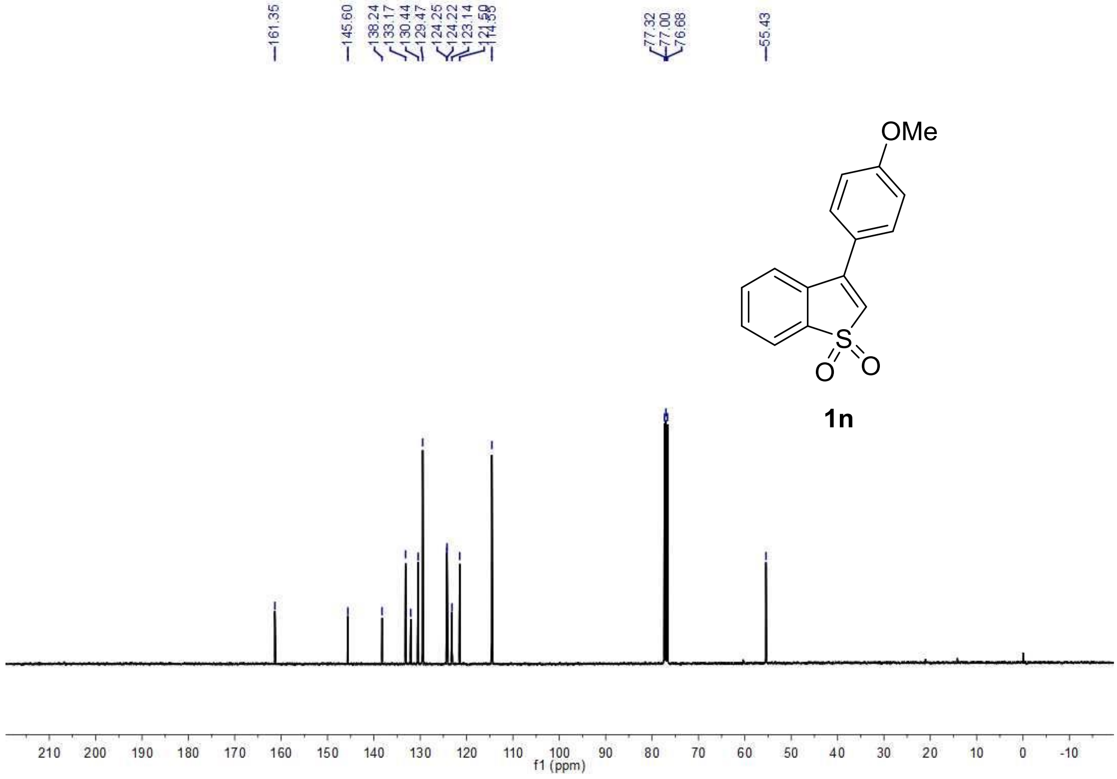
OMe
1n
f1 (ppm)

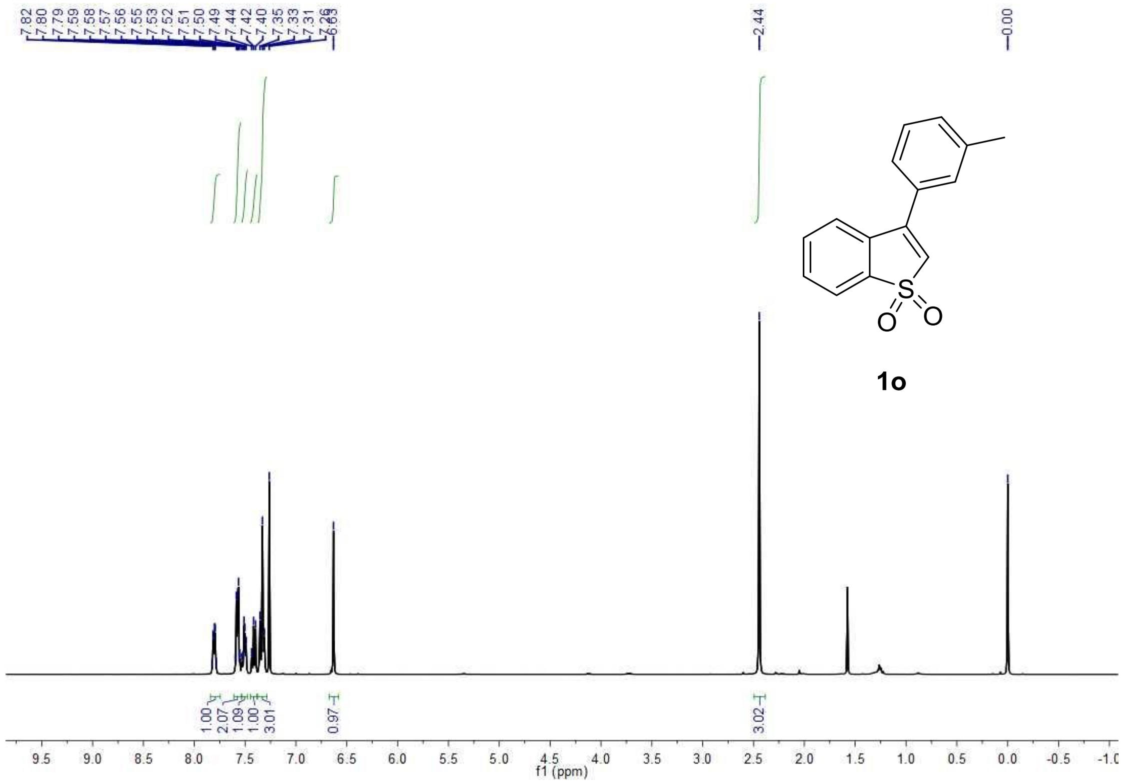
7.82 7.80 7.79 7.59 7.58 7.57 7.56 7.55 7.53 7.52 7.51 7.50 7.49 7.44 7.42 7.40 7.35 7.33 7.31 7.26 6.63
2.44
0.00
1o
1.00 2.07 1.09 1.00 3.01
0.97
3.02
9.5 9.0 8.5 8.0 7.5 7.0 6.5 6.0 5.5 5.0 4.5 4.0 3.5 3.0 2.5 2.0 1.5 1.0 0.5 0.0 -0.5 -1.0
f1 (ppm)

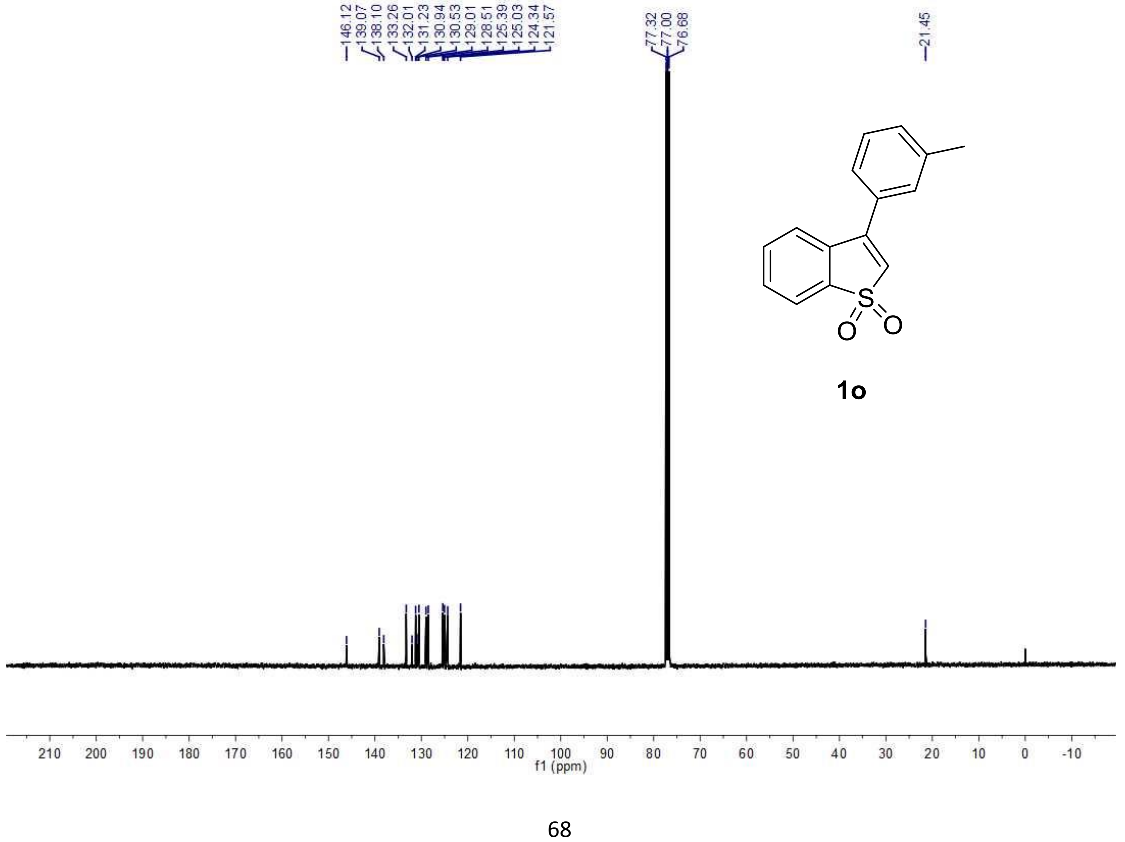
146.12 139.07 138.10 133.26 132.01 131.23 130.94 130.53 129.01 128.51 125.39 125.03 124.34 121.57
77.32 77.00 76.68
21.45
1o
210 200 190 180 170 160 150 140 130 120 110 100 90 80 70 60 50 40 30 20 10 0 -10
f1 (ppm)

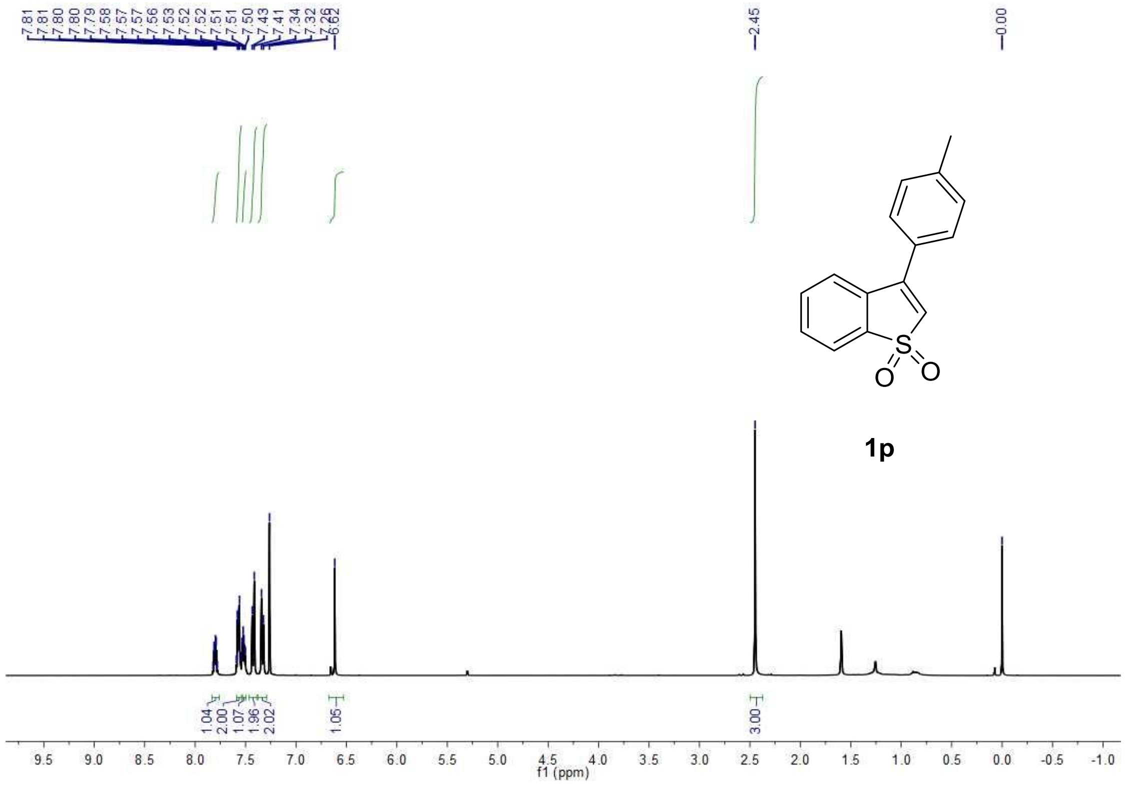
7.81
7.81
7.80
7.79
7.58
7.57
7.56
7.53
7.52
7.52
7.51
7.51
7.50
7.43
7.41
7.34
7.32
7.26
6.62
2.45
0.00
S
O
O
1.04
2.00
1.07
1.96
2.02
1.05
3.00
f1 (ppm)

1p

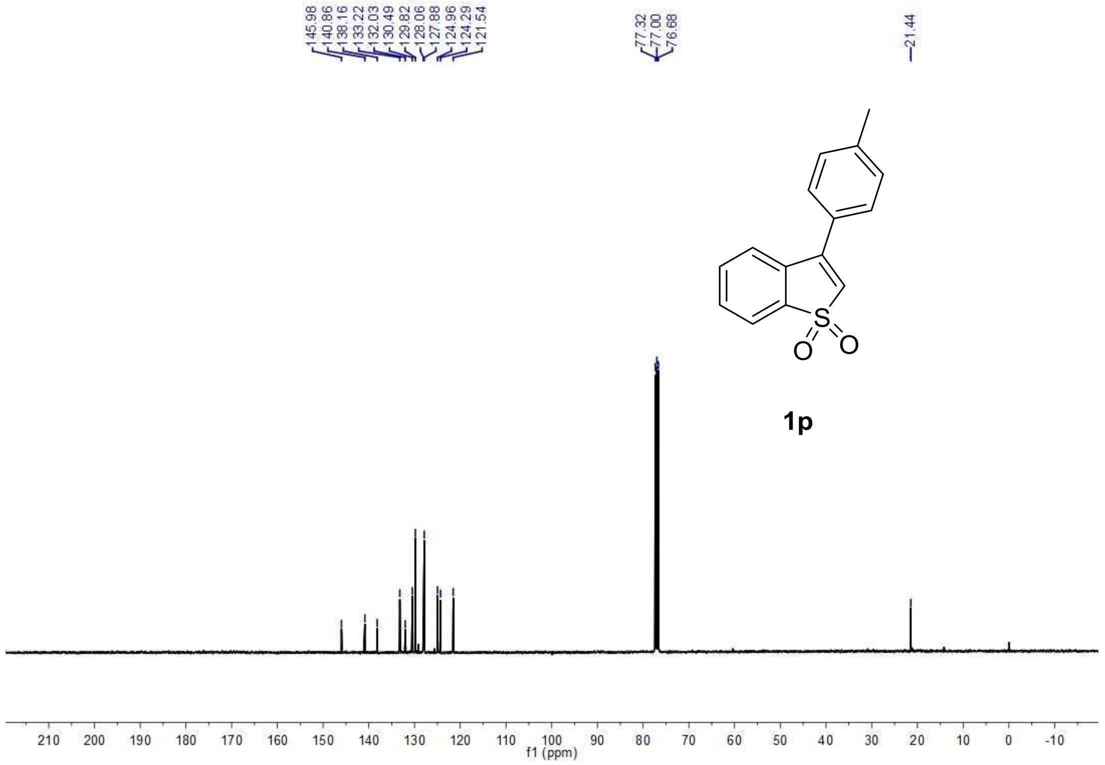
145.98
140.86
138.16
133.22
132.03
130.49
129.82
128.06
127.88
124.96
124.29
121.54
77.32
77.00
76.68
21.44
S
O
O
f1 (ppm)

1p

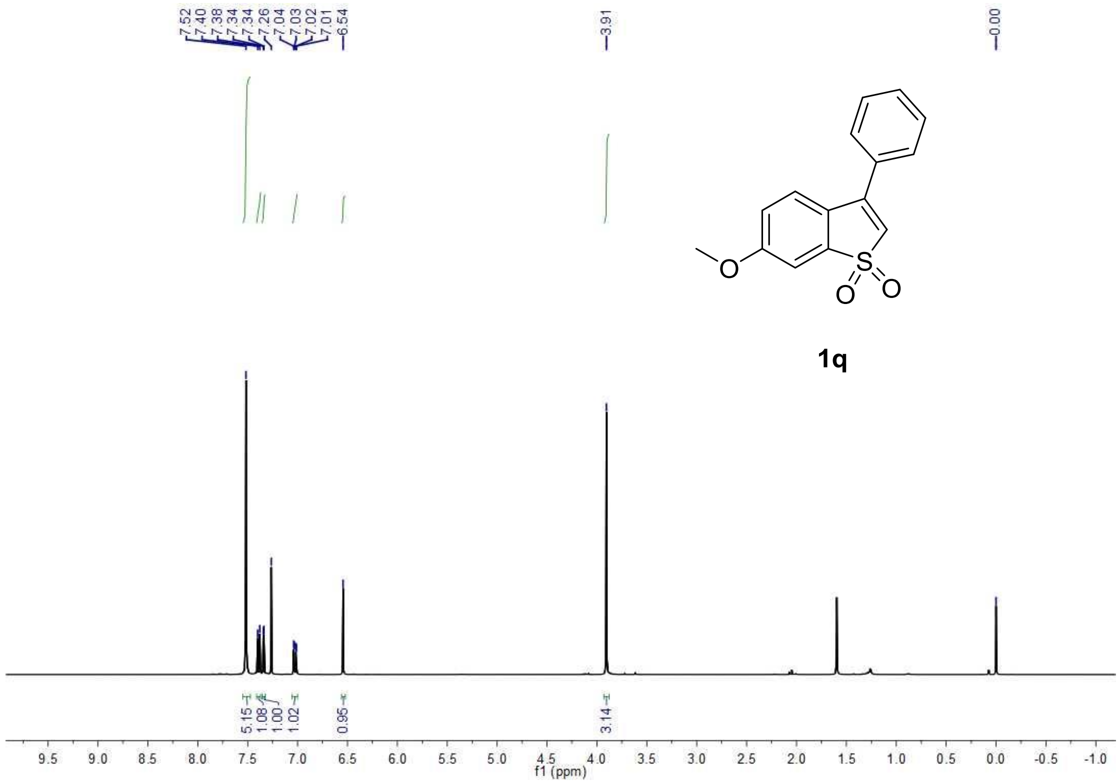
7.52
7.40
7.38
7.34
7.34
7.26
7.04
7.03
7.02
7.01
6.54
3.91
0.00
1q
5.15
1.08
1.00
1.02
0.95
3.14
9.5 9.0 8.5 8.0 7.5 7.0 6.5 6.0 5.5 5.0 4.5 4.0 3.5 3.0 2.5 2.0 1.5 1.0 0.5 0.0 -0.5 -1.0
f1 (ppm)

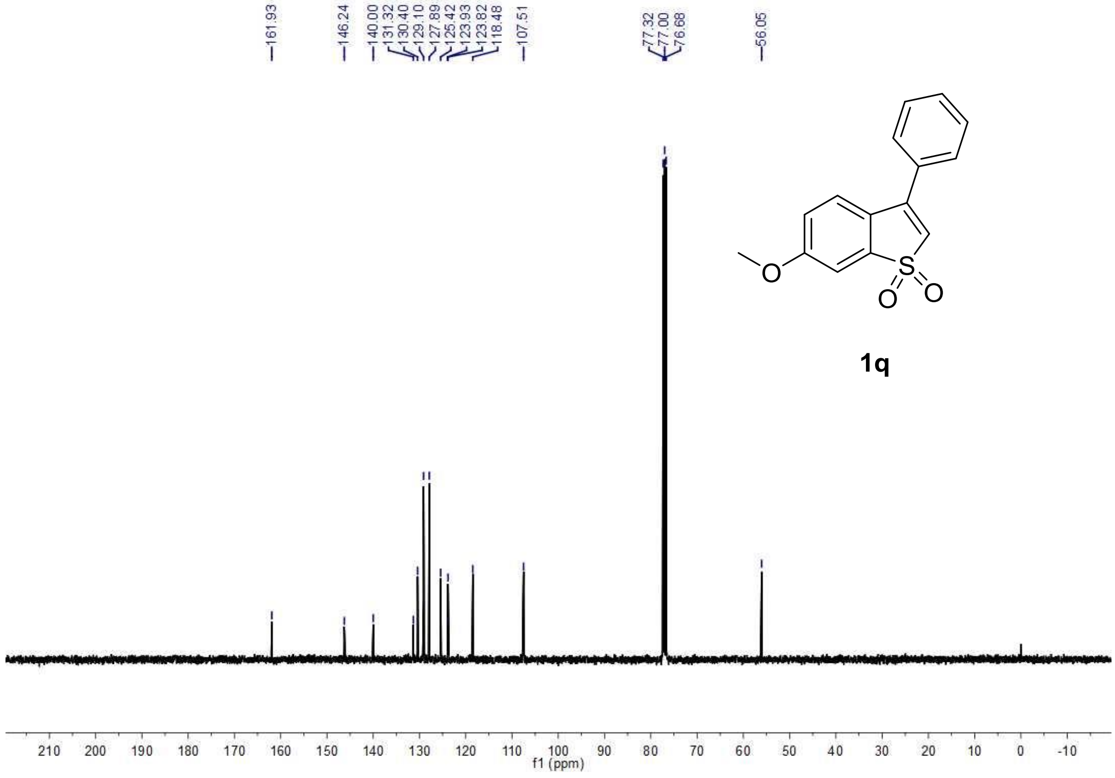
161.93
146.24
140.00
131.32
130.40
129.10
127.89
125.42
123.93
123.82
118.48
107.51
77.32
77.00
76.68
56.05
1q
210 200 190 180 170 160 150 140 130 120 110 100 90 80 70 60 50 40 30 20 10 0 -10
f1 (ppm)

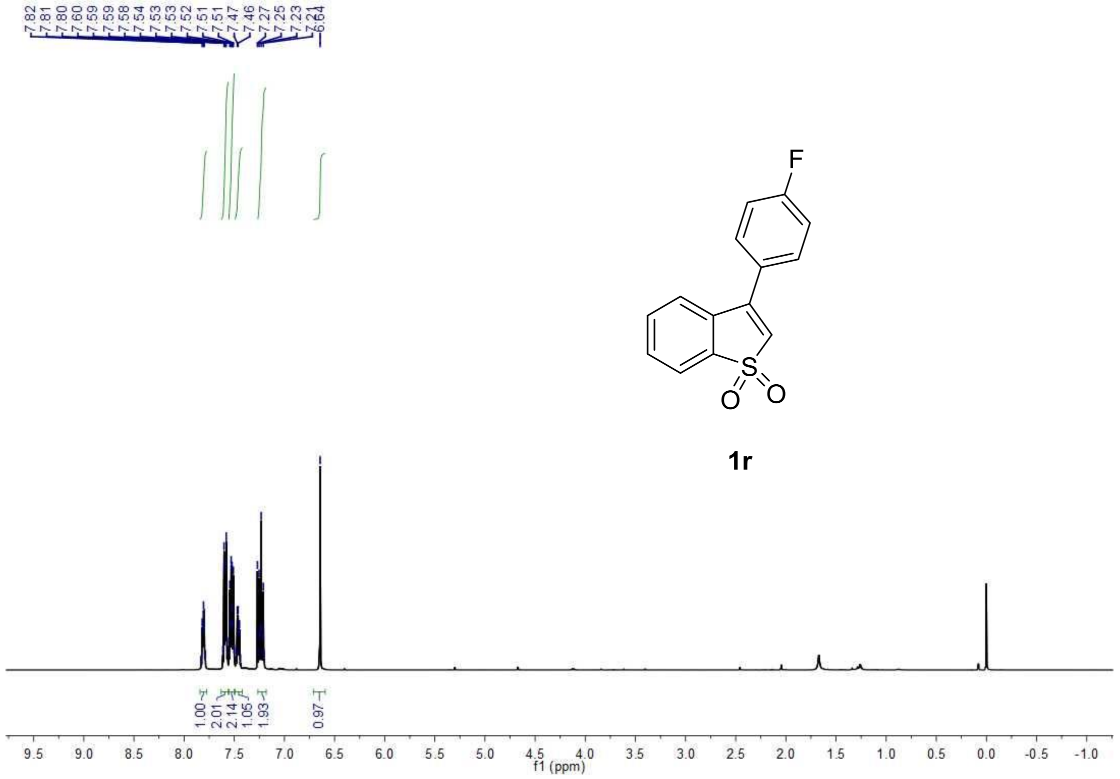
7.82 7.81 7.80 7.60 7.59 7.59 7.58 7.54 7.53 7.53 7.52 7.51 7.51 7.47 7.46 7.27 7.25 7.23 7.21 6.64
F
S
O
O
1r
1.00 2.01 2.14 1.05 1.93 0.97
f1 (ppm)

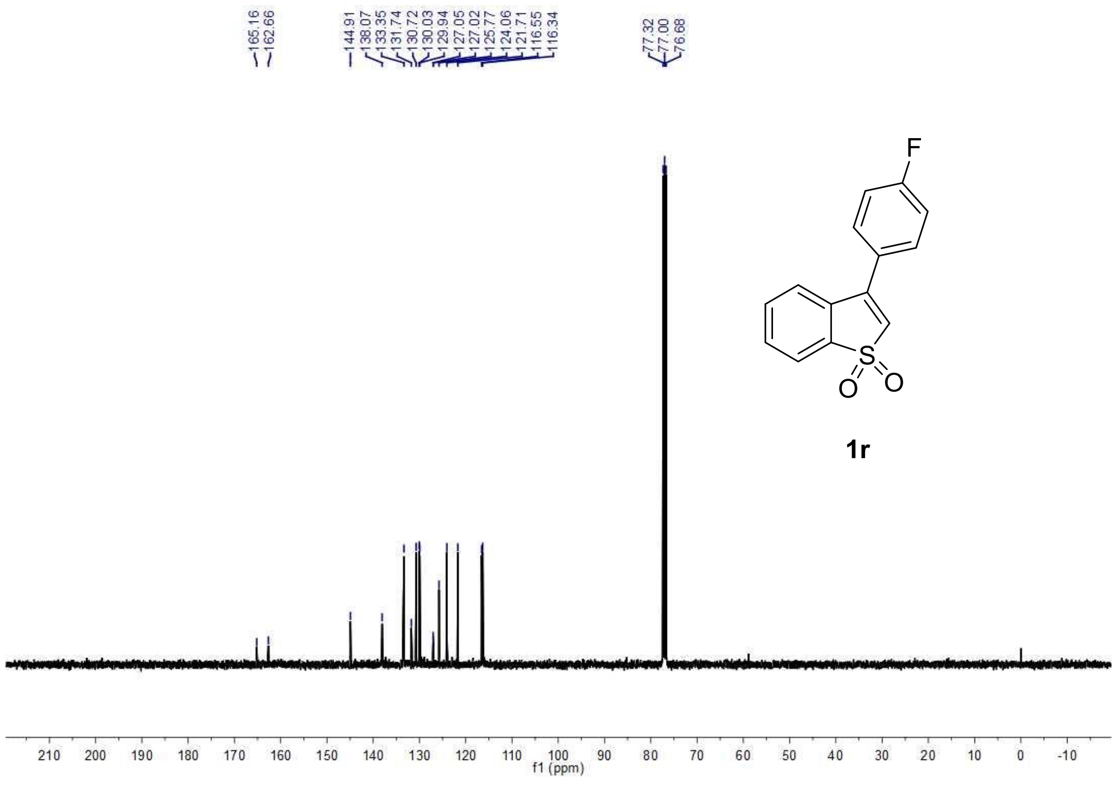
165.16 162.66
144.91 138.07 133.35 131.74 130.72 130.03 129.94 127.05 127.02 125.77 124.06 121.71 116.55 116.34
77.32 77.00 76.68
F
S
O
O
1r
f1 (ppm)

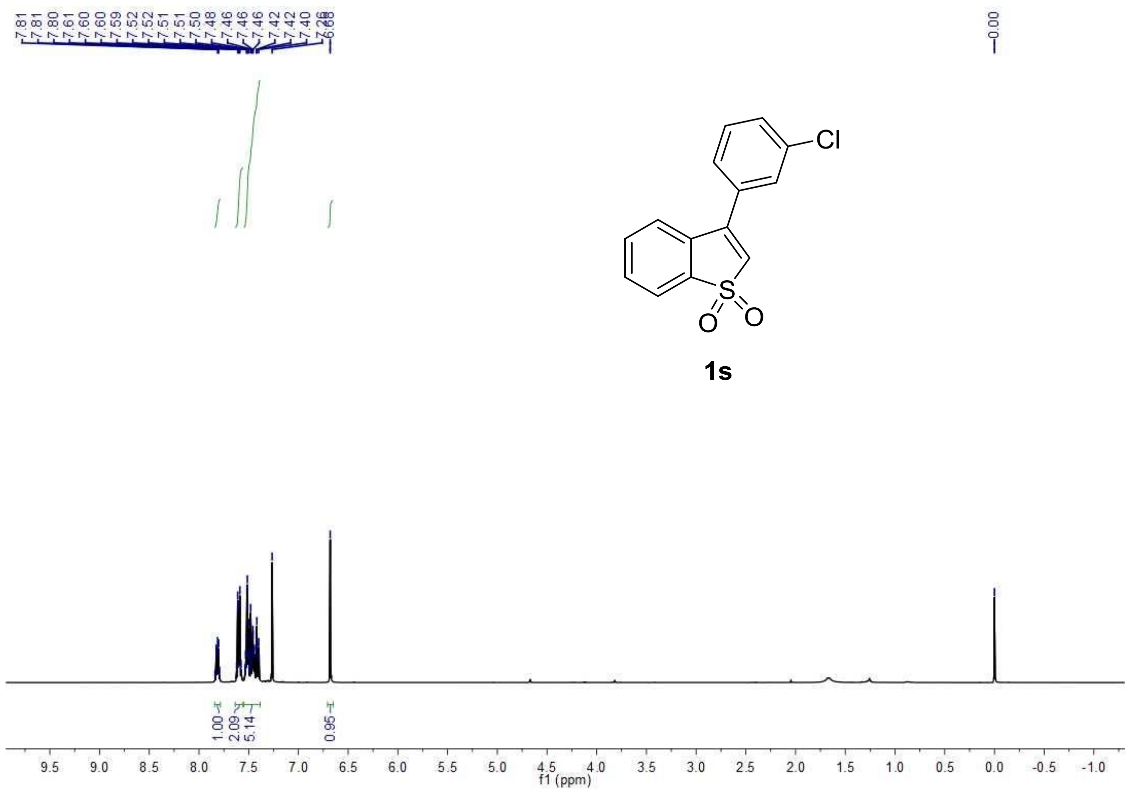
7.81
7.81
7.80
7.61
7.60
7.59
7.52
7.52
7.51
7.51
7.50
7.48
7.46
7.46
7.46
7.42
7.42
7.40
7.26
6.68
0.00
Cl
S
O
O
1s
1.00
2.09
5.14
0.95
f1 (ppm)

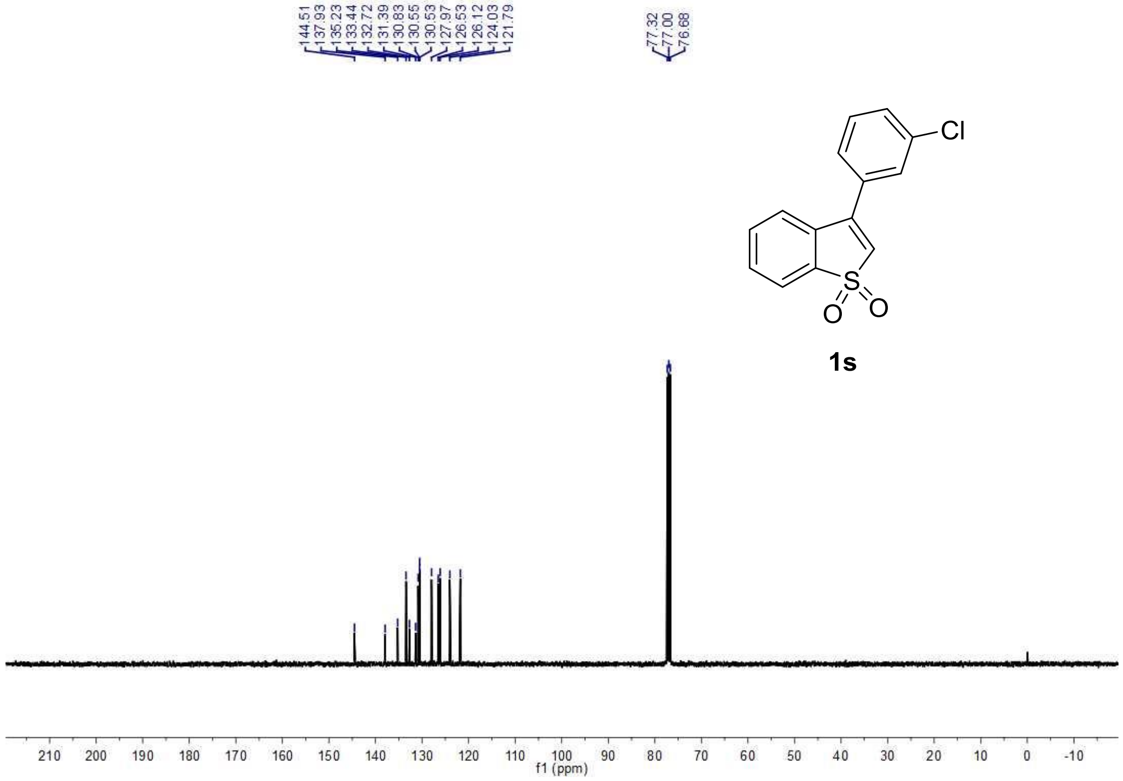
144.51
137.93
135.23
133.44
132.72
131.39
130.83
130.55
130.53
127.97
126.53
126.12
124.03
121.79
77.32
77.00
76.68
Cl
S
O
O
1s
f1 (ppm)

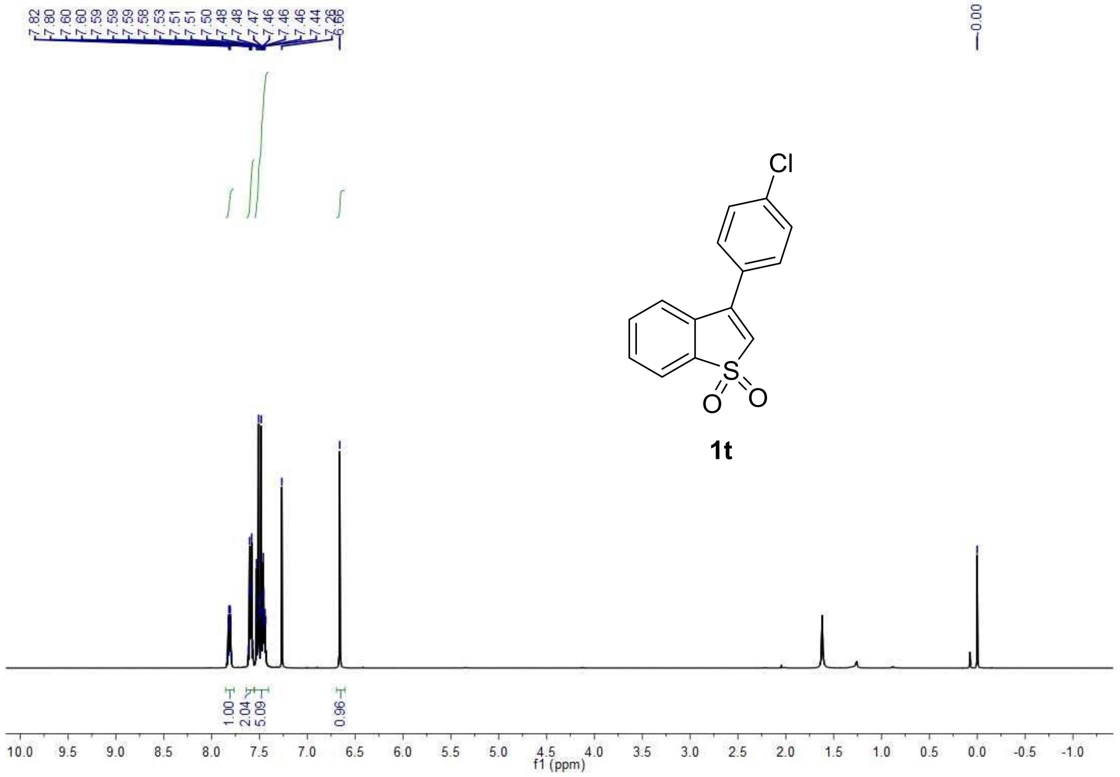
7.82
7.80
7.60
7.60
7.59
7.59
7.59
7.58
7.53
7.51
7.51
7.50
7.48
7.48
7.47
7.46
7.46
7.46
7.44
7.26
6.68
0.00
Cl
S
O
O
1t
1.00
2.04
5.09
0.96
10.0 9.5 9.0 8.5 8.0 7.5 7.0 6.5 6.0 5.5 5.0 4.5 4.0 3.5 3.0 2.5 2.0 1.5 1.0 0.5 0.0 -0.5 -1.0
f1 (ppm)

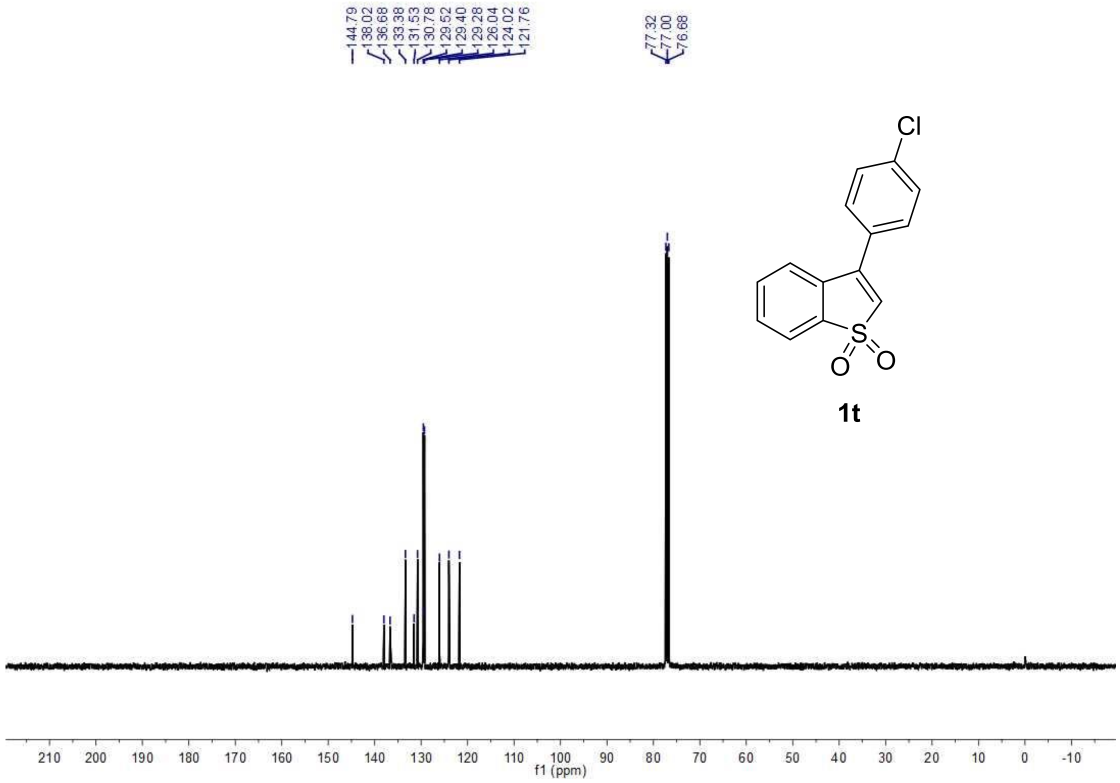
144.79
138.02
136.68
133.38
131.53
130.78
129.52
129.40
129.28
126.04
124.02
121.76
77.32
77.00
76.68
Cl
S
O
O
1t
210 200 190 180 170 160 150 140 130 120 110 100 90 80 70 60 50 40 30 20 10 0 -10
f1 (ppm)

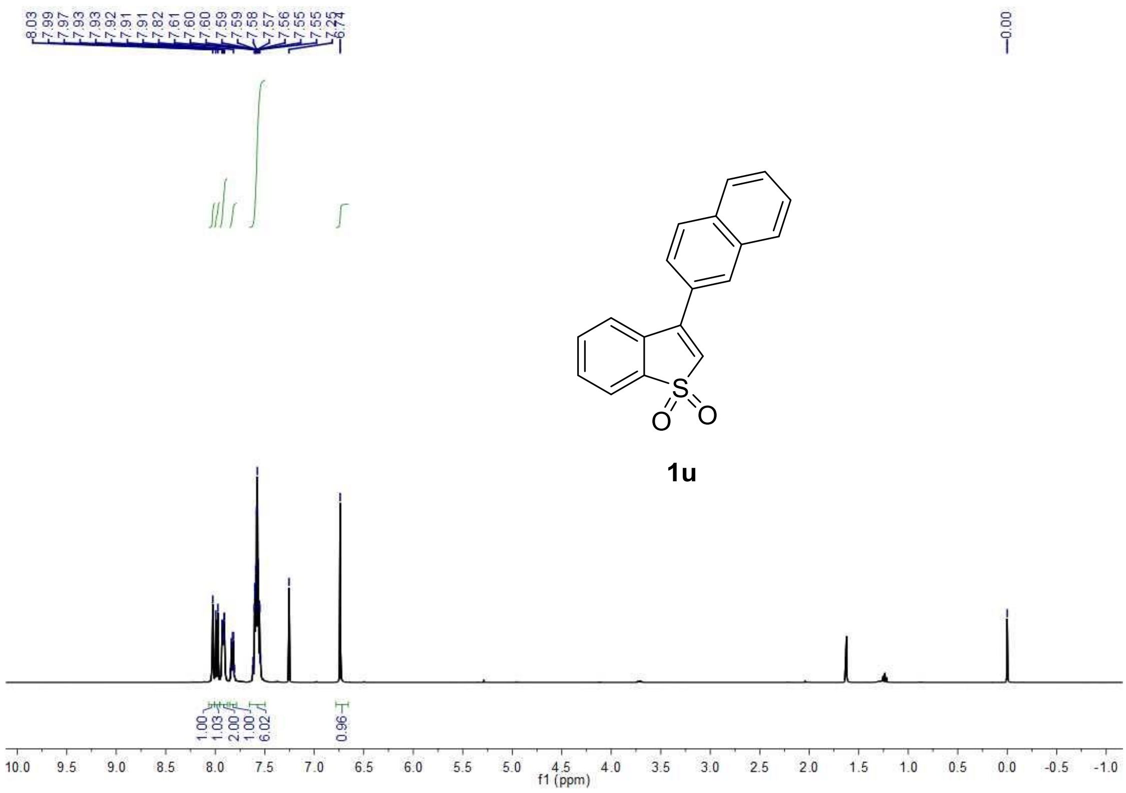
8.03 7.99 7.97 7.93 7.93 7.92 7.91 7.82 7.61 7.60 7.59 7.59 7.58 7.57 7.56 7.55 7.55 7.25 6.74
0.00
1.00 1.03 2.00 1.00 6.02
0.96
f1 (ppm)

1u

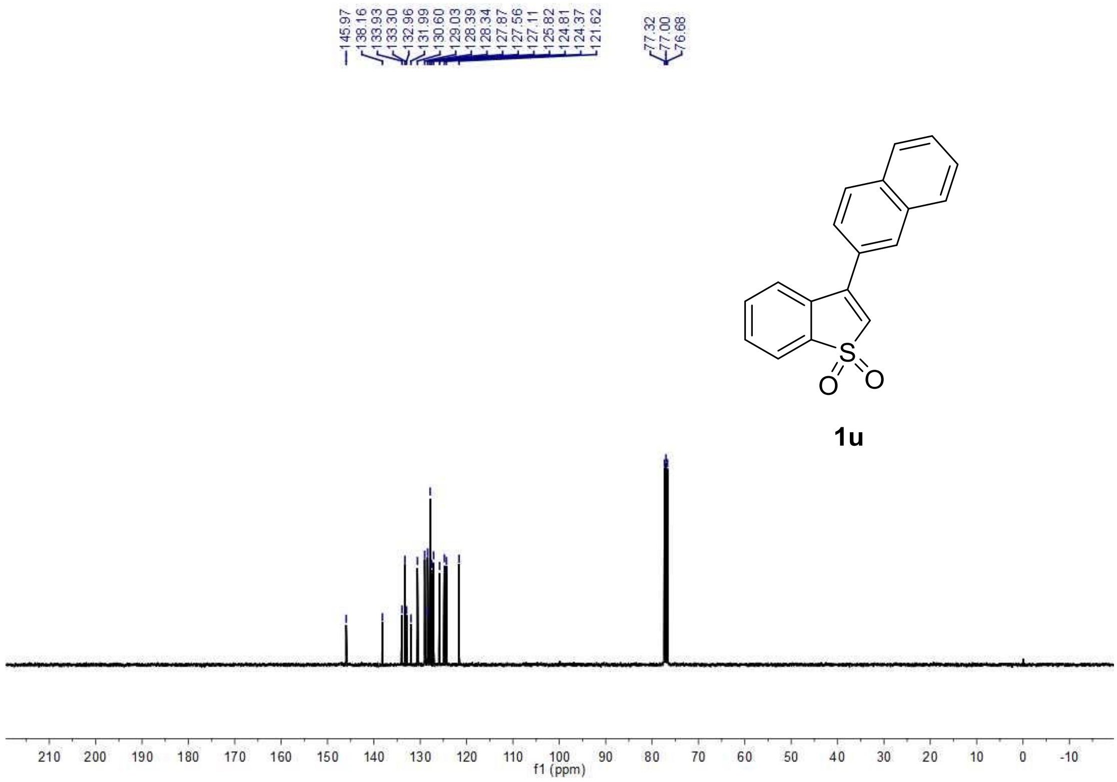
145.97 138.16 133.93 133.30 132.96 131.99 130.60 129.03 128.39 128.34 127.87 127.56 127.11 125.82 124.81 124.37 121.62
77.32 77.00 76.68
f1 (ppm)

1u

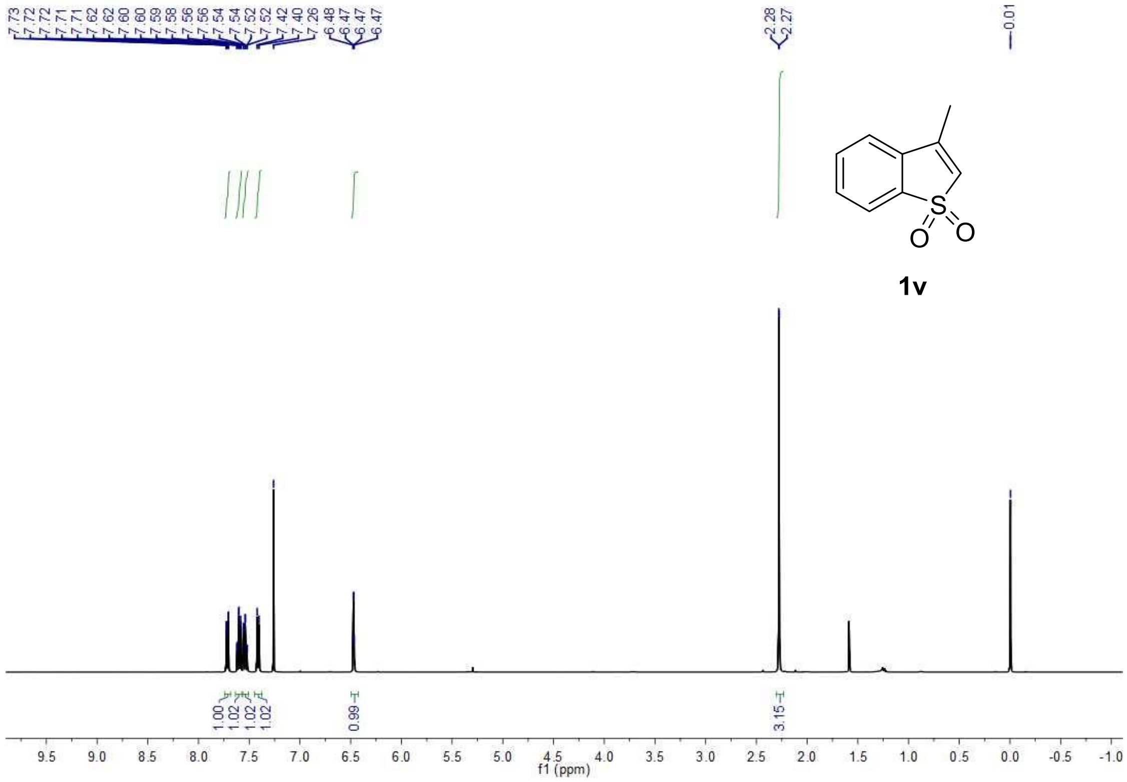

1v

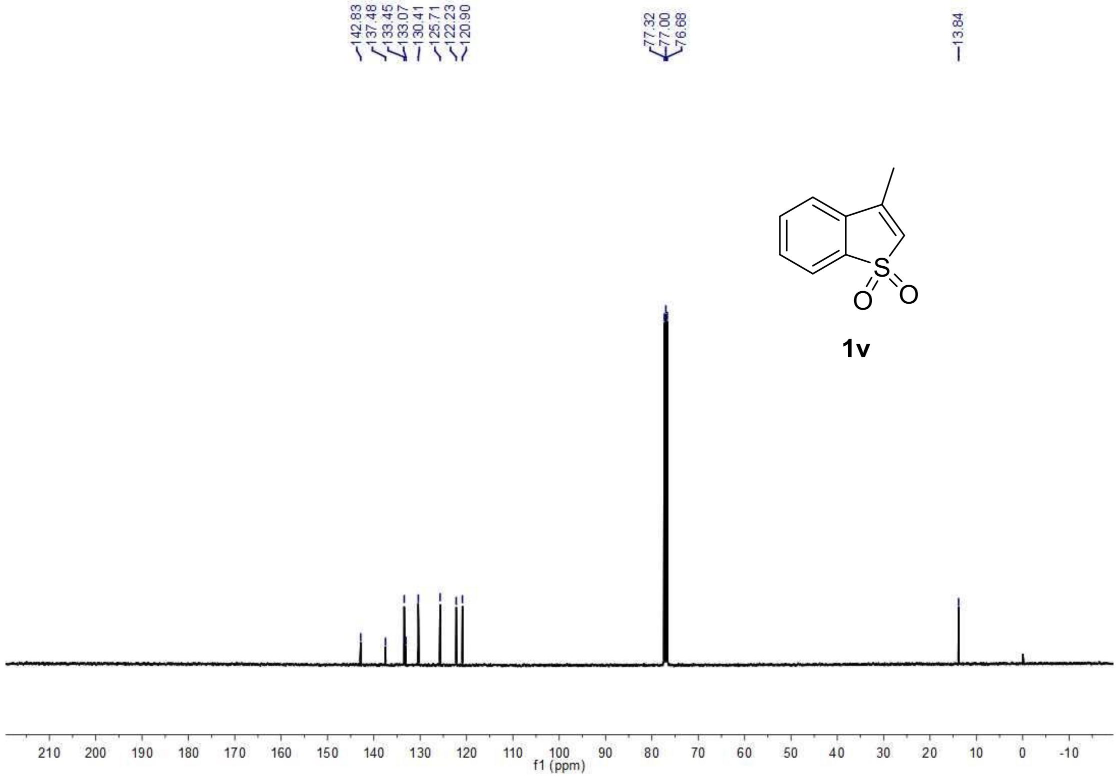

1v

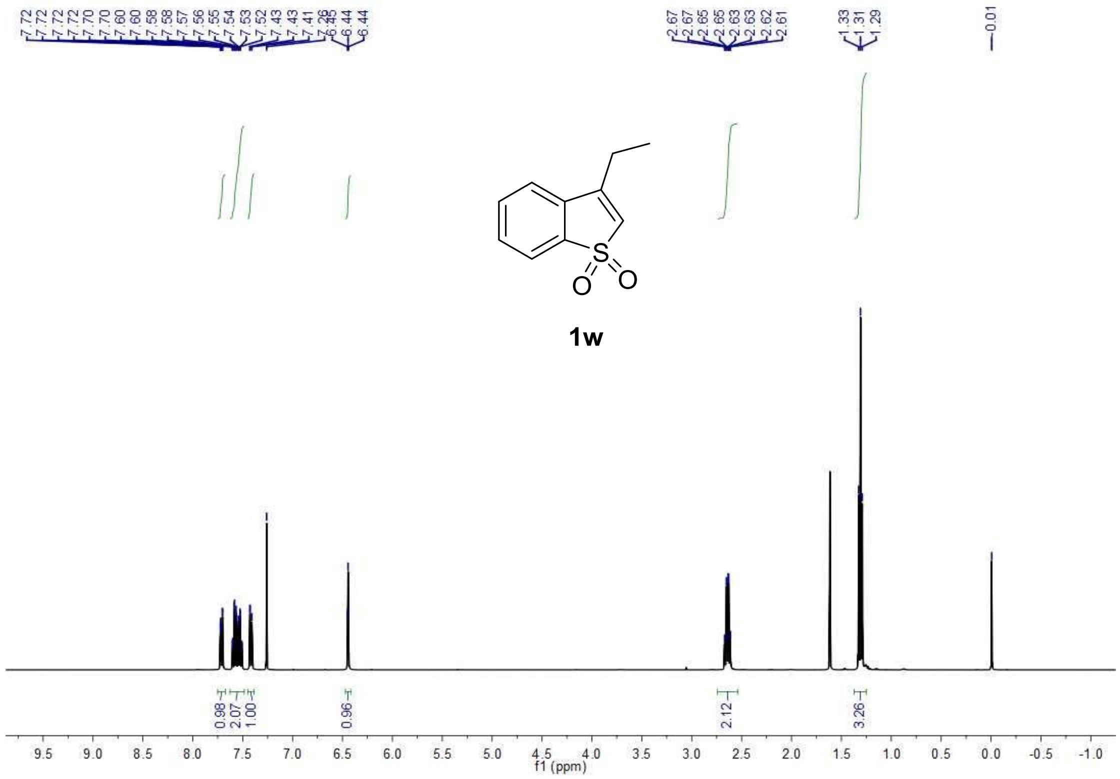

1w

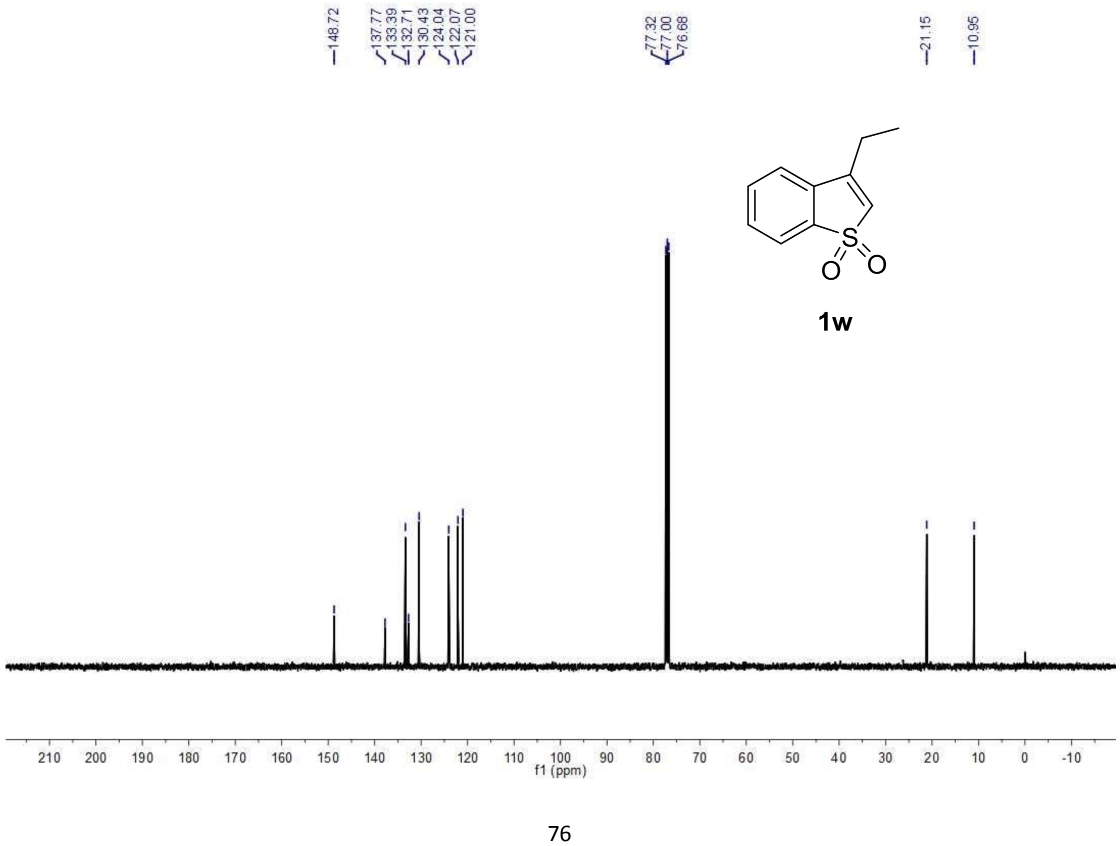

1w

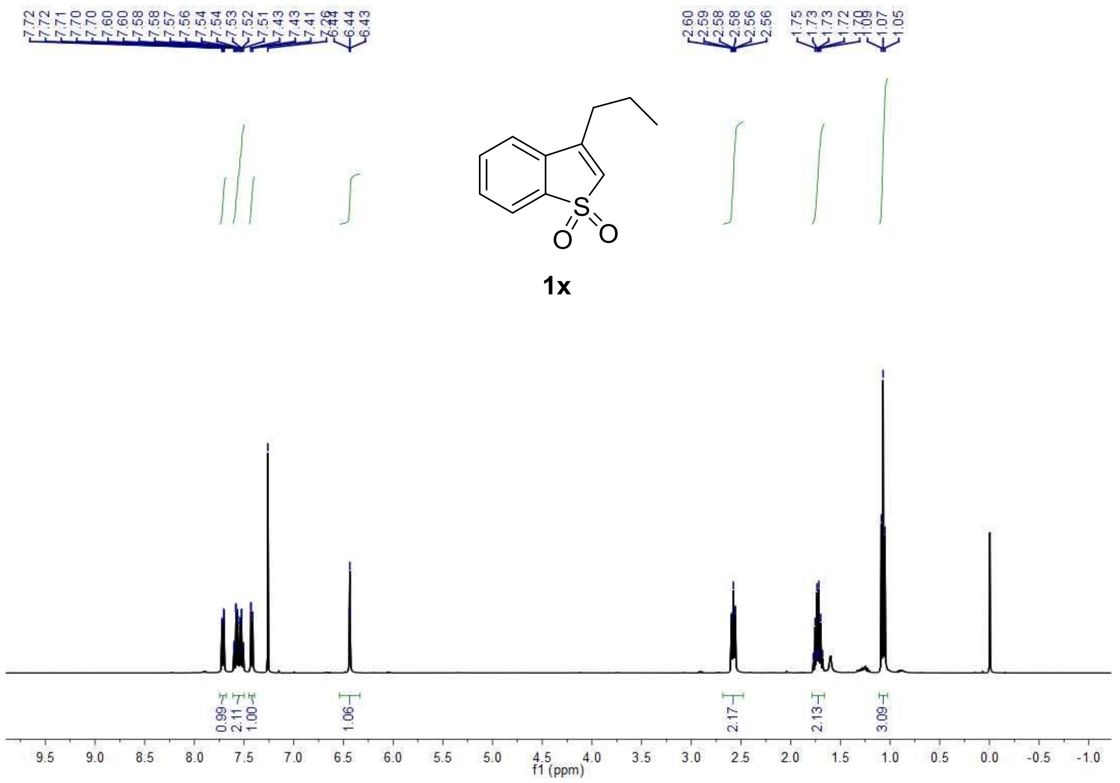

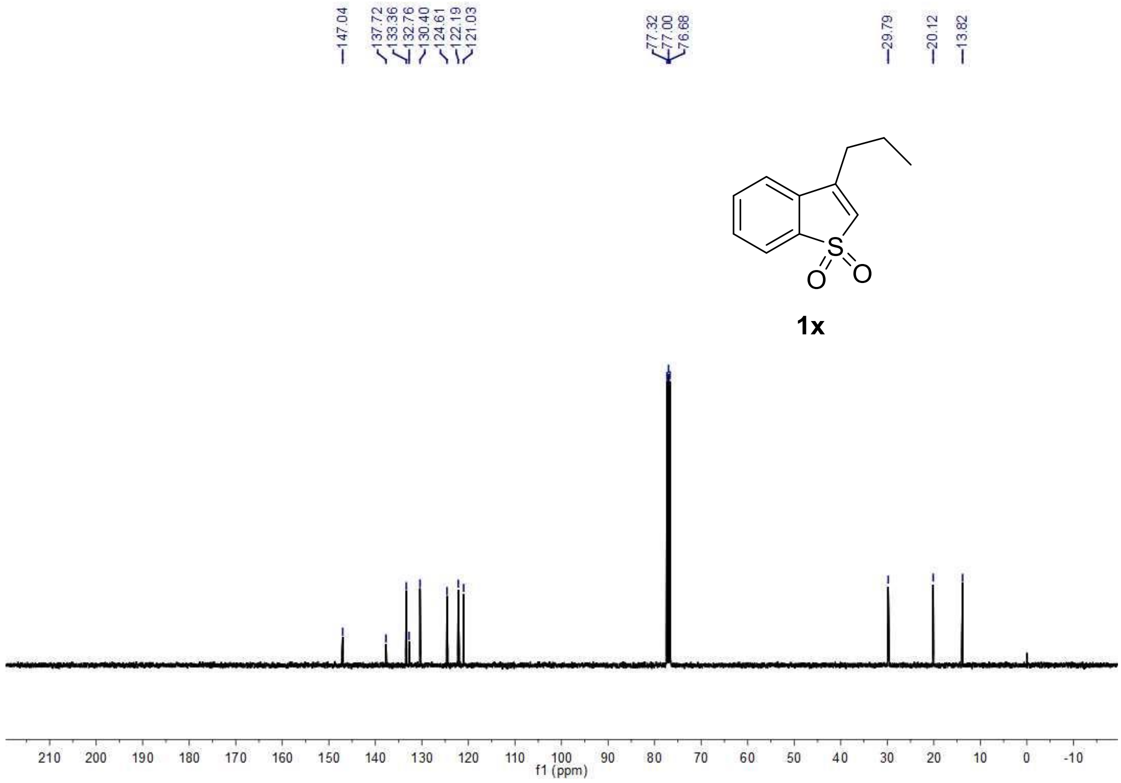

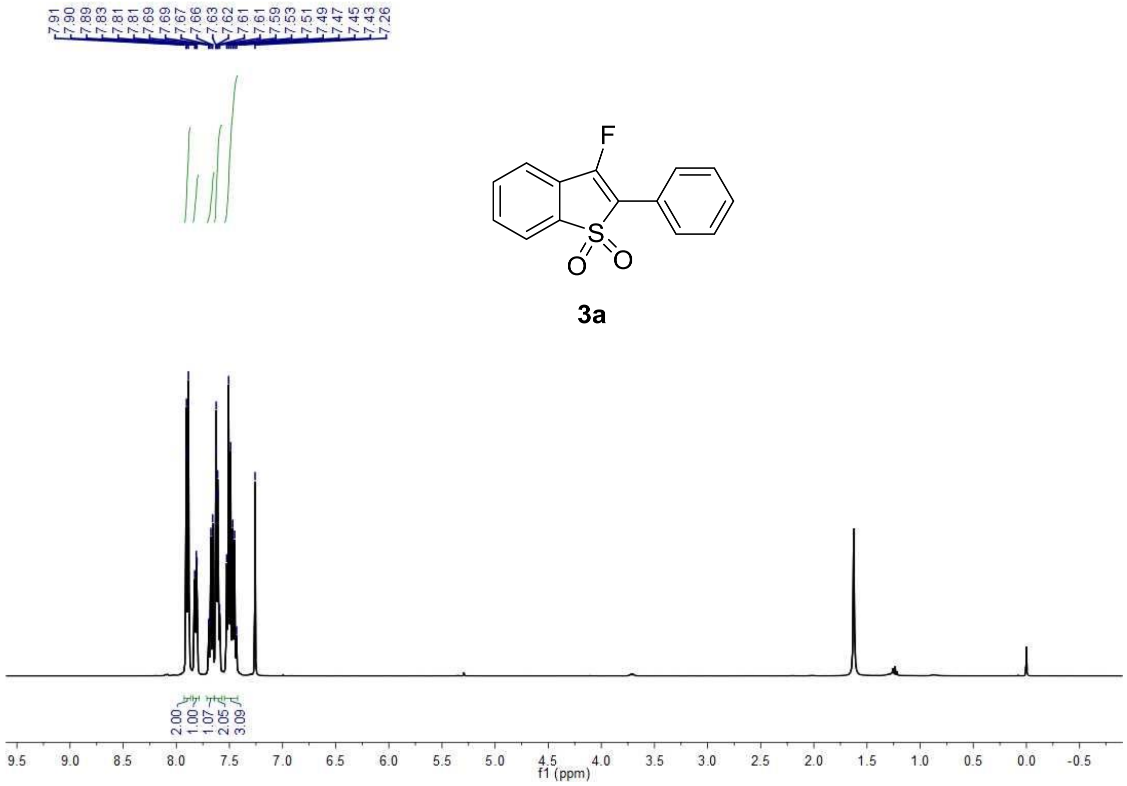
7.91 7.90 7.89 7.83 7.81 7.81 7.69 7.67 7.66 7.63 7.62 7.61 7.59 7.53 7.51 7.49 7.47 7.45 7.43 7.26
F
S
O O
3a
2.00 1.00 1.07 2.05 3.09
f1 (ppm)

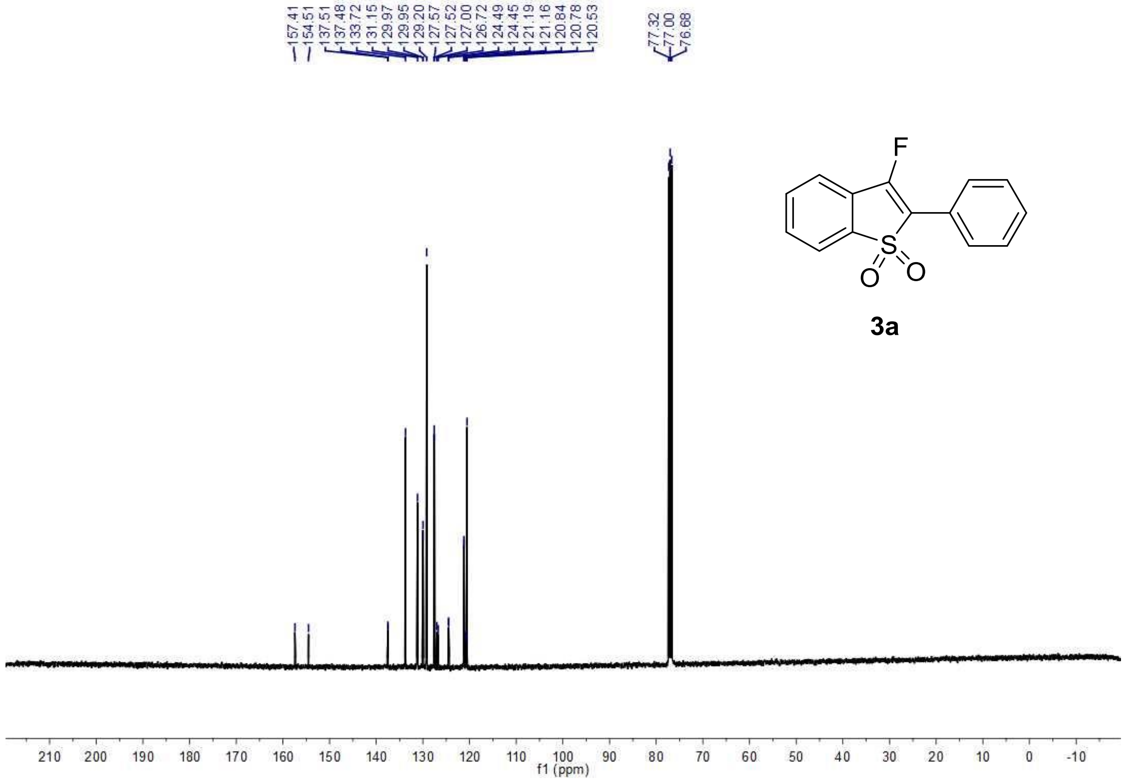
157.41 154.51 137.51 137.48 133.72 131.15 129.97 129.95 129.20 127.57 127.52 127.00 126.72 124.49 124.45 121.19 121.16 120.84 120.78 120.53
77.32 77.00 76.68
F
S
O O
3a
f1 (ppm)

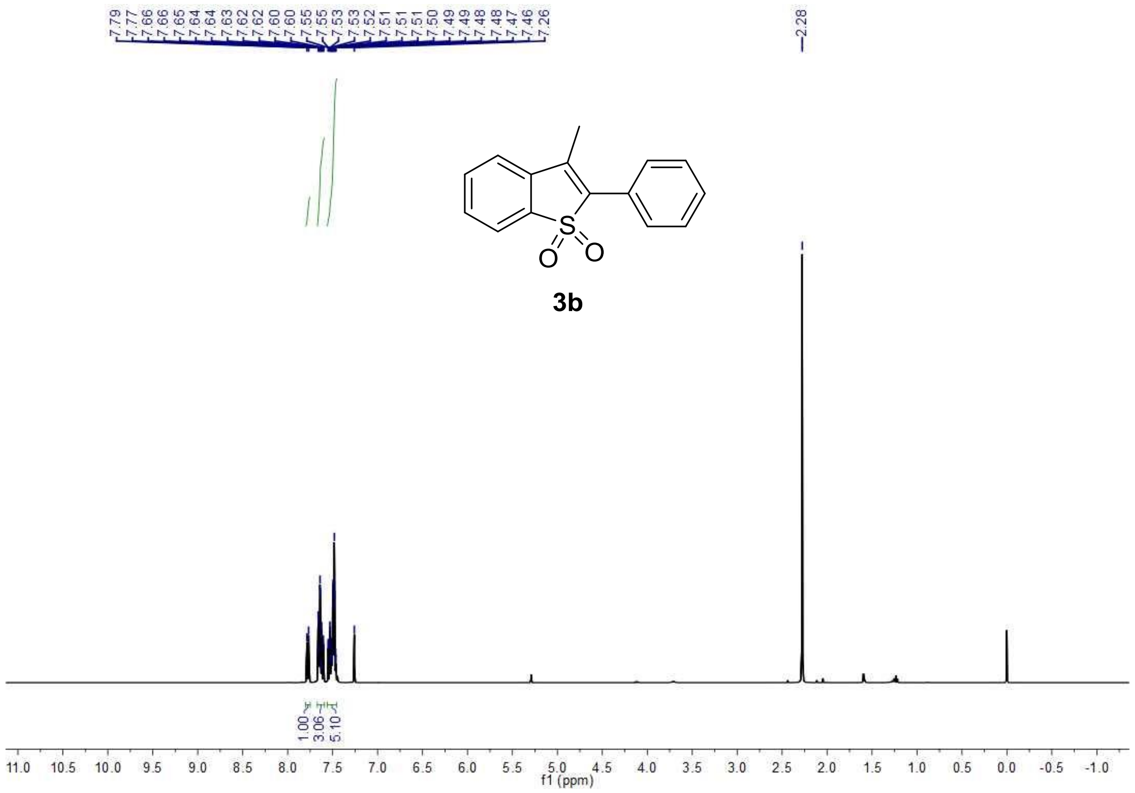

7.79
7.77
7.66
7.65
7.64
7.64
7.63
7.62
7.62
7.60
7.60
7.55
7.55
7.53
7.53
7.52
7.51
7.51
7.50
7.49
7.49
7.48
7.48
7.47
7.46
7.26
2.28
3b
1.00
3.06
5.10
f1 (ppm)

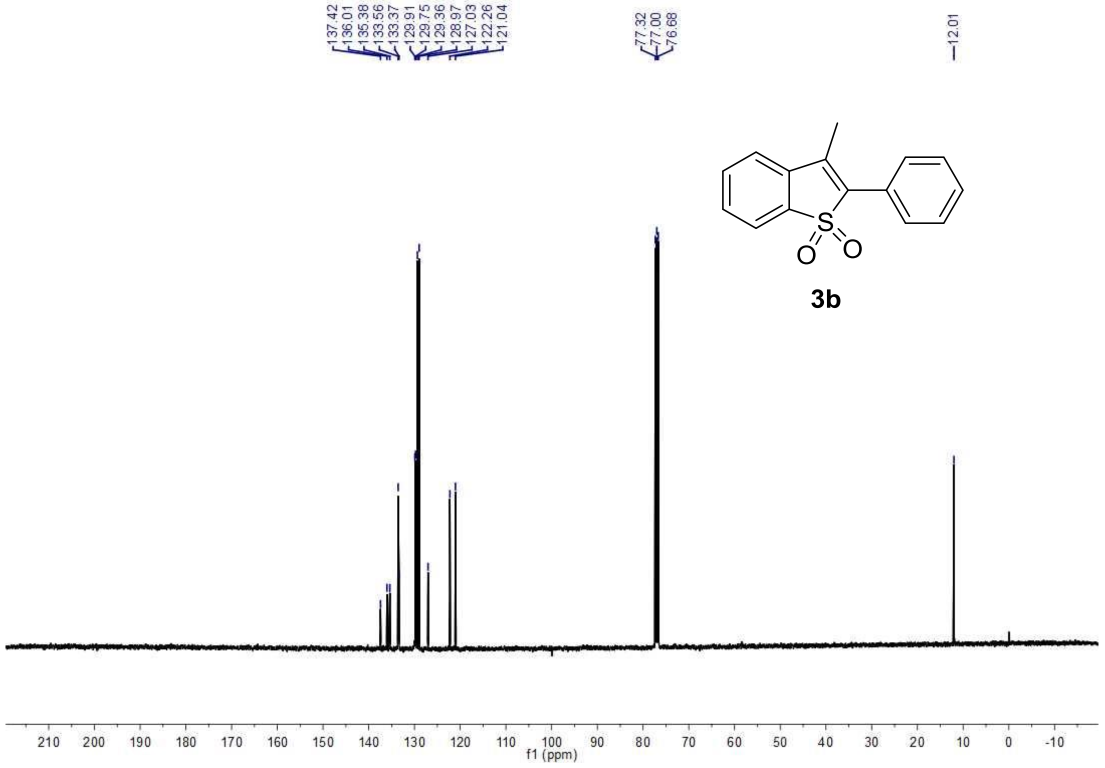

137.42
136.01
135.38
133.56
133.37
129.91
129.75
129.36
128.97
127.03
122.26
121.04
77.32
77.00
76.68
12.01
3b
f1 (ppm)

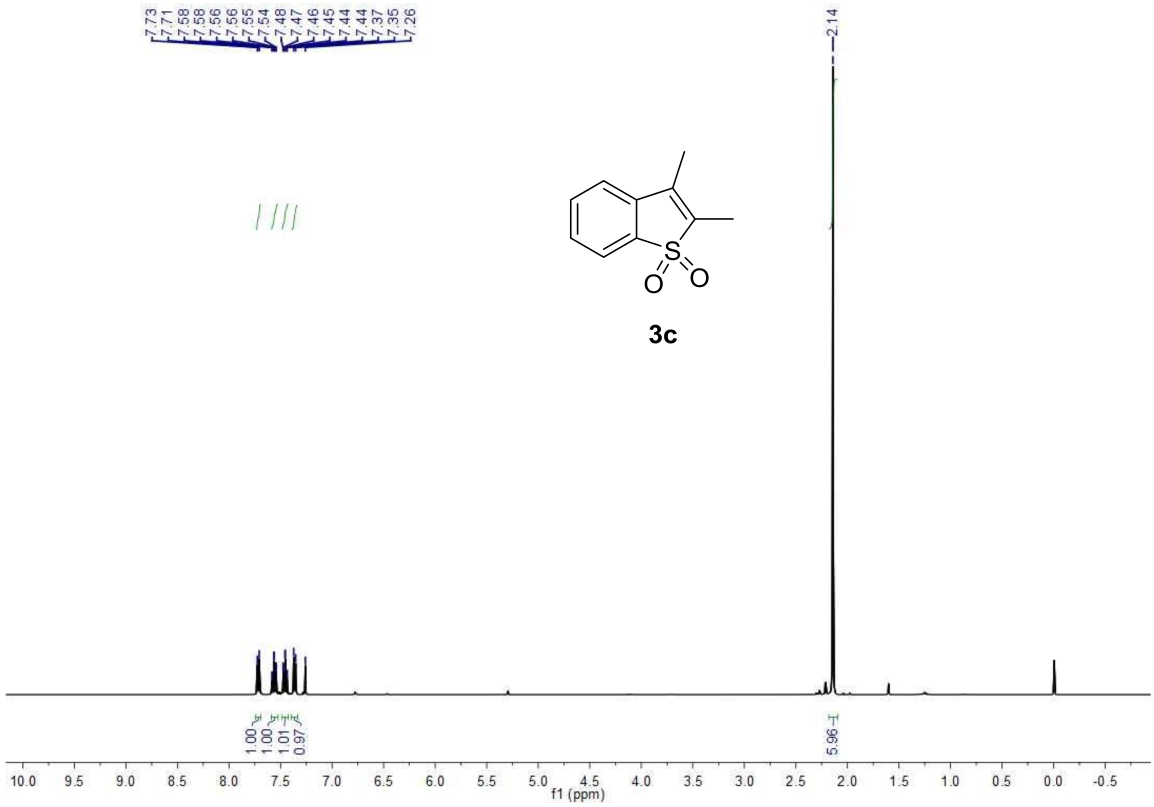
7.73 7.71 7.58 7.58 7.56 7.56 7.55 7.54 7.48 7.47 7.46 7.45 7.44 7.44 7.37 7.35 7.26
2.14
3c
1.00 1.00 1.01 0.97
5.96
10.0 9.5 9.0 8.5 8.0 7.5 7.0 6.5 6.0 5.5 5.0 4.5 4.0 3.5 3.0 2.5 2.0 1.5 1.0 0.5 0.0 -0.5
f1 (ppm)

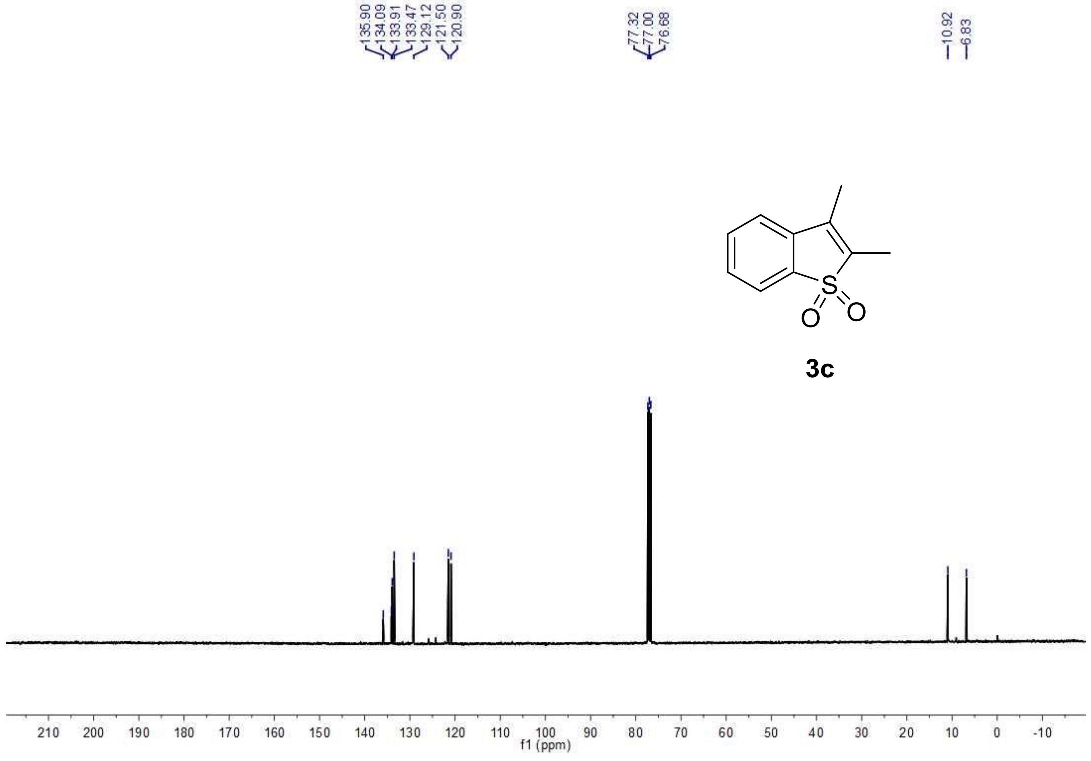
135.90 134.09 133.91 133.47 129.12 121.50 120.90
77.32 77.00 76.68
10.92
6.83
3c
210 200 190 180 170 160 150 140 130 120 110 100 90 80 70 60 50 40 30 20 10 0 -10
f1 (ppm)

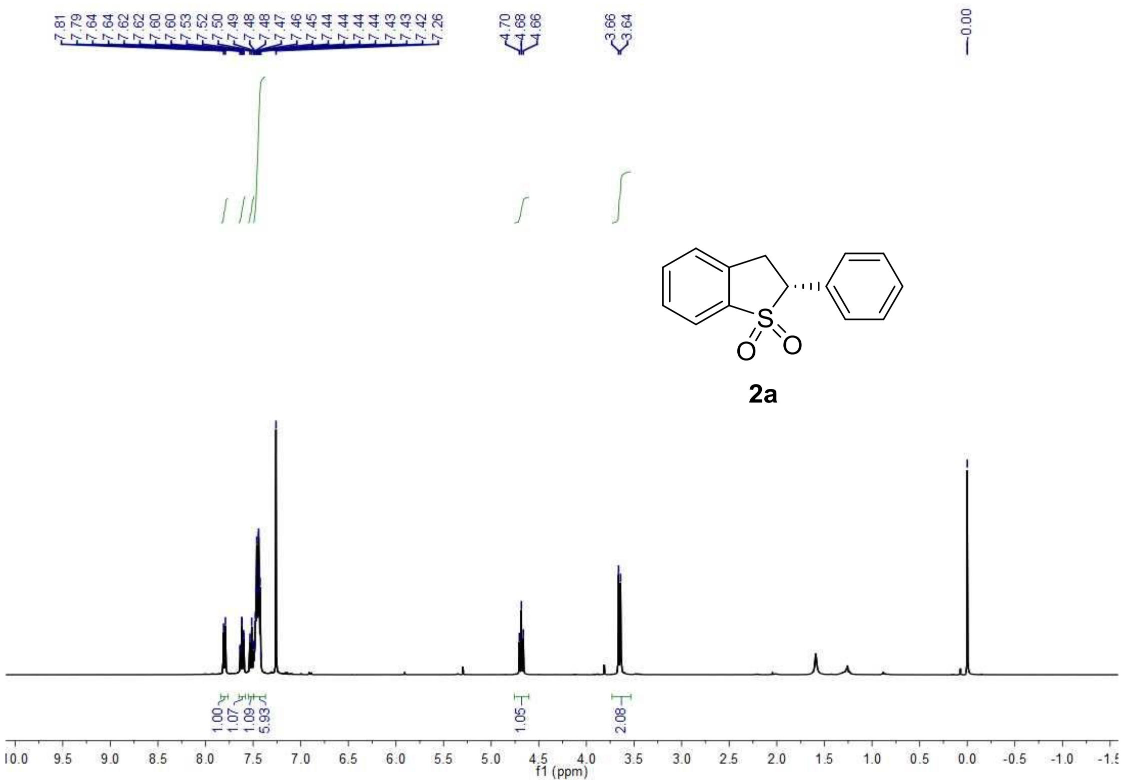

7.81 7.79 7.64 7.64 7.62 7.62 7.60 7.53 7.52 7.50 7.49 7.48 7.48 7.47 7.46 7.45 7.44 7.44 7.44 7.43 7.43 7.42 7.26
4.70 4.68 4.66
3.66 3.64
0.00
1.00 1.07 1.09 5.93
1.05
2.08
f1 (ppm)

2a

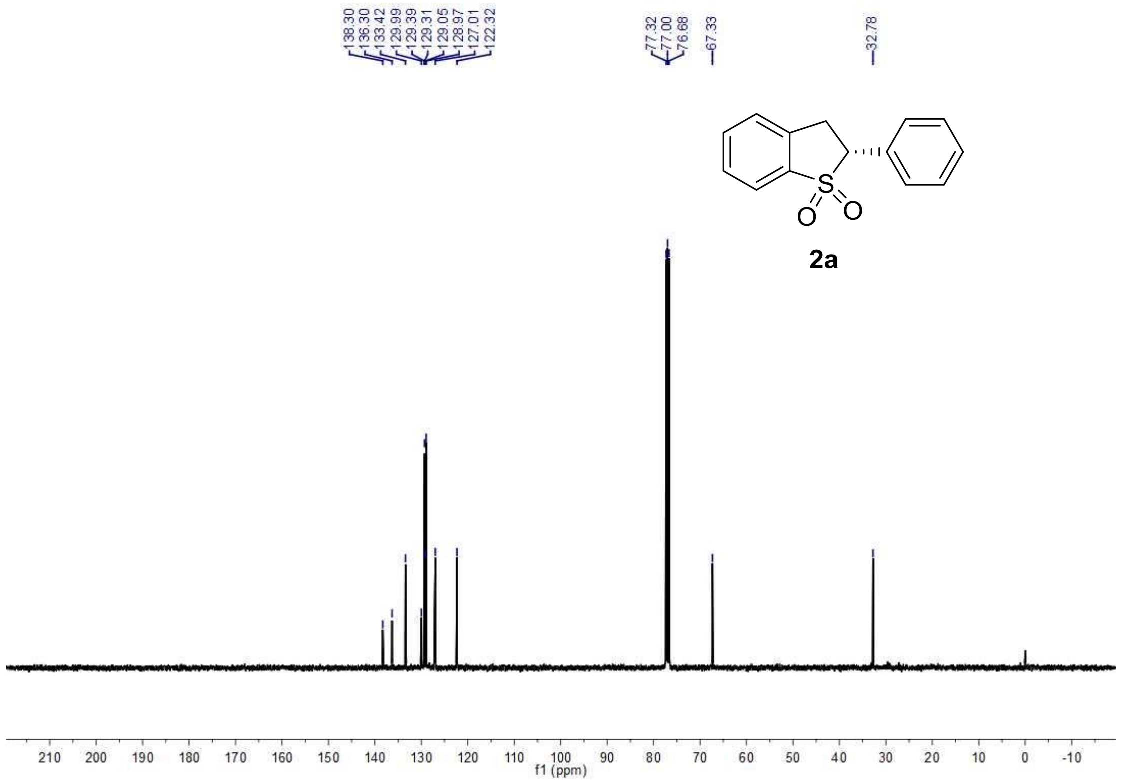

138.30 136.30 133.42 129.99 129.39 129.31 129.05 128.97 127.01 122.32
77.32 77.00 76.68
67.33
32.78
f1 (ppm)

2a

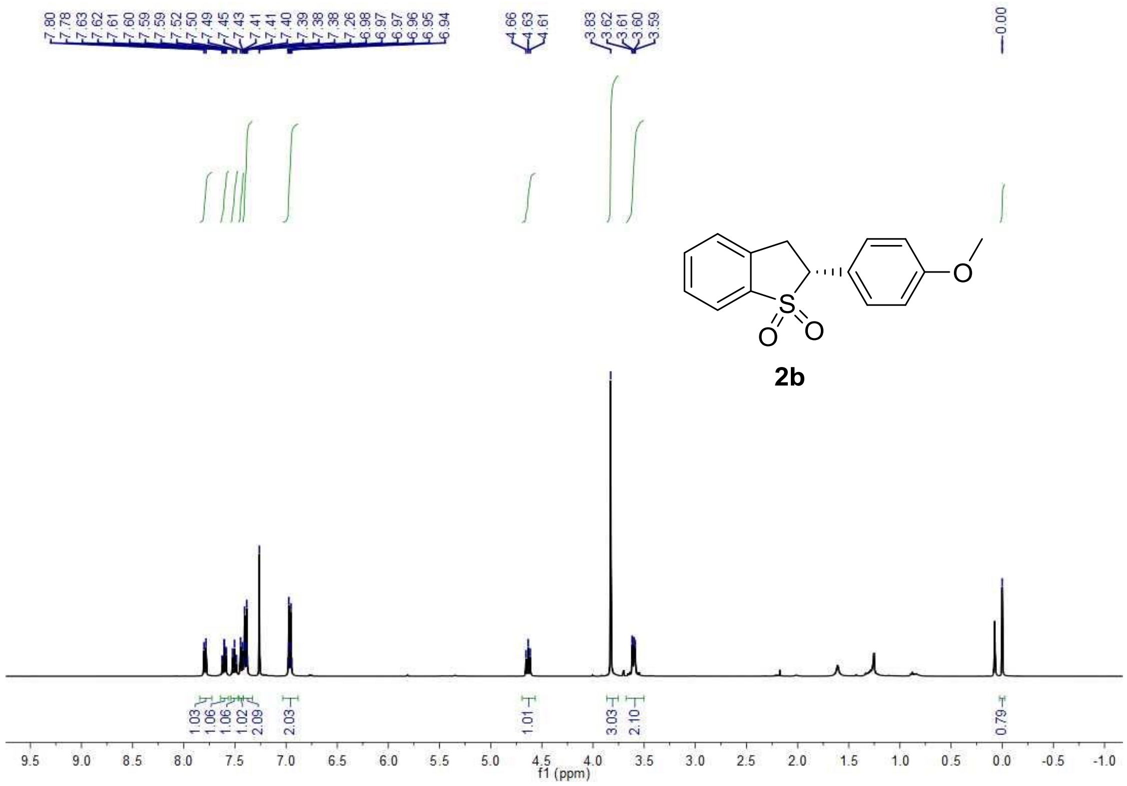
7.80 7.78 7.63 7.62 7.61 7.60 7.59 7.59 7.52 7.50 7.49 7.45 7.43 7.41 7.41 7.40 7.39 7.38 7.38 7.26 6.98 6.97 6.97 6.96 6.95 6.94
4.66 4.63 4.61
3.83 3.62 3.61 3.60 3.59
0.00
1.03 1.06 1.06 1.02 2.09 2.03 1.01 3.03 2.10 0.79
f1 (ppm)

2b

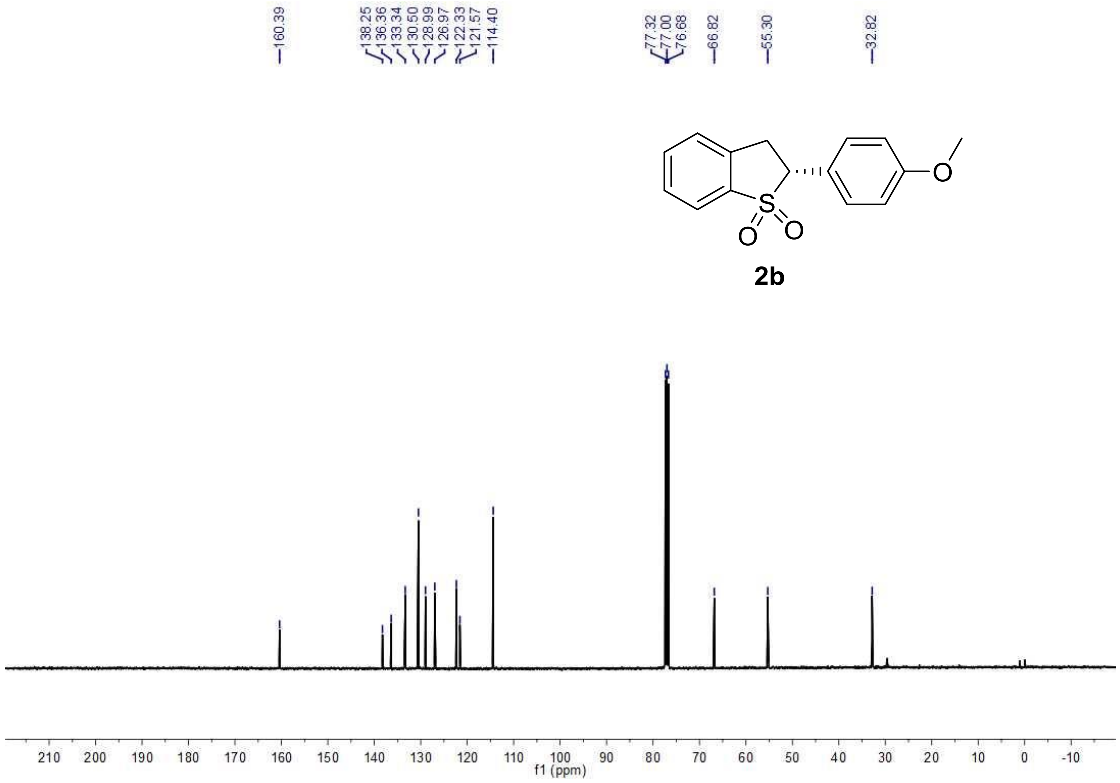
160.39
138.25 136.36 133.34 130.50 128.99 126.97 122.33 121.57 114.40
77.32 77.00 76.68
66.82
55.30
32.82
f1 (ppm)

2b

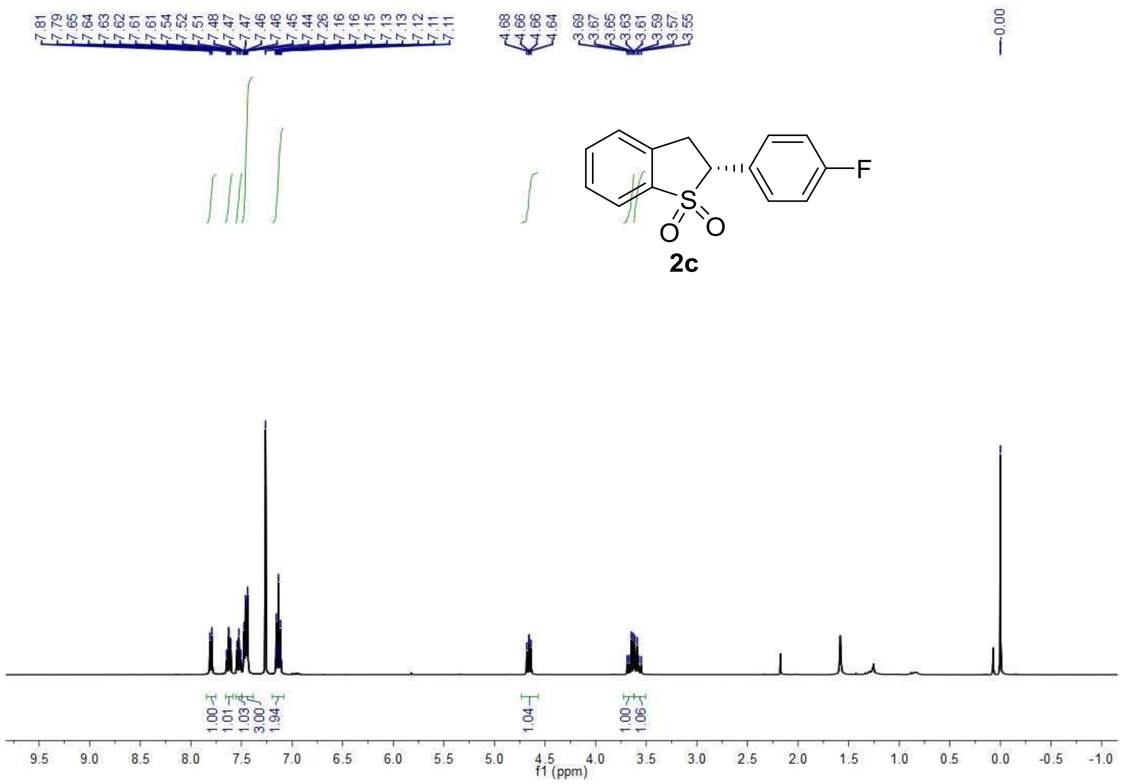

7.81 7.79 7.65 7.64 7.63 7.62 7.61 7.61 7.54 7.52 7.51 7.48 7.47 7.47 7.46 7.46 7.45 7.44 7.26 7.16 7.16 7.15 7.13 7.13 7.12 7.11 7.11
4.68 4.66 4.66 4.64
3.69 3.67 3.65 3.63 3.61 3.59 3.57 3.55
0.00
F
S
O
O
2c
1.00 1.01 1.03 3.00 1.94
1.04
1.00 1.06
9.5 9.0 8.5 8.0 7.5 7.0 6.5 6.0 5.5 5.0 4.5 4.0 3.5 3.0 2.5 2.0 1.5 1.0 0.5 0.0 -0.5 -1.0
f1 (ppm)

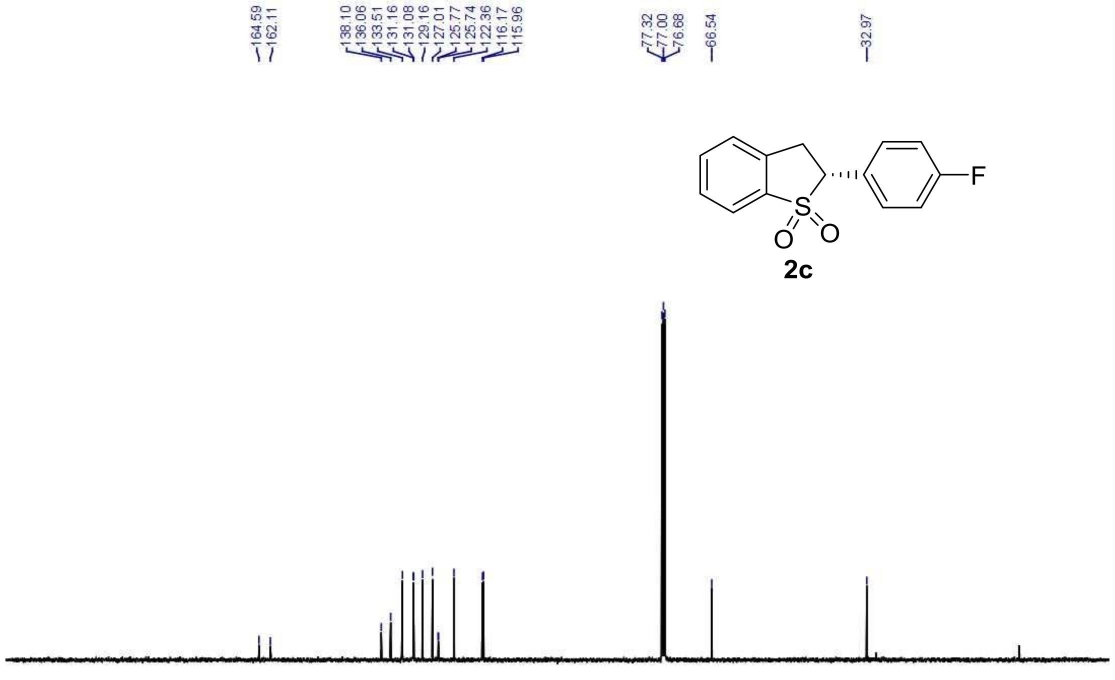

164.59 162.11
138.10 136.06 133.51 131.16 131.08 129.16 127.01 125.77 125.74 122.36 116.17 115.96
77.32 77.00 76.68
66.54
32.97
F
S
O
O
2c
210 200 190 180 170 160 150 140 130 120 110 100 90 80 70 60 50 40 30 20 10 0 -10
f1 (ppm)

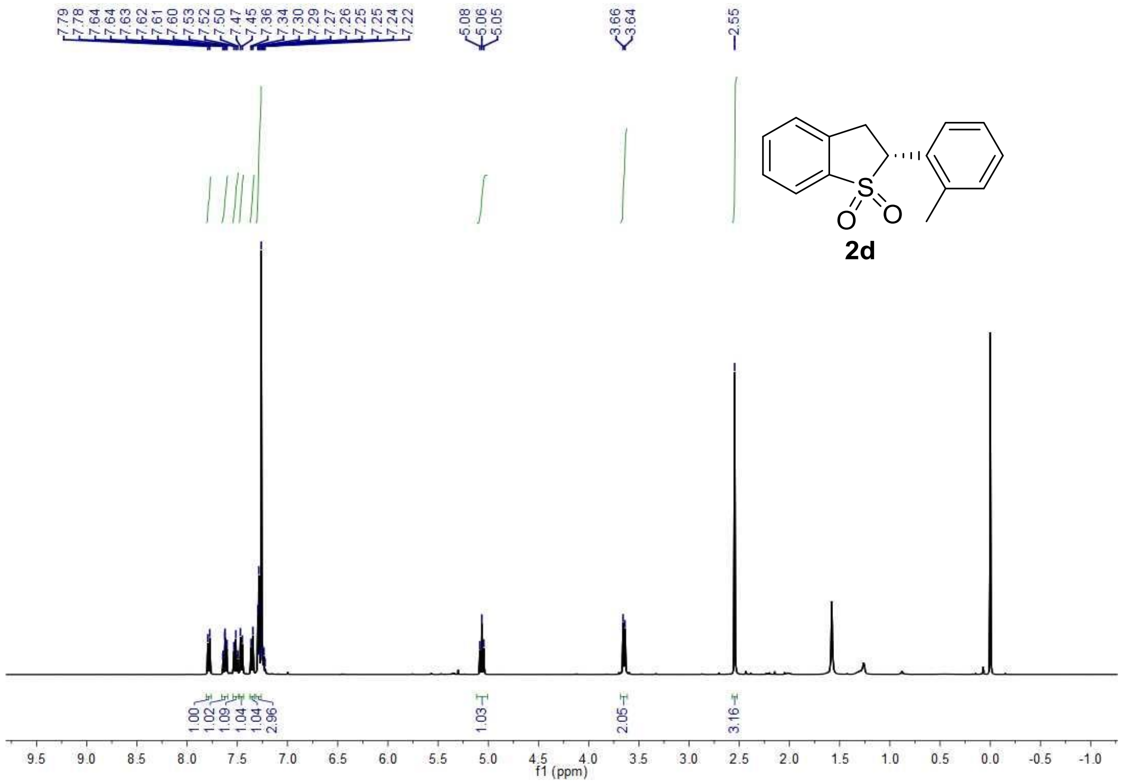
7.79 7.78 7.64 7.64 7.63 7.62 7.61 7.60 7.53 7.52 7.50 7.47 7.45 7.36 7.34 7.30 7.29 7.27 7.26 7.25 7.25 7.24 7.22
5.08 5.06 5.05
3.66 3.64
2.55
2d
1.00 1.02 1.09 1.04 1.04 2.96
1.03
2.05
3.16
f1 (ppm)

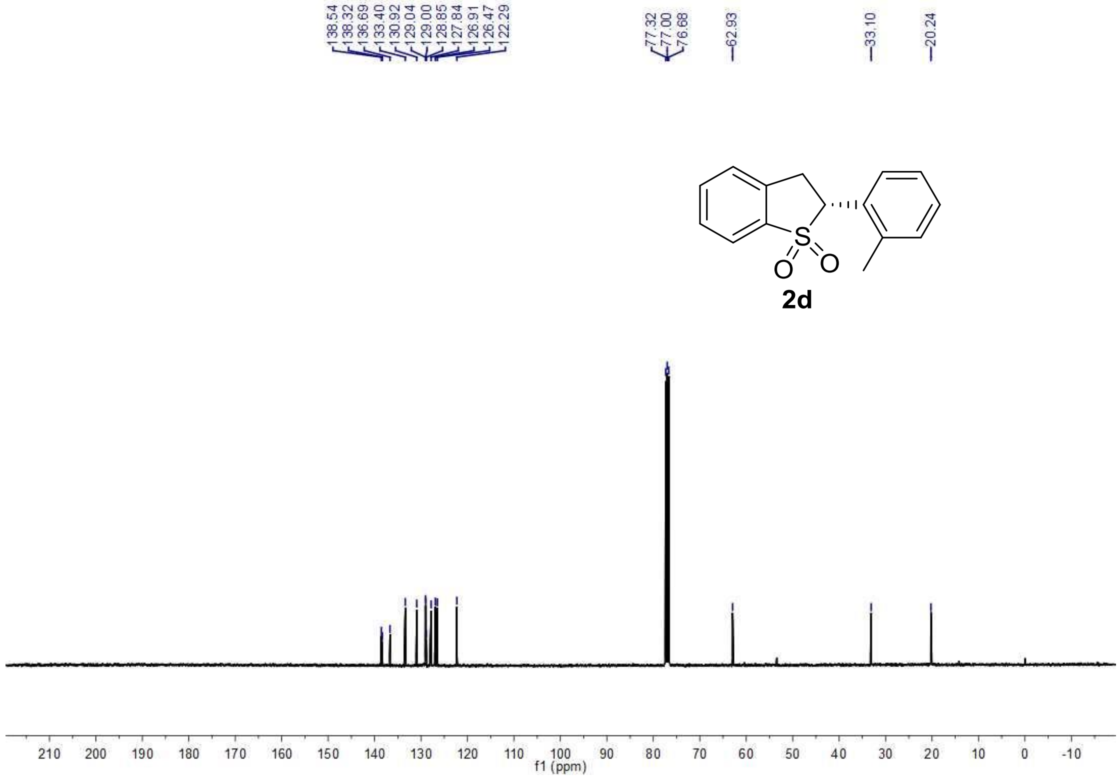
138.54 138.32 136.69 133.40 130.92 129.04 129.00 128.85 127.84 126.91 126.47 122.29
77.32 77.00 76.68
62.93
33.10
20.24
2d
f1 (ppm)

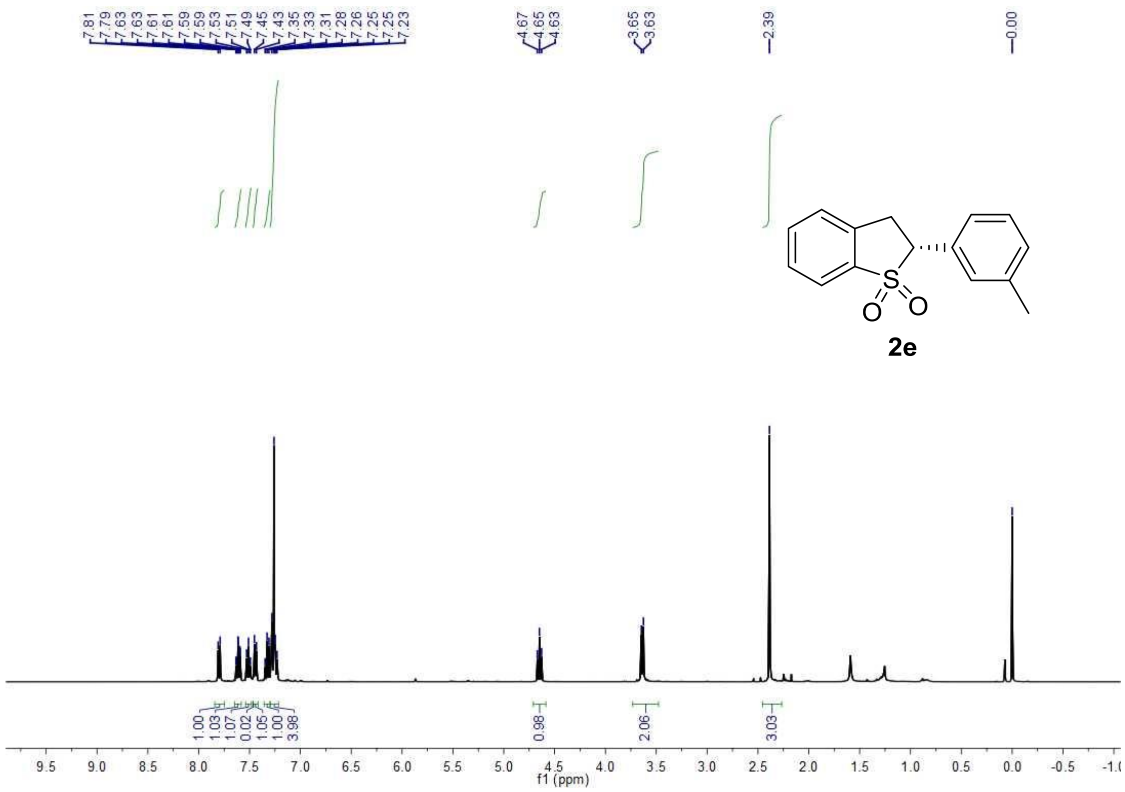

7.81 7.79 7.63 7.63 7.61 7.61 7.59 7.53 7.51 7.49 7.45 7.43 7.35 7.33 7.31 7.28 7.26 7.25 7.25 7.23
4.67 4.65 4.63
3.65 3.63
2.39
0.00
2e
1.00 1.03 1.07 0.02 1.05 1.00 3.98
0.98
2.06
3.03
f1 (ppm)

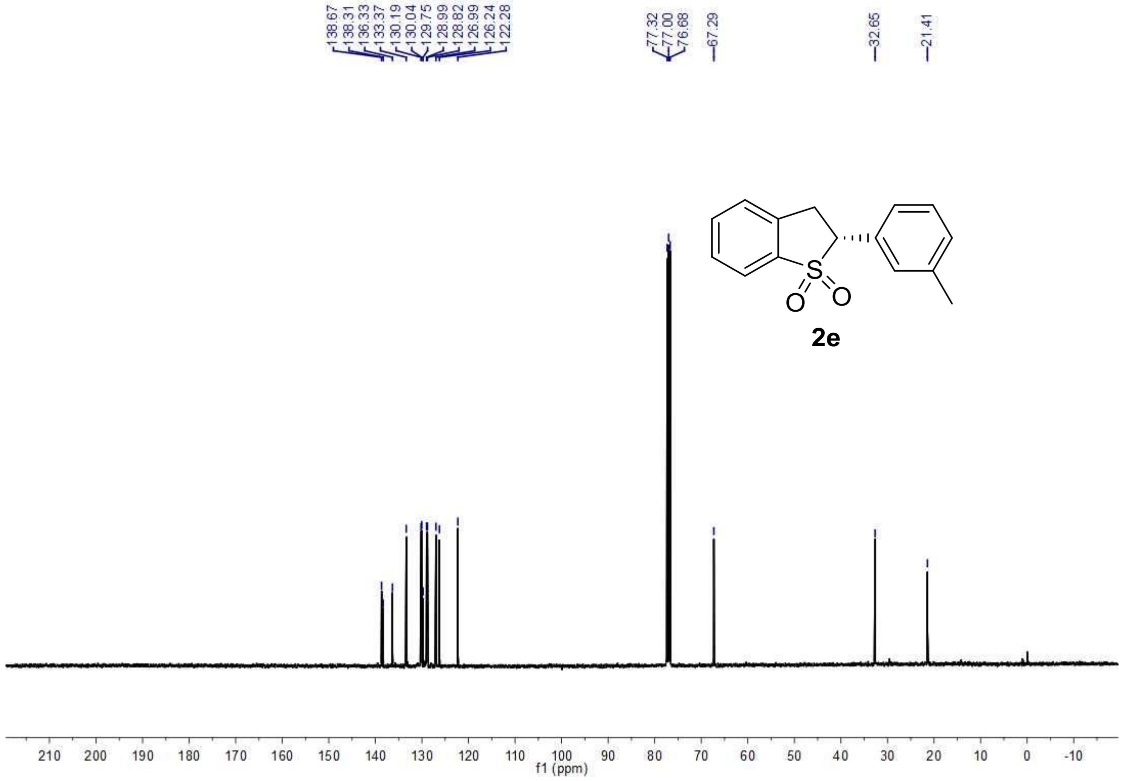

138.67 138.31 136.33 133.37 130.19 130.04 129.75 128.99 128.82 126.99 126.24 122.28
77.32 77.00 76.68
67.29
32.65
21.41
2e
f1 (ppm)

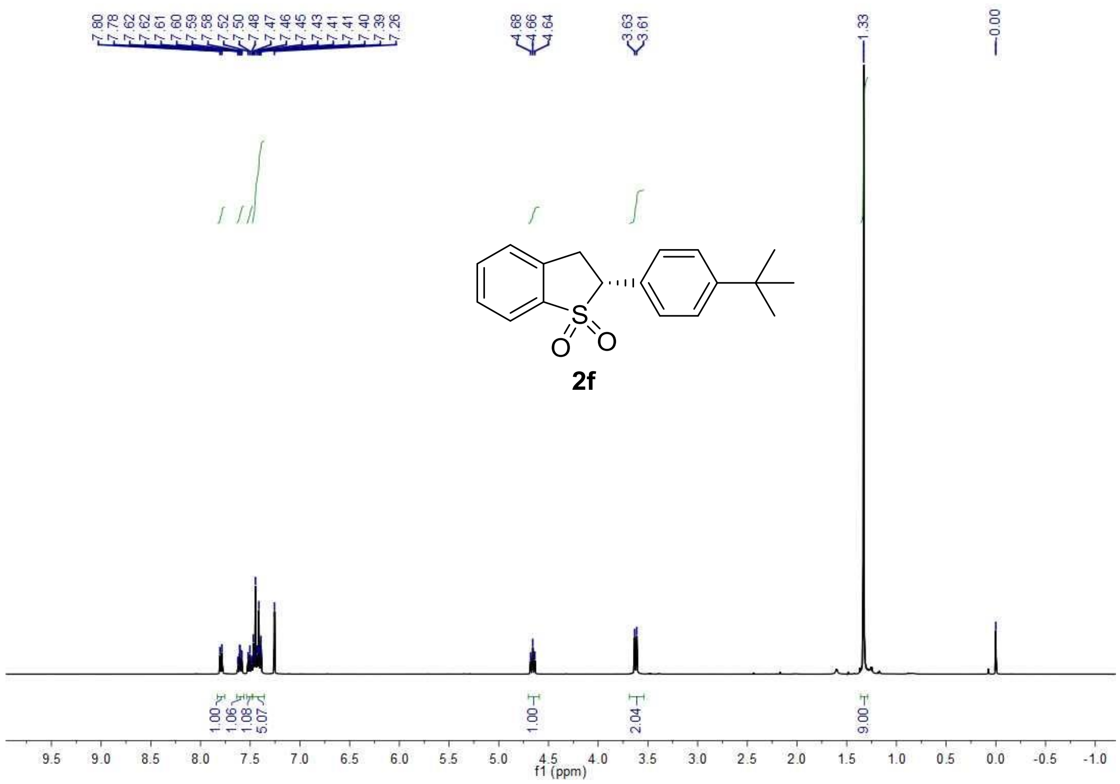
7.80 7.78 7.62 7.61 7.60 7.59 7.58 7.52 7.50 7.48 7.47 7.46 7.45 7.43 7.41 7.40 7.39 7.26
4.68 4.66 4.64
3.63 3.61
1.33
0.00
2f
1.00 1.06 1.08 5.07
1.00
2.04
9.00
9.5 9.0 8.5 8.0 7.5 7.0 6.5 6.0 5.5 5.0 4.5 4.0 3.5 3.0 2.5 2.0 1.5 1.0 0.5 0.0 -0.5 -1.0
f1 (ppm)

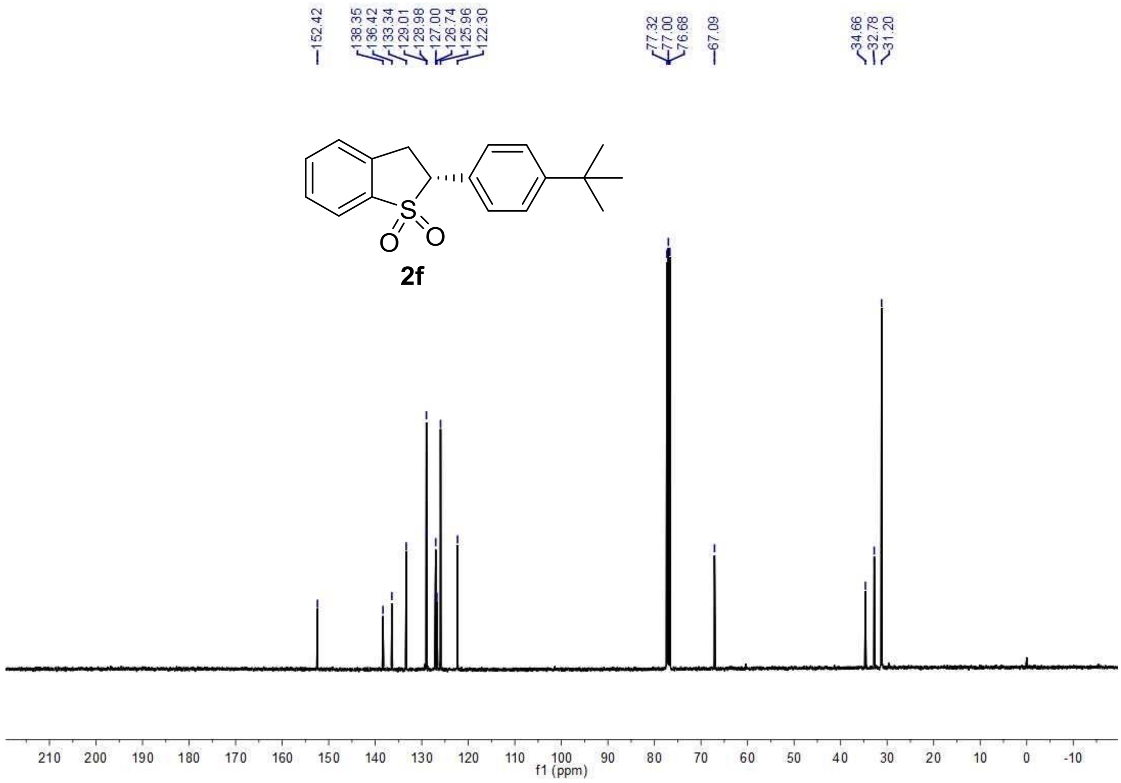
152.42
138.35 136.42 133.34 129.01 128.98 127.00 126.74 125.96 122.30
77.32 77.00 76.68
67.09
34.66 32.78 31.20
2f
210 200 190 180 170 160 150 140 130 120 110 100 90 80 70 60 50 40 30 20 10 0 -10
f1 (ppm)

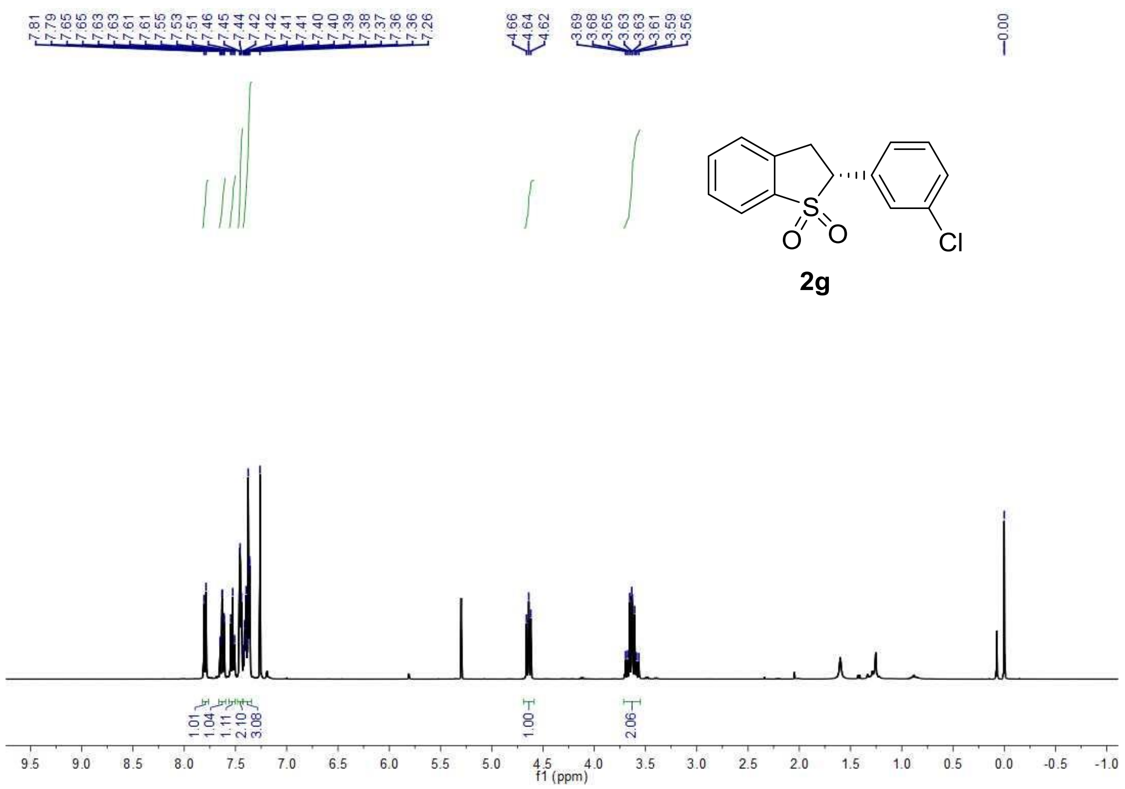

7.81 7.79 7.65 7.65 7.63 7.63 7.61 7.61 7.55 7.53 7.51 7.46 7.45 7.44 7.42 7.42 7.41 7.41 7.40 7.39 7.38 7.37 7.36 7.36 7.26
4.66 4.64 4.62
3.69 3.68 3.65 3.63 3.63 3.61 3.59 3.56
0.00
Cl
2g
1.01 1.04 1.11 2.10 3.08
1.00
2.06
f1 (ppm)

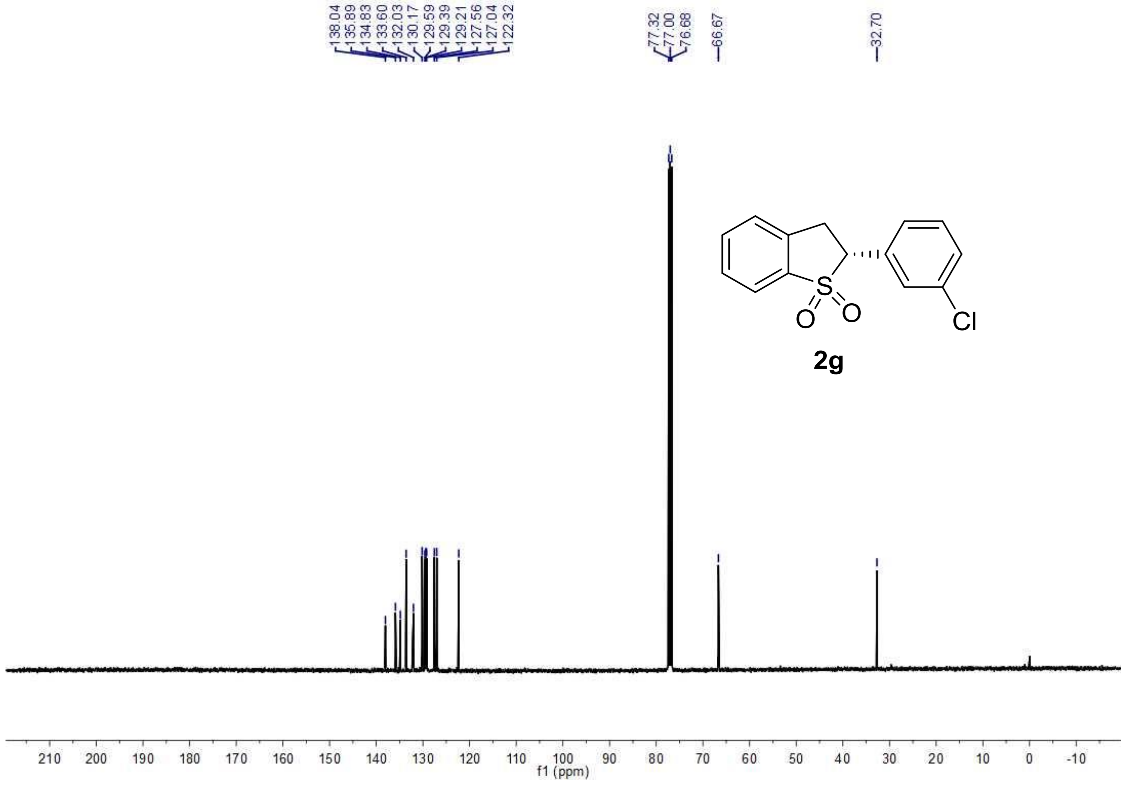

138.04 135.89 134.83 133.60 132.03 130.17 129.59 129.39 129.21 127.56 127.04 122.32
77.32 77.00 76.68
66.67
32.70
Cl
2g
f1 (ppm)

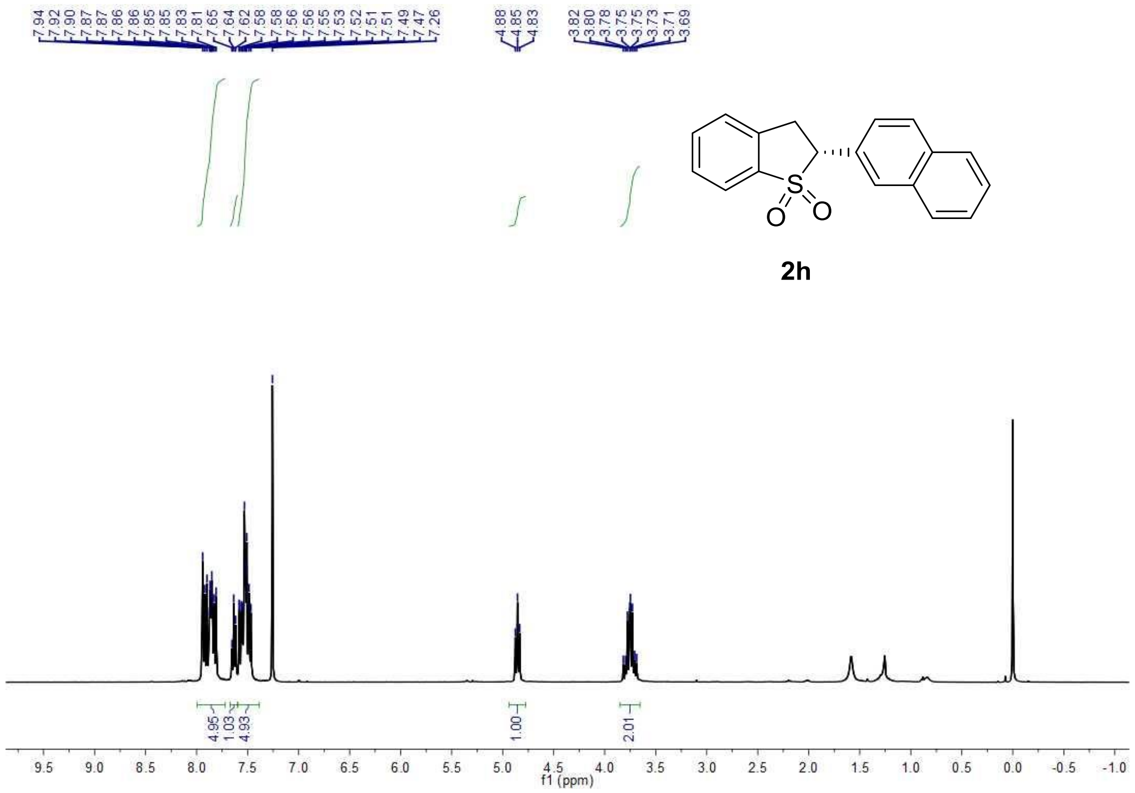
7.94 7.92 7.90 7.87 7.87 7.86 7.86 7.85 7.85 7.83 7.81 7.65 7.64 7.62 7.58 7.58 7.56 7.56 7.55 7.53 7.52 7.51 7.51 7.49 7.47 7.26
4.88 4.85 4.83
3.82 3.80 3.78 3.75 3.75 3.73 3.71 3.69
2h
4.95 1.03 4.93 1.00 2.01
9.5 9.0 8.5 8.0 7.5 7.0 6.5 6.0 5.5 5.0 4.5 4.0 3.5 3.0 2.5 2.0 1.5 1.0 0.5 0.0 -0.5 -1.0
f1 (ppm)

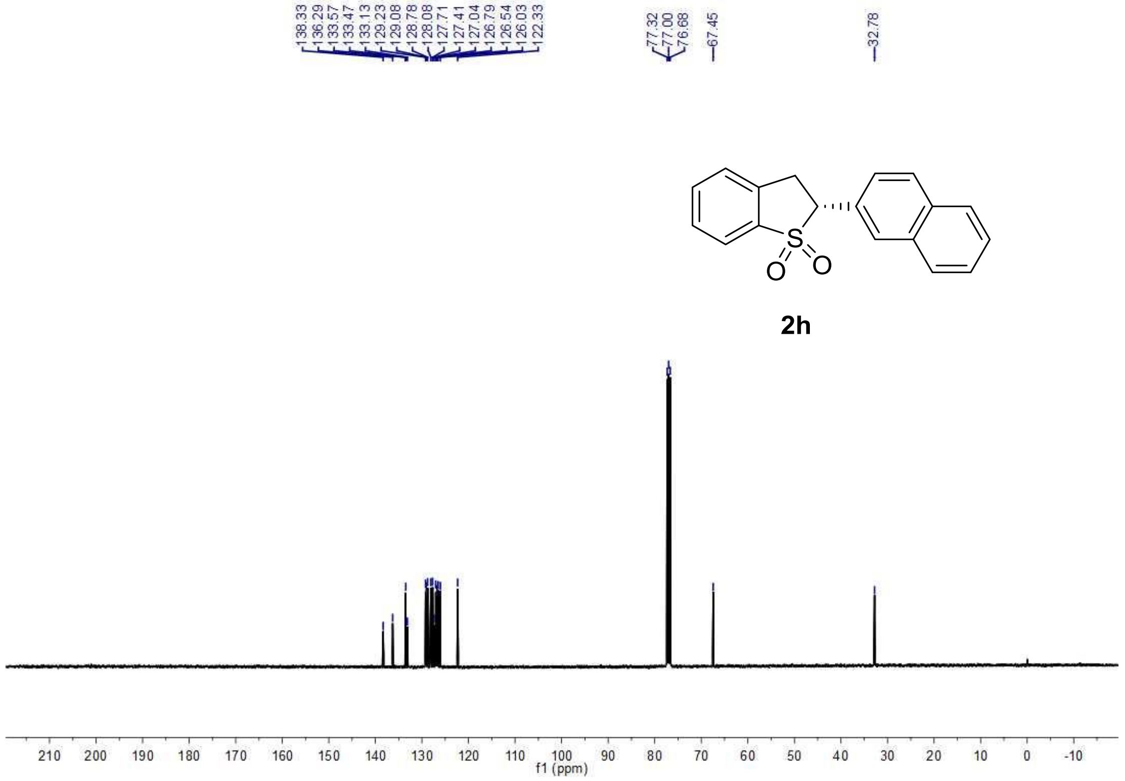
138.33 136.29 133.57 133.47 133.13 129.23 129.08 128.78 128.08 127.71 127.41 127.04 126.79 126.54 126.03 122.33
77.32 77.00 76.68
67.45
32.78
2h
210 200 190 180 170 160 150 140 130 120 110 100 90 80 70 60 50 40 30 20 10 0 -10
f1 (ppm)

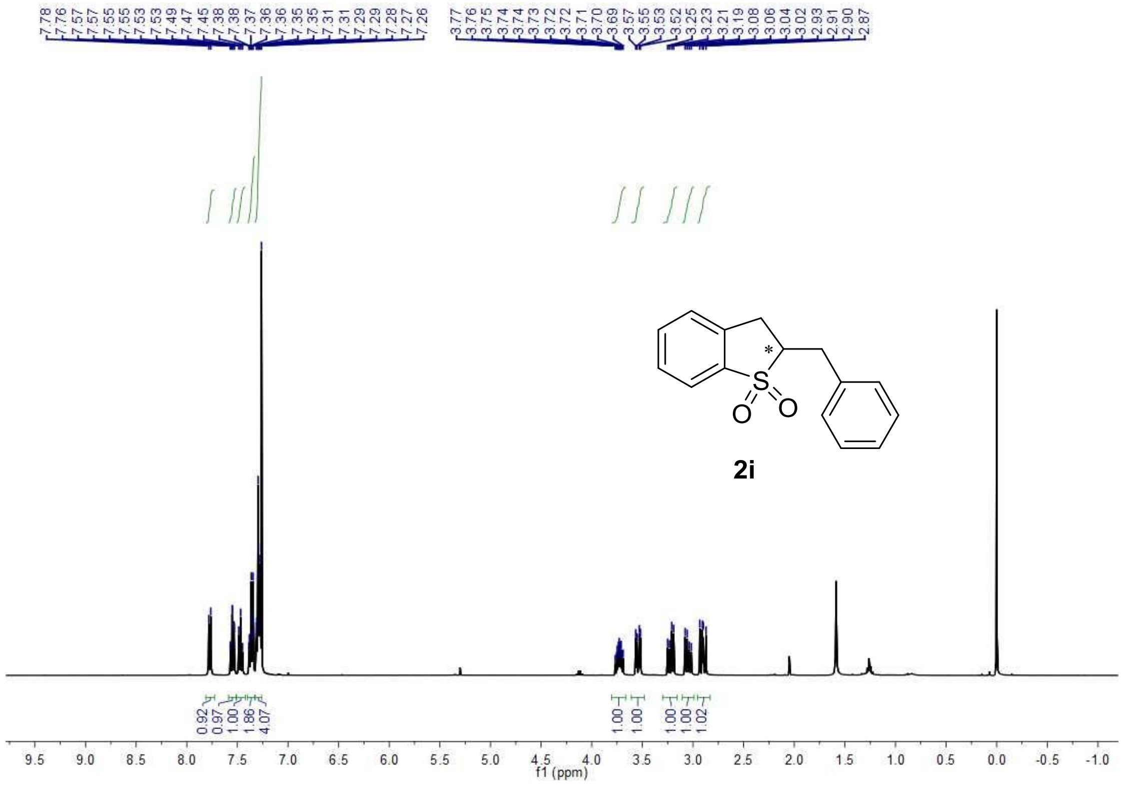

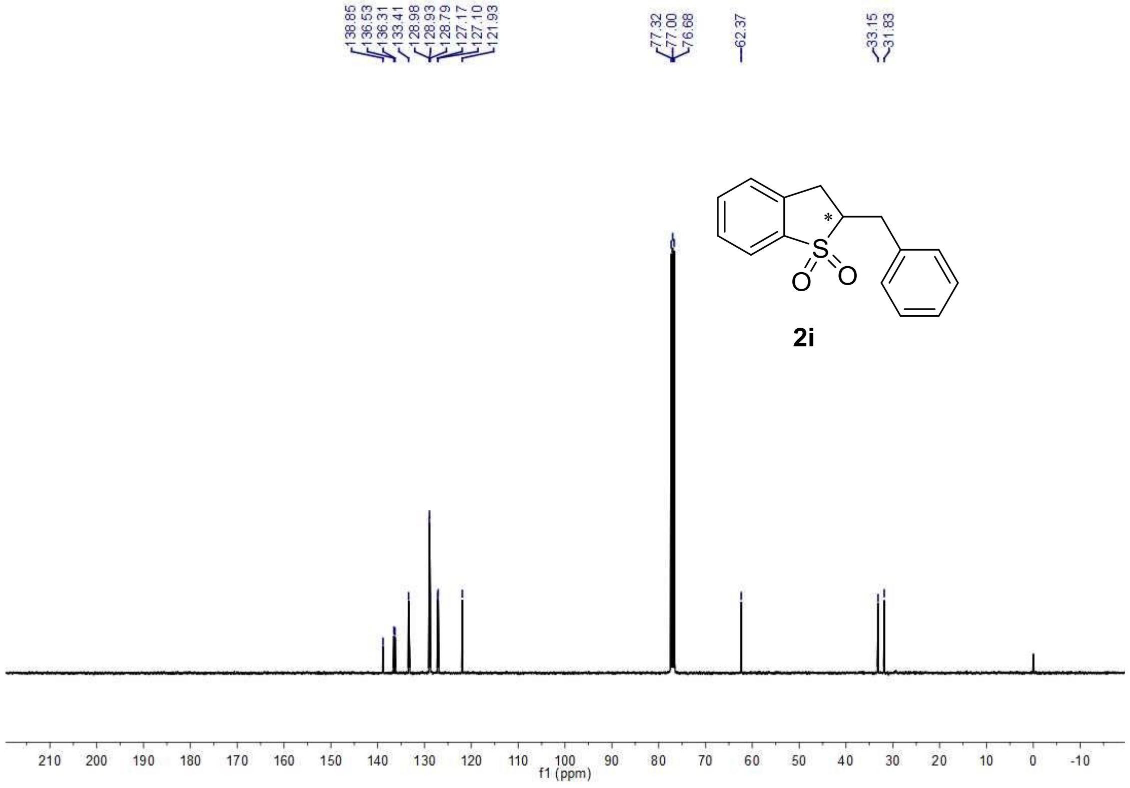

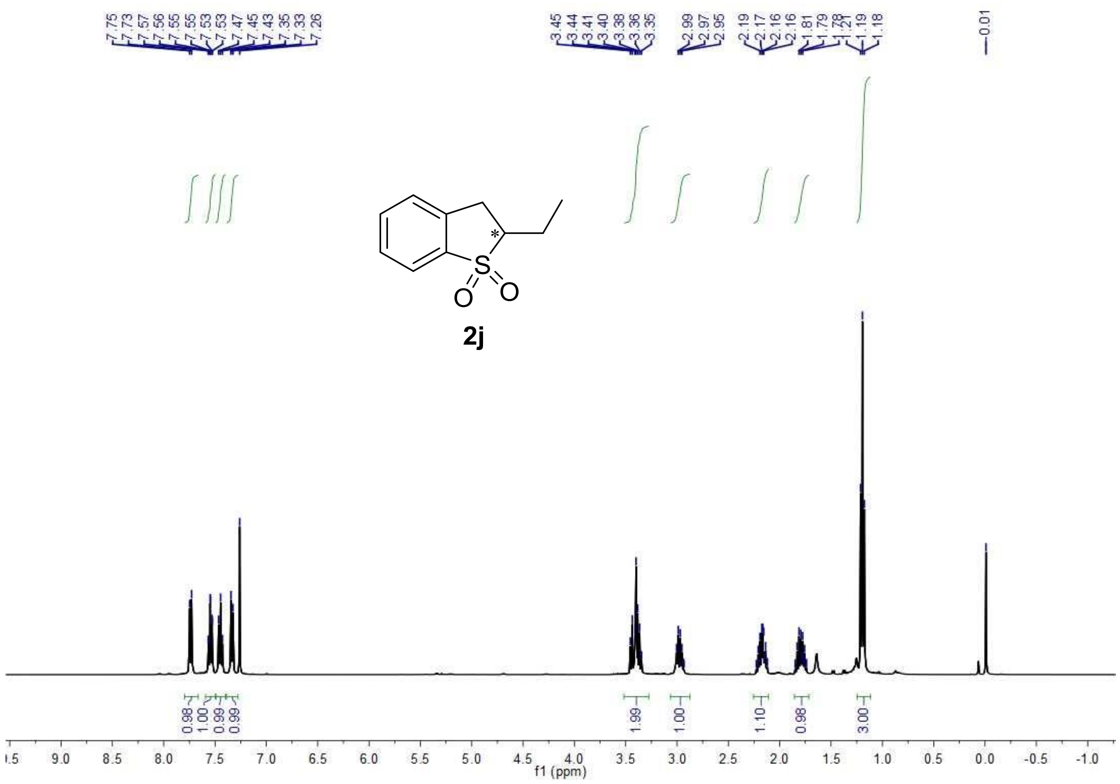

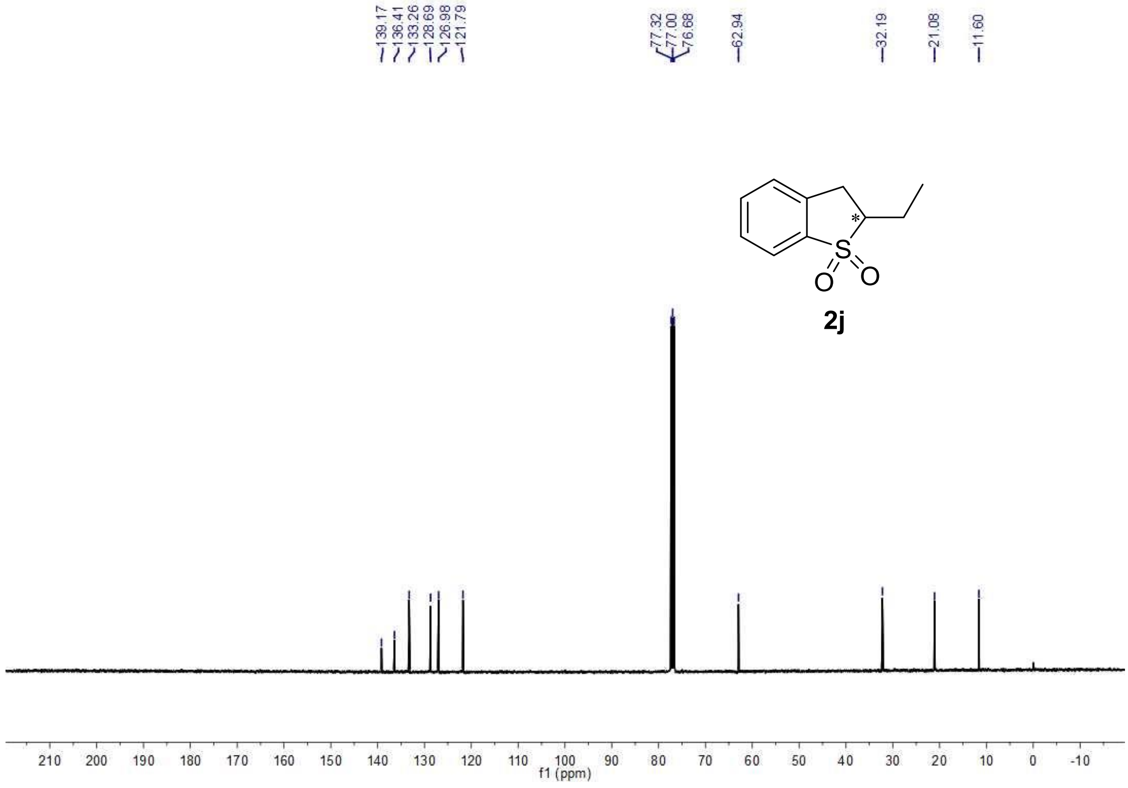

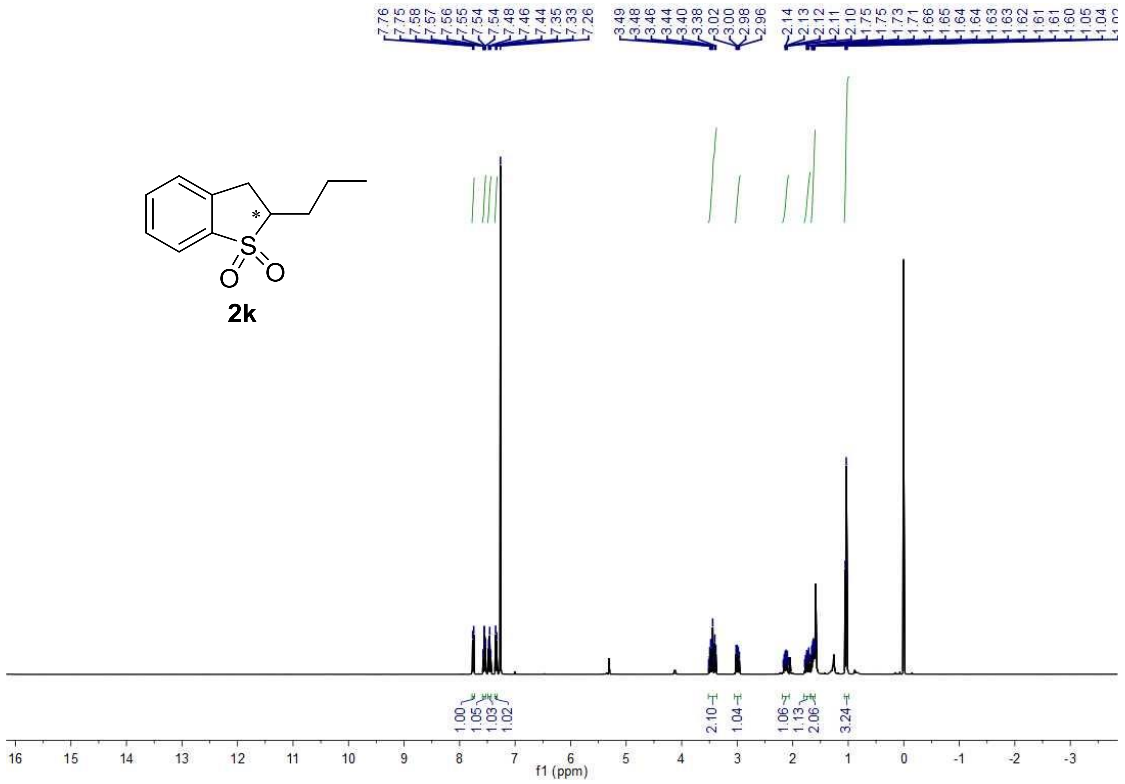
7.76
7.75
7.58
7.57
7.56
7.55
7.54
7.54
7.48
7.46
7.44
7.35
7.33
7.26
3.49
3.48
3.46
3.44
3.40
3.38
3.02
3.00
2.98
2.96
2.14
2.13
2.12
2.11
2.10
1.75
1.75
1.73
1.71
1.66
1.65
1.64
1.64
1.63
1.63
1.62
1.61
1.61
1.60
1.05
1.04
2k
1.00
1.05
1.03
1.02
2.10
1.04
1.06
1.13
2.06
3.24
f1 (ppm)

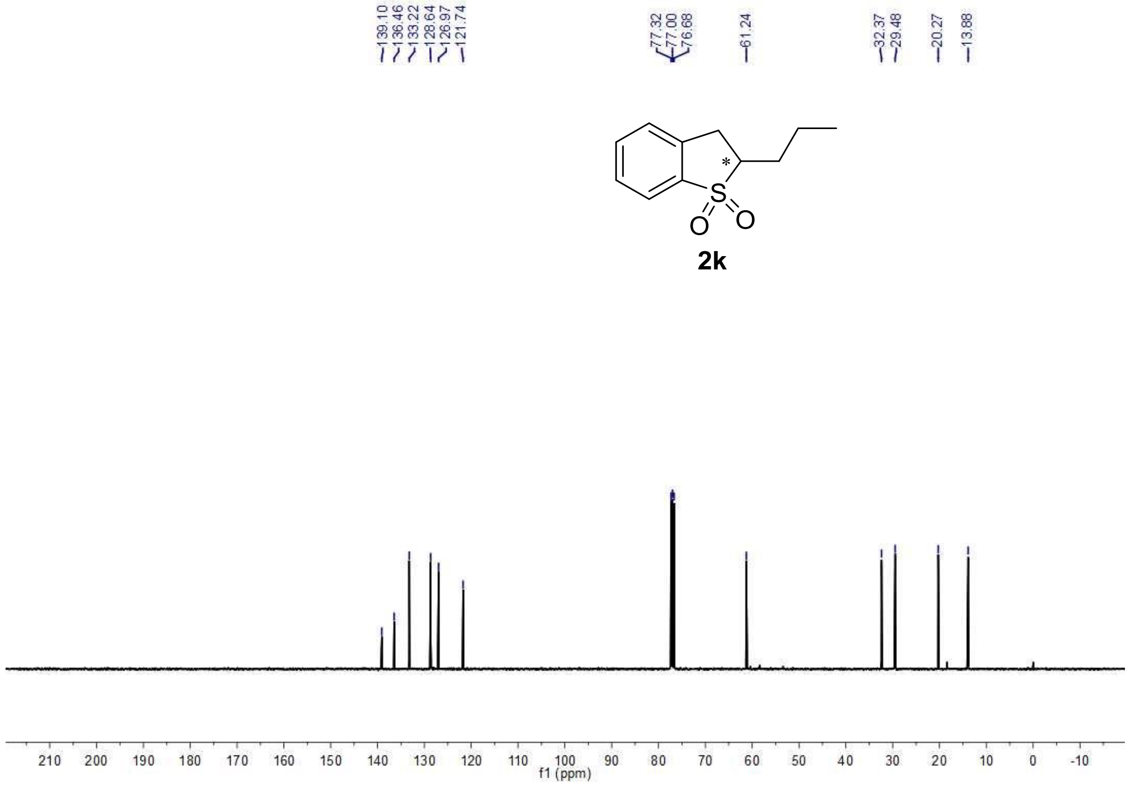
139.10
136.46
133.22
128.64
126.97
121.74
77.32
77.00
76.68
61.24
32.37
29.48
20.27
13.88
2k
f1 (ppm)

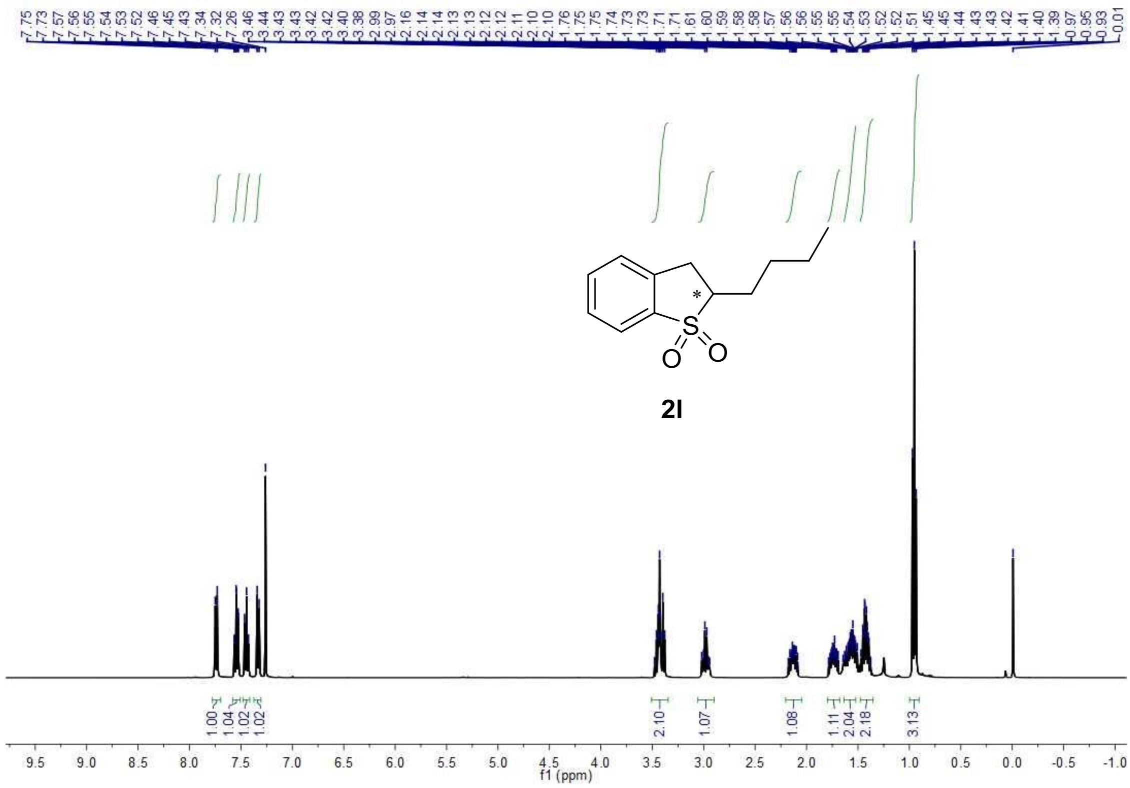
S
O
O
2l
f1 (ppm)

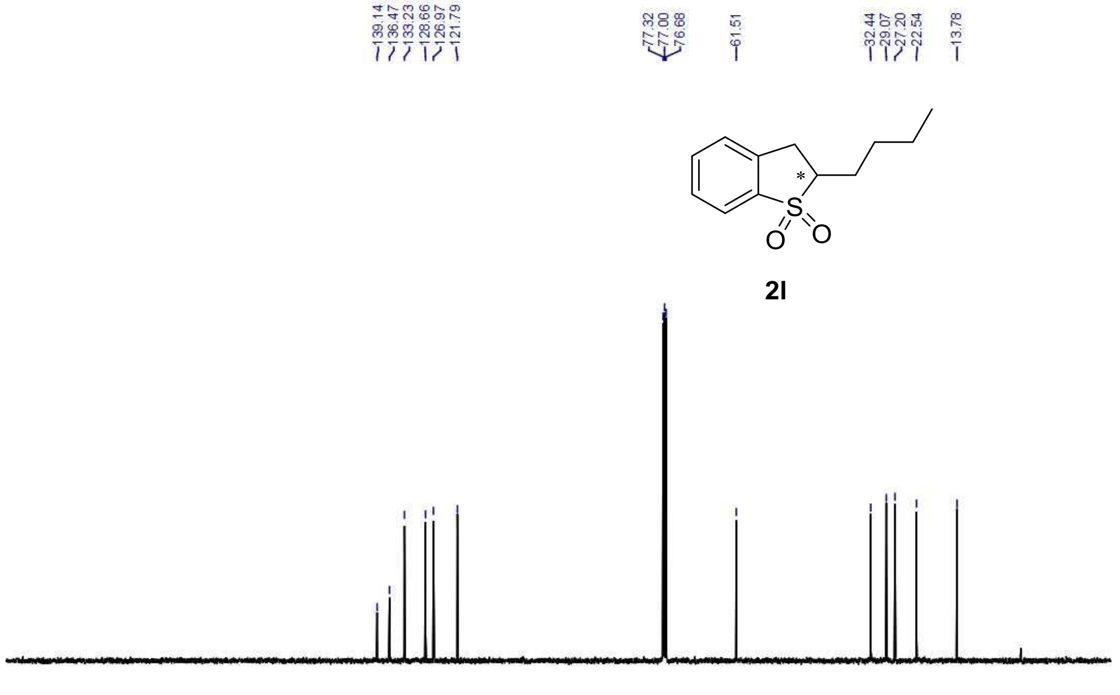
139.14
136.47
133.23
128.66
126.97
121.79
77.32
77.00
76.68
61.51
32.44
29.07
27.20
22.54
13.78
S
O
O
2l
f1 (ppm)

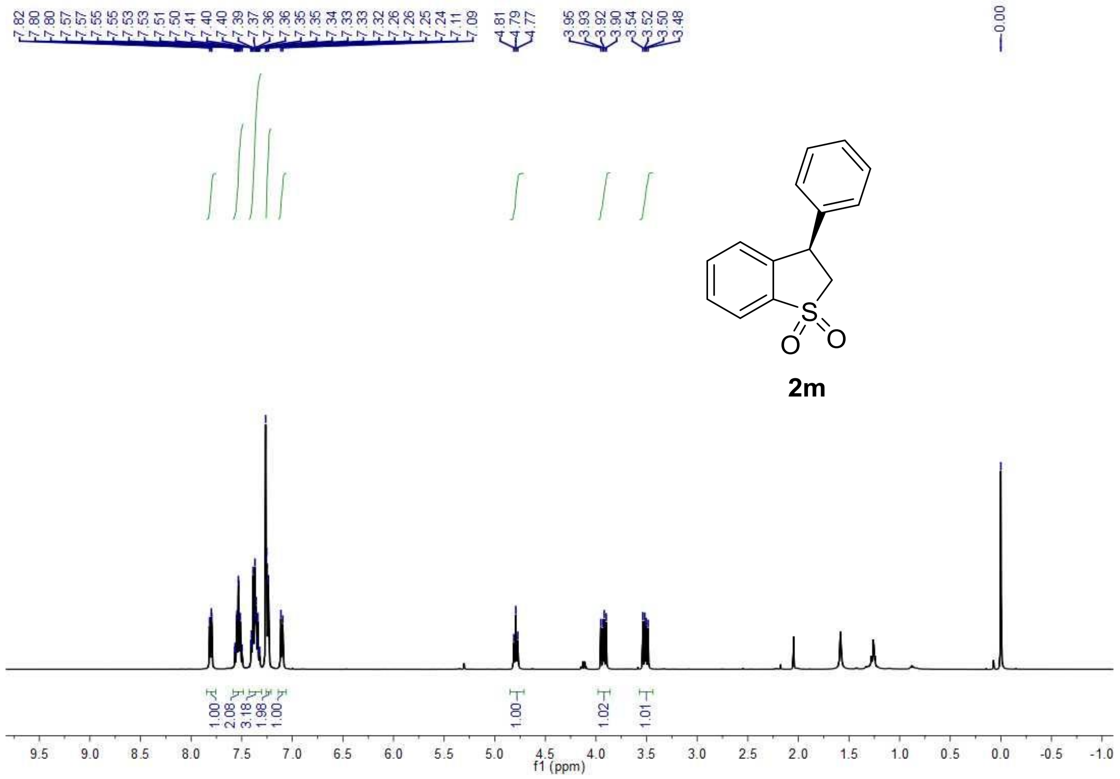
7.82 7.80 7.57 7.57 7.55 7.53 7.53 7.51 7.50 7.41 7.40 7.39 7.37 7.36 7.36 7.35 7.34 7.33 7.33 7.32 7.26 7.26 7.25 7.24 7.11 7.09
4.81 4.79 4.77
3.95 3.93 3.92 3.90 3.54 3.52 3.50 3.48
0.00
2m
1.00 2.08 3.18 1.98 1.00
1.00
1.02
1.01
f1 (ppm)

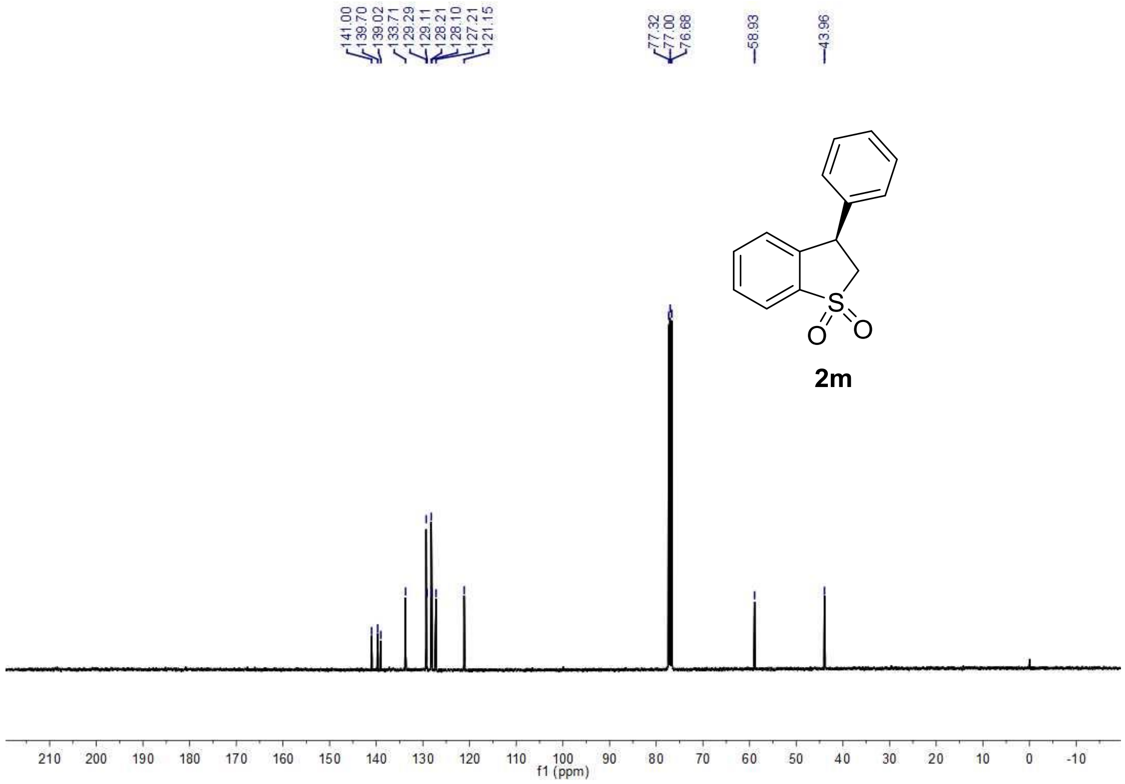
141.00 139.70 139.02 133.71 129.29 129.11 128.21 128.10 127.21 121.15
77.32 77.00 76.68
58.93
43.96
2m
f1 (ppm)

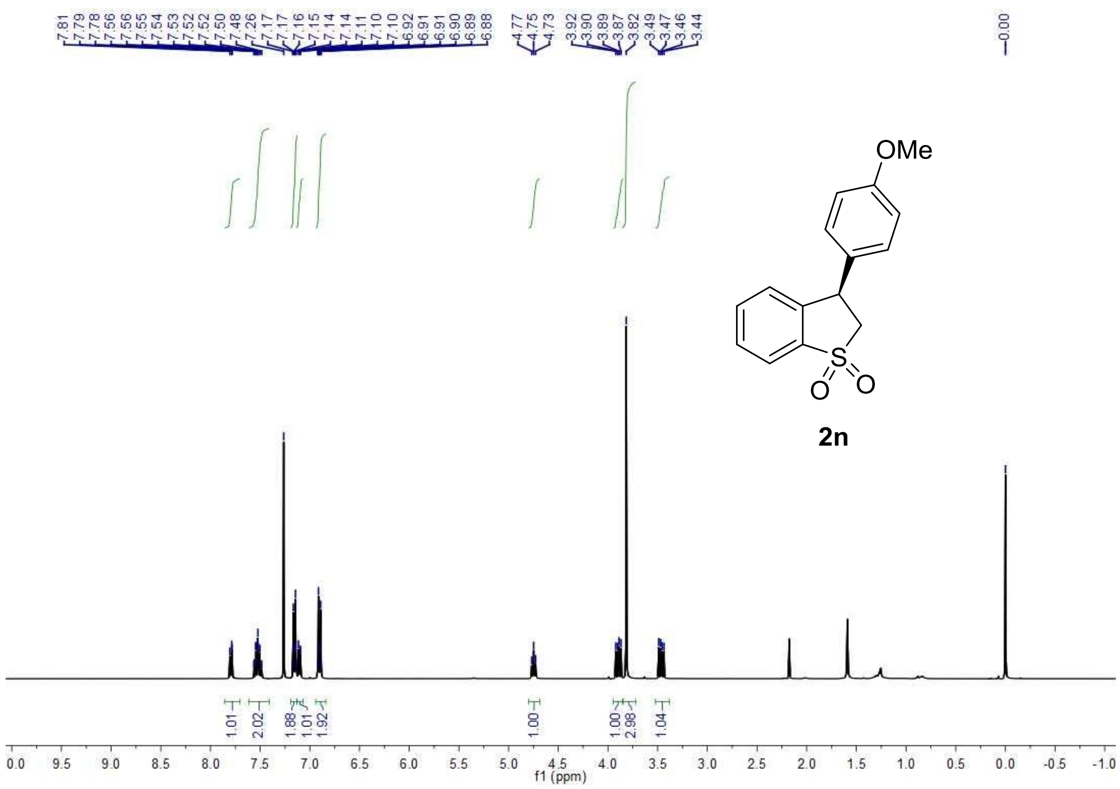
7.81 7.79 7.78 7.56 7.55 7.54 7.53 7.52 7.50 7.48 7.26 7.17 7.17 7.16 7.15 7.14 7.11 7.10 6.92 6.91 6.91 6.90 6.89 6.88
4.77 4.75 4.73
3.92 3.90 3.89 3.87 3.82 3.49 3.47 3.46 3.44
0.00
OMe
S
O
O
2n
1.01 2.02 1.88 1.01 1.92 1.00 1.00 2.98 1.04
f1 (ppm)

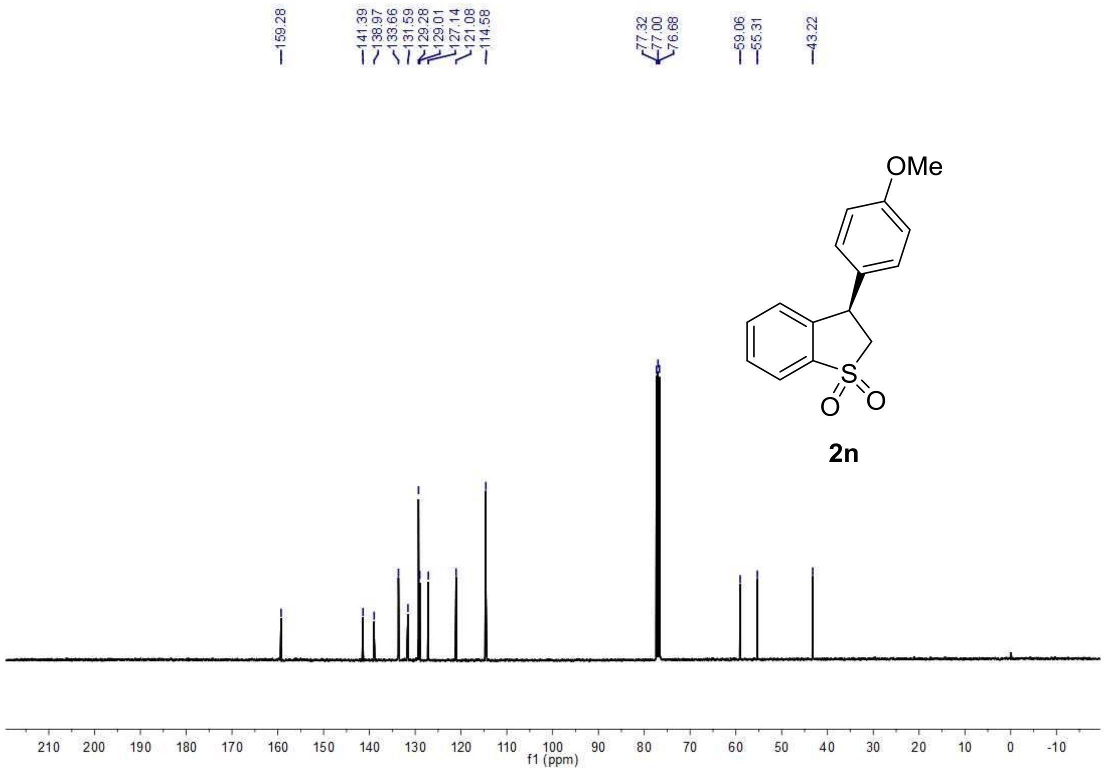
159.28
141.39 138.97 133.66 131.59 129.28 129.01 127.14 121.08 114.58
77.32 77.00 76.68
59.06 55.31
43.22
OMe
S
O
O
2n
f1 (ppm)

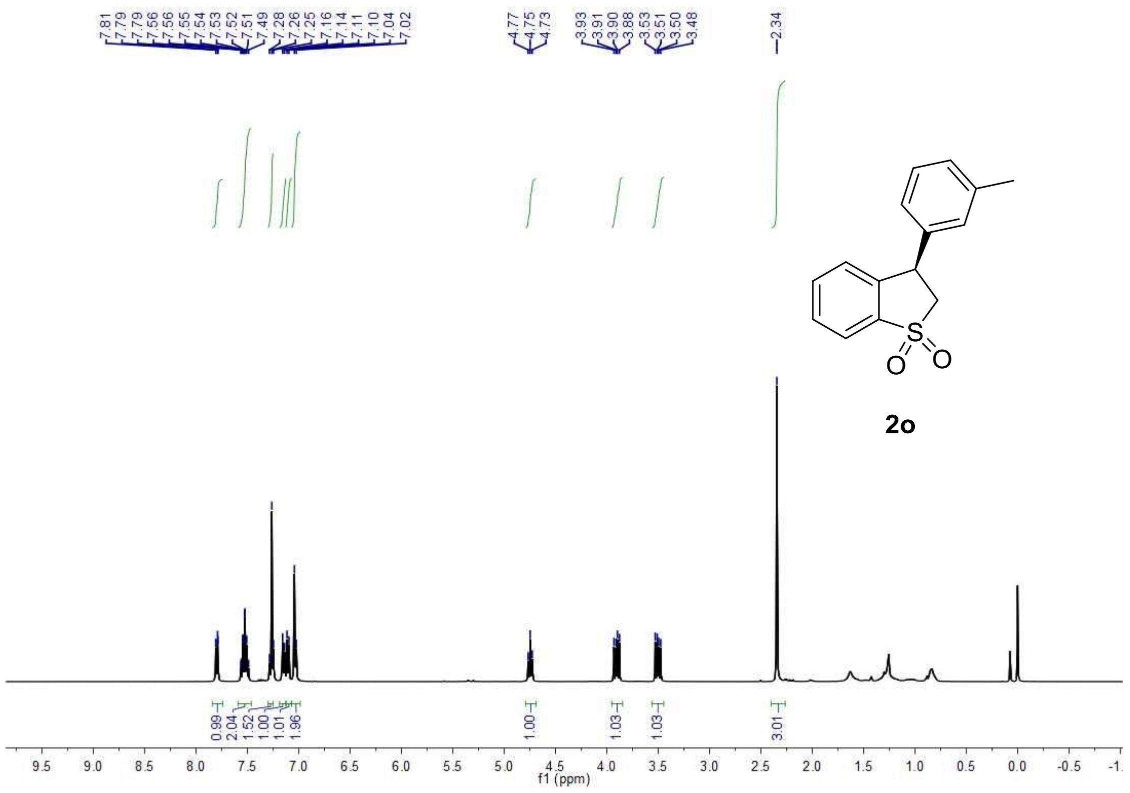
7.81 7.79 7.79 7.56 7.55 7.54 7.53 7.52 7.51 7.49 7.28 7.26 7.25 7.16 7.14 7.11 7.10 7.04 7.02
4.77 4.75 4.73
3.93 3.91 3.90 3.88 3.53 3.51 3.50 3.48
2.34
0.99 2.04 1.52 1.00 1.01 1.96
1.00
1.03
1.03
3.01
2o
f1 (ppm)

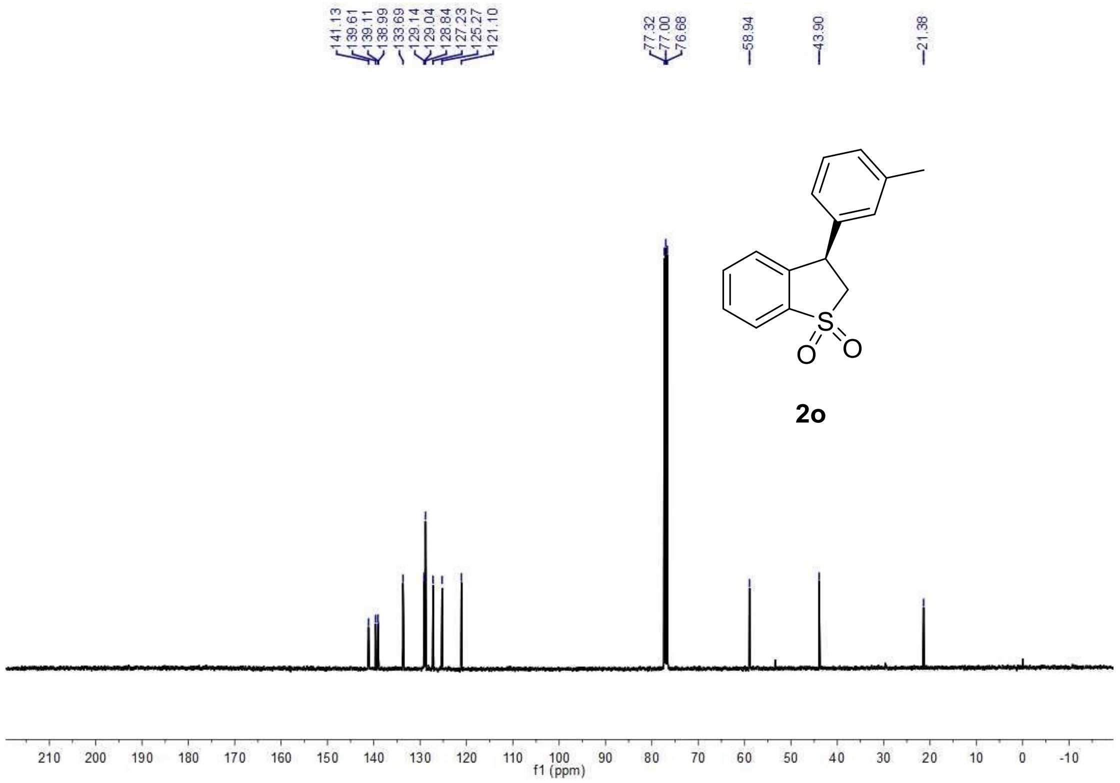
141.13 139.61 139.11 138.99 133.69 129.14 129.04 128.84 127.23 125.27 121.10
77.32 77.00 76.68
58.94
43.90
21.38
2o
f1 (ppm)

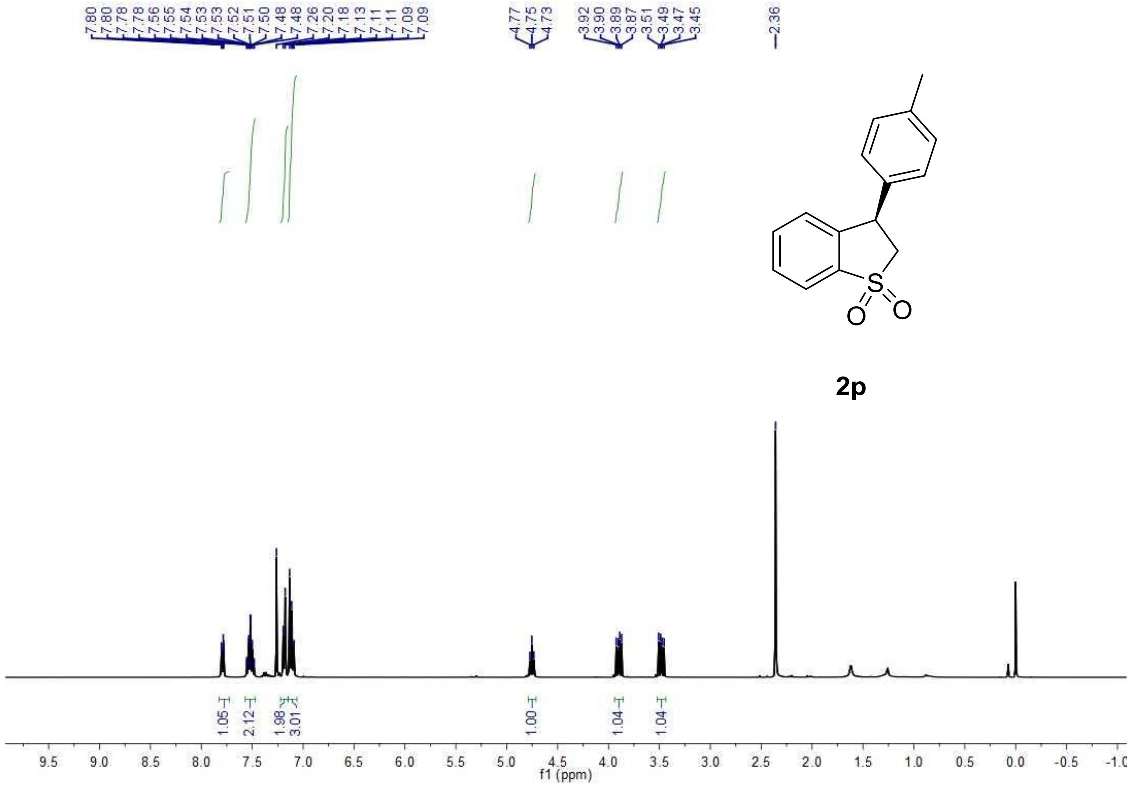
7.80 7.80 7.78 7.56 7.55 7.54 7.53 7.52 7.51 7.50 7.48 7.48 7.26 7.20 7.18 7.13 7.11 7.11 7.09 7.09
4.77 4.75 4.73
3.92 3.90 3.89 3.87 3.51 3.49 3.47 3.45
2.36
S
O
O
2p
1.05 2.12 1.98 3.01 1.00 1.04 1.04
9.5 9.0 8.5 8.0 7.5 7.0 6.5 6.0 5.5 5.0 4.5 4.0 3.5 3.0 2.5 2.0 1.5 1.0 0.5 0.0 -0.5 -1.0
f1 (ppm)

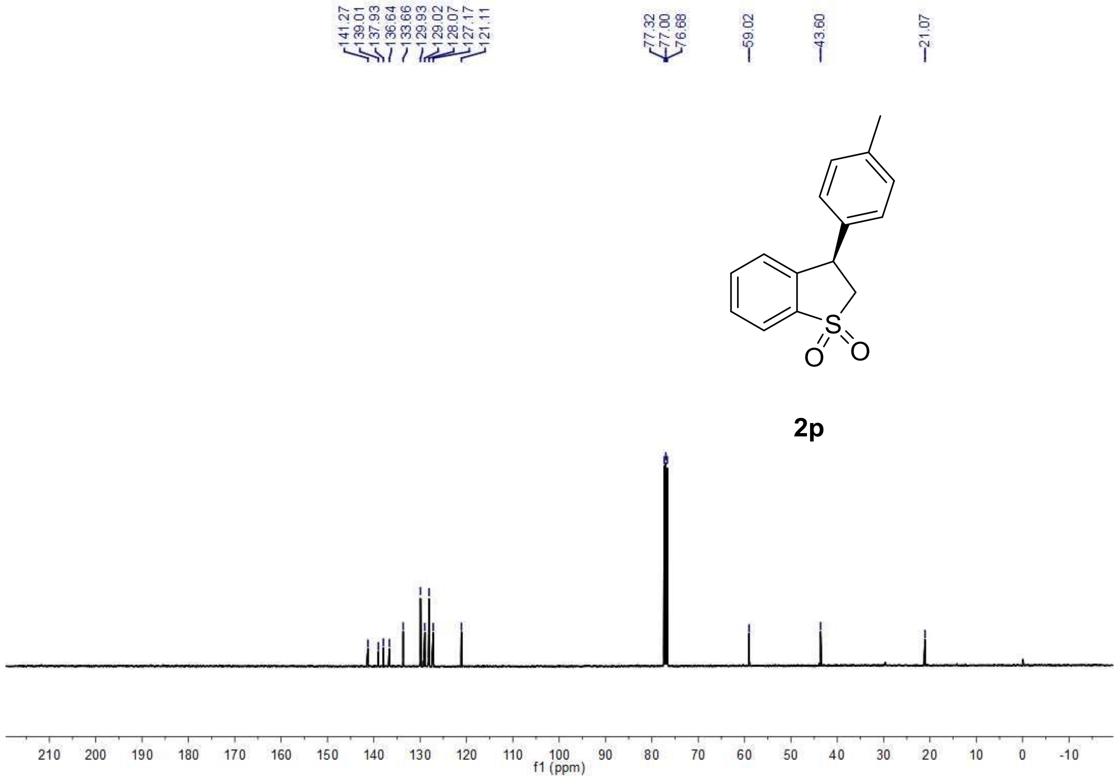
141.27 139.01 137.93 136.64 133.66 129.93 129.02 128.07 127.17 121.11
77.32 77.00 76.68
59.02
43.60
21.07
S
O
O
2p
210 200 190 180 170 160 150 140 130 120 110 100 90 80 70 60 50 40 30 20 10 0 -10
f1 (ppm)

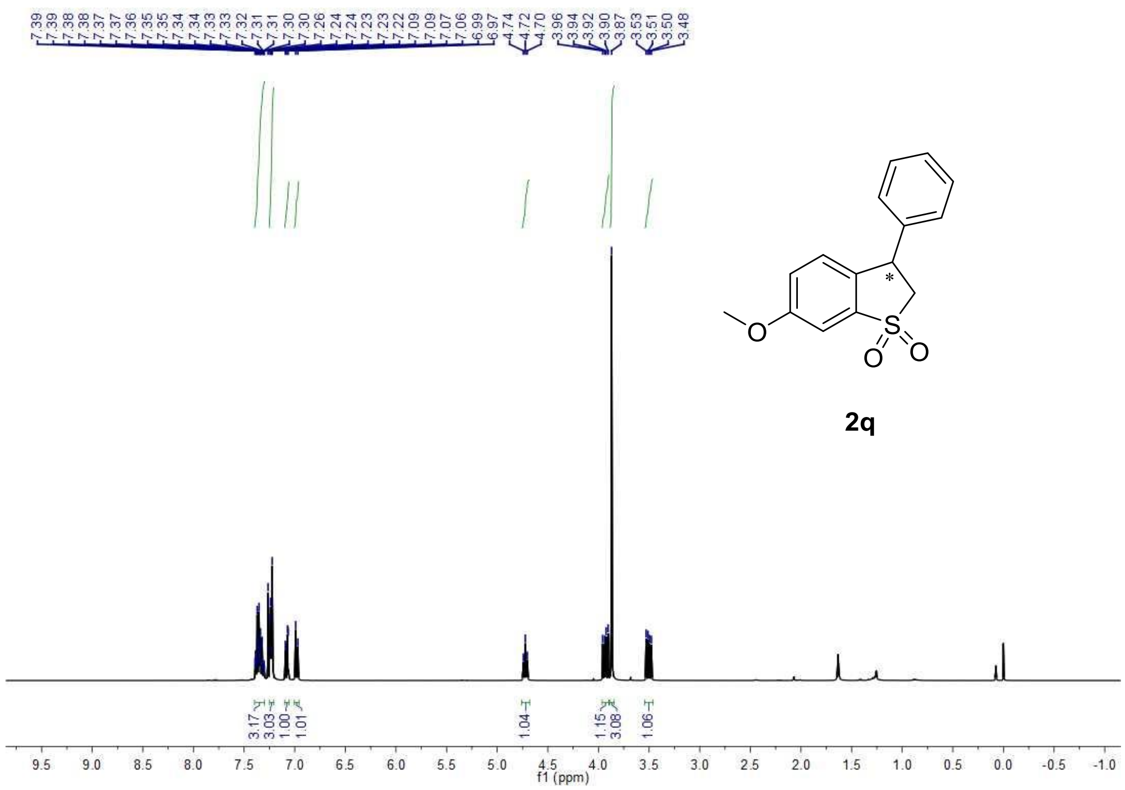
7.39 7.39 7.38 7.38 7.37 7.37 7.36 7.35 7.35 7.34 7.34 7.33 7.33 7.32 7.31 7.31 7.30 7.30 7.26 7.24 7.24 7.23 7.23 7.22 7.09 7.09 7.07 7.06 6.99 6.97 4.74 4.72 4.70 3.96 3.94 3.92 3.90 3.87 3.53 3.51 3.50 3.48
3.17 3.03 1.00 1.01 1.04 1.15 3.08 1.06
2q
f1 (ppm)

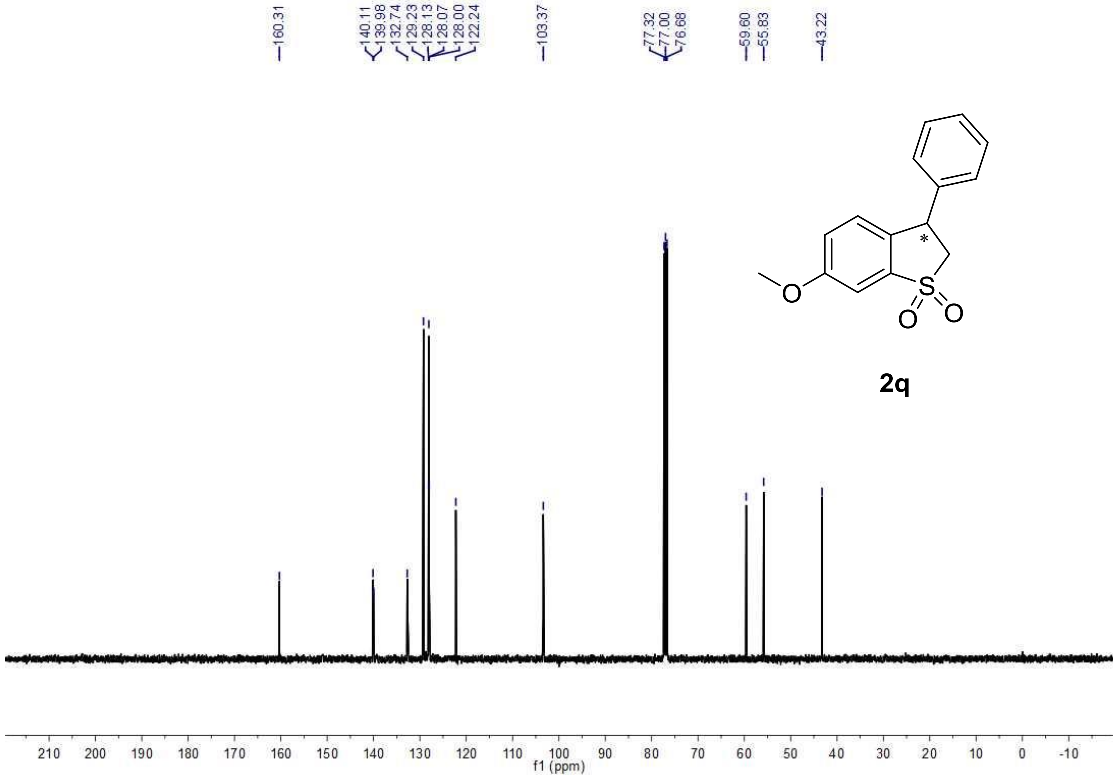
160.31
140.11 139.98 132.74 129.23 128.13 128.07 128.00 122.24
103.37
77.32 77.00 76.68
59.60 55.83
43.22
2q
f1 (ppm)

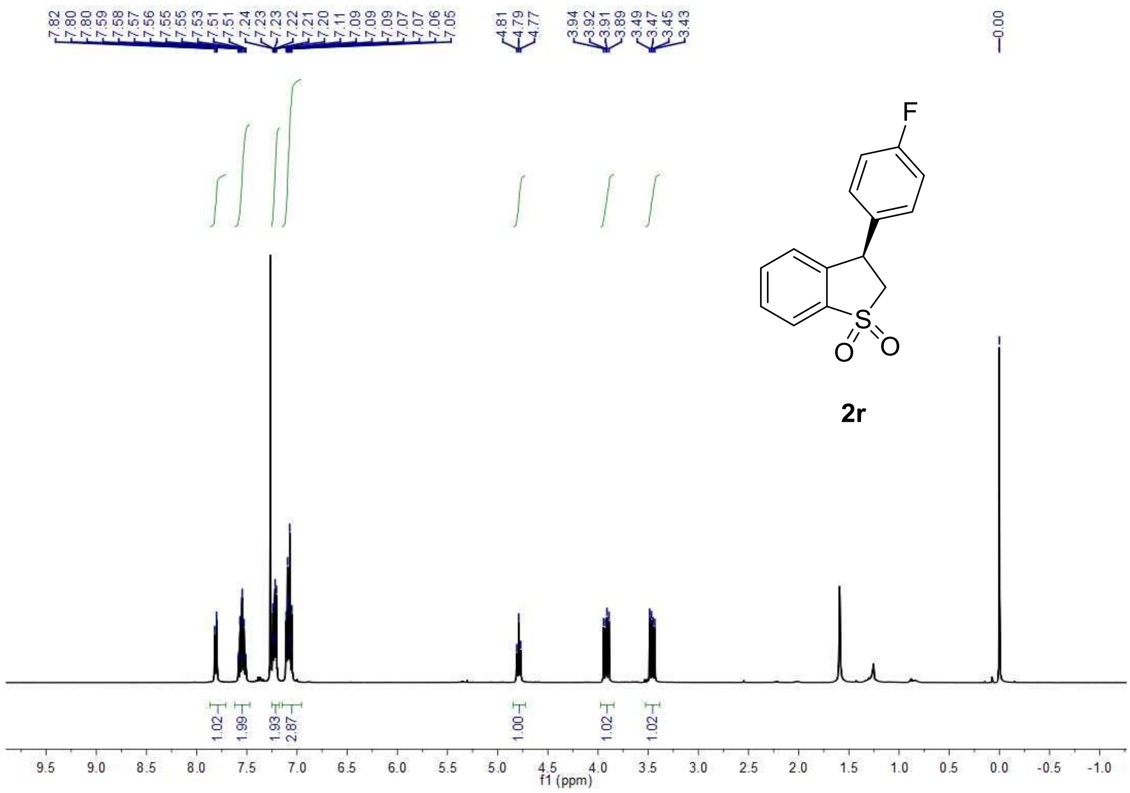
7.82
7.80
7.80
7.59
7.58
7.57
7.56
7.55
7.55
7.53
7.51
7.51
7.24
7.23
7.23
7.22
7.21
7.20
7.11
7.09
7.09
7.09
7.07
7.07
7.06
7.05
4.81
4.79
4.77
3.94
3.92
3.91
3.89
3.49
3.47
3.45
3.43
0.00
F
S
O
O
1.02
1.99
1.93
2.87
1.00
1.02
1.02
f1 (ppm)

2r

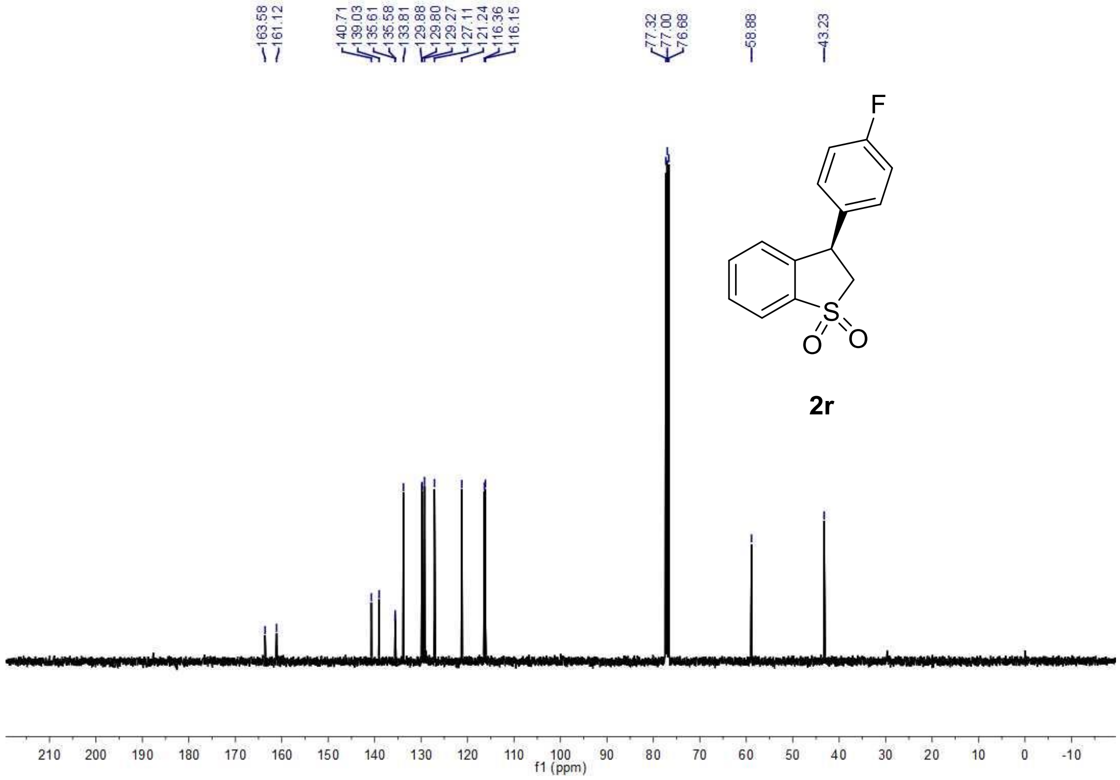
163.58
161.12
140.71
139.03
135.61
135.58
133.81
129.88
129.80
129.27
127.11
121.24
116.36
116.15
77.32
77.00
76.68
58.88
43.23
F
S
O
O
f1 (ppm)

2r

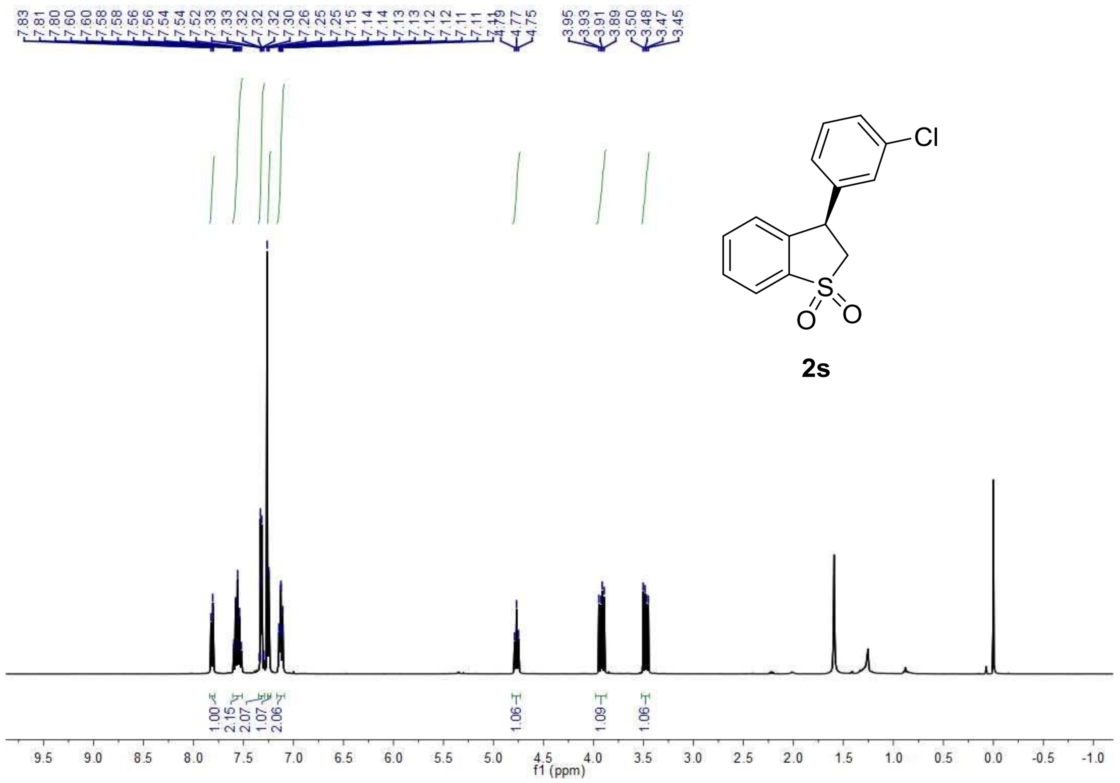
7.83 7.81 7.80 7.60 7.58 7.58 7.56 7.56 7.54 7.54 7.52 7.33 7.32 7.32 7.32 7.30 7.26 7.25 7.25 7.15 7.14 7.14 7.13 7.13 7.12 7.11 7.11 7.11 4.79 4.77 4.75
3.95 3.93 3.91 3.89 3.50 3.48 3.47 3.45
Cl
S
O
O
2s
1.00 2.15 2.07 1.07 2.06 1.06 1.09 1.06
f1 (ppm)

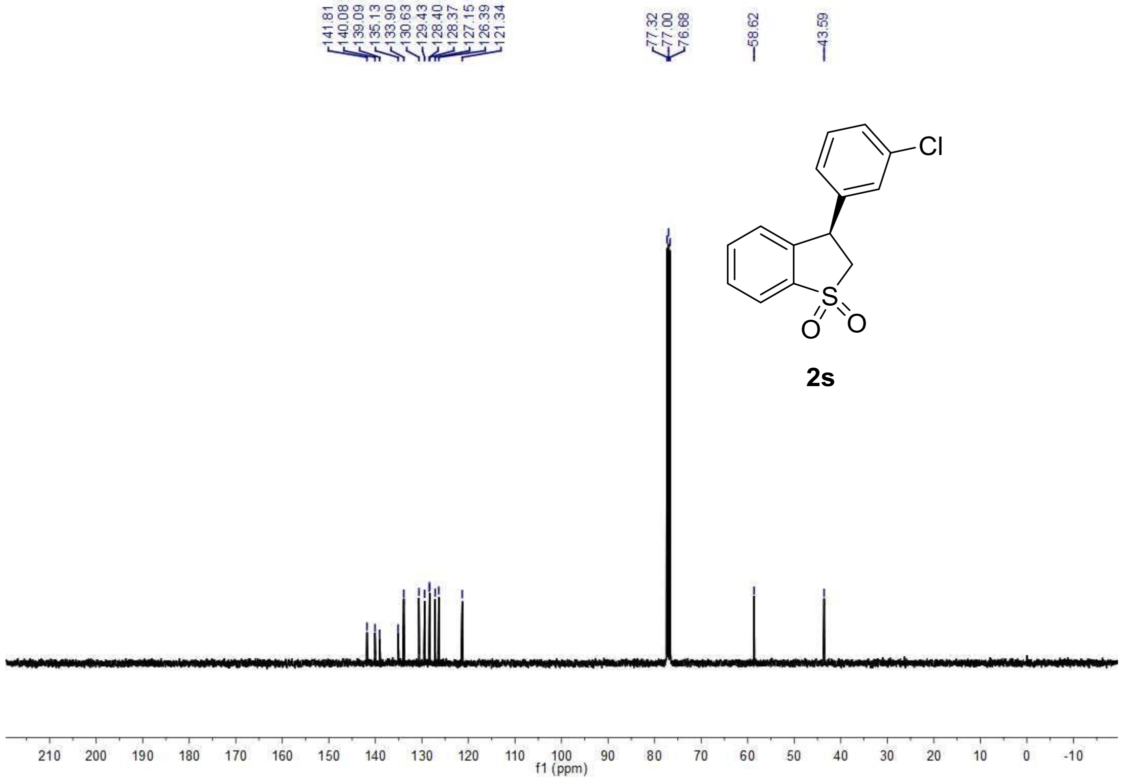
141.81 140.08 139.09 135.13 133.90 130.63 129.43 128.40 128.37 127.15 126.39 121.34
77.32 77.00 76.68
58.62
43.59
Cl
S
O
O
2s
f1 (ppm)

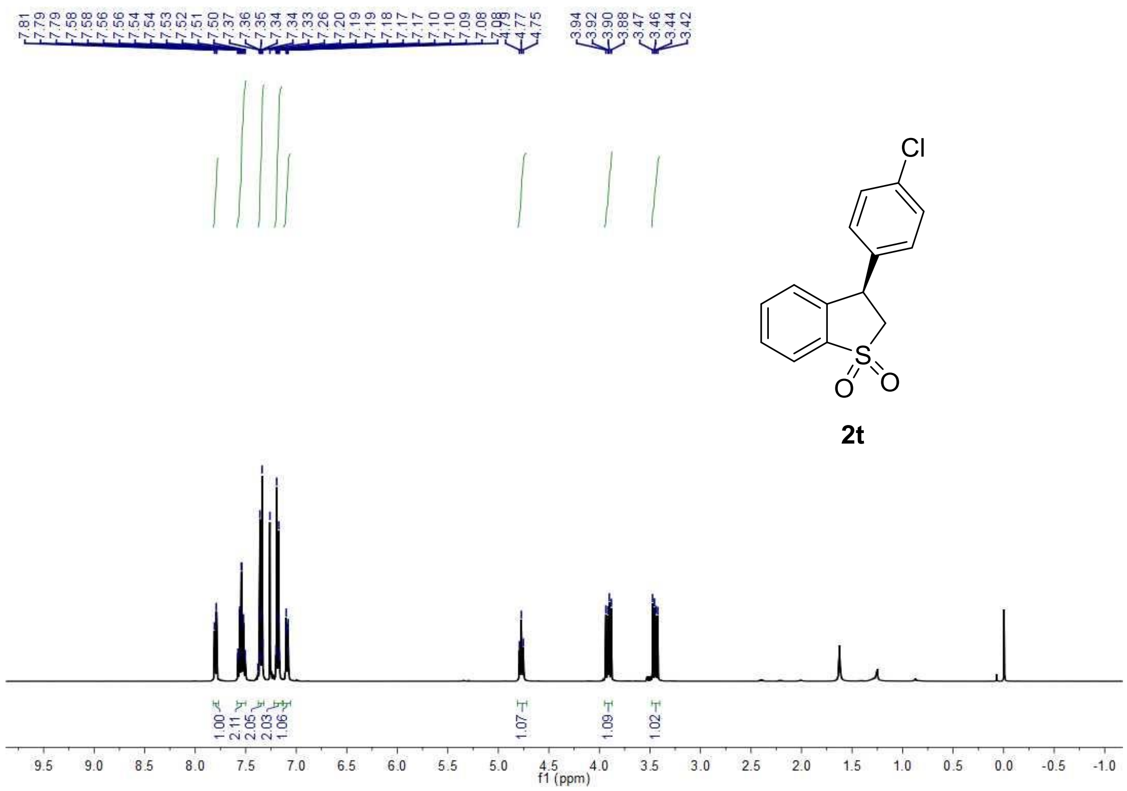
7.81 7.79 7.79 7.58 7.58 7.56 7.54 7.54 7.53 7.52 7.51 7.50 7.37 7.36 7.35 7.34 7.34 7.33 7.26 7.20 7.19 7.19 7.18 7.17 7.17 7.10 7.10 7.09 7.08 4.79 4.77 4.75
3.94 3.92 3.90 3.88 3.47 3.46 3.44 3.42
Cl
S
O O
2t
1.00 2.11 2.05 2.03 1.06 1.07 1.09 1.02
9.5 9.0 8.5 8.0 7.5 7.0 6.5 6.0 5.5 5.0 4.5 4.0 3.5 3.0 2.5 2.0 1.5 1.0 0.5 0.0 -0.5 -1.0
f1 (ppm)

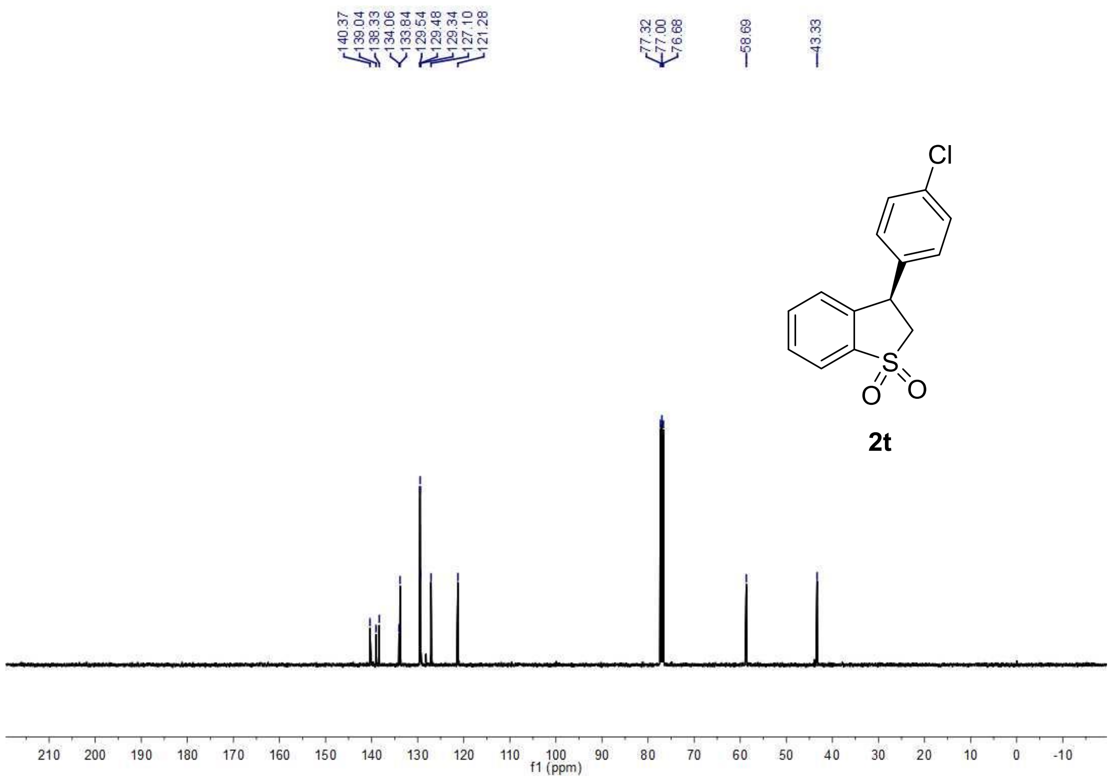
140.37 139.04 138.33 134.06 133.84 129.54 129.48 129.34 127.10 121.28
77.32 77.00 76.68
58.69
43.33
Cl
S
O O
2t
210 200 190 180 170 160 150 140 130 120 110 100 90 80 70 60 50 40 30 20 10 0 -10
f1 (ppm)

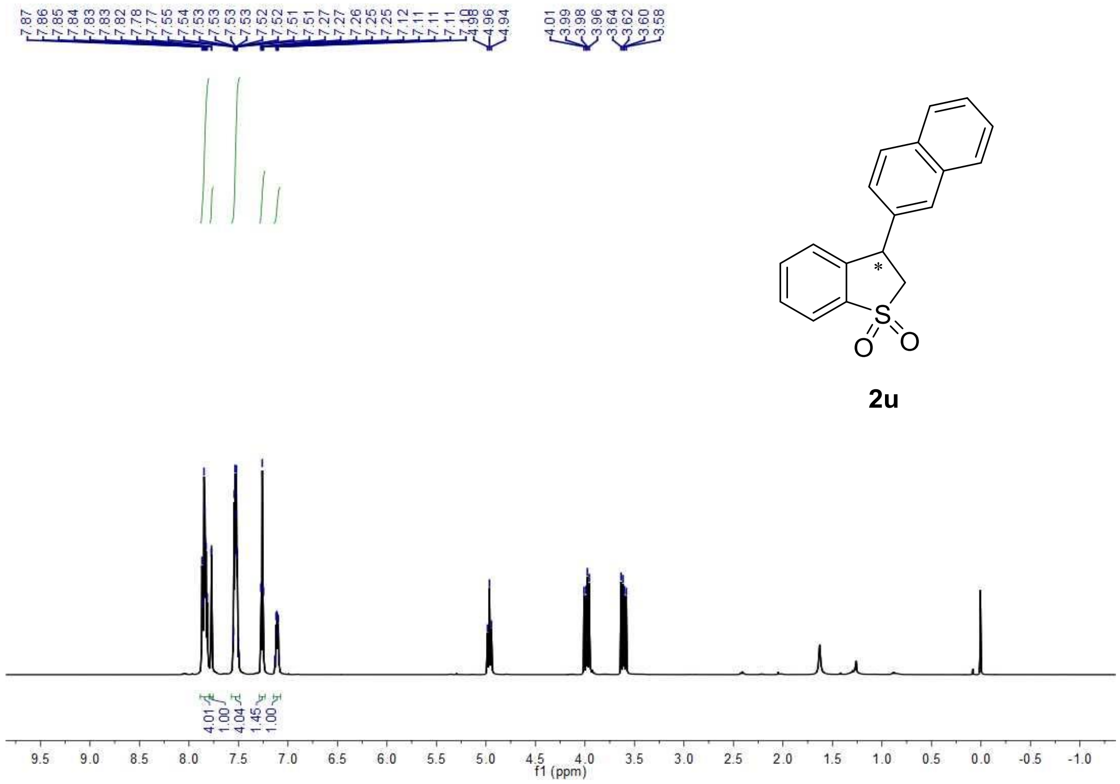
7.87 7.86 7.85 7.84 7.83 7.83 7.82 7.78 7.77 7.55 7.54 7.53 7.53 7.53 7.53 7.52 7.52 7.51 7.27 7.27 7.26 7.25 7.25 7.12 7.11 7.11 7.11 7.10 4.98 4.96 4.94
4.01 3.99 3.98 3.96 3.64 3.62 3.60 3.58
S
O
O
*
2u
4.01 1.00 4.04 1.45 1.00
9.5 9.0 8.5 8.0 7.5 7.0 6.5 6.0 5.5 5.0 4.5 4.0 3.5 3.0 2.5 2.0 1.5 1.0 0.5 0.0 -0.5 -1.0
f1 (ppm)

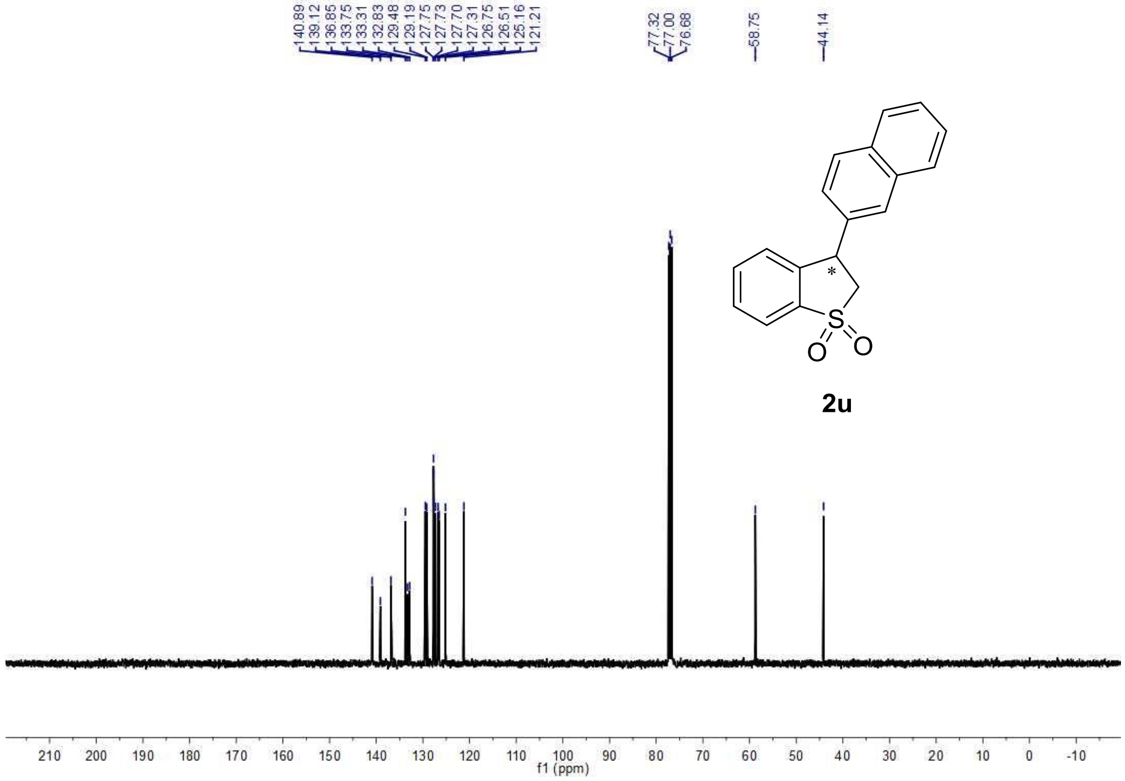
140.89 139.12 136.85 133.75 133.31 132.83 129.48 129.19 127.75 127.73 127.70 127.31 126.75 126.51 125.16 121.21
77.32 77.00 76.68
58.75
44.14
S
O
O
*
2u
210 200 190 180 170 160 150 140 130 120 110 100 90 80 70 60 50 40 30 20 10 0 -10
f1 (ppm)

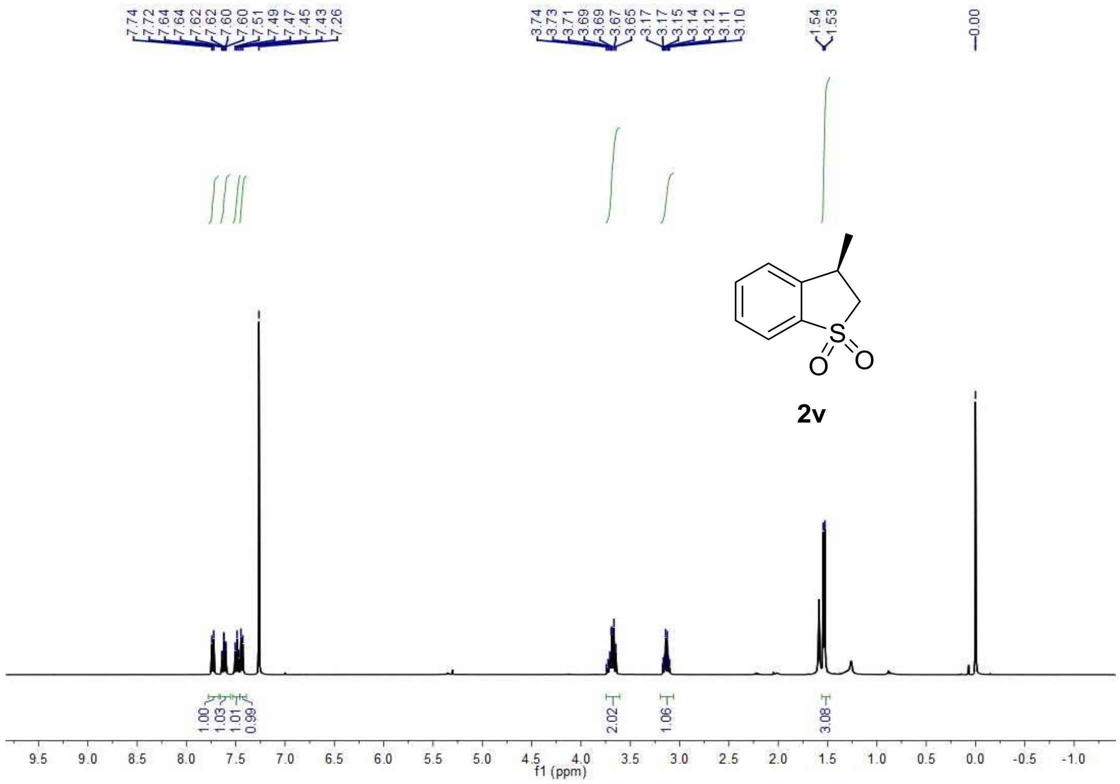
7.74
7.72
7.64
7.62
7.62
7.60
7.51
7.49
7.47
7.45
7.43
7.26
3.74
3.73
3.71
3.69
3.69
3.67
3.65
3.17
3.17
3.15
3.14
3.12
3.11
3.10
1.54
1.53
0.00
2v
1.00
1.03
1.01
0.99
2.02
1.06
3.08
f1 (ppm)

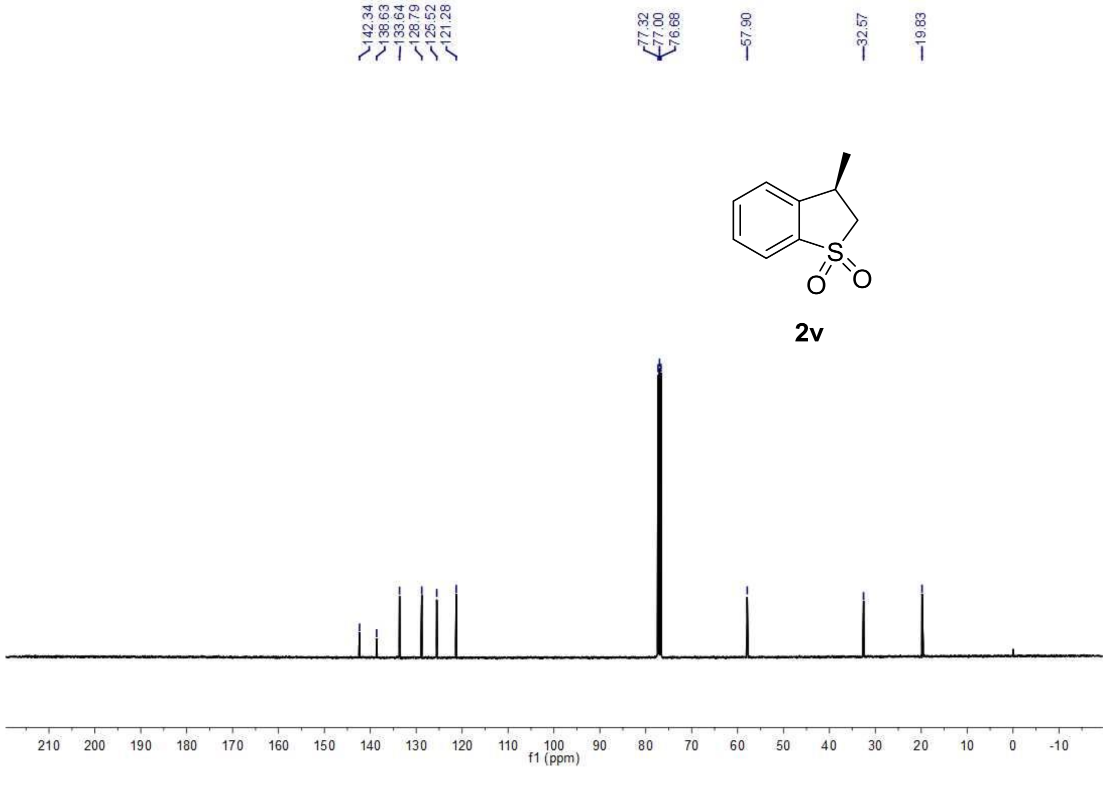
142.34
138.63
133.64
128.79
125.52
121.28
77.32
77.00
76.68
57.90
32.57
19.83
2v
f1 (ppm)

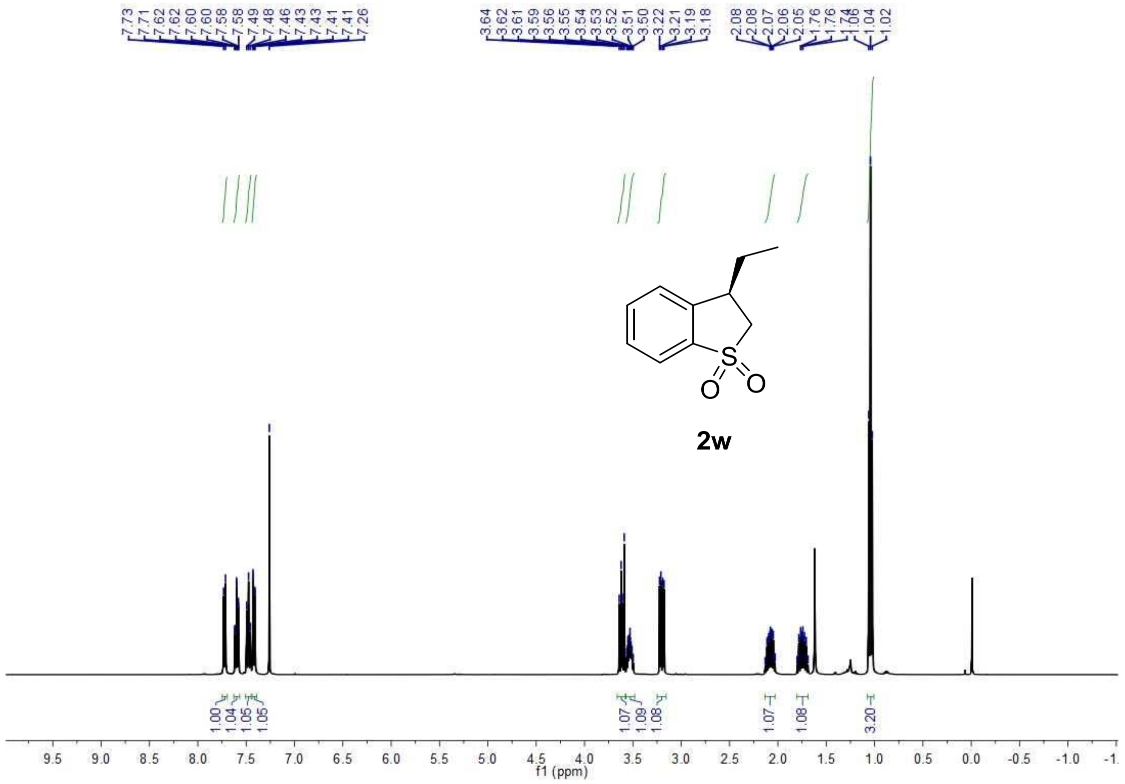
7.73
7.71
7.62
7.60
7.60
7.58
7.58
7.49
7.48
7.46
7.43
7.43
7.41
7.26
3.64
3.62
3.61
3.59
3.56
3.55
3.54
3.53
3.52
3.51
3.50
3.22
3.21
3.19
3.18
2.08
2.08
2.07
2.06
2.05
1.76
1.76
1.74
1.06
1.04
1.02
2w
1.00
1.04
1.05
1.05
1.07
1.09
1.08
1.07
1.08
3.20
f1 (ppm)

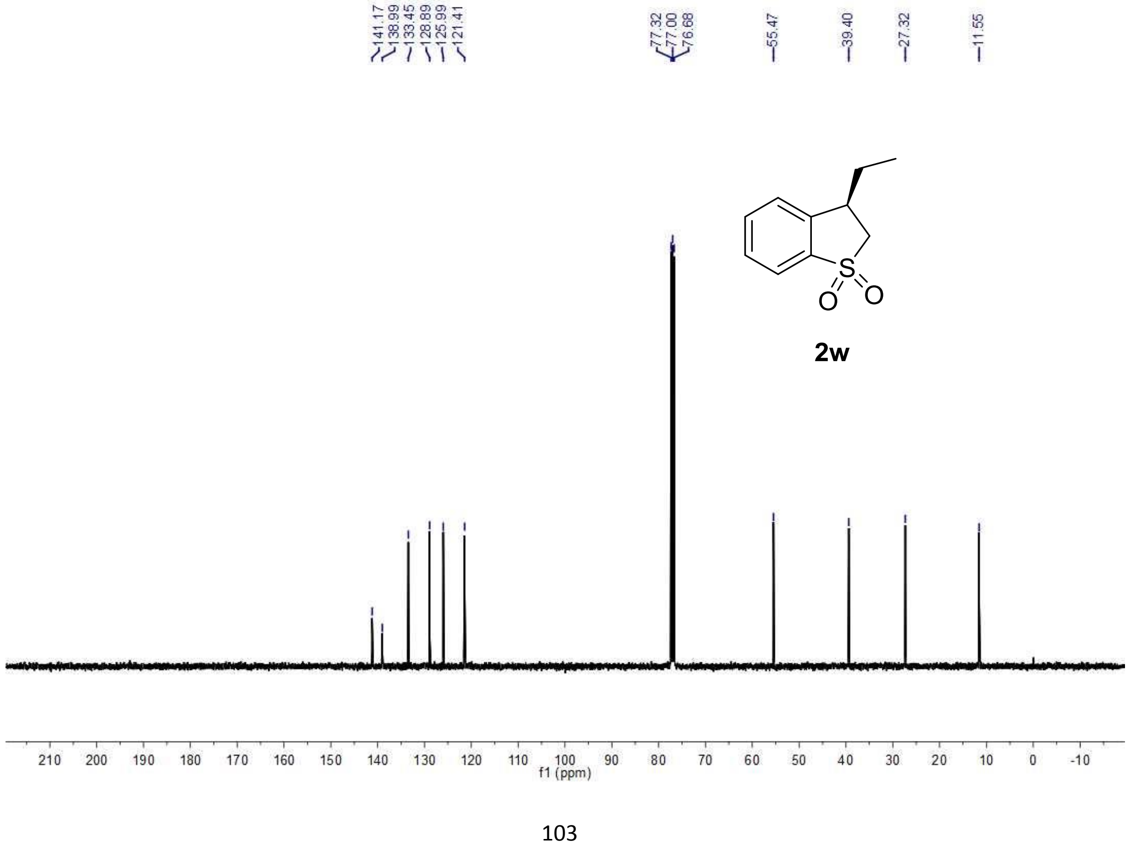
141.17
138.99
133.45
128.89
125.99
121.41
77.32
77.00
76.68
55.47
39.40
27.32
11.55
2w
f1 (ppm)

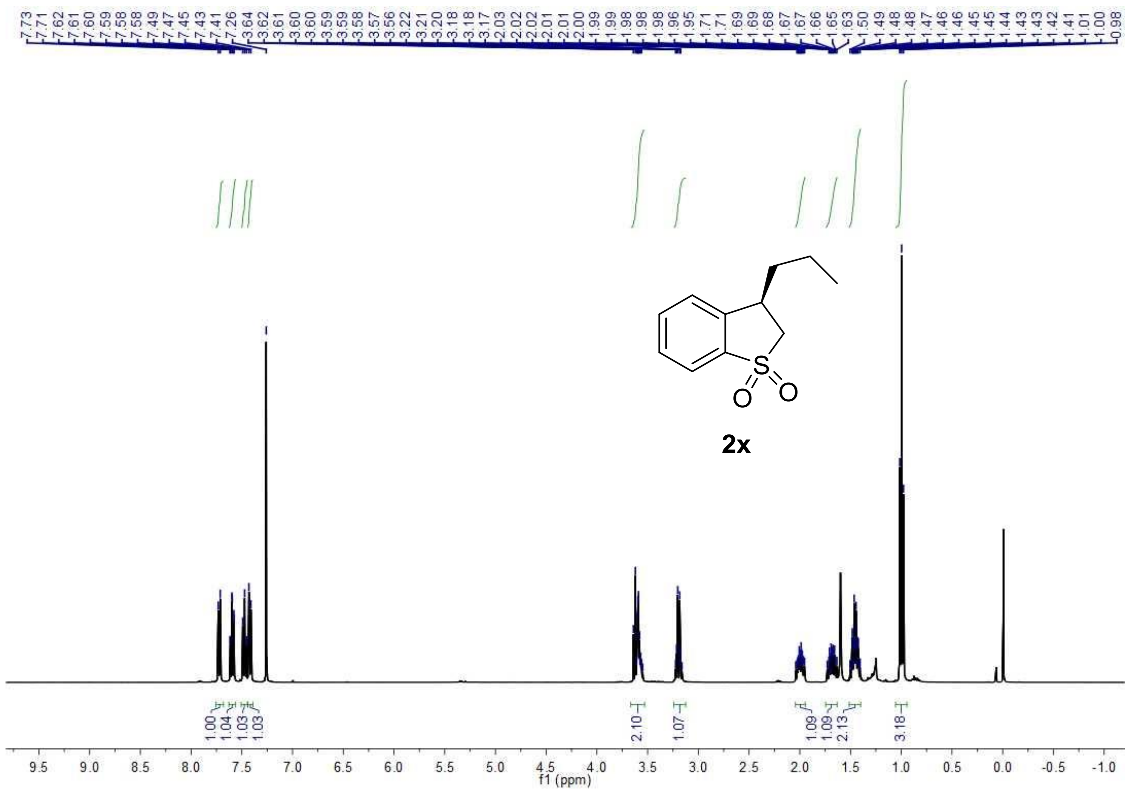
2x
f1 (ppm)

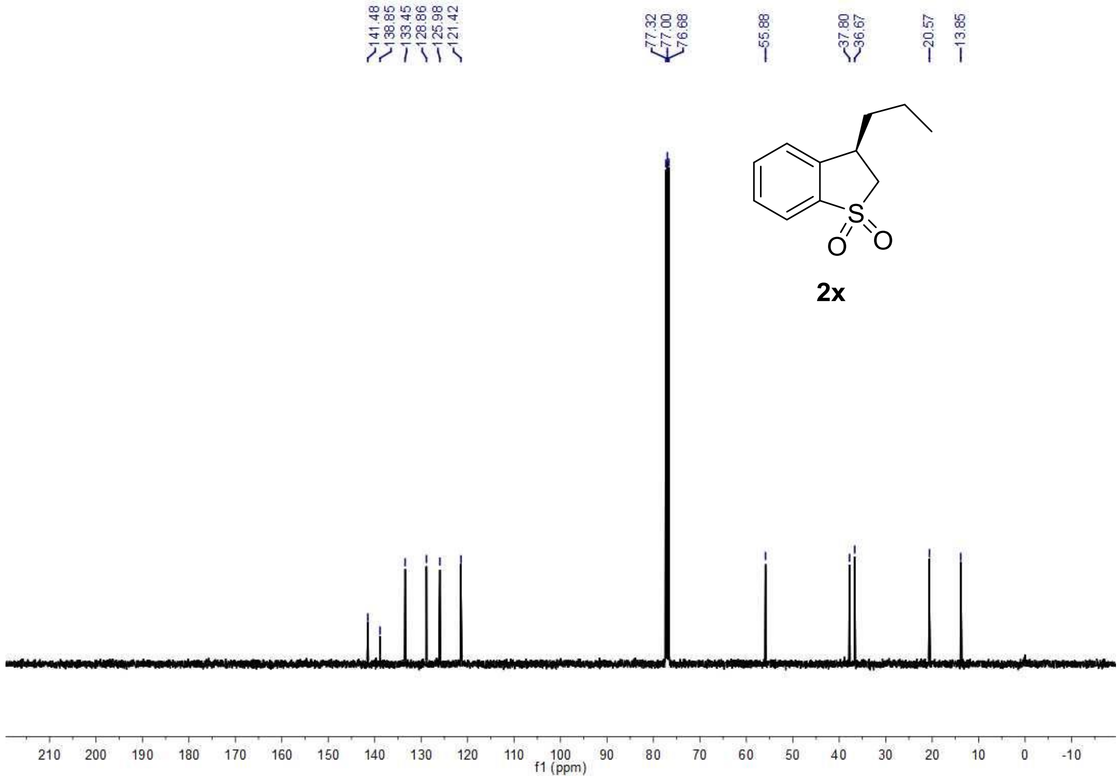
2x
f1 (ppm)

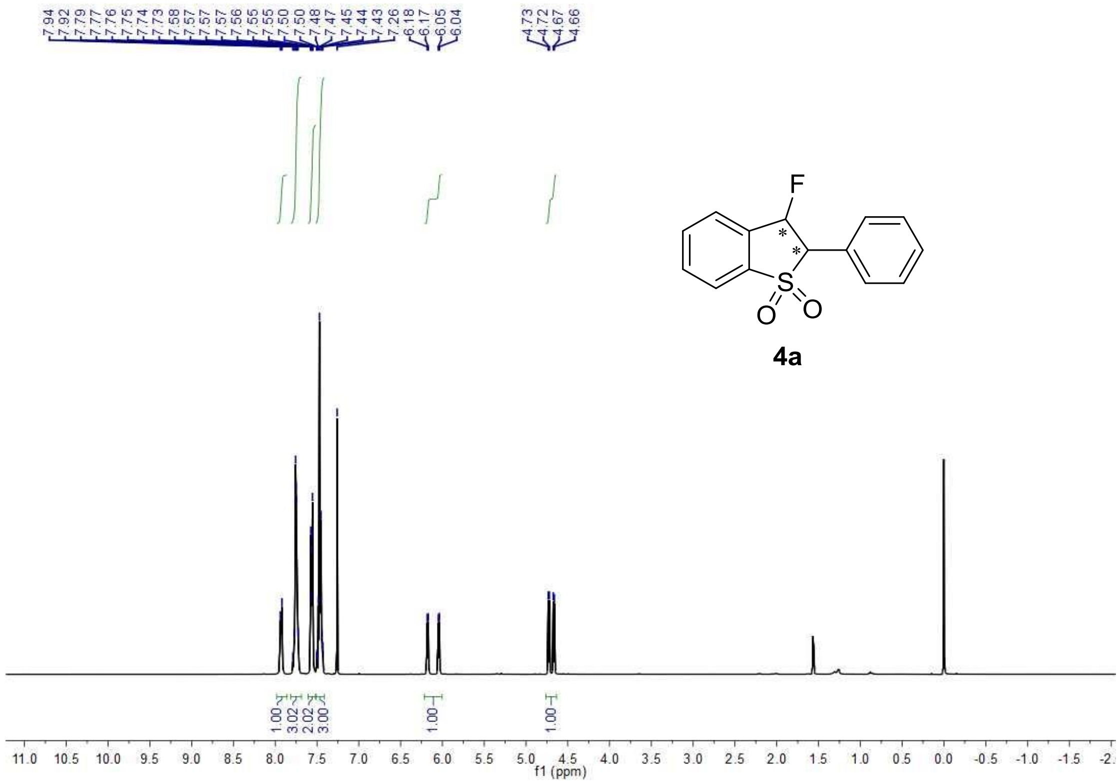
7.94 7.92 7.79 7.77 7.76 7.75 7.74 7.73 7.58 7.57 7.57 7.56 7.55 7.55 7.50 7.50 7.48 7.47 7.45 7.44 7.43 7.26 6.18 6.17 6.05 6.04
4.73 4.72 4.67 4.66
F
S
O
O
4a
1.00 3.02 2.02 3.00
1.00
1.00
f1 (ppm)

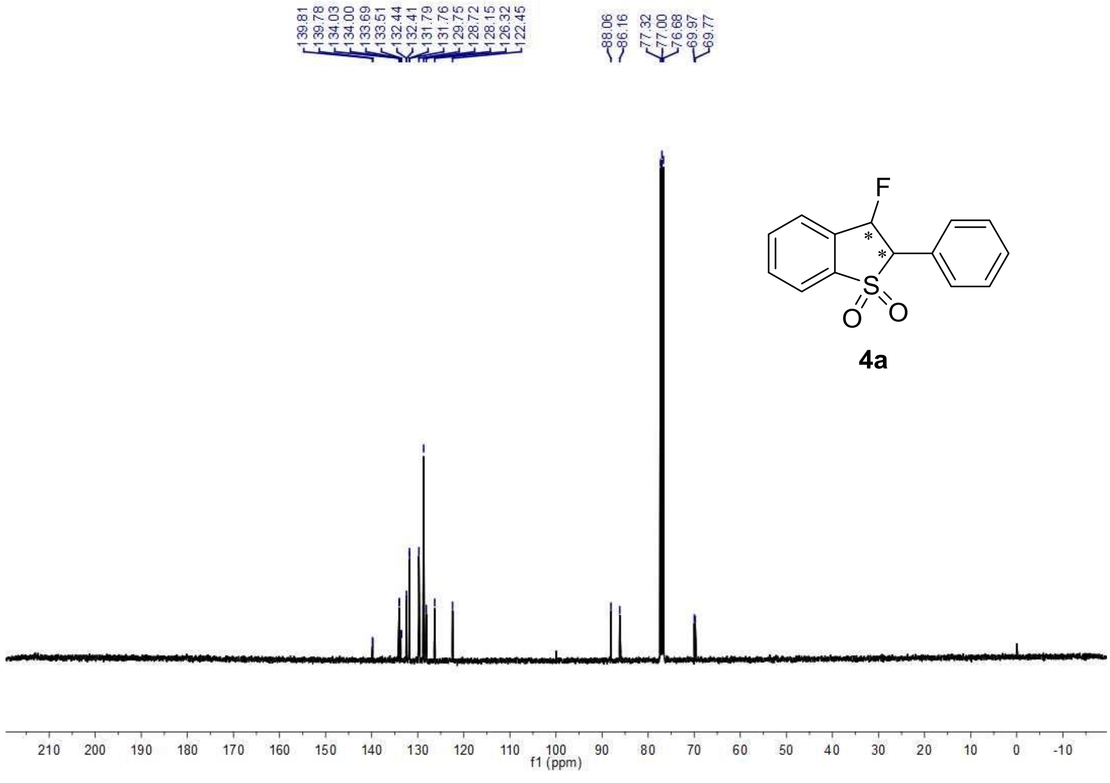
139.81 139.78 134.03 134.00 133.69 133.51 132.44 132.41 131.79 131.76 129.75 128.72 128.15 126.32 122.45
88.06 86.16 77.32 77.00 76.68 69.97 69.77
F
S
O
O
4a
f1 (ppm)

9. HPLC spectra

HPLC of racemic-**2a**

Data File D:\DATA\LGY\LGY-1-43\LGY-1-43 2018-09-11 14-49-28\011-0301.D
Sample Name: LGY-1-43-1

===

Acq. Operator : Seq. Line : 3
Acq. Instrument : Instrument 1 Location : Vial 11
Injection Date : 9/11/2018 3:15:08 PM Inj : 1
Inj Volume : 5.000 µl
Acq. Method : D:\DATA\LGY\LGY-1-43\LGY-1-43 2018-09-11 14-49-28\VWD-AS(1-6)-80-20-1ML-5UL-220NM-80MIN.M
Last changed : 9/11/2018 4:37:46 PM (modified after loading)
Analysis Method : D:\METHOD\LG\VWD-AD(1-2)-99-1-0.2ML-2UL-220NM-10MIN.M
Last changed : 9/12/2018 9:50:51 AM (modified after loading)
Additional Info : Peak(s) manually integrated

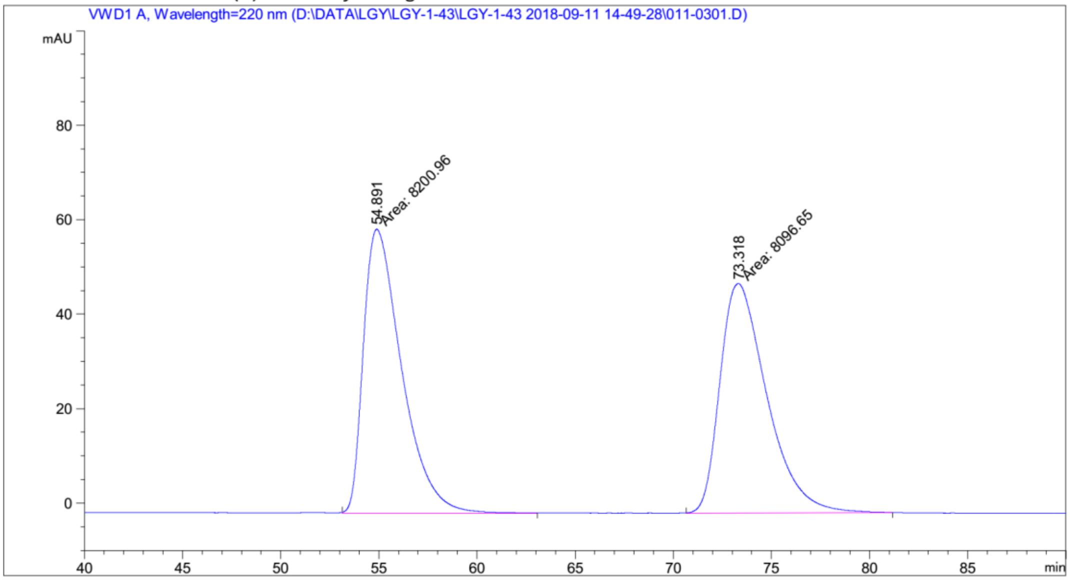

===

Area Percent Report

===

Sorted By : Retention Time
Multiplier : 1.0000
Dilution : 1.0000
Use Multiplier & Dilution Factor with ISTDs

Signal 1: VWD1 A, Wavelength=220 nm

Peak #	RetTime [min]	Sig	Type	Area [mAU*s]	Height [mAU]	Area %
1	54.891	1	MM	8200.96387	60.20780	50.3200
2	73.318	1	MP	8096.65479	48.65597	49.6800

Totals : 1.62976e4 108.86377

HPLC of **2a**

Data File E:\DATA\LGY\LGY-1-106\LGY-1-106 2018-02-03 16-42-19\023-0501.D
Sample Name: LGY-1-106-3

===

Acq. Operator : SYSTEM   Seq. Line : 5
Acq. Instrument : 1260HPLC-VWD   Location : Vial 23
Injection Date : 2/3/2018 8:47:02 PM   Inj : 1
Inj Volume : 5.000 µl
Acq. Method : E:\DATA\LGY\LGY-1-106\LGY-1-106 2018-02-03 16-42-19\VWD-AS(1-6)-80-20-1ML-5UL-220NM-90MIN.M
Last changed : 2/3/2018 4:42:20 PM by SYSTEM
Analysis Method : E:\DATA\LGY\LGY-1-106\LGY-1-106 2018-02-03 16-42-19\VWD-AS(1-6)-80-20-1ML-5UL-220NM-90MIN.M (Sequence Method)
Last changed : 9/14/2018 3:37:19 PM by SYSTEM
(modified after loading)
Additional Info : Peak(s) manually integrated

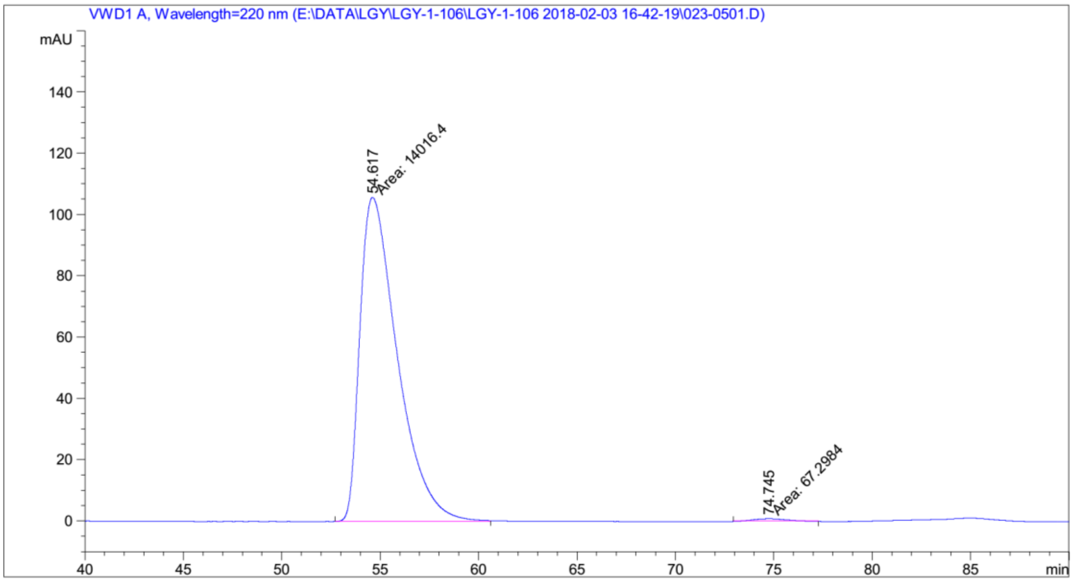

===

Area Percent Report

===

Sorted By : Signal
Multiplier : 1.0000
Dilution : 1.0000
Do not use Multiplier & Dilution Factor with ISTDs

Signal 1: VWD1 A, Wavelength=220 nm

Peak #	RetTime [min]	Type	Width [min]	Area [mAU*s]	Height [mAU]	Area %
1	54.617	MM	2.2089	1.40164e4	105.75460	99.5222
2	74.745	MM	1.6222	67.29838	6.91447e-1	0.4778

Totals : 1.40837e4 106.44605

HPLC of racemic-**2b**

```
Data File E:\DATA\LXX\YC-CN-3\CL-BR-CN-RAC-AD 2017-11-20 14-41-19\011-1501.D
Sample Name: LGY-1-30-1-RE-2

    =====================================================================
    Acq. Operator   : SYSTEM                          Seq. Line :  15
    Acq. Instrument : 1260HPLC-VWD                     Location : Vial 11
    Injection Date  : 11/21/2017 12:11:03 AM                Inj :   1
                                                     Inj Volume : 5.000 µl
    Acq. Method     : E:\DATA\LXX\YC-CN-3\CL-BR-CN-RAC-AD 2017-11-20 14-41-19\VWD-AD(1-2)-80-
                      20-1ML-5UL-220NM-120MIN.M
    Last changed    : 11/20/2017 7:15:44 PM by SYSTEM
    Analysis Method : E:\DATA\LXX\YC-CN-3\CL-BR-CN-RAC-AD 2017-11-20 14-41-19\VWD-AD(1-2)-80-
                      20-1ML-5UL-220NM-120MIN.M (Sequence Method)
    Last changed    : 4/13/2018 10:38:59 AM by SYSTEM
                      (modified after loading)
    Additional Info : Peak(s) manually integrated
```

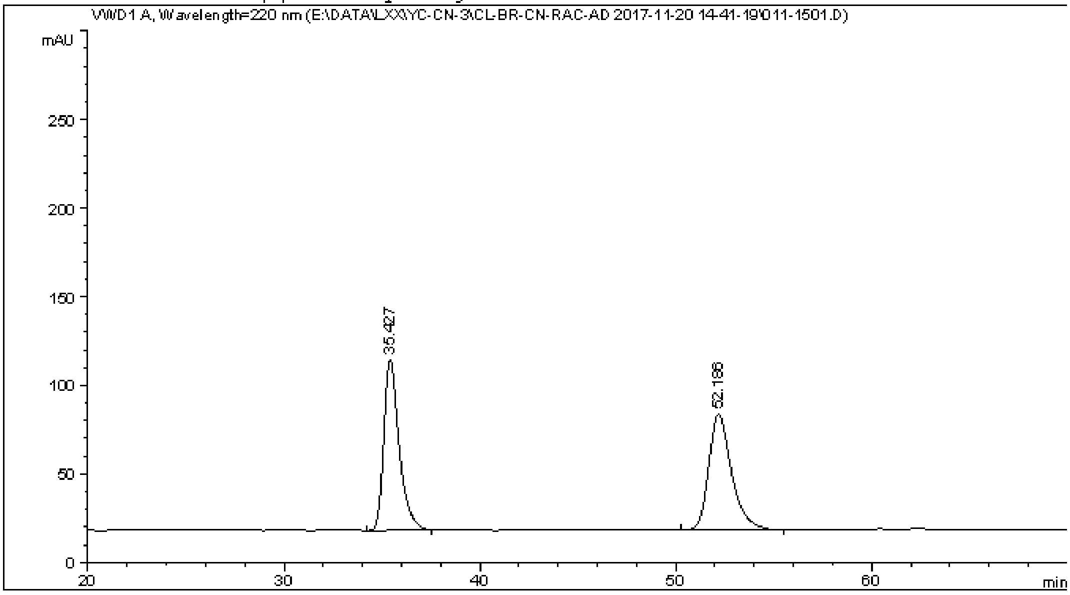


```
    =====================================================================
                             Area Percent Report
    =====================================================================

    Sorted By             :      Signal
    Multiplier            :      1.0000
    Dilution              :      1.0000
    Do not use Multiplier & Dilution Factor with ISTDs


    Signal 1: VWD1 A, Wavelength=220 nm

    Peak RetTime Type  Width     Area      Height     Area
      #   [min]        [min]   [mAU*s]     [mAU]        %
    ----|-------|----|-------|----------|----------|--------|
       1  35.427 BB    0.8183 5244.05957   96.45979  49.9569
       2  52.186 BB    1.2062 5253.11182   65.18310  50.0431

    Totals :                  1.04972e4   161.64289


    =====================================================================
                              *** End of Report ***

1260HPLC-VWD 4/13/2018 10:39:03 AM SYSTEM                             Page 1 of 1
```

HPLC of **2b**

```
Data File E:\DATA\LGY\LGY-1-95\LGY-1-95 2018-01-11 09-00-29\046-2101.D
Sample Name: LGY-1-95-6

    =====================================================================
    Acq. Operator   : SYSTEM                          Seq. Line :  21
    Acq. Instrument : 1260HPLC-VWD                     Location : Vial 46
    Injection Date  : 1/11/2018 10:48:07 PM                 Inj :   1
                                                     Inj Volume : 5.000 µl
    Acq. Method     : E:\DATA\LGY\LGY-1-95\LGY-1-95 2018-01-11 09-00-29\VWD-AD(1-2)-80-20-1ML-
                      5UL-220NM-70MIN.M
    Last changed    : 1/11/2018 9:32:22 AM by SYSTEM
    Analysis Method : E:\DATA\LGY\LGY-1-95\LGY-1-95 2018-01-11 09-00-29\VWD-AD(1-2)-80-20-1ML-
                      5UL-220NM-70MIN.M (Sequence Method)
    Last changed    : 4/13/2018 11:11:59 AM by SYSTEM
                      (modified after loading)
    Additional Info : Peak(s) manually integrated
```

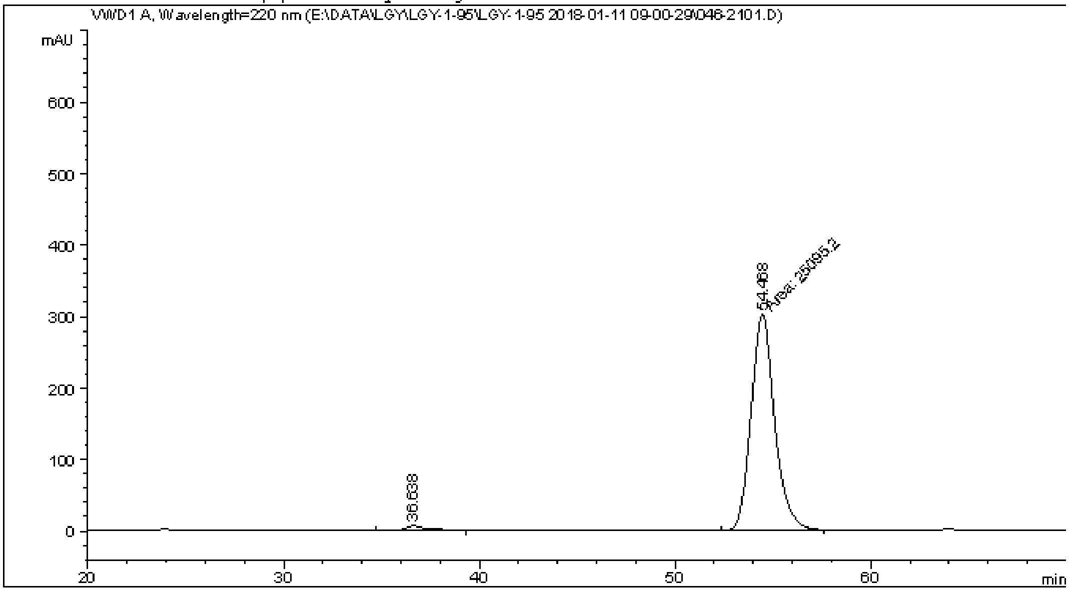


```
    =====================================================================
                             Area Percent Report
    =====================================================================

    Sorted By             :      Signal
    Multiplier            :      1.0000
    Dilution              :      1.0000
    Do not use Multiplier & Dilution Factor with ISTDs


    Signal 1: VWD1 A, Wavelength=220 nm

    Peak RetTime Type  Width     Area      Height     Area
      #   [min]        [min]   [mAU*s]     [mAU]        %
    ----|-------|----|-------|----------|----------|--------|
       1  36.638 BB    0.9879  444.26672    6.31377   1.7395
       2  54.468 MM    1.3885 2.50952e4  301.22711  98.2605

    Totals :                  2.55395e4  307.54088


    =====================================================================
                             *** End of Report ***
```

1260HPLC-VWD 4/13/2018 11:12:06 AM SYSTEM Page 1 of 1

HPLC of racemic-**2c**

Data File E:\DATA\LGY\LGY-1-30\LGY-1-30 2017-11-18 09-16-49\002-0301.D
Sample Name: LGY-1-30-2-RE

===
Acq. Operator : SYSTEM Seq. Line : 3
Acq. Instrument : 1260HPLC-VWD Location : Vial 2
Injection Date : 11/18/2017 11:29:08 AM Inj : 1
Inj Volume : 5.000 µl
Acq. Method : E:\DATA\LGY\LGY-1-30\LGY-1-30 2017-11-18 09-16-49\VWD-AD1-2)-90-10-1ML-5UL-220NM-120MIN.M
Last changed : 11/18/2017 9:16:49 AM by SYSTEM
Analysis Method : E:\DATA\LGY\LGY-1-30\LGY-1-30 2017-11-18 09-16-49\VWD-AD1-2)-90-10-1ML-5UL-220NM-120MIN.M (Sequence Method)
Last changed : 4/13/2018 11:15:55 AM by SYSTEM
(modified after loading)
Additional Info : Peak(s) manually integrated

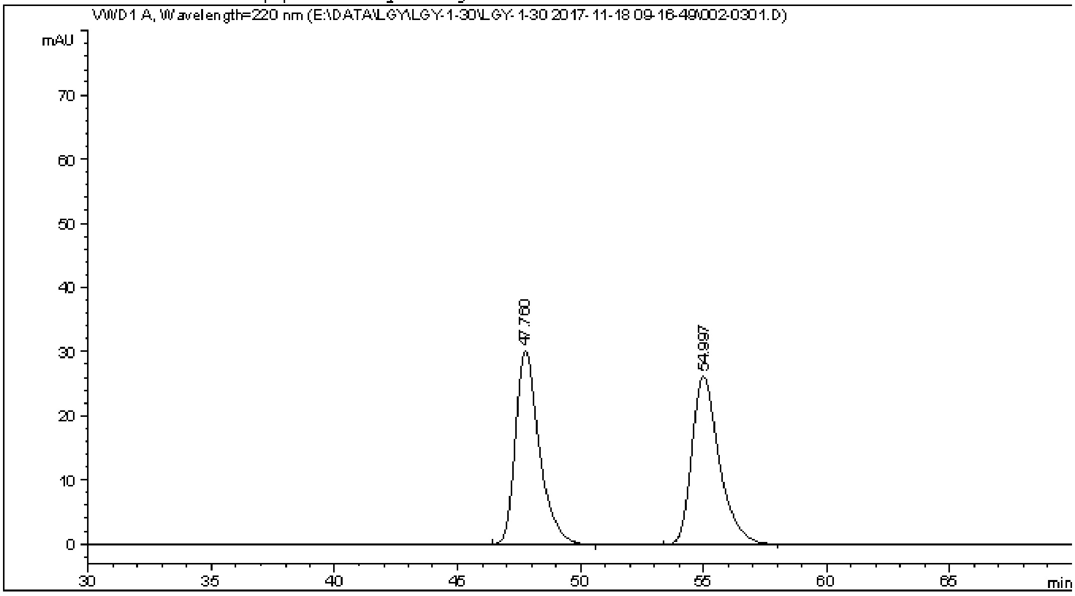

===
Area Percent Report
===

Sorted By : Signal
Multiplier : 1.0000
Dilution : 1.0000
Do not use Multiplier & Dilution Factor with ISTDs

Signal 1: VWD1 A, Wavelength=220 nm

Peak #	RetTime [min]	Type	Width [min]	Area [mAU*s]	Height [mAU]	Area %
1	47.760	BB	1.0081	2028.27917	30.18014	49.8963
2	54.997	BB	1.1583	2036.71033	26.21724	50.1037
Totals :				4064.98950	56.39738	

===
*** End of Report ***

1260HPLC-VWD 4/13/2018 11:17:17 AM SYSTEM Page 1 of 1

HPLC of **2c**

Data File E:\DATA\LGY\LGY-1-95\LGY-1-95 2018-01-11 09-00-29\051-2301.D
Sample Name: LGY-1-95-7

```
=====================================================================
Acq. Operator   : SYSTEM                          Seq. Line :  23
Acq. Instrument : 1260HPLC-VWD                     Location : Vial 51
Injection Date  : 1/12/2018 12:09:48 AM                 Inj :   1
                                                 Inj Volume : 5.000 µl
Acq. Method     : E:\DATA\LGY\LGY-1-95\LGY-1-95 2018-01-11 09-00-29\VWD-AD(1-2)-90-10-1ML-
                  5UL-220NM-70MIN.M
Last changed    : 1/11/2018 9:57:00 AM by SYSTEM
Analysis Method : E:\DATA\LGY\LGY-1-95\LGY-1-95 2018-01-11 09-00-29\VWD-AD(1-2)-90-10-1ML-
                  5UL-220NM-70MIN.M (Sequence Method)
Last changed    : 4/13/2018 11:25:57 AM by SYSTEM
                  (modified after loading)
Additional Info : Peak(s) manually integrated
```

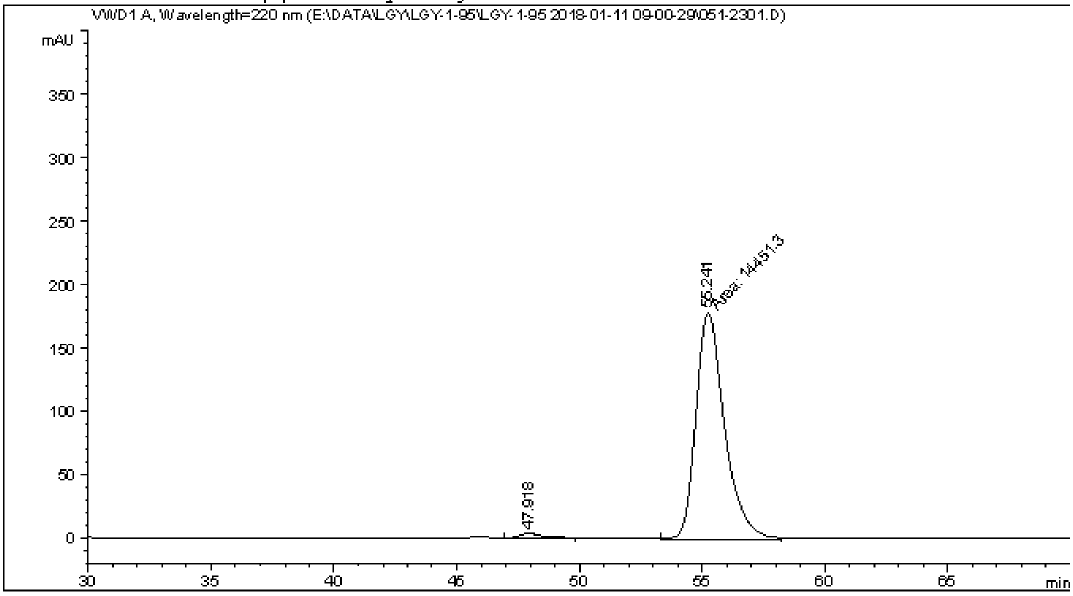


```
=====================================================================
                       Area Percent Report
=====================================================================

Sorted By             :      Signal
Multiplier            :      1.0000
Dilution              :      1.0000
Do not use Multiplier & Dilution Factor with ISTDs


Signal 1: VWD1 A, Wavelength=220 nm
```

Peak #	RetTime [min]	Type	Width [min]	Area [mAU*s]	Height [mAU]	Area %
1	47.918	BB	0.8864	201.08961	3.24308	1.3724
2	55.241	MM	1.3502	1.44513e4	178.37892	98.6276
Totals :				1.46524e4	181.62200	

```
=====================================================================
                       *** End of Report ***
```

1260HPLC-VWD 4/13/2018 11:26:05 AM SYSTEM   Page 1 of 1

HPLC of racemic-**2d**

```
Data File E:\DATA\LGY\LGY-1-48\LGY-1-48 2017-11-28 08-45-45\001-0201.D
Sample Name: LGY-1-48

    =====================================================================
    Acq. Operator   : SYSTEM                          Seq. Line :   2
    Acq. Instrument : 1260HPLC-VWD                     Location : Vial 1
    Injection Date  : 11/28/2017 8:59:18 AM                 Inj :   1
                                                     Inj Volume : 5.000 µl
    Acq. Method     : E:\DATA\LGY\LGY-1-48\LGY-1-48 2017-11-28 08-45-45\VWD-AD(1-2)-90-10-1ML-
                      5UL-220NM-60MIN.M
    Last changed    : 11/28/2017 8:45:45 AM by SYSTEM
    Analysis Method : E:\DATA\LGY\LGY-1-48\LGY-1-48 2017-11-28 08-45-45\VWD-AD(1-2)-90-10-1ML-
                      5UL-220NM-60MIN.M (Sequence Method)
    Last changed    : 4/13/2018 2:34:27 PM by SYSTEM
                      (modified after loading)
    Additional Info : Peak(s) manually integrated
```

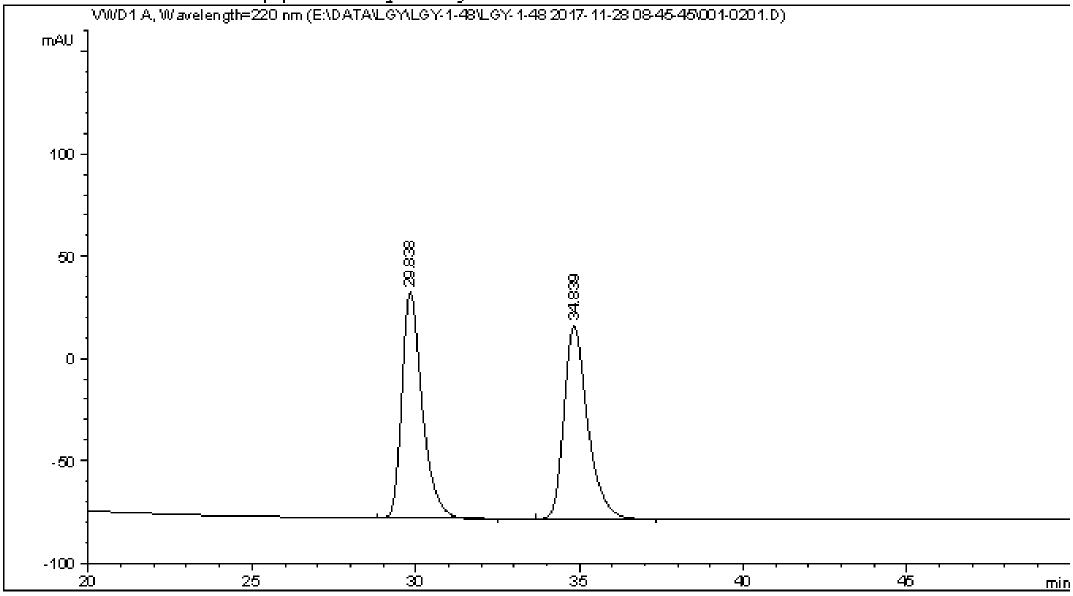


```
    =====================================================================
                             Area Percent Report
    =====================================================================

    Sorted By             :      Signal
    Multiplier            :      1.0000
    Dilution              :      1.0000
    Do not use Multiplier & Dilution Factor with ISTDs


    Signal 1: VWD1 A, Wavelength=220 nm

    Peak RetTime Type  Width     Area      Height     Area
      #   [min]        [min]   [mAU*s]     [mAU]        %
    ----|-------|----|-------|----------|----------|--------|
       1  29.838 BB    0.6488 4745.69092  110.33386  50.0321
       2  34.839 BB    0.7594 4739.59814   94.30391  49.9679

    Totals :                  9485.28906  204.63777

    =====================================================================
                              *** End of Report ***
```

1260HPLC-VWD 4/13/2018 2:34:39 PM SYSTEM   Page 1 of 1

HPLC of **2d**

Data File E:\DATA\LGY\LGY-1-95\LGY-1-95 2018-01-11 09-00-29\052-2401.D
Sample Name: LGY-1-95-8

```
=====================================================================
Acq. Operator   : SYSTEM                          Seq. Line :  24
Acq. Instrument : 1260HPLC-VWD                     Location : Vial 52
Injection Date  : 1/12/2018 1:20:36 AM                  Inj :   1
                                                 Inj Volume : 5.000 µl
Acq. Method     : E:\DATA\LGY\LGY-1-95\LGY-1-95 2018-01-11 09-00-29\VWD-AD(1-2)-90-10-1ML-
                  5UL-220NM-60MIN.M
Last changed    : 1/11/2018 9:58:22 AM by SYSTEM
Analysis Method : E:\DATA\LGY\LGY-1-95\LGY-1-95 2018-01-11 09-00-29\VWD-AD(1-2)-90-10-1ML-
                  5UL-220NM-60MIN.M (Sequence Method)
Last changed    : 4/13/2018 2:41:55 PM by SYSTEM
                  (modified after loading)
Additional Info : Peak(s) manually integrated
```

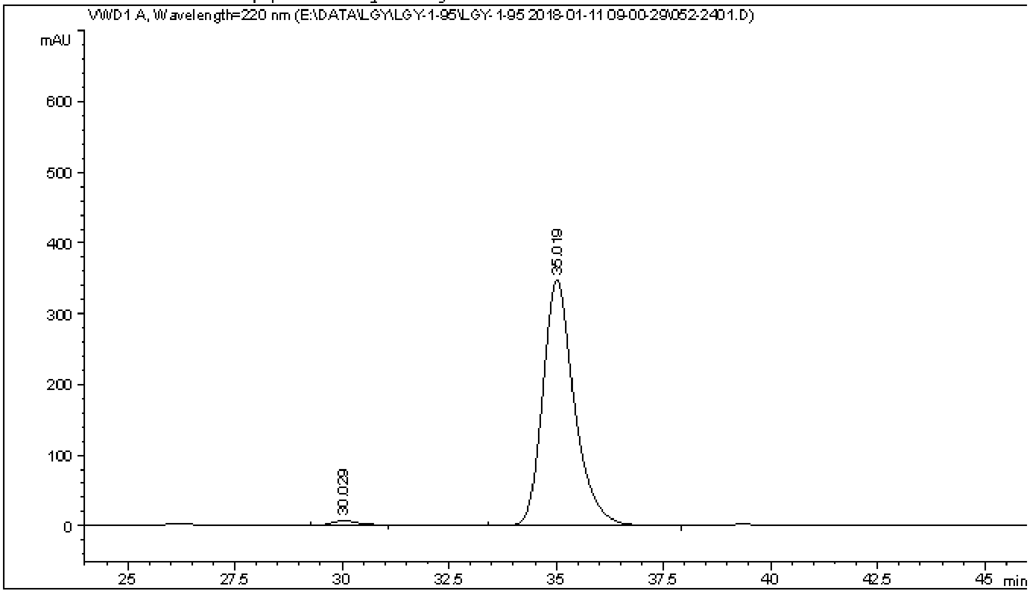


```
=====================================================================
                         Area Percent Report
=====================================================================

Sorted By             :      Signal
Multiplier            :      1.0000
Dilution              :      1.0000
Do not use Multiplier & Dilution Factor with ISTDs


Signal 1: VWD1 A, Wavelength=220 nm

Peak RetTime Type  Width     Area      Height     Area
  #   [min]        [min]   [mAU*s]     [mAU]        %
----|-------|----|-------|----------|----------|--------|
   1  30.029 BB    0.6273  273.22952    6.62337   1.5151
   2  35.019 BB    0.7703 1.77611e4   347.47638  98.4849

Totals :                  1.80343e4   354.09975

=====================================================================
                          *** End of Report ***
```

1260HPLC-VWD 4/13/2018 2:42:05 PM SYSTEM Page 1 of 1

HPLC of racemic-**2e**

Data File E:\DATA\LGY\LGY-1-43-RE\LGY-1-43-RE 2017-11-21 20-17-16\001-0201.D
Sample Name: LGY-1-43-2

===

Acq. Operator : SYSTEM Seq. Line : 2
Acq. Instrument : 1260HPLC-VWD Location : Vial 1
Injection Date : 11/21/2017 8:28:50 PM Inj : 1
Inj Volume : 5.000 µl
Acq. Method : E:\DATA\LGY\LGY-1-43-RE\LGY-1-43-RE 2017-11-21 20-17-16\VWD-AD(1-2)-90-10-1ML-5UL-220NM-80MIN.M
Last changed : 11/21/2017 8:17:16 PM by SYSTEM
Analysis Method : E:\DATA\LGY\LGY-1-43-RE\LGY-1-43-RE 2017-11-21 20-17-16\VWD-AD(1-2)-90-10-1ML-5UL-220NM-80MIN.M (Sequence Method)
Last changed : 4/13/2018 2:45:27 PM by SYSTEM
(modified after loading)
Additional Info : Peak(s) manually integrated

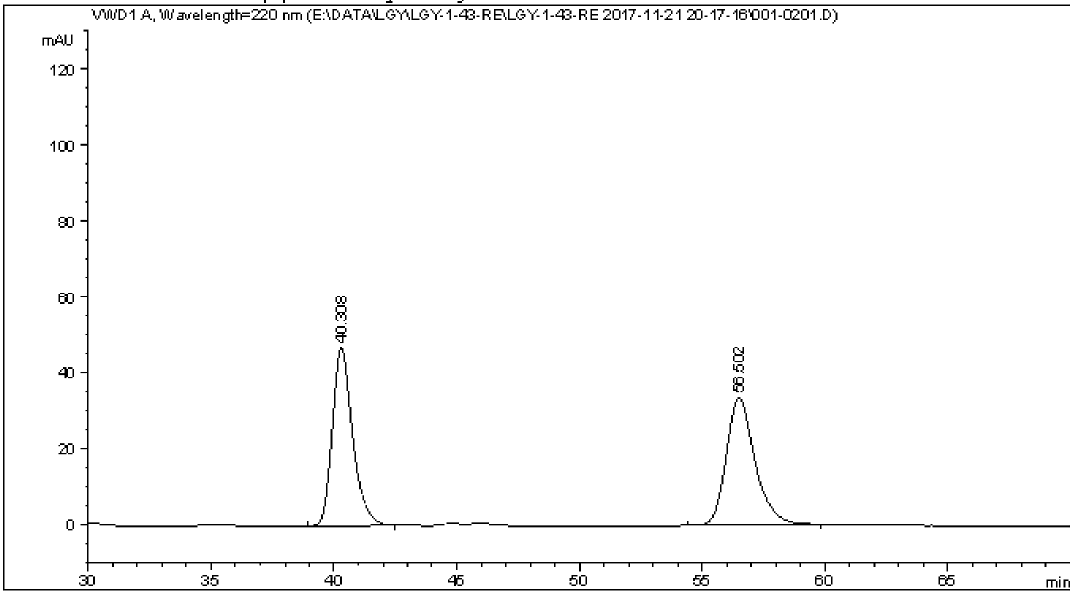

===

Area Percent Report

===

Sorted By : Signal
Multiplier : 1.0000
Dilution : 1.0000
Do not use Multiplier & Dilution Factor with ISTDs

Signal 1: VWD1 A, Wavelength=220 nm

Peak #	RetTime [min]	Type	Width [min]	Area [mAU*s]	Height [mAU]	Area %
1	40.308	BB	0.8700	2698.65698	46.85027	49.7331
2	56.502	BB	1.2107	2727.62354	33.54262	50.2669

Totals : 5426.28052 80.39289

===

*** End of Report ***

1260HPLC-VWD 4/13/2018 2:45:37 PM SYSTEM Page 1 of 1

HPLC of **2e**

Data File E:\DATA\LGY\LGY-1-95\LGY-1-95 2018-01-11 09-00-29\053-2501.D
Sample Name: LGY-1-95-9

===
Acq. Operator : SYSTEM Seq. Line : 25
Acq. Instrument : 1260HPLC-VWD Location : Vial 53
Injection Date : 1/12/2018 2:21:26 AM Inj : 1
Inj Volume : 5.000 µl
Acq. Method : E:\DATA\LGY\LGY-1-95\LGY-1-95 2018-01-11 09-00-29\VWD-AD(1-2)-90-10-1ML-5UL-220NM-70MIN.M
Last changed : 1/11/2018 9:57:00 AM by SYSTEM
Analysis Method : E:\DATA\LGY\LGY-1-95\LGY-1-95 2018-01-11 09-00-29\VWD-AD(1-2)-90-10-1ML-5UL-220NM-70MIN.M (Sequence Method)
Last changed : 4/13/2018 2:52:00 PM by SYSTEM
(modified after loading)
Additional Info : Peak(s) manually integrated

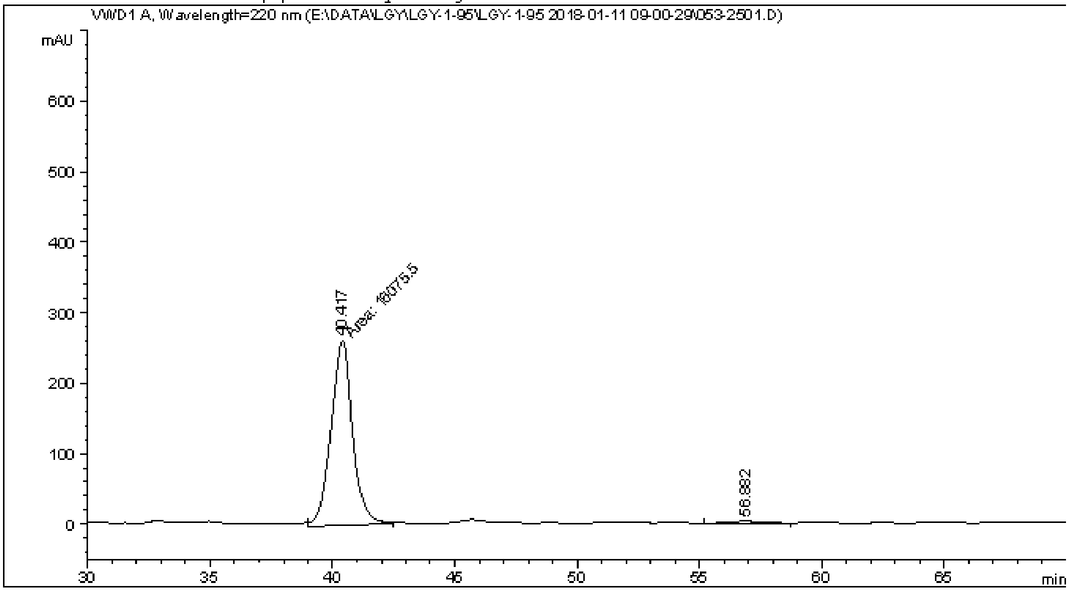

===
Area Percent Report
===

Sorted By : Signal
Multiplier : 1.0000
Dilution : 1.0000
Do not use Multiplier & Dilution Factor with ISTDs

Signal 1: VWD1 A, Wavelength=220 nm

Peak #	RetTime [min]	Type	Width [min]	Area [mAU*s]	Height [mAU]	Area %
1	40.417	MM	1.0239	1.60755e4	261.68359	98.2730
2	56.882	BB	1.1743	282.50827	2.90835	1.7270

Totals : 1.63580e4 264.59194

===
*** End of Report ***

1260HPLC-VWD 4/13/2018 2:52:24 PM SYSTEM Page 1 of 1

HPLC of racemic-**2f**

```
Data File E:\DATA\LGY\LGY-1-30\LGY-1-30 2017-11-18 09-16-49\003-0701.D
Sample Name: LGY-1-30-3

    =====================================================================
    Acq. Operator   : SYSTEM                          Seq. Line :   7
    Acq. Instrument : 1260HPLC-VWD                     Location : Vial 3
    Injection Date  : 11/18/2017 2:32:24 PM                 Inj :   1
                                                     Inj Volume : 5.000 µl
    Acq. Method     : E:\DATA\LGY\LGY-1-30\LGY-1-30 2017-11-18 09-16-49\VWD-AD1-2)-90-10-1ML-
                      5UL-220NM-120MIN.M
    Last changed    : 11/18/2017 3:20:37 PM by SYSTEM
                      (modified after loading)
    Analysis Method : E:\DATA\LGY\LGY-1-30\LGY-1-30 2017-11-18 09-16-49\VWD-AD1-2)-90-10-1ML-
                      5UL-220NM-120MIN.M (Sequence Method)
    Last changed    : 4/13/2018 2:55:30 PM by SYSTEM
                      (modified after loading)
    Additional Info : Peak(s) manually integrated
```

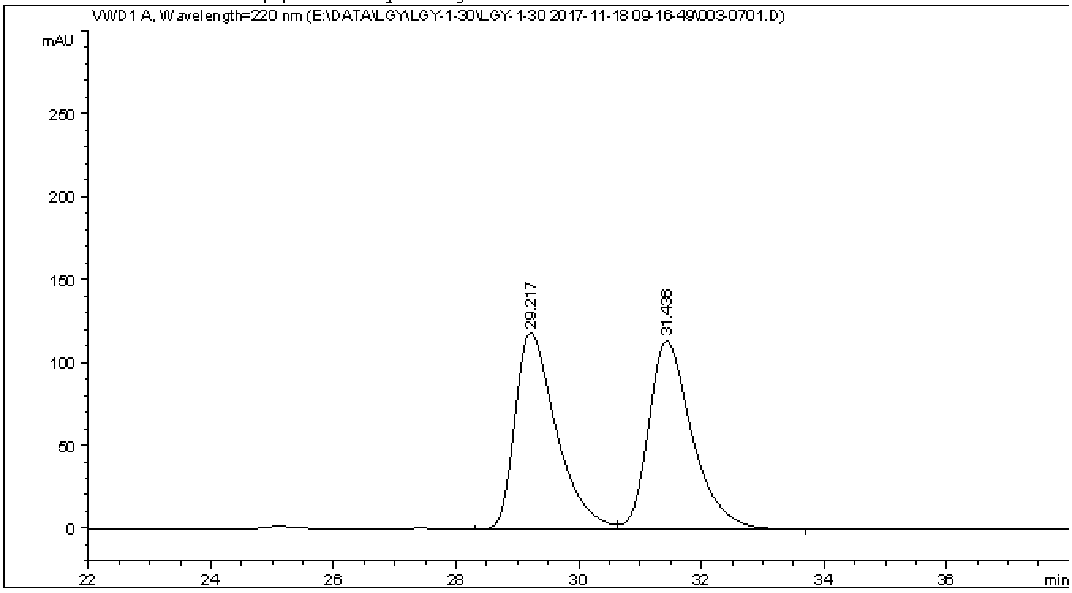


```
    =====================================================================
                             Area Percent Report
    =====================================================================

    Sorted By             :      Signal
    Multiplier            :      1.0000
    Dilution              :      1.0000
    Do not use Multiplier & Dilution Factor with ISTDs


    Signal 1: VWD1 A, Wavelength=220 nm
```

Peak #	RetTime [min]	Type	Width [min]	Area [mAU*s]	Height [mAU]	Area %
1	29.217	BV	0.7098	5531.45459	118.23405	49.8168
2	31.436	VB	0.7460	5572.14600	113.45072	50.1832

Totals : 1.11036e4   231.68477

===

HPLC of **2f**

Data File E:\DATA\LGY\LGY-1-99\LGY-1-99 2018-01-17 23-28-41\011-0301.D
Sample Name: LGY-1-99-1

```
=====================================================================
Acq. Operator   : SYSTEM                          Seq. Line :   3
Acq. Instrument : 1260HPLC-VWD                     Location : Vial 11
Injection Date  : 1/18/2018 12:11:03 AM                 Inj :   1
                                                 Inj Volume : 5.000 µl
Acq. Method     : E:\DATA\LGY\LGY-1-99\LGY-1-99 2018-01-17 23-28-41\VWD-AD(1-2)-90-10-1ML-
                  5UL-220NM-40MIN.M
Last changed    : 1/17/2018 11:28:41 PM by SYSTEM
Analysis Method : E:\DATA\LGY\LGY-1-99\LGY-1-99 2018-01-17 23-28-41\VWD-AD(1-2)-90-10-1ML-
                  5UL-220NM-40MIN.M (Sequence Method)
Last changed    : 4/13/2018 3:01:00 PM by SYSTEM
                  (modified after loading)
Additional Info : Peak(s) manually integrated
```

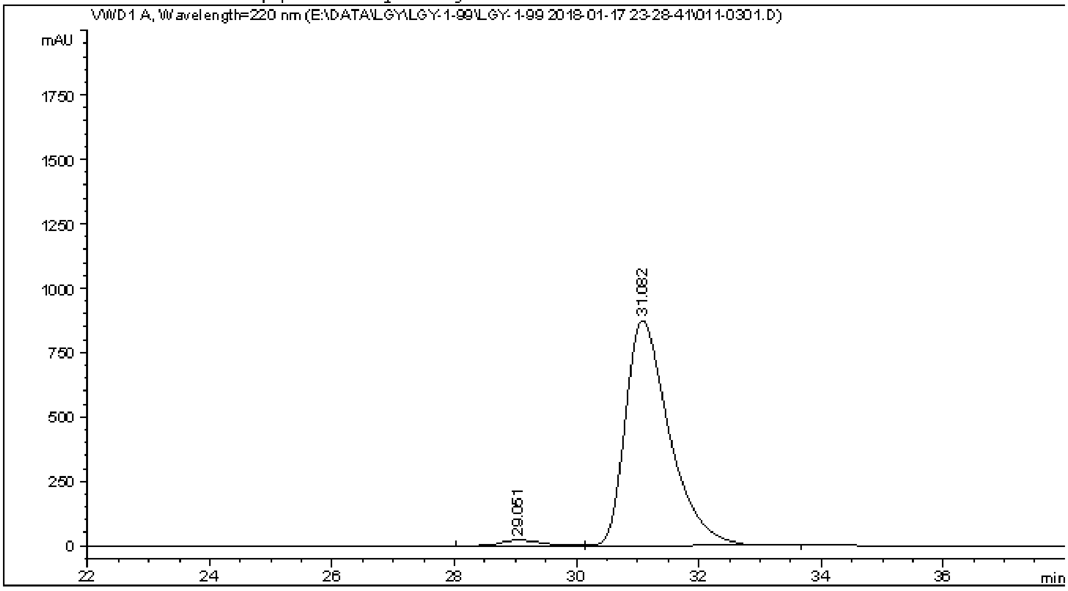


```
=====================================================================
                         Area Percent Report
=====================================================================

Sorted By             :      Signal
Multiplier            :      1.0000
Dilution              :      1.0000
Do not use Multiplier & Dilution Factor with ISTDs


Signal 1: VWD1 A, Wavelength=220 nm

Peak RetTime Type  Width     Area      Height     Area
  #   [min]        [min]   [mAU*s]     [mAU]        %
----|-------|----|-------|----------|----------|--------|
   1  29.051 BV    0.6723  909.31097   20.34970   2.0434
   2  31.082 VB    0.7529 4.35909e4  873.90997  97.9566

Totals :                  4.45002e4  894.25967

=====================================================================
                          *** End of Report ***
```

HPLC of racemic-**2g**

```
Data File E:\DATA\LGY\LGY-1-43-RE-1\LGY-1-43-RE-1 2017-11-22 08-15-14\001-0401.D
Sample Name: LGY-1-43-3--RE

    =====================================================================
    Acq. Operator    : SYSTEM                          Seq. Line :   4
    Acq. Instrument : 1260HPLC-VWD                      Location : Vial 1
    Injection Date   : 11/22/2017 10:40:29 AM                Inj :   1
                                                      Inj Volume : 5.000 µl
    Acq. Method      : E:\DATA\LGY\LGY-1-43-RE-1\LGY-1-43-RE-1 2017-11-22 08-15-14\VWD-AD1-2)-
                       90-10-1ML-5UL-220NM-120MIN.M
    Last changed     : 11/22/2017 10:25:44 AM by SYSTEM
    Analysis Method : E:\DATA\LGY\LGY-1-43-RE-1\LGY-1-43-RE-1 2017-11-22 08-15-14\VWD-AD1-2)-
                       90-10-1ML-5UL-220NM-120MIN.M (Sequence Method)
    Last changed     : 4/13/2018 3:11:26 PM by SYSTEM
                       (modified after loading)
    Additional Info : Peak(s) manually integrated
```

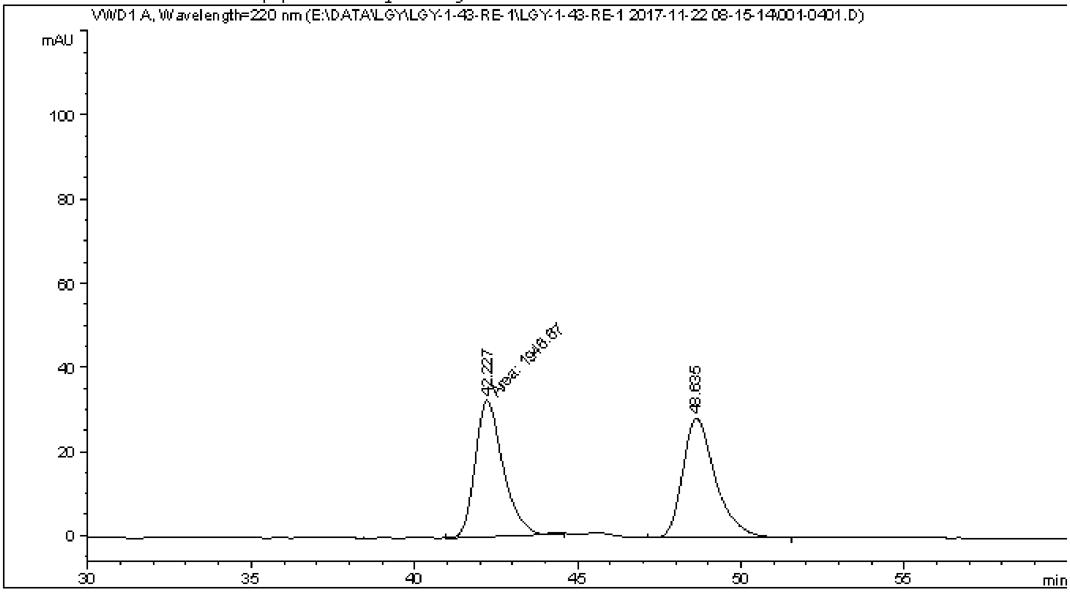


```
    =====================================================================
                         Area Percent Report
    =====================================================================

    Sorted By             :      Signal
    Multiplier            :      1.0000
    Dilution              :      1.0000
    Do not use Multiplier & Dilution Factor with ISTDs


    Signal 1: VWD1 A, Wavelength=220 nm

    Peak RetTime Type  Width     Area      Height     Area
      #   [min]        [min]   [mAU*s]     [mAU]        %
    ----|-------|----|-------|----------|----------|--------|
       1  42.227 MM    0.9985 1946.67358   32.49449  49.5317
       2  48.635 BB    1.0459 1983.48608   28.43331  50.4683

    Totals :                  3930.15967   60.92780

    =====================================================================
                          *** End of Report ***
```

1260HPLC-VWD 4/13/2018 3:11:31 PM SYSTEM   Page 1 of 1

HPLC of **2g**

```
Data File E:\DATA\LGY\LGY-1-99\LGY-1-99 2018-01-17 23-28-41\012-0401.D
Sample Name: LGY-1-99-2

    =====================================================================
    Acq. Operator   : SYSTEM                          Seq. Line :   4
    Acq. Instrument : 1260HPLC-VWD                     Location : Vial 12
    Injection Date  : 1/18/2018 12:51:51 AM                  Inj :   1
                                                     Inj Volume : 5.000 µl
    Acq. Method     : E:\DATA\LGY\LGY-1-99\LGY-1-99 2018-01-17 23-28-41\VWD-AD(1-2)-90-10-1ML-
                      5UL-220NM-60MIN.M
    Last changed    : 1/17/2018 11:28:42 PM by SYSTEM
    Analysis Method : E:\DATA\LGY\LGY-1-99\LGY-1-99 2018-01-17 23-28-41\VWD-AD(1-2)-90-10-1ML-
                      5UL-220NM-60MIN.M (Sequence Method)
    Last changed    : 4/13/2018 3:16:25 PM by SYSTEM
                      (modified after loading)
    Additional Info : Peak(s) manually integrated
```

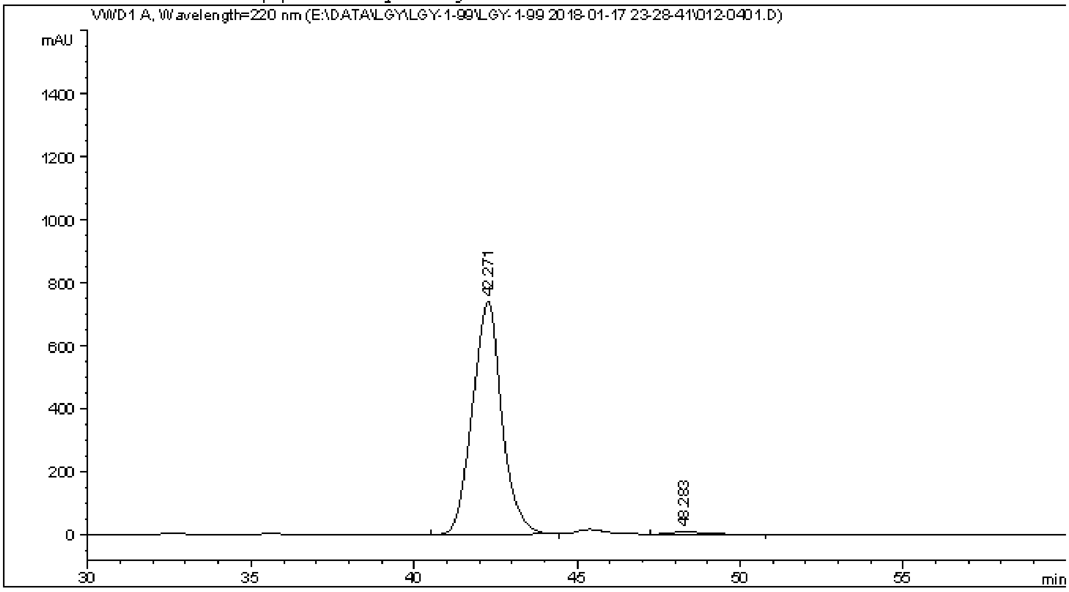


```
    =====================================================================
                             Area Percent Report
    =====================================================================

    Sorted By             :      Signal
    Multiplier            :      1.0000
    Dilution              :      1.0000
    Do not use Multiplier & Dilution Factor with ISTDs


    Signal 1: VWD1 A, Wavelength=220 nm

    Peak RetTime Type  Width     Area      Height     Area
      #   [min]        [min]   [mAU*s]     [mAU]        %
    ----|-------|----|-------|----------|----------|--------|
       1  42.271 BB    0.9493 4.63086e4  739.79163  98.8203
       2  48.283 BB    0.9826  552.80939    8.31575   1.1797

    Totals :                  4.68614e4  748.10738

    =====================================================================
                              *** End of Report ***

1260HPLC-VWD 4/13/2018 3:16:32 PM SYSTEM                                  Page 1 of 1
```

HPLC of racemic-**2h**

Data File E:\DATA\LGY\LGY-1-62-RAC-2\LGY-1-62-RAC-2 2017-12-20 09-19-39\005-0301.D
Sample Name: LGY-1-62-1

===

Acq. Operator : SYSTEM Seq. Line : 3
Acq. Instrument : 1260HPLC-DAD Location : Vial 5
Injection Date : 12/20/2017 10:48:46 AM Inj : 1
Inj Volume : 7.000 µl
Acq. Method : E:\DATA\LGY\LGY-1-62-RAC-2\LGY-1-62-RAC-2 2017-12-20 09-19-39\DAD-OD(1-2)-85-15-1ML-7UL-ALL-70MIN.M
Last changed : 12/20/2017 9:19:39 AM by SYSTEM
Analysis Method : E:\DATA\LGY\LGY-1-62-RAC-2\LGY-1-62-RAC-2 2017-12-20 09-19-39\DAD-OD(1-2)-85-15-1ML-7UL-ALL-70MIN.M (Sequence Method)
Last changed : 4/13/2018 3:25:12 PM by SYSTEM
(modified after loading)
Additional Info : Peak(s) manually integrated

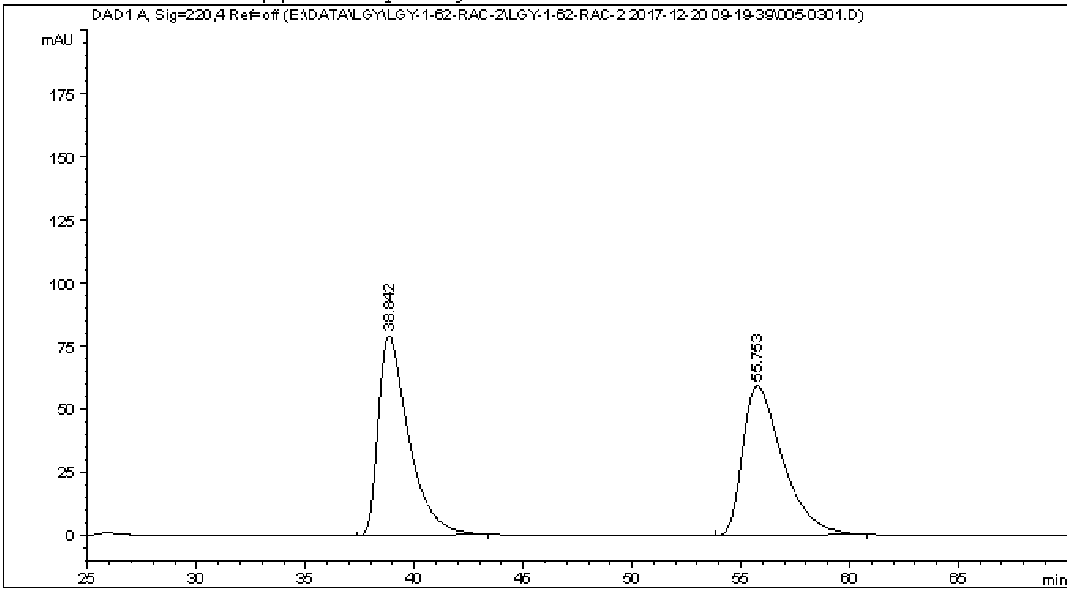

===

Area Percent Report

===

Sorted By : Signal
Multiplier : 1.0000
Dilution : 1.0000
Do not use Multiplier & Dilution Factor with ISTDs

Signal 1: DAD1 A, Sig=220,4 Ref=off

Peak #	RetTime [min]	Type	Width [min]	Area [mAU*s]	Height [mAU]	Area %
1	38.842	BB	1.3572	7681.99365	79.09193	50.3339
2	55.753	BB	1.5096	7580.08252	59.13079	49.6661

Totals : 1.52621e4 138.22271

===

*** End of Report ***

1260HPLC-VWD 4/13/2018 3:25:17 PM SYSTEM Page 1 of 1

HPLC of **2h**

Data File E:\DATA\YCC\YCC-2018-01-17\YCC-233 2018-04-12 08-57-37\004-5001.D
Sample Name: LGY-1-99-3

===

```
Acq. Operator   : SYSTEM                        Seq. Line :  50
Acq. Instrument : 1260HPLC-DAD                   Location : Vial 4
Injection Date  : 4/13/2018 8:38:57 AM                Inj :   1
                                               Inj Volume : 7.000 µl
Acq. Method     : E:\DATA\YCC\YCC-2018-01-17\YCC-233 2018-04-12 08-57-37\DAD-OD(1-2)-85-15
                  -1ML-7UL-ALL-70MIN.M
Last changed    : 4/12/2018 8:57:25 PM by SYSTEM
Analysis Method : E:\DATA\YCC\YCC-2018-01-17\YCC-233 2018-04-12 08-57-37\DAD-OD(1-2)-85-15
                  -1ML-7UL-ALL-70MIN.M (Sequence Method)
Last changed    : 4/13/2018 3:30:25 PM by SYSTEM
                  (modified after loading)
Additional Info : Peak(s) manually integrated
```

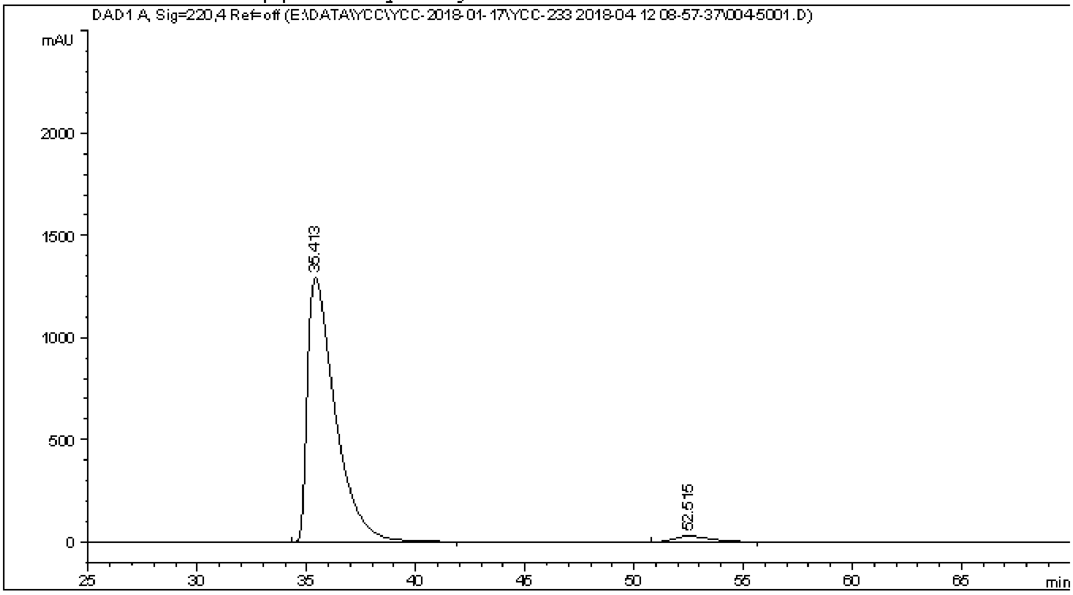

===

Area Percent Report

===

```
Sorted By             :      Signal
Multiplier            :      1.0000
Dilution              :      1.0000
Do not use Multiplier & Dilution Factor with ISTDs
```

Signal 1: DAD1 A, Sig=220,4 Ref=off

Peak #	RetTime [min]	Type	Width [min]	Area [mAU*s]	Height [mAU]	Area %
1	35.413	BB	1.2040	1.13739e5	1290.86548	97.4011
2	52.515	BB	1.2788	3034.80859	27.77357	2.5989

Totals : 1.16774e5 1318.63905

===

*** End of Report ***

HPLC of racemic-**2i**

Data File E:\DATA\LGY\LGY-1-89\LGY-1-89 2017-12-28 08-35-12\041-0201.D
Sample Name: LGY-1-89-RAC

===
Acq. Operator : SYSTEM Seq. Line : 2
Acq. Instrument : 1260HPLC-VWD Location : Vial 41
Injection Date : 12/28/2017 9:09:00 AM Inj : 1
Inj Volume : 5.000 µl
Acq. Method : E:\DATA\LGY\LGY-1-89\LGY-1-89 2017-12-28 08-35-12\VWD-AS(1-6)-80-20-1ML-5UL-220NM-90MIN.M
Last changed : 12/28/2017 10:31:49 AM by SYSTEM
(modified after loading)
Analysis Method : E:\DATA\LGY\LGY-1-89\LGY-1-89 2017-12-28 08-35-12\VWD-AS(1-6)-80-20-1ML-5UL-220NM-90MIN.M (Sequence Method)
Last changed : 4/13/2018 3:35:12 PM by SYSTEM
(modified after loading)
Additional Info : Peak(s) manually integrated

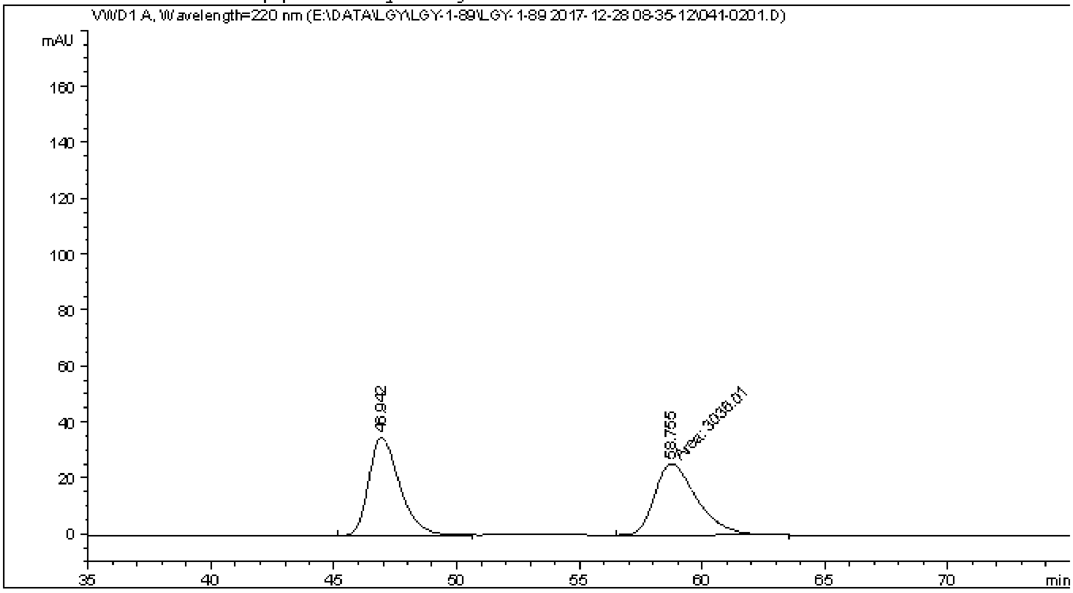

===
Area Percent Report
===

Sorted By : Signal
Multiplier : 1.0000
Dilution : 1.0000
Do not use Multiplier & Dilution Factor with ISTDs

Signal 1: VWD1 A, Wavelength=220 nm

Peak #	RetTime [min]	Type	Width [min]	Area [mAU*s]	Height [mAU]	Area %
1	46.942	BB	1.3121	3043.56836	34.98038	50.0622
2	58.755	MM	1.9840	3036.00806	25.50352	49.9378

Totals : 6079.57642 60.48391

===

1260HPLC-VWD 4/13/2018 3:35:23 PM SYSTEM Page 1 of 1

HPLC of **2i**

Data File E:\DATA\LGY\LGY-1-95\LGY-1-95-RE 2018-01-13 09-06-53\045-0801.D
Sample Name: LGY-1-95-11-RE

===

Acq. Operator : SYSTEM Seq. Line : 8
Acq. Instrument : 1260HPLC-VWD Location : Vial 45
Injection Date : 1/13/2018 3:01:51 PM Inj : 1
Inj Volume : 5.000 µl
Acq. Method : E:\DATA\LGY\LGY-1-95\LGY-1-95-RE 2018-01-13 09-06-53\VWD-AS(1-6)-80-20-1ML-5UL-220NM-75MIN.M
Last changed : 1/13/2018 9:06:54 AM by SYSTEM
Analysis Method : E:\DATA\LGY\LGY-1-95\LGY-1-95-RE 2018-01-13 09-06-53\VWD-AS(1-6)-80-20-1ML-5UL-220NM-75MIN.M (Sequence Method)
Last changed : 4/13/2018 3:50:00 PM by SYSTEM
(modified after loading)
Additional Info : Peak(s) manually integrated

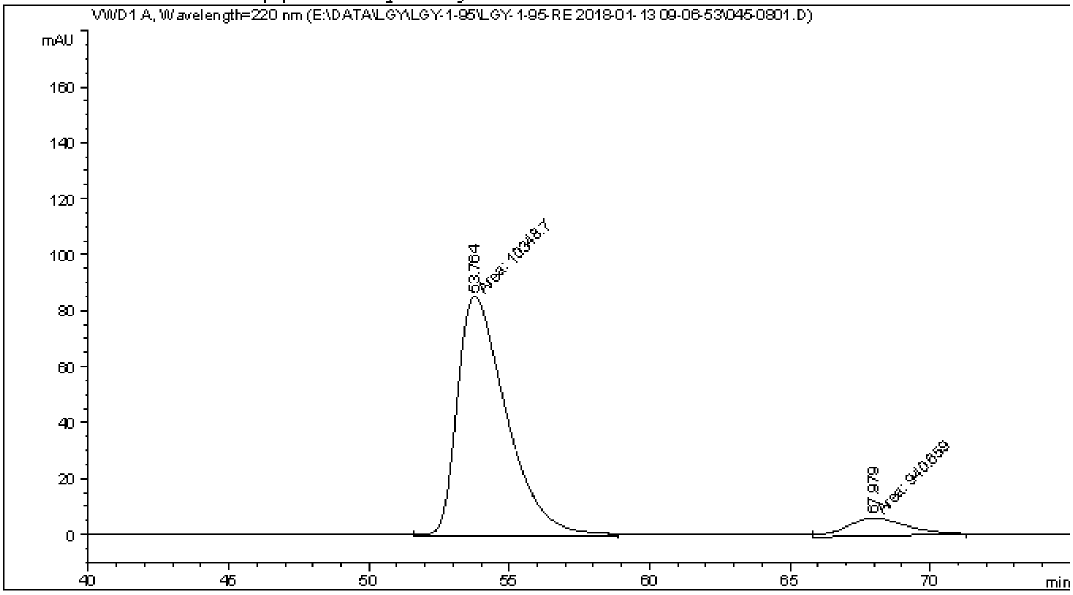

===

Area Percent Report

===

Sorted By : Signal
Multiplier : 1.0000
Dilution : 1.0000
Do not use Multiplier & Dilution Factor with ISTDs

Signal 1: VWD1 A, Wavelength=220 nm

Peak #	RetTime [min]	Type	Width [min]	Area [mAU*s]	Height [mAU]	Area %
1	53.764	MM	2.0249	1.03487e4	85.17876	91.6677
2	67.979	MM	2.4811	940.65881	6.31878	8.3323

Totals : 1.12894e4 91.49755

===

*** End of Report ***

1260HPLC-VWD 4/13/2018 3:50:20 PM SYSTEM Page 1 of 1

HPLC of racemic-**2j**

Data File E:\DATA\LGY\LGY-1-95\LGY-1-95-RE 2018-01-13 18-47-33\064-0701.D
Sample Name: LGY-1-94-1-RA

```
=====================================================================
Acq. Operator   : SYSTEM                          Seq. Line :   7
Acq. Instrument : 1260HPLC-VWD                     Location : Vial 64
Injection Date  : 1/14/2018 2:53:48 AM                  Inj :   1
                                                Inj Volume : 5.000 µl
Acq. Method     : E:\DATA\LGY\LGY-1-95\LGY-1-95-RE 2018-01-13 18-47-33\VWD-AS(1-6)-80-20-
                  1ML-5UL-220NM-90MIN.M
Last changed    : 1/13/2018 6:47:33 PM by SYSTEM
Analysis Method : E:\DATA\LGY\LGY-1-95\LGY-1-95-RE 2018-01-13 18-47-33\VWD-AS(1-6)-80-20-
                  1ML-5UL-220NM-90MIN.M (Sequence Method)
Last changed    : 4/13/2018 5:11:04 PM by SYSTEM
                  (modified after loading)
Additional Info : Peak(s) manually integrated
```

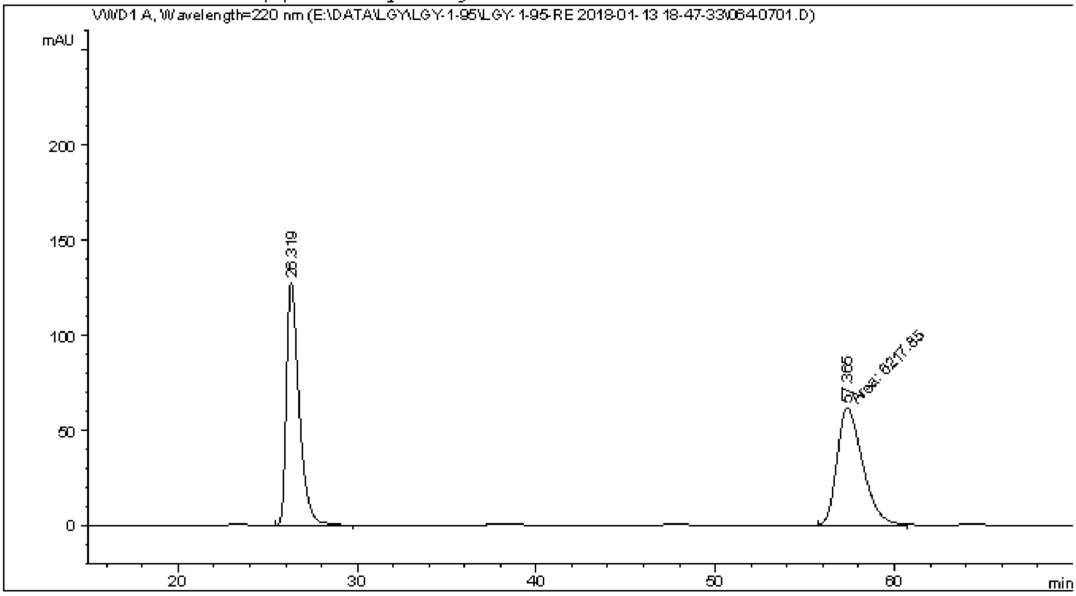


```
=====================================================================
                         Area Percent Report
=====================================================================

Sorted By             :      Signal
Multiplier            :      1.0000
Dilution              :      1.0000
Do not use Multiplier & Dilution Factor with ISTDs


Signal 1: VWD1 A, Wavelength=220 nm

Peak RetTime Type  Width     Area      Height     Area
  #   [min]        [min]   [mAU*s]     [mAU]        %
----|-------|----|-------|----------|----------|--------|
   1  26.319 BB    0.7423 6276.41748  127.73354  50.2344
   2  57.365 MM    1.6722 6217.85449   61.97150  49.7656

Totals :                  1.24943e4  189.70504

=====================================================================
                          *** End of Report ***
```

HPLC of **2j**

Data File E:\DATA\LGY\LGY-1-99\LGY-1-99 2018-01-18 18-48-43\012-0801.D
Sample Name: LGY-1-99-5-RE-1

===
Acq. Operator : SYSTEM Seq. Line : 8
Acq. Instrument : 1260HPLC-VWD Location : Vial 12
Injection Date : 1/19/2018 12:35:40 AM Inj : 1
Inj Volume : 5.000 µl
Acq. Method : E:\DATA\LGY\LGY-1-99\LGY-1-99 2018-01-18 18-48-43\VWD-AS(1-6)-80-20-1ML-5UL-220NM-70MIN.M
Last changed : 1/18/2018 6:48:44 PM by SYSTEM
Analysis Method : E:\DATA\LGY\LGY-1-99\LGY-1-99 2018-01-18 18-48-43\VWD-AS(1-6)-80-20-1ML-5UL-220NM-70MIN.M (Sequence Method)
Last changed : 4/13/2018 5:15:55 PM by SYSTEM
(modified after loading)
Additional Info : Peak(s) manually integrated

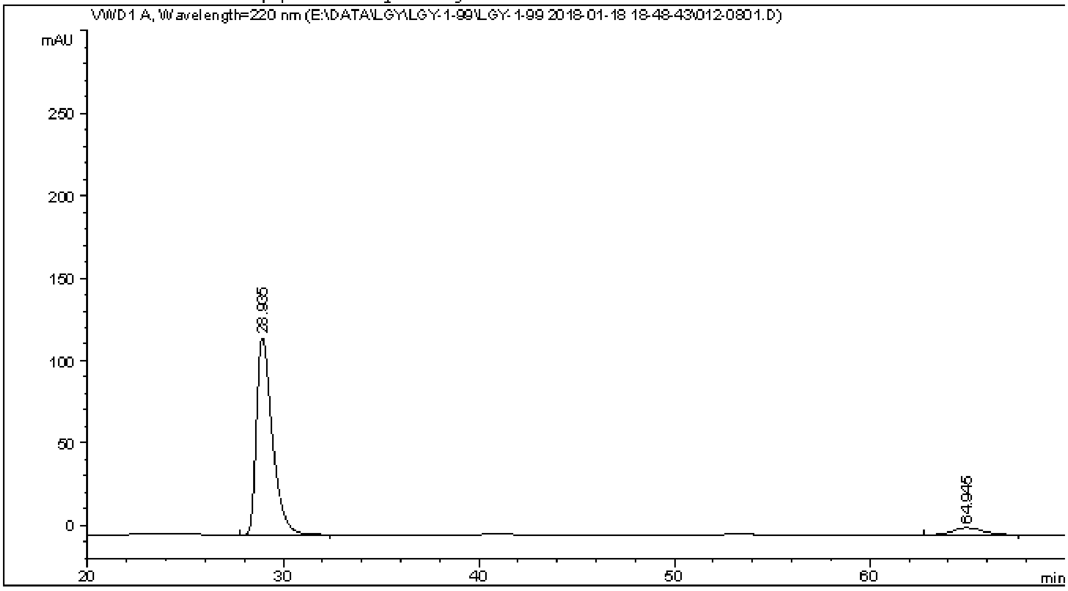

===
Area Percent Report
===

Sorted By : Signal
Multiplier : 1.0000
Dilution : 1.0000
Do not use Multiplier & Dilution Factor with ISTDs

Signal 1: VWD1 A, Wavelength=220 nm

Peak #	RetTime [min]	Type	Width [min]	Area [mAU*s]	Height [mAU]	Area %
1	28.935	BB	0.8552	6747.79639	119.24654	93.9898
2	64.945	BB	1.2595	431.48953	4.03849	6.0102

Totals : 7179.28592 123.28502

===
*** End of Report ***

1260HPLC-VWD 4/13/2018 5:16:41 PM SYSTEM Page 1 of 1

HPLC of racemic-**2k**

Data File E:\DATA\YJX\YJX-1-93\YJX-1-93 2018-03-28 09-24-48\041-1301.D
Sample Name: LGY-1-130-RA

===

Acq. Operator : SYSTEM Seq. Line : 13
Acq. Instrument : 1260HPLC-VWD Location : Vial 41
Injection Date : 3/28/2018 3:52:49 PM Inj : 1
Inj Volume : 5.000 µl
Acq. Method : E:\DATA\YJX\YJX-1-93\YJX-1-93 2018-03-28 09-24-48\VWD-AS(1-6)-80-20-1ML-5UL-220NM-90MIN.M
Last changed : 3/28/2018 9:59:05 AM by SYSTEM
Analysis Method : E:\DATA\YJX\YJX-1-93\YJX-1-93 2018-03-28 09-24-48\VWD-AS(1-6)-80-20-1ML-5UL-220NM-90MIN.M (Sequence Method)
Last changed : 4/26/2018 9:45:39 PM by SYSTEM
(modified after loading)
Additional Info : Peak(s) manually integrated

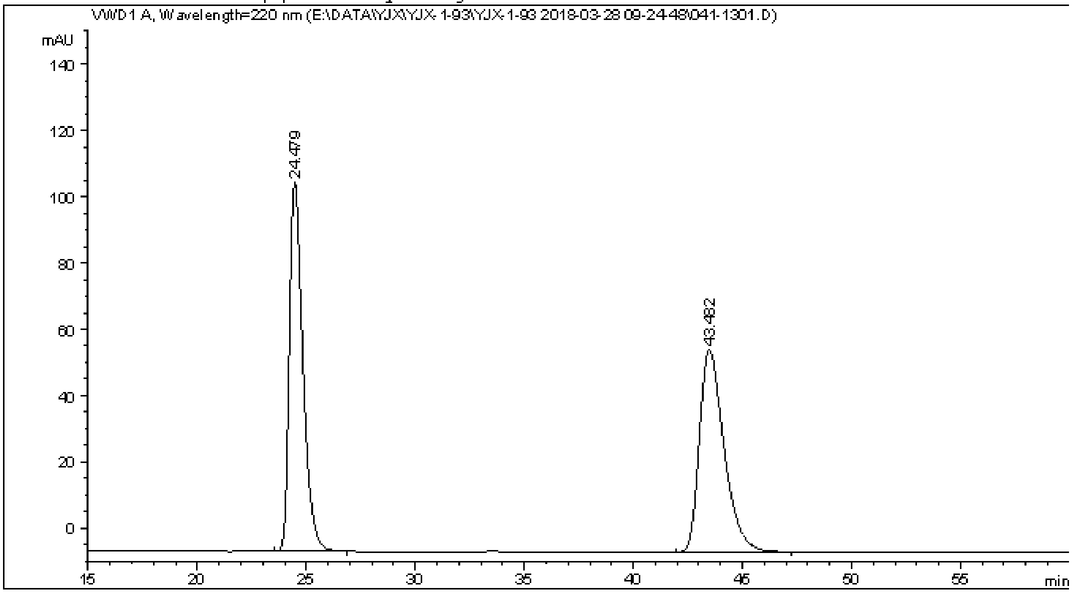

===

Area Percent Report

===

Sorted By : Signal
Multiplier : 1.0000
Dilution : 1.0000
Do not use Multiplier & Dilution Factor with ISTDs

Signal 1: VWD1 A, Wavelength=220 nm

Peak #	RetTime [min]	Type	Width [min]	Area [mAU*s]	Height [mAU]	Area %
1	24.479	BB	0.6556	4785.61572	111.09396	49.9677
2	43.482	BB	1.1908	4791.80518	60.91084	50.0323

Totals : 9577.42090 172.00481

===

*** End of Report ***

1260HPLC-VWD 4/26/2018 9:45:50 PM SYSTEM Page 1 of 1

HPLC of **2k**

Data File E:\DATA\LGY\LGY-1-153\LGY-1-153 2018-04-19 21-33-46\022-3301.D
Sample Name: LGY-1-154-2

===

Acq. Operator : SYSTEM Seq. Line : 33
Acq. Instrument : 1260HPLC-VWD Location : Vial 22
Injection Date : 4/21/2018 12:45:12 AM Inj : 1
Inj Volume : 5.000 µl
Acq. Method : E:\DATA\LGY\LGY-1-153\LGY-1-153 2018-04-19 21-33-46\VWD-AS(1-6)-80-20-1ML-5UL-220NM-90MIN.M
Last changed : 4/19/2018 9:33:47 PM by SYSTEM
Analysis Method : E:\DATA\LGY\LGY-1-153\LGY-1-153 2018-04-19 21-33-46\VWD-AS(1-6)-80-20-1ML-5UL-220NM-90MIN.M (Sequence Method)
Last changed : 4/26/2018 9:53:18 PM by SYSTEM
(modified after loading)
Additional Info : Peak(s) manually integrated

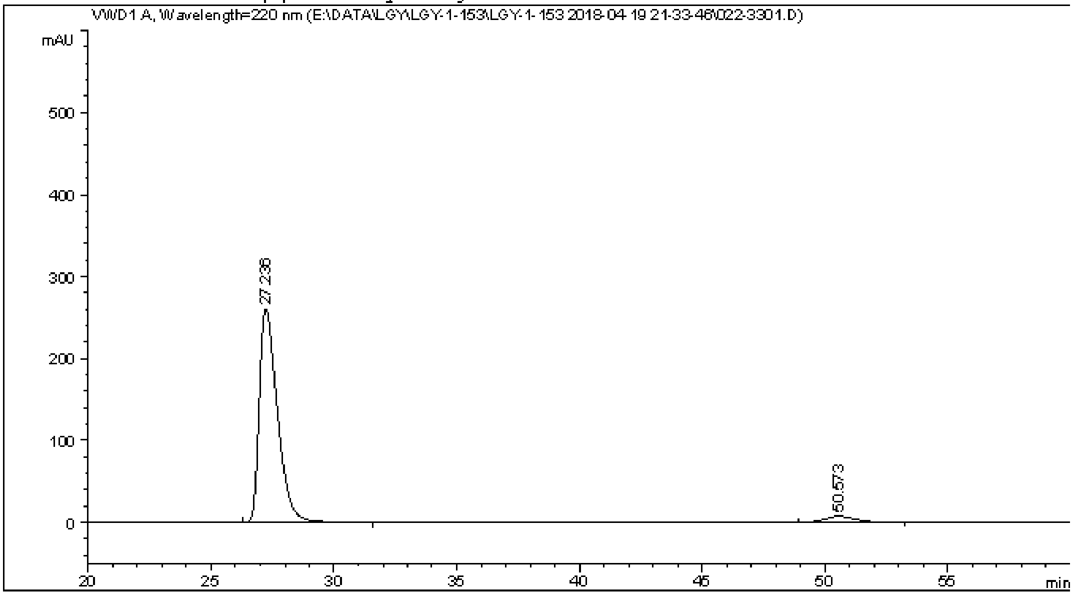

===

Area Percent Report

===

Sorted By : Signal
Multiplier : 1.0000
Dilution : 1.0000
Do not use Multiplier & Dilution Factor with ISTDs

Signal 1: VWD1 A, Wavelength=220 nm

Peak #	RetTime [min]	Type	Width [min]	Area [mAU*s]	Height [mAU]	Area %
1	27.236	BB	0.7804	1.32302e4	259.66959	95.8221
2	50.573	BB	1.1083	576.84888	7.01531	4.1779

Totals : 1.38071e4 266.68490

===

*** End of Report ***

1260HPLC-VWD 4/26/2018 9:53:26 PM SYSTEM Page 1 of 1

HPLC of racemic-**2l**

```
Data File E:\DATA\LGY\LGY-1-161\LGY-1-161 2018-04-25 10-36-34\011-0301.D
Sample Name: LGY-1-161

    =====================================================================
    Acq. Operator   : SYSTEM                          Seq. Line :   3
    Acq. Instrument : 1260HPLC-VWD                     Location : Vial 11
    Injection Date  : 4/25/2018 11:21:38 AM                 Inj :   1
                                                     Inj Volume : 5.000 µl
    Acq. Method     : E:\DATA\LGY\LGY-1-161\LGY-1-161 2018-04-25 10-36-34\VWD-AS(1-6)-80-20-
                      1ML-5UL-220NM-90MIN.M
    Last changed    : 4/25/2018 10:36:35 AM by SYSTEM
    Analysis Method : E:\DATA\LGY\LGY-1-161\LGY-1-161 2018-04-25 10-36-34\VWD-AS(1-6)-80-20-
                      1ML-5UL-220NM-90MIN.M (Sequence Method)
    Last changed    : 4/26/2018 9:55:57 PM by SYSTEM
                      (modified after loading)
    Additional Info : Peak(s) manually integrated
```

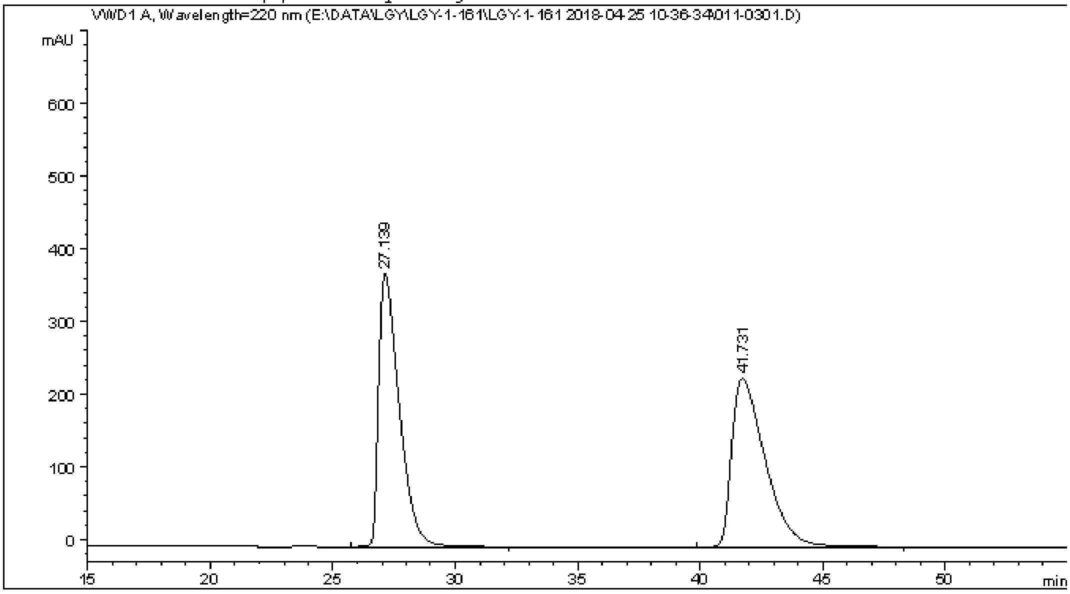


```
    =====================================================================
                            Area Percent Report
    =====================================================================

    Sorted By             :      Signal
    Multiplier            :      1.0000
    Dilution              :      1.0000
    Do not use Multiplier & Dilution Factor with ISTDs


    Signal 1: VWD1 A, Wavelength=220 nm
```

Peak #	RetTime [min]	Type	Width [min]	Area [mAU*s]	Height [mAU]	Area %
1	27.139	BB	0.8775	2.18521e4	375.71609	49.9349
2	41.731	BB	1.4134	2.19090e4	231.87233	50.0651

Totals : 4.37611e4 607.58842

```
    =====================================================================
                             *** End of Report ***
```

1260HPLC-VWD 4/26/2018 9:56:06 PM SYSTEM Page 1 of 1

HPLC of **2l**

Data File E:\DATA\LGY\LGY-1-162\LGY-1-162 2018-04-26 14-42-05\072-0401.D
Sample Name: LGY-1-162-2

===
Acq. Operator : SYSTEM Seq. Line : 4
Acq. Instrument : 1260HPLC-VWD Location : Vial 72
Injection Date : 4/26/2018 4:25:20 PM Inj : 1
Inj Volume : 5.000 µl
Acq. Method : E:\DATA\LGY\LGY-1-162\LGY-1-162 2018-04-26 14-42-05\VWD-AS(1-6)-80-20-1ML-5UL-220NM-60MIN.M
Last changed : 4/26/2018 2:42:05 PM by SYSTEM
Analysis Method : E:\DATA\LGY\LGY-1-162\LGY-1-162 2018-04-26 14-42-05\VWD-AS(1-6)-80-20-1ML-5UL-220NM-60MIN.M (Sequence Method)
Last changed : 4/26/2018 10:00:14 PM by SYSTEM
(modified after loading)
Additional Info : Peak(s) manually integrated

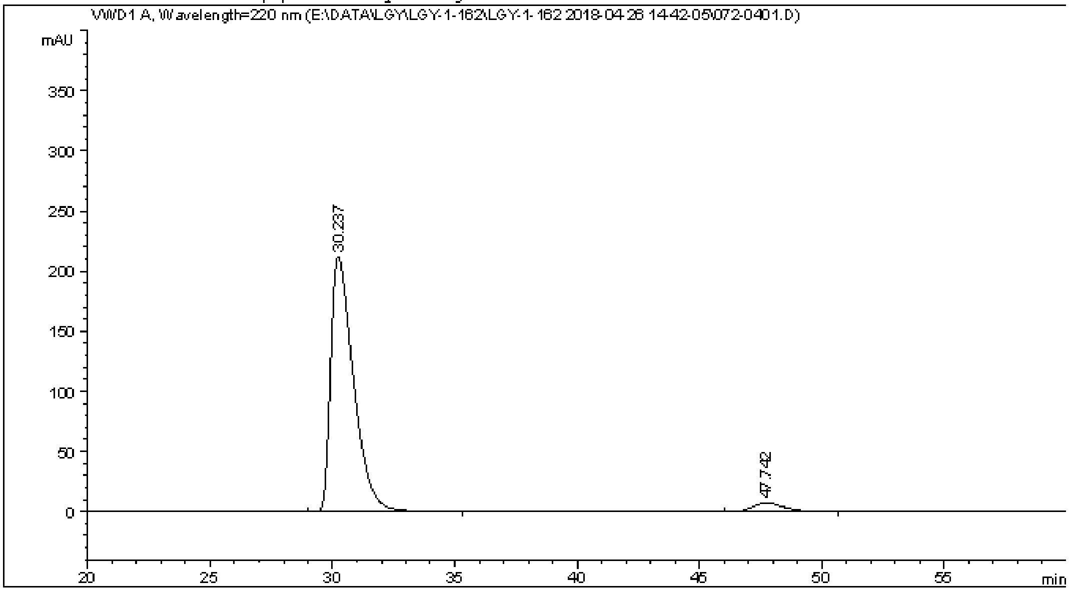

===
Area Percent Report
===

Sorted By : Signal
Multiplier : 1.0000
Dilution : 1.0000
Do not use Multiplier & Dilution Factor with ISTDs

Signal 1: VWD1 A, Wavelength=220 nm

Peak #	RetTime [min]	Type	Width [min]	Area [mAU*s]	Height [mAU]	Area %
1	30.237	BB	0.9804	1.37557e4	212.17484	95.6042
2	47.742	BB	1.2271	632.47974	7.19835	4.3958

Totals : 1.43881e4 219.37319

===
*** End of Report ***

1260HPLC-VWD 4/26/2018 10:00:29 PM SYSTEM Page 1 of 1

HPLC of racemic-**2m**

```
Data File E:\DATA\WSW\WSW-2-162-RACE-2\XZC-171205-1 2017-12-06 09-37-46\002-0801.D
Sample Name: LGY-1-47

    =====================================================================
    Acq. Operator   : SYSTEM                        Seq. Line :   8
    Acq. Instrument : 1260HPLC-VWD                   Location : Vial 2
    Injection Date  : 12/6/2017 1:15:36 PM                Inj :   1
                                                   Inj Volume : 5.000 µl
    Acq. Method     : E:\DATA\WSW\WSW-2-162-RACE-2\XZC-171205-1 2017-12-06 09-37-46\VWD-AS(1-6
                      )-80-20-1ML-5UL-220NM-100MIN.M
    Last changed    : 12/6/2017 9:52:57 AM by SYSTEM
    Analysis Method : E:\DATA\WSW\WSW-2-162-RACE-2\XZC-171205-1 2017-12-06 09-37-46\VWD-AS(1-6
                      )-80-20-1ML-5UL-220NM-100MIN.M (Sequence Method)
    Last changed    : 4/13/2018 5:20:12 PM by SYSTEM
                      (modified after loading)
    Additional Info : Peak(s) manually integrated
```

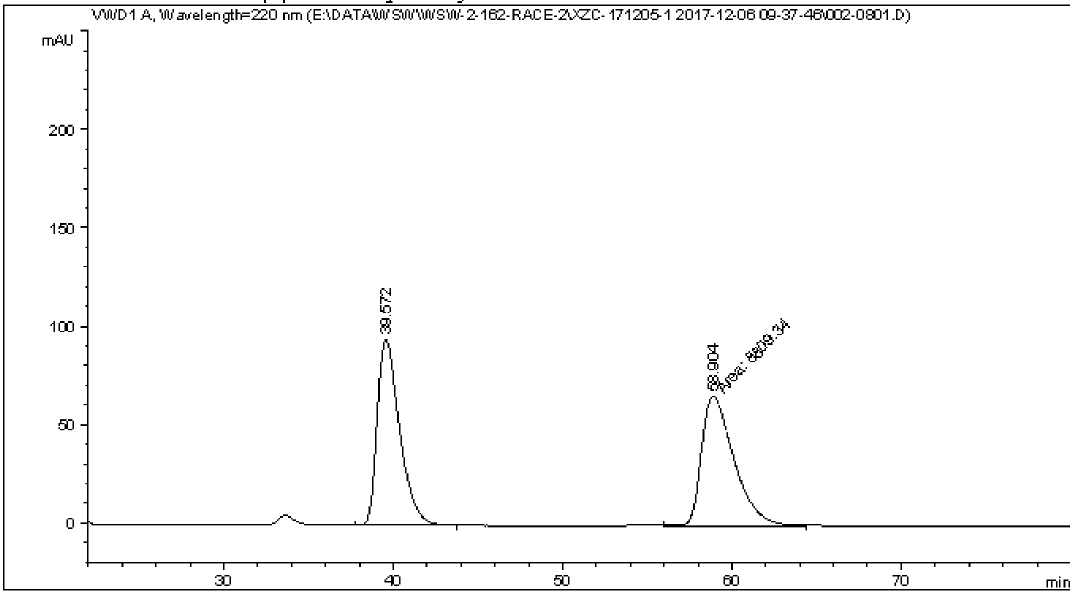


```
    =====================================================================
                             Area Percent Report
    =====================================================================

    Sorted By             :      Signal
    Multiplier            :      1.0000
    Dilution              :      1.0000
    Do not use Multiplier & Dilution Factor with ISTDs


    Signal 1: VWD1 A, Wavelength=220 nm

    Peak RetTime Type  Width     Area      Height     Area
      #   [min]        [min]   [mAU*s]     [mAU]        %
    ----|-------|----|-------|----------|----------|--------|
       1  39.572 BB    1.3921 8682.41602   94.48367  49.6372
       2  58.904 MM    2.2334 8809.33691   65.74026  50.3628

    Totals :                  1.74918e4   160.22394


    =====================================================================
                              *** End of Report ***
```

1260HPLC-VWD 4/13/2018 5:21:15 PM SYSTEM   Page 1 of 1

HPLC of **2m**

Data File E:\DATA\LGY\LGY-1-93\LGY-1-93 2018-01-04 11-23-37\023-1201.D
Sample Name: LGY-1-92-4

```
=====================================================================
Acq. Operator   : SYSTEM                         Seq. Line :  12
Acq. Instrument : 1260HPLC-VWD                    Location : Vial 23
Injection Date  : 1/4/2018 7:00:13 PM                  Inj :   1
                                                Inj Volume : 5.000 µl
Acq. Method     : E:\DATA\LGY\LGY-1-93\LGY-1-93 2018-01-04 11-23-37\VWD-AS(1-6)-80-20-1ML-
                  5UL-220NM-70MIN.M
Last changed    : 1/4/2018 11:27:15 AM by SYSTEM
Analysis Method : E:\DATA\LGY\LGY-1-93\LGY-1-93 2018-01-04 11-23-37\VWD-AS(1-6)-80-20-1ML-
                  5UL-220NM-70MIN.M (Sequence Method)
Last changed    : 4/13/2018 5:25:11 PM by SYSTEM
                  (modified after loading)
Additional Info : Peak(s) manually integrated
```

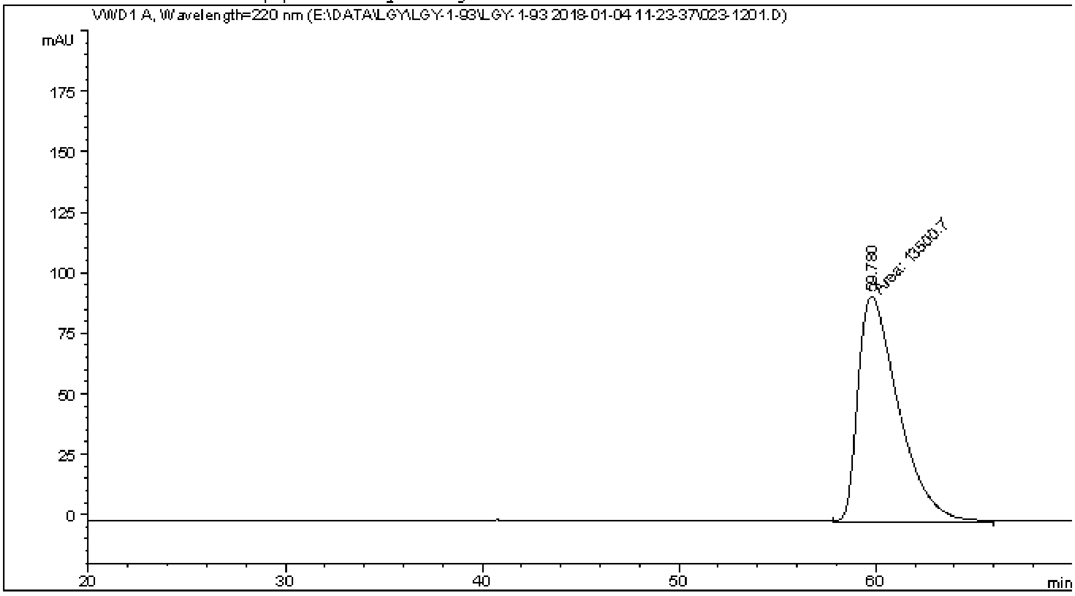


```
=====================================================================
                         Area Percent Report
=====================================================================

Sorted By             :      Signal
Multiplier            :      1.0000
Dilution              :      1.0000
Do not use Multiplier & Dilution Factor with ISTDs


Signal 1: VWD1 A, Wavelength=220 nm

Peak RetTime Type  Width     Area      Height     Area
  #   [min]        [min]   [mAU*s]     [mAU]        %
----|-------|----|-------|----------|----------|--------|
   1  59.780 MM    2.4132 1.35007e4   93.24097 100.0000

Totals :                  1.35007e4   93.24097

=====================================================================
                          *** End of Report ***
```

HPLC of racemic-**2n**

```
Data File E:\DATA\LGY\LGY-1-53-RE-3\LGY-1-53-RE-3 2017-12-01 08-52-48\002-0401.D
Sample Name: LGY-1-54

=====================================================================
Acq. Operator   : SYSTEM                        Seq. Line :   4
Acq. Instrument : 1260HPLC-VWD                   Location : Vial 2
Injection Date  : 12/1/2017 11:08:34 AM               Inj :   1
                                                Inj Volume : 5.000 µl
Acq. Method     : E:\DATA\LGY\LGY-1-53-RE-3\LGY-1-53-RE-3 2017-12-01 08-52-48\VWD-AS(1-6)-
                  80-20-1ML-5UL-220NM-120MIN.M
Last changed    : 12/1/2017 8:52:49 AM by SYSTEM
Analysis Method : E:\DATA\LGY\LGY-1-53-RE-3\LGY-1-53-RE-3 2017-12-01 08-52-48\VWD-AS(1-6)-
                  80-20-1ML-5UL-220NM-120MIN.M (Sequence Method)
Last changed    : 4/13/2018 5:26:51 PM by SYSTEM
                  (modified after loading)
Additional Info : Peak(s) manually integrated
```

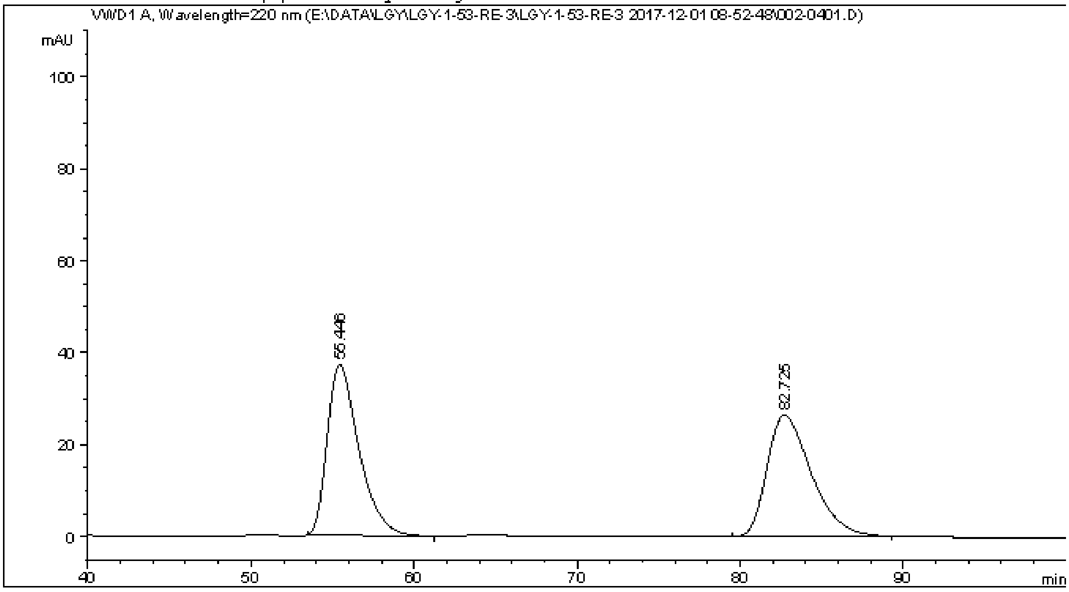


```
=====================================================================
                         Area Percent Report
=====================================================================

Sorted By             :      Signal
Multiplier            :      1.0000
Dilution              :      1.0000
Do not use Multiplier & Dilution Factor with ISTDs


Signal 1: VWD1 A, Wavelength=220 nm

Peak RetTime Type  Width     Area      Height     Area
  #   [min]        [min]   [mAU*s]     [mAU]        %
----|-------|----|-------|----------|----------|--------|
   1  55.446 BB    1.8387 5016.63232   37.12011  49.9658
   2  82.725 BB    2.5774 5023.49561   26.50241  50.0342

Totals :                  1.00401e4   63.62252


=====================================================================
                          *** End of Report ***
```

HPLC of **2n**

Data File E:\DATA\LGY\LGY-1-95\LGY-1-95 2018-01-11 09-00-29\041-1501.D
Sample Name: LGY-1-95-1

```
=====================================================================
Acq. Operator   : SYSTEM                          Seq. Line :  15
Acq. Instrument : 1260HPLC-VWD                     Location : Vial 41
Injection Date  : 1/11/2018 3:18:34 PM                  Inj :   1
                                                 Inj Volume : 5.000 µl
Acq. Method     : E:\DATA\LGY\LGY-1-95\LGY-1-95 2018-01-11 09-00-29\VWD-AS(1-6)-80-20-1ML-
                  5UL-220NM-100MIN.M
Last changed    : 1/11/2018 9:00:30 AM by SYSTEM
Analysis Method : E:\DATA\LGY\LGY-1-95\LGY-1-95 2018-01-11 09-00-29\VWD-AS(1-6)-80-20-1ML-
                  5UL-220NM-100MIN.M (Sequence Method)
Last changed    : 4/13/2018 5:44:37 PM by SYSTEM
                  (modified after loading)
Additional Info : Peak(s) manually integrated
```

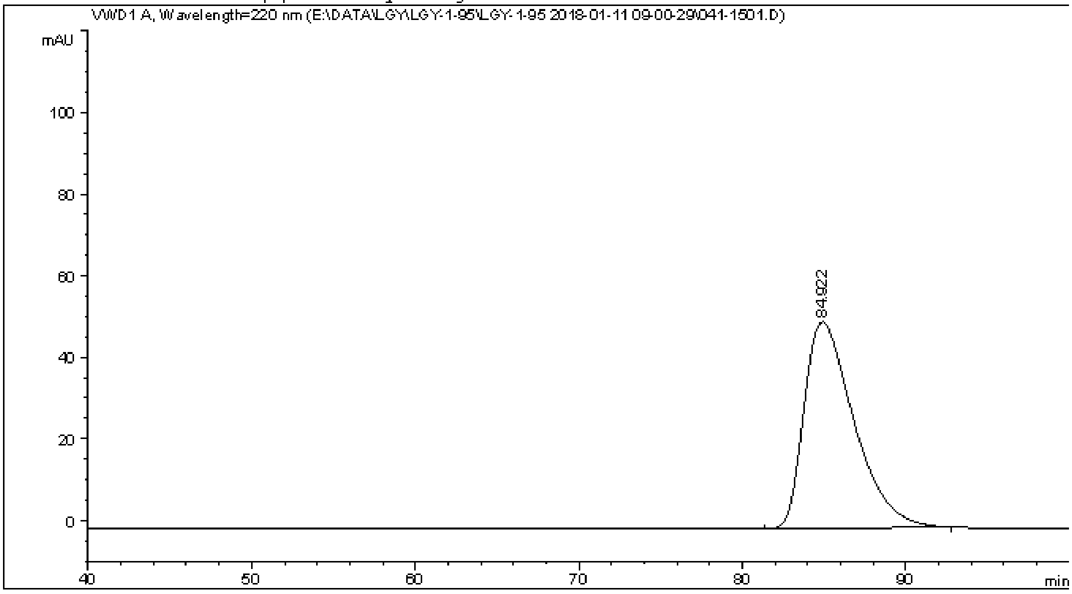


```
=====================================================================
                         Area Percent Report
=====================================================================

Sorted By             :      Signal
Multiplier            :      1.0000
Dilution              :      1.0000
Do not use Multiplier & Dilution Factor with ISTDs


Signal 1: VWD1 A, Wavelength=220 nm

Peak RetTime Type  Width     Area      Height     Area
  #   [min]        [min]   [mAU*s]     [mAU]        %
----|-------|----|-------|----------|----------|--------|
   1  84.922 BB    2.6560 1.07293e4   50.53551 100.0000

Totals :                  1.07293e4   50.53551

=====================================================================
                          *** End of Report ***
```

1260HPLC-VWD 4/13/2018 5:44:40 PM SYSTEM   Page 1 of 1

HPLC of racemic-**2o**

Data File E:\DATA\LGY\LGY-1-72-RACE\YJX-1-19-0 2017-12-16 19-17-14\002-0401.D
Sample Name: LGY-1-72-2

```
=====================================================================
Acq. Operator   : SYSTEM                          Seq. Line :    4
Acq. Instrument : 1260HPLC-VWD                     Location : Vial 2
Injection Date  : 12/16/2017 9:11:48 PM                 Inj :    1
                                                 Inj Volume : 5.000 µl
Acq. Method     : E:\DATA\LGY\LGY-1-72-RACE\YJX-1-19-0 2017-12-16 19-17-14\VWD-AS(1-6)-80-
                  20-1ML-5UL-220NM-70MIN.M
Last changed    : 12/16/2017 7:17:15 PM by SYSTEM
Analysis Method : E:\DATA\LGY\LGY-1-72-RACE\YJX-1-19-0 2017-12-16 19-17-14\VWD-AS(1-6)-80-
                  20-1ML-5UL-220NM-70MIN.M (Sequence Method)
Last changed    : 4/14/2018 7:46:00 PM by SYSTEM
                  (modified after loading)
Additional Info : Peak(s) manually integrated
```

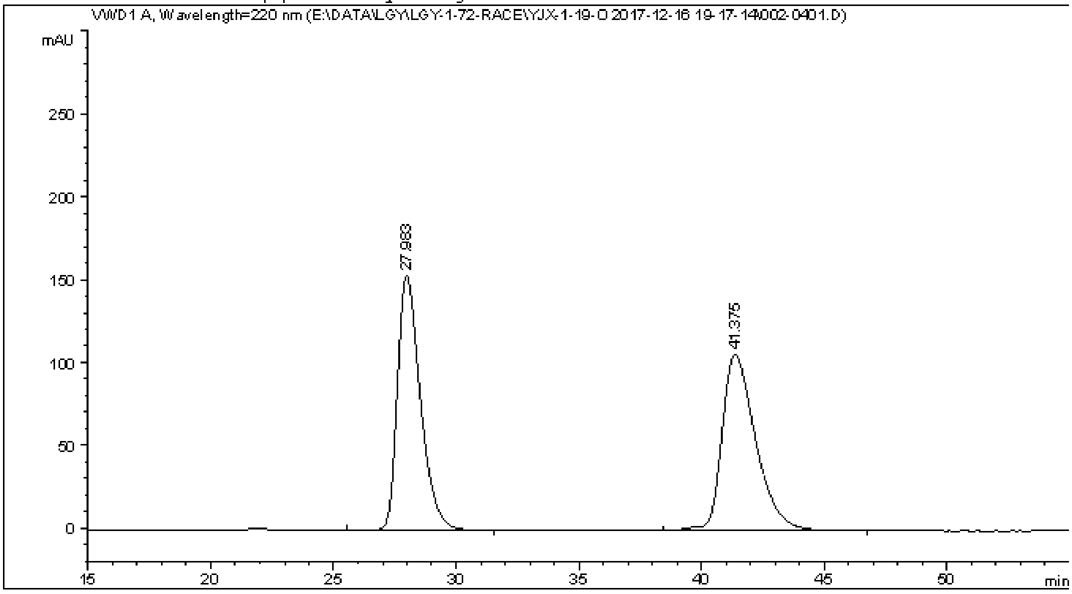


```
=====================================================================
                         Area Percent Report
=====================================================================

Sorted By             :      Signal
Multiplier            :      1.0000
Dilution              :      1.0000
Do not use Multiplier & Dilution Factor with ISTDs


Signal 1: VWD1 A, Wavelength=220 nm

Peak RetTime Type  Width     Area      Height     Area
  #   [min]        [min]   [mAU*s]     [mAU]        %
----|-------|----|-------|----------|----------|--------|
   1  27.983 BB    0.9959 9999.52246  153.72369  49.6051
   2  41.375 BB    1.4506 1.01587e4   106.13880  50.3949

Totals :                  2.01583e4   259.86250


=====================================================================
                          *** End of Report ***
```

1260HPLC-DAD 4/14/2018 7:46:12 PM SYSTEM   Page 1 of 1

HPLC of **2o**

```
Data File E:\DATA\LGY\LGY-1-95\LGY-1-95-RE 2018-01-13 09-06-53\042-0501.D
Sample Name: LGY-1-95-4-RE

    =====================================================================
    Acq. Operator   : SYSTEM                          Seq. Line :   5
    Acq. Instrument : 1260HPLC-VWD                     Location : Vial 42
    Injection Date  : 1/13/2018 11:55:11 AM                 Inj :   1
                                                    Inj Volume : 5.000 µl
    Acq. Method     : E:\DATA\LGY\LGY-1-95\LGY-1-95-RE 2018-01-13 09-06-53\VWD-AS(1-6)-80-20-
                      1ML-5UL-220NM-60MIN.M
    Last changed    : 1/13/2018 9:06:54 AM by SYSTEM
    Analysis Method : E:\DATA\LGY\LGY-1-95\LGY-1-95-RE 2018-01-13 09-06-53\VWD-AS(1-6)-80-20-
                      1ML-5UL-220NM-60MIN.M (Sequence Method)
    Last changed    : 4/14/2018 7:50:03 PM by SYSTEM
                      (modified after loading)
    Additional Info : Peak(s) manually integrated
```

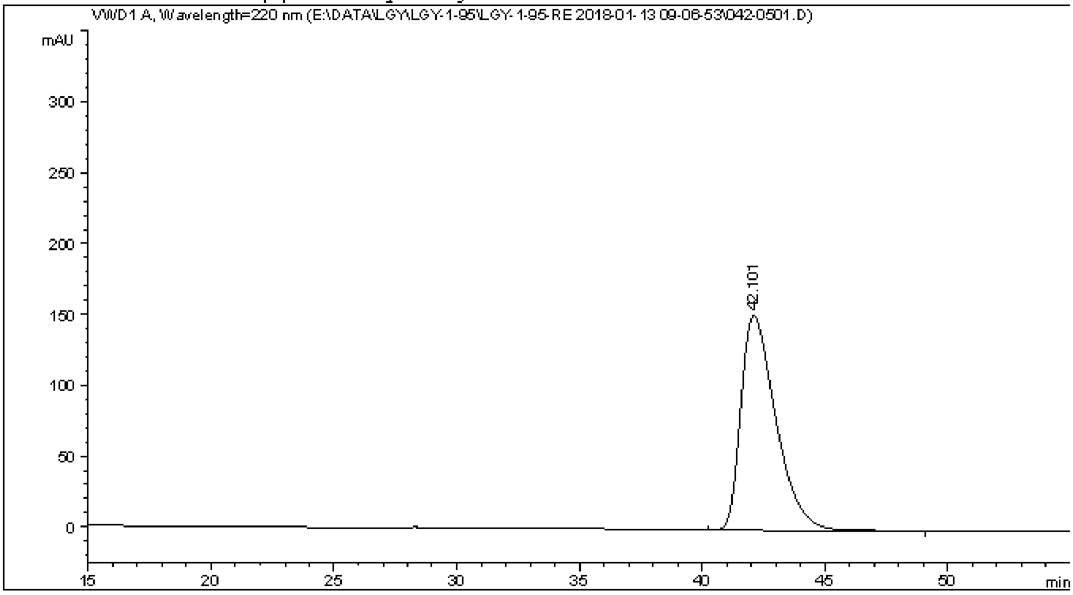


```
    =====================================================================
                             Area Percent Report
    =====================================================================

    Sorted By             :      Signal
    Multiplier            :      1.0000
    Dilution              :      1.0000
    Do not use Multiplier & Dilution Factor with ISTDs


    Signal 1: VWD1 A, Wavelength=220 nm

    Peak RetTime Type  Width     Area      Height     Area
      #   [min]        [min]   [mAU*s]     [mAU]       %
    ----|-------|----|-------|----------|----------|--------|
       1  42.101 BB    1.5028 1.50565e4   151.58403 100.0000

    Totals :                  1.50565e4   151.58403

    =====================================================================
                              *** End of Report ***
```

1260HPLC-DAD 4/14/2018 7:50:09 PM SYSTEM   Page 1 of 1

HPLC of racemic-**2p**

Data File E:\DATA\LGY\LGY-1-53-RE-3\LGY-1-53-RE-3 2017-12-01 08-52-48\003-0601.D
Sample Name: LGY-1-55

===

Acq. Operator : SYSTEM Seq. Line : 6
Acq. Instrument : 1260HPLC-VWD Location : Vial 3
Injection Date : 12/1/2017 1:20:12 PM Inj : 1
Inj Volume : 5.000 µl
Acq. Method : E:\DATA\LGY\LGY-1-53-RE-3\LGY-1-53-RE-3 2017-12-01 08-52-48\VWD-AS(1-6)-80-20-1ML-5UL-220NM-120MIN.M
Last changed : 12/1/2017 8:52:49 AM by SYSTEM
Analysis Method : E:\DATA\LGY\LGY-1-53-RE-3\LGY-1-53-RE-3 2017-12-01 08-52-48\VWD-AS(1-6)-80-20-1ML-5UL-220NM-120MIN.M (Sequence Method)
Last changed : 4/14/2018 7:53:32 PM by SYSTEM
(modified after loading)
Additional Info : Peak(s) manually integrated

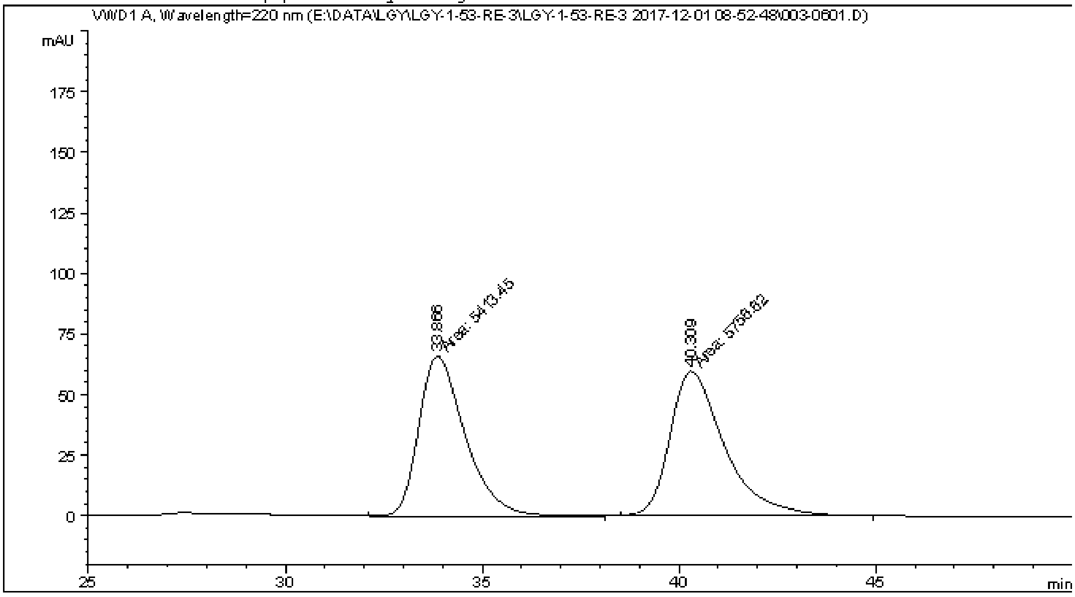

===

Area Percent Report

===

Sorted By : Signal
Multiplier : 1.0000
Dilution : 1.0000
Do not use Multiplier & Dilution Factor with ISTDs

Signal 1: VWD1 A, Wavelength=220 nm

Peak #	RetTime [min]	Type	Width [min]	Area [mAU*s]	Height [mAU]	Area %
1	33.866	MM	1.3651	5413.44727	66.09346	48.4639
2	40.309	MM	1.6109	5756.62109	59.55768	51.5361

Totals : 1.11701e4 125.65114

===

*** End of Report ***

1260HPLC-DAD 4/14/2018 7:53:39 PM SYSTEM Page 1 of 1

HPLC of **2p**

Data File E:\DATA\LGY\LGY-1-99\LGY-1-99 2018-01-18 18-48-43\011-0701.D
Sample Name: LGY-1-99-4-RE-1

```
=====================================================================
Acq. Operator   : SYSTEM                          Seq. Line :   7
Acq. Instrument : 1260HPLC-VWD                     Location : Vial 11
Injection Date  : 1/18/2018 11:34:54 PM                 Inj :   1
                                                Inj Volume : 5.000 µl
Acq. Method     : E:\DATA\LGY\LGY-1-99\LGY-1-99 2018-01-18 18-48-43\VWD-AS(1-6)-80-20-1ML-
                  5UL-220NM-60MIN.M
Last changed    : 1/18/2018 6:48:44 PM by SYSTEM
Analysis Method : E:\DATA\LGY\LGY-1-99\LGY-1-99 2018-01-18 18-48-43\VWD-AS(1-6)-80-20-1ML-
                  5UL-220NM-60MIN.M (Sequence Method)
Last changed    : 4/14/2018 7:57:47 PM by SYSTEM
                  (modified after loading)
Additional Info : Peak(s) manually integrated
```

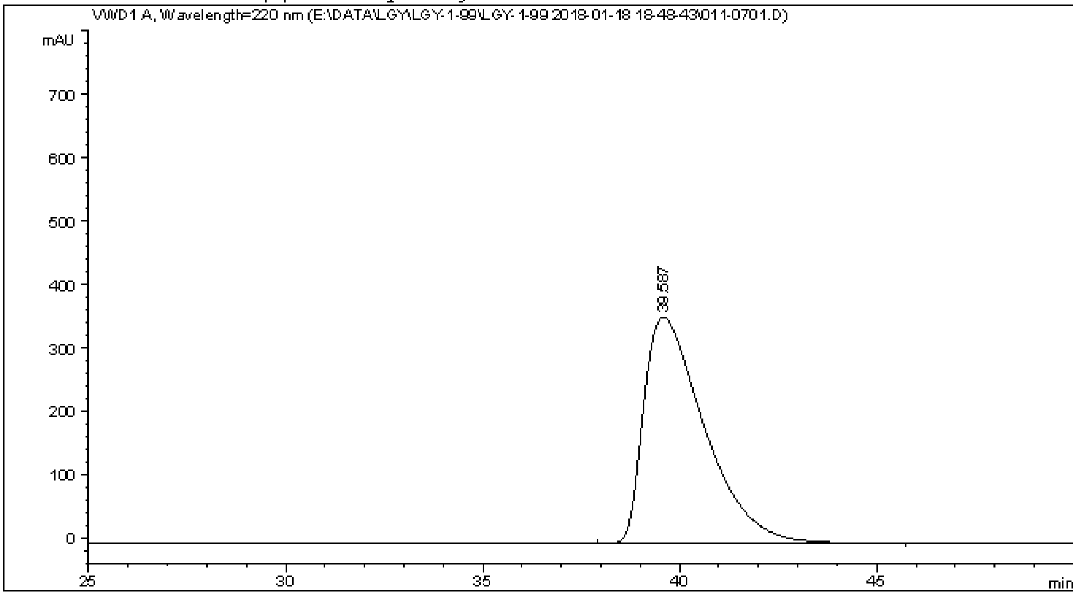


```
=====================================================================
                         Area Percent Report
=====================================================================

Sorted By             :      Signal
Multiplier            :      1.0000
Dilution              :      1.0000
Do not use Multiplier & Dilution Factor with ISTDs


Signal 1: VWD1 A, Wavelength=220 nm
```

Peak #	RetTime [min]	Type	Width [min]	Area [mAU*s]	Height [mAU]	Area %
1	39.587	BB	1.6551	3.82501e4	356.39026	100.0000

Totals : 3.82501e4 356.39026

```
=====================================================================
                         *** End of Report ***
```

HPLC of racemic-**2q**

Data File D:\DATA\LGY\LGY-2-73-RE\LGY-2-73-RE 2018-09-07 16-23-54\031-2701.D
Sample Name: LGY-2-32-RE

===

Acq. Operator : Seq. Line : 27
Acq. Instrument : Instrument 1 Location : Vial 31
Injection Date : 9/8/2018 2:57:05 PM Inj : 1
Inj Volume : 5.000 µl
Acq. Method : D:\DATA\LGY\LGY-2-73-RE\LGY-2-73-RE 2018-09-07 16-23-54\VWD-AS(1-6)-80-20-1ML-5UL-220NM-60MIN.M
Last changed : 5/31/2018 10:45:35 AM
Analysis Method : D:\METHOD\LG\VWD-AD(1-2)-99-1-0.2ML-2UL-220NM-10MIN.M
Last changed : 9/10/2018 10:22:01 PM
(modified after loading)
Additional Info : Peak(s) manually integrated

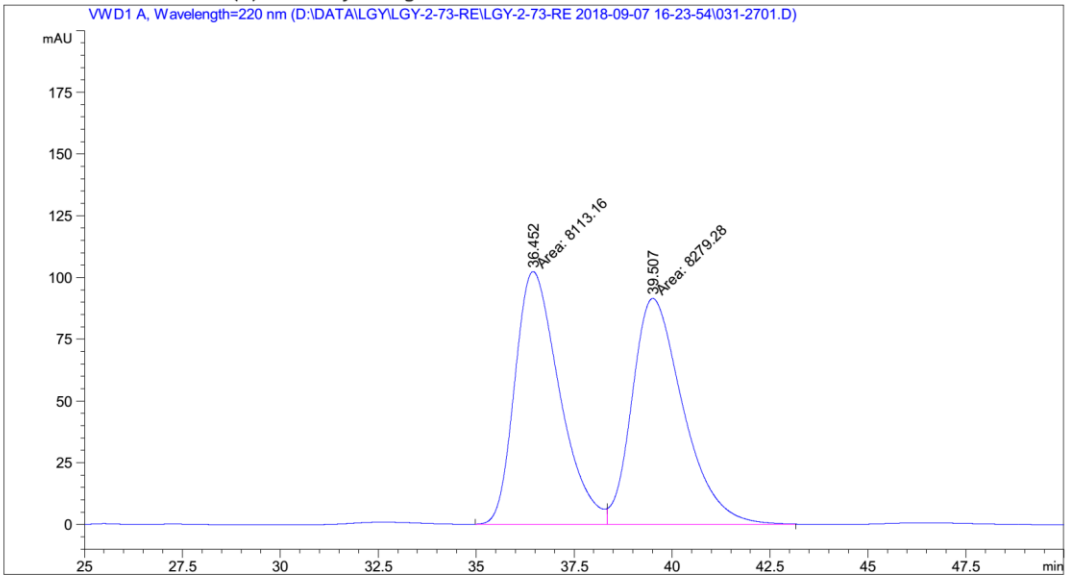

===

Area Percent Report

===

Sorted By : Retention Time
Multiplier : 1.0000
Dilution : 1.0000
Use Multiplier & Dilution Factor with ISTDs

Signal 1: VWD1 A, Wavelength=220 nm

Peak #	RetTime [min]	Sig	Type	Area [mAU*s]	Height [mAU]	Area %
1	36.452	1	MF	8113.15771	102.32407	49.4933
2	39.507	1	FM	8279.27539	91.44144	50.5067

Totals : 1.63924e4 193.76551

===

HPLC of **2q**

Data File D:\DATA\LGY\LGY-2-73-RE\LGY-2-73-RE 2018-09-07 16-23-54\032-2801.D
Sample Name: LGY-2-73-7

```
=====================================================================
Acq. Operator   :                                 Seq. Line :  28
Acq. Instrument : Instrument 1                     Location : Vial 32
Injection Date  : 9/8/2018 3:57:57 PM                   Inj :   1
                                                 Inj Volume : 5.000 µl
Acq. Method     : D:\DATA\LGY\LGY-2-73-RE\LGY-2-73-RE 2018-09-07 16-23-54\VWD-AS(1-6)-80-20-
                  1ML-5UL-220NM-60MIN.M
Last changed    : 9/8/2018 4:51:09 PM
                  (modified after loading)
Analysis Method : D:\METHOD\LG\VWD-AD(1-2)-99-1-0.2ML-2UL-220NM-10MIN.M
Last changed    : 9/10/2018 10:24:16 PM
                  (modified after loading)
Additional Info : Peak(s) manually integrated
```

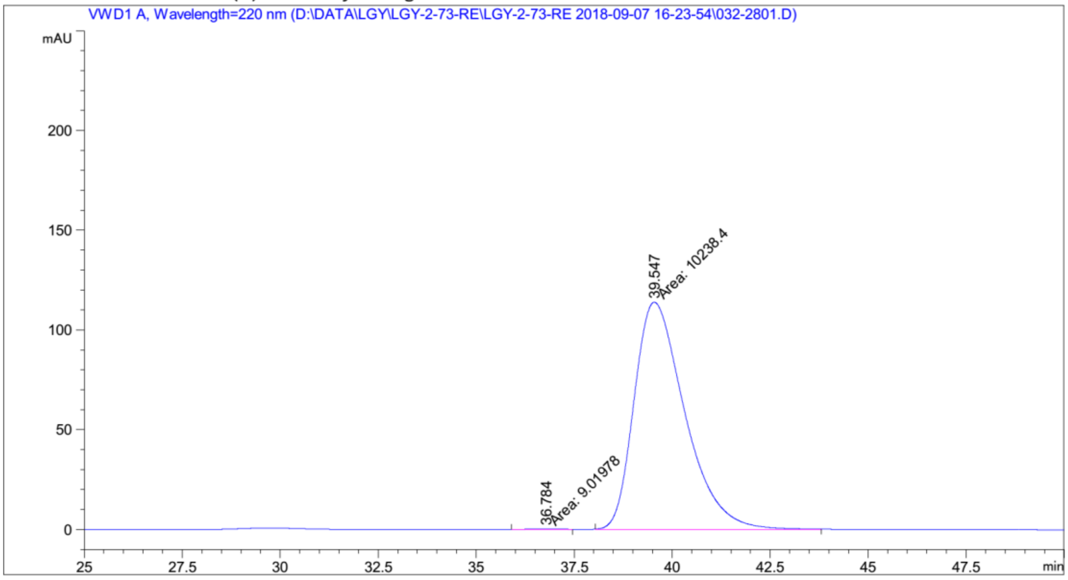


```
=====================================================================
                         Area Percent Report
=====================================================================

Sorted By             :      Retention Time
Multiplier            :      1.0000
Dilution              :      1.0000
Use Multiplier & Dilution Factor with ISTDs

Signal 1: VWD1 A, Wavelength=220 nm

Peak RetTime Sig Type    Area      Height     Area
  #   [min]             [mAU*s]     [mAU]       %
----|-------|---|----|----------|----------|--------|
   1  36.784  1   MM      9.01978 1.69177e-1   0.0880
   2  39.547  1   MM   1.02384e4  113.83140  99.9120

Totals :               1.02474e4  114.00058
```

HPLC of racemic-**2r**

```
Data File E:\DATA\LGY\LGY-1-95\LGY-1-95-RE 2018-01-13 18-47-33\065-0801.D
Sample Name: LGY-1-53-RA

    =====================================================================
    Acq. Operator   : SYSTEM                        Seq. Line :   8
    Acq. Instrument : 1260HPLC-VWD                   Location : Vial 65
    Injection Date  : 1/14/2018 4:24:33 AM                Inj :   1
                                                   Inj Volume : 5.000 µl
    Acq. Method     : E:\DATA\LGY\LGY-1-95\LGY-1-95-RE 2018-01-13 18-47-33\VWD-AS(1-6)-80-20-
                      1ML-5UL-220NM-90MIN.M
    Last changed    : 1/13/2018 6:47:33 PM by SYSTEM
    Analysis Method : E:\DATA\LGY\LGY-1-95\LGY-1-95-RE 2018-01-13 18-47-33\VWD-AS(1-6)-80-20-
                      1ML-5UL-220NM-90MIN.M (Sequence Method)
    Last changed    : 4/13/2018 9:31:55 PM by SYSTEM
                      (modified after loading)
    Additional Info : Peak(s) manually integrated
```

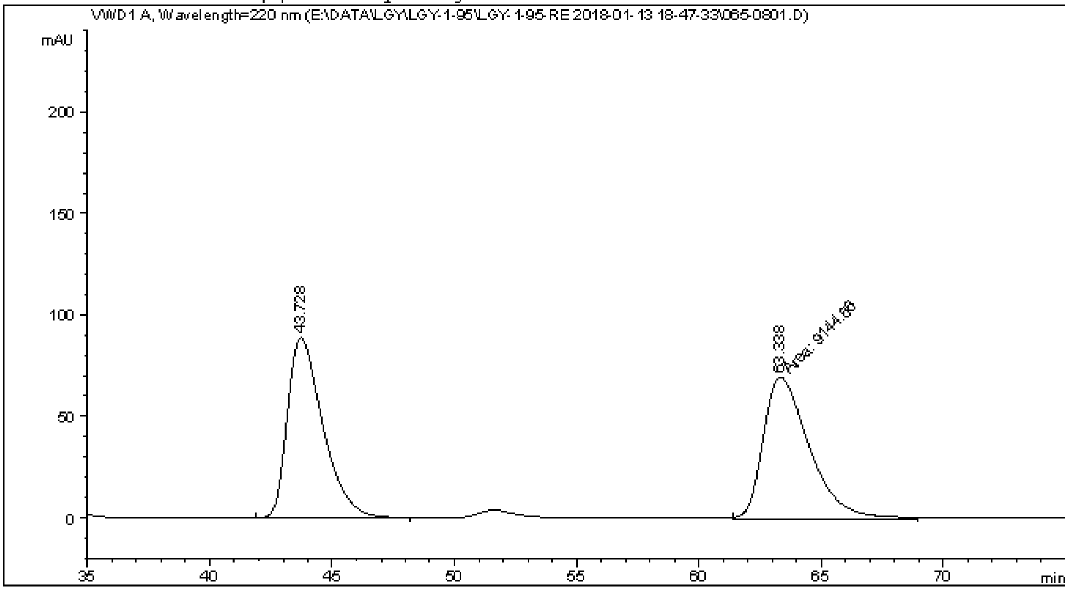


```
    =====================================================================
                         Area Percent Report
    =====================================================================

    Sorted By             :      Signal
    Multiplier            :      1.0000
    Dilution              :      1.0000
    Do not use Multiplier & Dilution Factor with ISTDs


    Signal 1: VWD1 A, Wavelength=220 nm
```

Peak #	RetTime [min]	Type	Width [min]	Area [mAU*s]	Height [mAU]	Area %
1	43.728	BB	1.5127	8898.92871	88.68528	49.3191
2	63.338	MM	2.1875	9144.65723	69.67457	50.6809
Totals :				1.80436e4	158.35985	

```
    =====================================================================
                          *** End of Report ***

1260HPLC-VWD 4/13/2018 9:32:08 PM SYSTEM                              Page 1 of 1
```

HPLC of **2r**

Data File E:\DATA\LGY\LGY-1-95-2\LGY-1-95-2-RE 2018-05-07 07-47-01\063-0201.D
Sample Name: LGY-1-95-2-RE

```
=====================================================================
Acq. Operator   : SYSTEM                          Seq. Line :   2
Acq. Instrument : 1260HPLC-VWD                     Location : Vial 63
Injection Date  : 5/7/2018 8:01:28 AM                   Inj :   1
                                                Inj Volume : 5.000 µl
Acq. Method     : E:\DATA\LGY\LGY-1-95-2\LGY-1-95-2-RE 2018-05-07 07-47-01\VWD-AS(1-6)-80-
                  20-1ML-5UL-220NM-90MIN.M
Last changed    : 5/7/2018 7:47:01 AM by SYSTEM
Analysis Method : E:\DATA\LGY\LGY-1-95-2\LGY-1-95-2-RE 2018-05-07 07-47-01\VWD-AS(1-6)-80-
                  20-1ML-5UL-220NM-90MIN.M (Sequence Method)
Last changed    : 5/7/2018 7:15:25 PM by SYSTEM
                  (modified after loading)
Additional Info : Peak(s) manually integrated
```

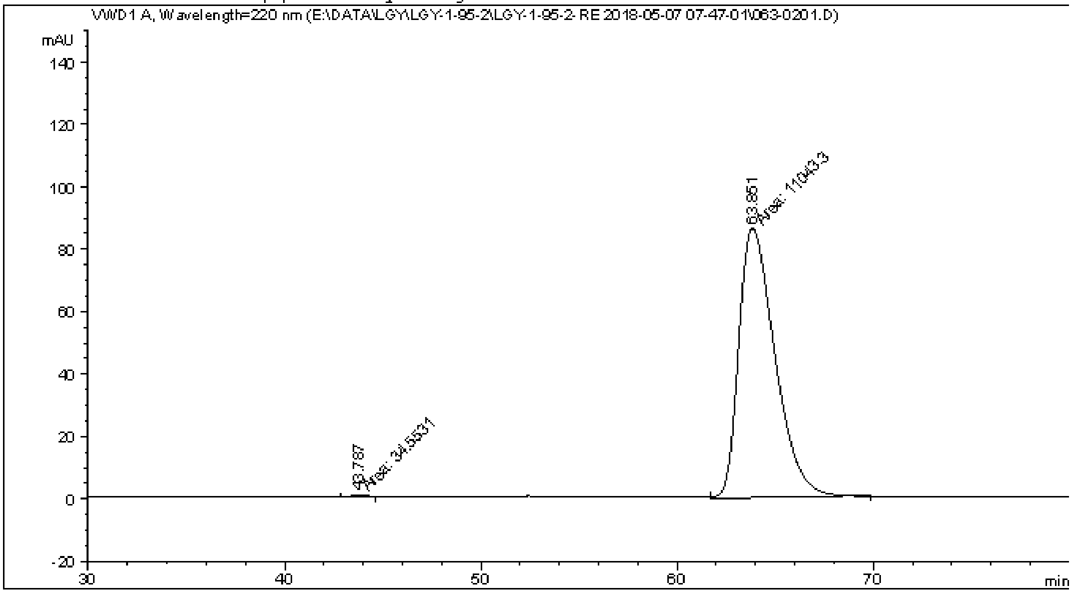


```
=====================================================================
                        Area Percent Report
=====================================================================

Sorted By             :      Signal
Multiplier            :      1.0000
Dilution              :      1.0000
Do not use Multiplier & Dilution Factor with ISTDs


Signal 1: VWD1 A, Wavelength=220 nm

Peak RetTime Type  Width     Area      Height     Area
  #   [min]        [min]   [mAU*s]     [mAU]        %
----|-------|----|-------|----------|----------|--------|
   1  43.787 MM    0.6485   34.55308 6.31873e-1   0.3119
   2  63.851 MM    2.1334 1.10433e4   86.27399  99.6881

Totals :                  1.10778e4   86.90586

=====================================================================
                        *** End of Report ***
```

1260HPLC-VWD 5/7/2018 7:15:31 PM SYSTEM   Page 1 of 1

HPLC of racemic-**2s**

Data File D:\DATA\LGY\LGY-2-73-RE\LGY-2-73-RE 2018-09-07 16-23-54\011-1101.D
Sample Name: LGY-2-62-2

===

Acq. Operator : Seq. Line : 11
Acq. Instrument : Instrument 1 Location : Vial 11
Injection Date : 9/8/2018 1:33:26 AM Inj : 1
Inj Volume : 5.000 µl
Acq. Method : D:\DATA\LGY\LGY-2-73-RE\LGY-2-73-RE 2018-09-07 16-23-54\VWD-AS(1-6)-80-20-1ML-5UL-220NM-80MIN.M
Last changed : 5/26/2018 3:44:58 PM
Analysis Method : D:\METHOD\LGY\VWD-AD(1-2)-95-5-0.3ML-5UL-220NM-100MIN.M
Last changed : 9/10/2018 9:48:14 PM
(modified after loading)
Additional Info : Peak(s) manually integrated

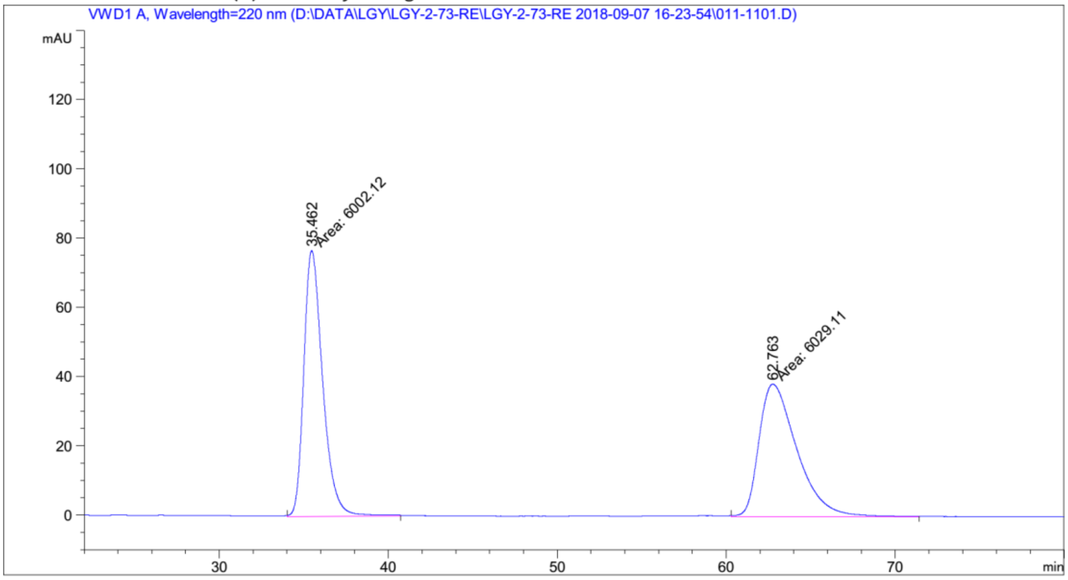

===

Area Percent Report

===

Sorted By : Retention Time
Multiplier : 1.0000
Dilution : 1.0000
Use Multiplier & Dilution Factor with ISTDs

Signal 1: VWD1 A, Wavelength=220 nm

Peak #	RetTime [min]	Sig	Type	Area [mAU*s]	Height [mAU]	Area %
1	35.462	1	MM	6002.12256	76.84860	49.8878
2	62.763	1	MM	6029.10986	38.34726	50.1122

Totals : 1.20312e4 115.19587

===

Instrument 1 9/10/2018 9:48:17 PM Page 1 of 1

HPLC of **2s**

Data File D:\DATA\LGY\LGY-2-73-RE\LGY-2-73-RE 2018-09-07 16-23-54\042-3401.D
Sample Name: LGY-2-73-10

```
=====================================================================
Acq. Operator   :                                 Seq. Line :  34
Acq. Instrument : Instrument 1                     Location : Vial 42
Injection Date  : 9/8/2018 11:41:08 PM                  Inj :   1
                                                 Inj Volume : 5.000 µl
Acq. Method     : D:\DATA\LGY\LGY-2-73-RE\LGY-2-73-RE 2018-09-07 16-23-54\VWD-AS(1-6)-80-20-
                  1ML-5UL-220NM-80MIN.M
Last changed    : 9/8/2018 10:08:46 PM
                  (modified after loading)
Analysis Method : D:\METHOD\LG\VWD-AD(1-2)-99-1-0.2ML-2UL-220NM-10MIN.M
Last changed    : 9/10/2018 10:32:41 PM
                  (modified after loading)
Additional Info : Peak(s) manually integrated
```

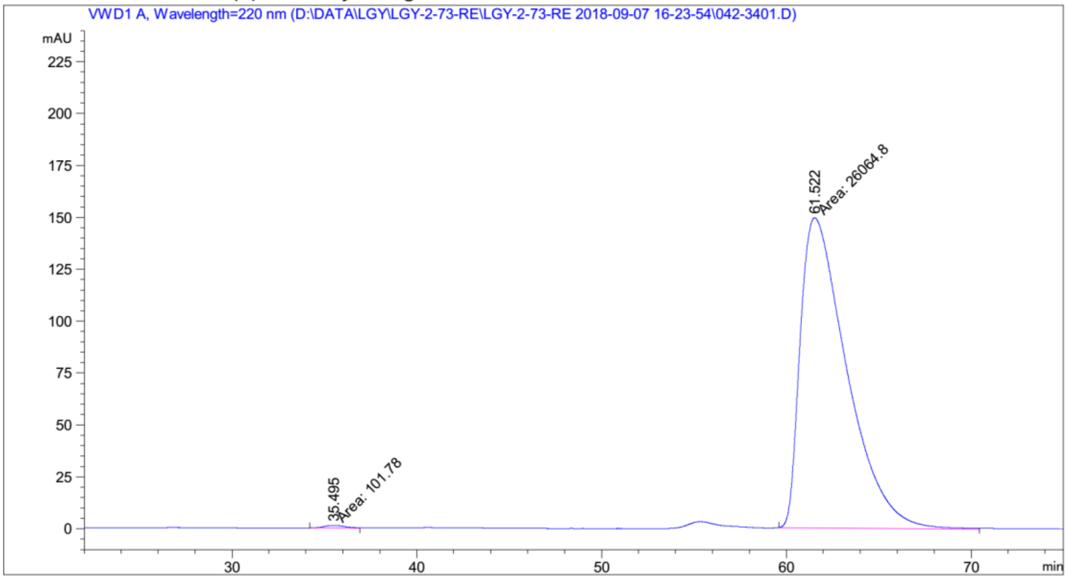


```
=====================================================================
                         Area Percent Report
=====================================================================

Sorted By             :      Retention Time
Multiplier            :      1.0000
Dilution              :      1.0000
Use Multiplier & Dilution Factor with ISTDs

Signal 1: VWD1 A, Wavelength=220 nm
```

Peak #	RetTime [min]	Sig	Type	Area [mAU*s]	Height [mAU]	Area %
1	35.495	1	MM	101.77962	1.28853	0.3890
2	61.522	1	MM	2.60648e4	149.35780	99.6110

Totals : 2.61665e4 150.64634

HPLC of racemic-**2t**

Data File D:\DATA\LGY\LGY-2-73-RE\LGY-2-73-RE 2018-09-07 16-23-54\005-0601.D
Sample Name: LGY-2-62-1

===
Acq. Operator   :   Seq. Line :   6
Acq. Instrument : Instrument 1   Location : Vial 5
Injection Date : 9/7/2018 9:19:02 PM   Inj :   1
                                                    Inj Volume : 5.000 µl
Acq. Method   : D:\DATA\LGY\LGY-2-73-RE\LGY-2-73-RE 2018-09-07 16-23-54\VWD-AS(1-6)-80-20-1ML-5UL-220NM-80MIN.M
Last changed   : 5/26/2018 3:44:58 PM
Analysis Method : D:\METHOD\LGY\VWD-AD(1-2)-95-5-0.3ML-5UL-220NM-100MIN.M
Last changed   : 9/10/2018 9:42:43 PM
                  (modified after loading)
Additional Info : Peak(s) manually integrated

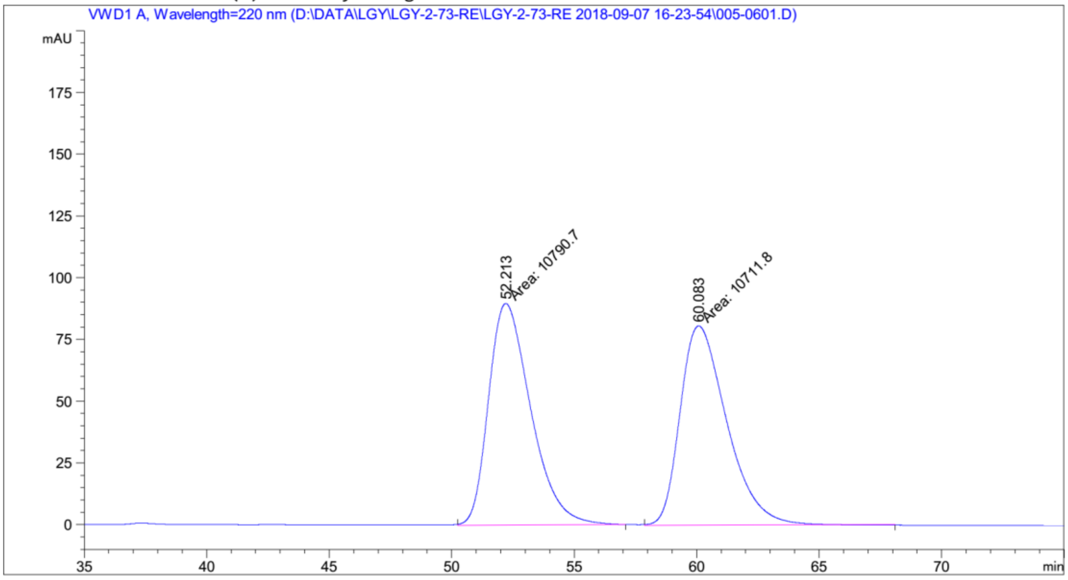

===
Area Percent Report
===

Sorted By : Retention Time
Multiplier : 1.0000
Dilution : 1.0000
Use Multiplier & Dilution Factor with ISTDs

Signal 1: VWD1 A, Wavelength=220 nm

Peak #	RetTime [min]	Sig	Type	Area [mAU*s]	Height [mAU]	Area %
1	52.213	1	MM	1.07907e4	89.78436	50.1836
2	60.083	1	MM	1.07118e4	80.76544	49.8164
Totals :				2.15025e4	170.54980	

===

Instrument 1 9/10/2018 9:42:46 PM   Page 1 of 1

HPLC of **2t**

Data File D:\DATA\LGY\LGY-2-73-RE\LGY-2-73-RE 2018-09-07 16-23-54\036-3201.D
Sample Name: LGY-2-73-9

===

Acq. Operator : Seq. Line : 32
Acq. Instrument : Instrument 1 Location : Vial 36
Injection Date : 9/8/2018 8:39:25 PM Inj : 1
Inj Volume : 5.000 µl
Acq. Method : D:\DATA\LGY\LGY-2-73-RE\LGY-2-73-RE 2018-09-07 16-23-54\VWD-AS(1-6)-80-20-1ML-5UL-220NM-80MIN.M
Last changed : 9/8/2018 8:41:54 PM
(modified after loading)
Analysis Method : D:\METHOD\LG\VWD-AD(1-2)-99-1-0.2ML-2UL-220NM-10MIN.M
Last changed : 9/10/2018 10:30:13 PM
(modified after loading)
Additional Info : Peak(s) manually integrated

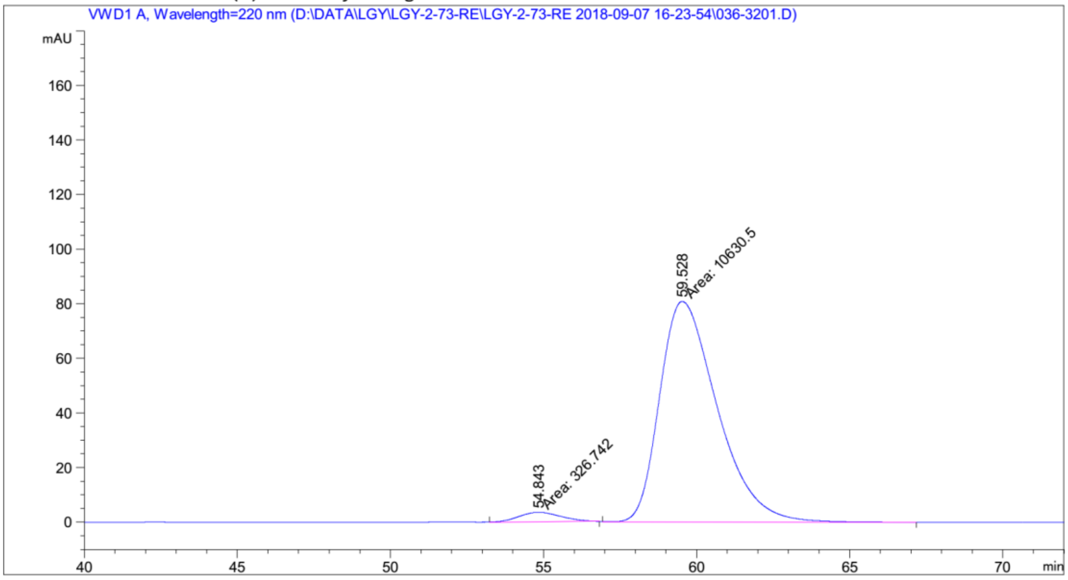

===

Area Percent Report

===

Sorted By : Retention Time
Multiplier : 1.0000
Dilution : 1.0000
Use Multiplier & Dilution Factor with ISTDs

Signal 1: VWD1 A, Wavelength=220 nm

Peak #	RetTime [min]	Sig	Type	Area [mAU*s]	Height [mAU]	Area %
1	54.843	1	MM	326.74197	3.44832	2.9820
2	59.528	1	MM	1.06305e4	80.87628	97.0180

Totals : 1.09572e4 84.32460

HPLC of racemic-**2u**

Data File D:\DATA\LGY\LGY-2-73-RE\LGY-2-73-RE 2018-09-07 16-23-54\003-0401.D
Sample Name: LGY-2-53-4

===

Acq. Operator : Seq. Line : 4
Acq. Instrument : Instrument 1 Location : Vial 3
Injection Date : 9/7/2018 6:37:24 PM Inj : 1
Inj Volume : 5.000 µl
Acq. Method : D:\DATA\LGY\LGY-2-73-RE\LGY-2-73-RE 2018-09-07 16-23-54\VWD-AS(1-6)-80-20-1ML-5UL-220NM-80MIN.M
Last changed : 5/26/2018 3:44:58 PM
Analysis Method : D:\METHOD\LGY\VWD-AD(1-2)-95-5-0.3ML-5UL-220NM-100MIN.M
Last changed : 9/10/2018 9:36:41 PM
(modified after loading)
Additional Info : Peak(s) manually integrated

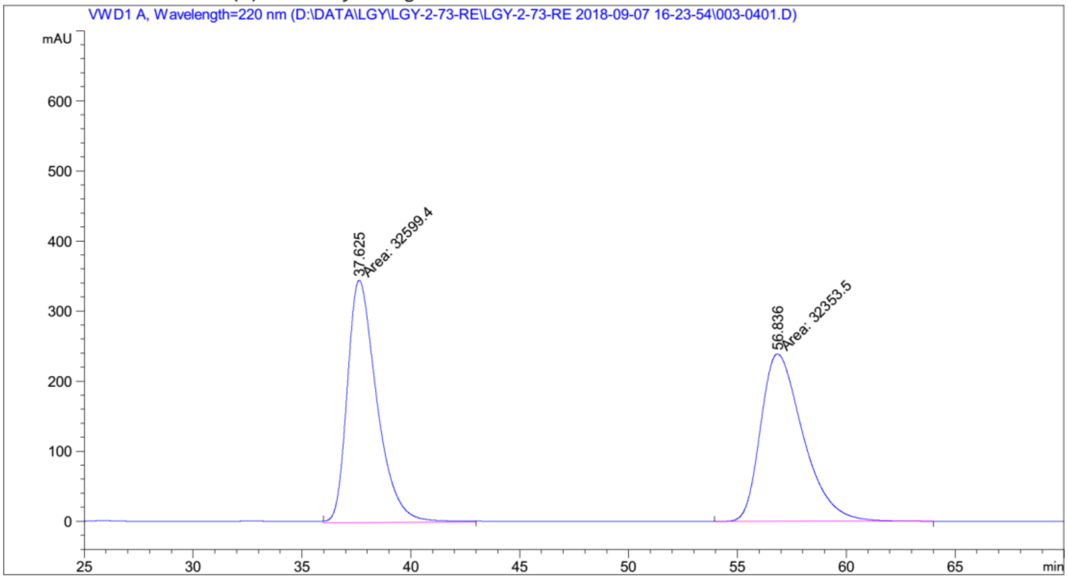

===

Area Percent Report

===

Sorted By : Retention Time
Multiplier : 1.0000
Dilution : 1.0000
Use Multiplier & Dilution Factor with ISTDs

Signal 1: VWD1 A, Wavelength=220 nm

Peak #	RetTime [min]	Sig	Type	Area [mAU*s]	Height [mAU]	Area %
1	37.625	1	MM	3.25994e4	345.56903	50.1893
2	56.836	1	MM	3.23535e4	238.93916	49.8107

Totals : 6.49529e4 584.50819

===

Instrument 1 9/10/2018 9:36:46 PM Page 1 of 1

HPLC of **2u**

```
Data File D:\DATA\LGY\LGY-2-73-RE\LGY-2-73-RE 2018-09-07 16-23-54\034-3001.D
Sample Name: LGY-2-73-8

=====================================================================
Acq. Operator   :                               Seq. Line :  30
Acq. Instrument : Instrument 1                   Location : Vial 34
Injection Date  : 9/8/2018 6:13:42 PM                 Inj :   1
                                               Inj Volume : 5.000 µl
Acq. Method     : D:\DATA\LGY\LGY-2-73-RE\LGY-2-73-RE 2018-09-07 16-23-54\VWD-AS(1-6)-80-20-
                  1ML-5UL-220NM-80MIN.M
Last changed    : 9/8/2018 6:39:09 PM
                  (modified after loading)
Analysis Method : D:\METHOD\LG\VWD-AD(1-2)-99-1-0.2ML-2UL-220NM-10MIN.M
Last changed    : 9/10/2018 10:26:57 PM
                  (modified after loading)
Additional Info : Peak(s) manually integrated
```

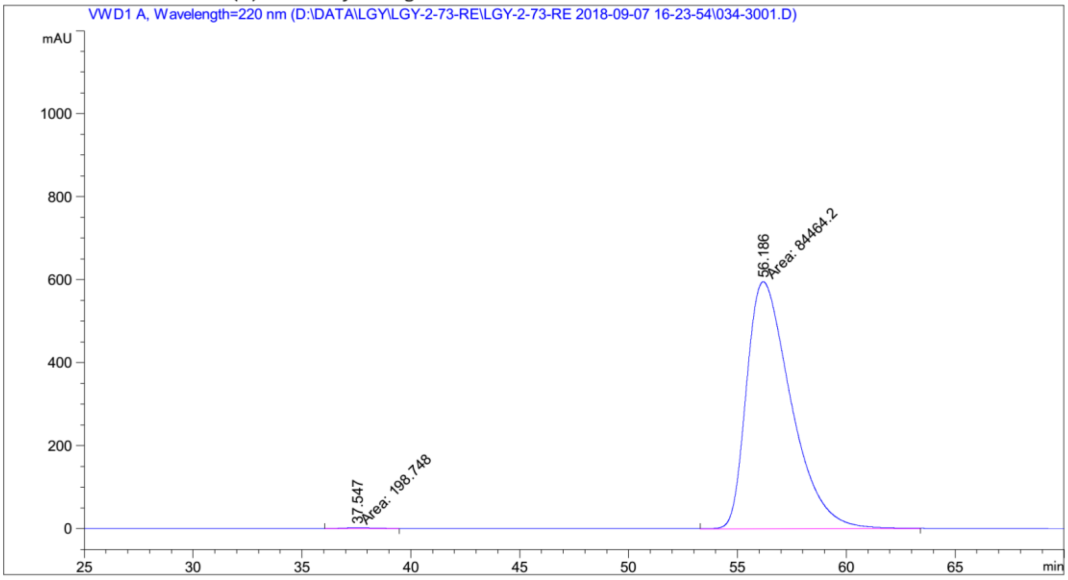


```
=====================================================================
                         Area Percent Report
=====================================================================

Sorted By             :      Retention Time
Multiplier            :      1.0000
Dilution              :      1.0000
Use Multiplier & Dilution Factor with ISTDs

Signal 1: VWD1 A, Wavelength=220 nm

Peak RetTime Sig Type    Area       Height     Area
  #   [min]              [mAU*s]    [mAU]        %
----|-------|---|----|----------|----------|--------|
   1  37.547   1  MM    198.74828    2.21174   0.2348
   2  56.186   1  MM   8.44642e4   595.49066  99.7652

Totals :               8.46630e4   597.70240
```

HPLC of racemic-**2v**

```
Data File E:\DATA\LGY\LGY-1-101\LGY-1-101 2018-01-20 15-54-03\041-0301.D
Sample Name: LGY-1-101

    =====================================================================
    Acq. Operator   : SYSTEM                          Seq. Line :   3
    Acq. Instrument : 1260HPLC-VWD                     Location : Vial 41
    Injection Date  : 1/20/2018 4:36:17 PM                  Inj :   1
                                                     Inj Volume : 3.000 µl
    Acq. Method     : E:\DATA\LGY\LGY-1-101\LGY-1-101 2018-01-20 15-54-03\VWD-AD(1-2)-95-5-1ML
                      -3UL-220NM-60MIN.M
    Last changed    : 1/20/2018 3:54:03 PM by SYSTEM
    Analysis Method : E:\DATA\LGY\LGY-1-101\LGY-1-101 2018-01-20 15-54-03\VWD-AD(1-2)-95-5-1ML
                      -3UL-220NM-60MIN.M (Sequence Method)
    Last changed    : 4/14/2018 8:00:08 PM by SYSTEM
                      (modified after loading)
    Additional Info : Peak(s) manually integrated
```

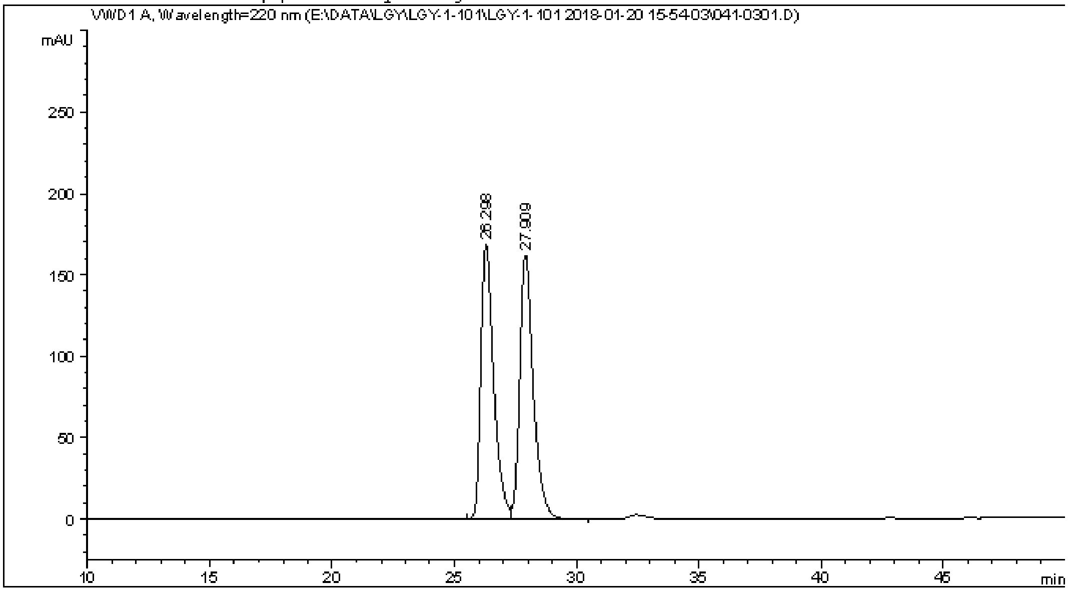


```
    =====================================================================
                          Area Percent Report
    =====================================================================

    Sorted By             :      Signal
    Multiplier            :      1.0000
    Dilution              :      1.0000
    Do not use Multiplier & Dilution Factor with ISTDs


    Signal 1: VWD1 A, Wavelength=220 nm

    Peak RetTime Type  Width     Area      Height     Area
      #   [min]        [min]   [mAU*s]     [mAU]        %
    ----|-------|----|-------|----------|----------|--------|
       1  26.298 BV    0.5487 6153.86182  168.66835  49.6834
       2  27.909 VB    0.5753 6232.27979  162.20204  50.3166

    Totals :                  1.23861e4   330.87039


    =====================================================================
                           *** End of Report ***
```

1260HPLC-DAD 4/14/2018 8:00:32 PM SYSTEM Page 1 of 1

HPLC of **2v**

Data File E:\DATA\LGY\LGY-1-101\LGY-1-101 2018-01-20 15-54-03\042-0401.D
Sample Name: LGY-1-99-6

```
=====================================================================
Acq. Operator   : SYSTEM                          Seq. Line :   4
Acq. Instrument : 1260HPLC-VWD                     Location : Vial 42
Injection Date  : 1/20/2018 5:37:01 PM                  Inj :   1
                                                 Inj Volume : 3.000 µl
Acq. Method     : E:\DATA\LGY\LGY-1-101\LGY-1-101 2018-01-20 15-54-03\VWD-AD(1-2)-95-5-1ML
                  -3UL-220NM-60MIN.M
Last changed    : 1/20/2018 3:54:03 PM by SYSTEM
Analysis Method : E:\DATA\LGY\LGY-1-101\LGY-1-101 2018-01-20 15-54-03\VWD-AD(1-2)-95-5-1ML
                  -3UL-220NM-60MIN.M (Sequence Method)
Last changed    : 4/14/2018 8:06:25 PM by SYSTEM
                  (modified after loading)
Additional Info : Peak(s) manually integrated
```

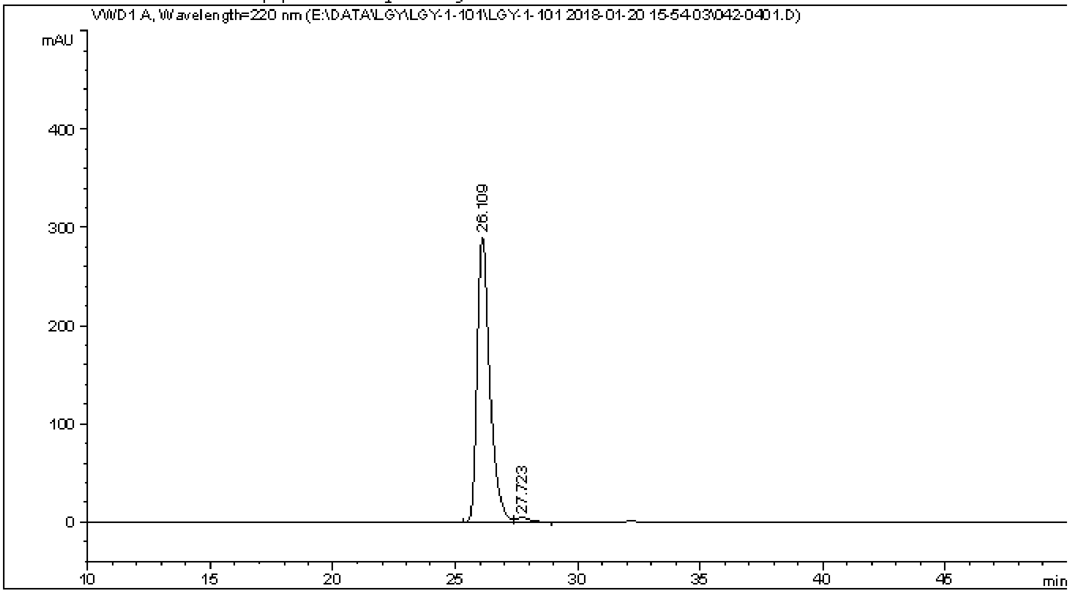


```
=====================================================================
                       Area Percent Report
=====================================================================

Sorted By             :      Signal
Multiplier            :      1.0000
Dilution              :      1.0000
Do not use Multiplier & Dilution Factor with ISTDs


Signal 1: VWD1 A, Wavelength=220 nm

Peak RetTime Type  Width     Area      Height     Area
  #   [min]        [min]   [mAU*s]     [mAU]        %
----|-------|----|-------|----------|----------|--------|
   1  26.109 BV    0.5409 1.04457e4   290.20370  98.5545
   2  27.723 VB    0.5486  153.20755    4.02011   1.4455

Totals :                  1.05989e4   294.22382

=====================================================================
                         *** End of Report ***
```

1260HPLC-DAD 4/14/2018 8:06:42 PM SYSTEM

HPLC of racemic-**2w**

Data File D:\DATA\LGY\LGY-2-73-RE\LGY-2-73-RE 2018-09-07 16-23-54\013-1401.D
Sample Name: LGY-2-35-1

===
Acq. Operator   :   Seq. Line : 14
Acq. Instrument : Instrument 1   Location : Vial 13
Injection Date : 9/8/2018 4:36:06 AM   Inj :   1
                                                  Inj Volume : 5.000 µl
Acq. Method   : D:\DATA\LGY\LGY-2-73-RE\LGY-2-73-RE 2018-09-07 16-23-54\VWD-AD(1-2)-95-5-0.3ML-5UL-220NM-100MIN.M
Last changed   : 9/7/2018 3:24:25 PM
Analysis Method : D:\METHOD\LGY\VWD-AD(1-2)-95-5-0.3ML-5UL-220NM-100MIN.M
Last changed   : 9/10/2018 9:53:01 PM
                  (modified after loading)
Additional Info : Peak(s) manually integrated

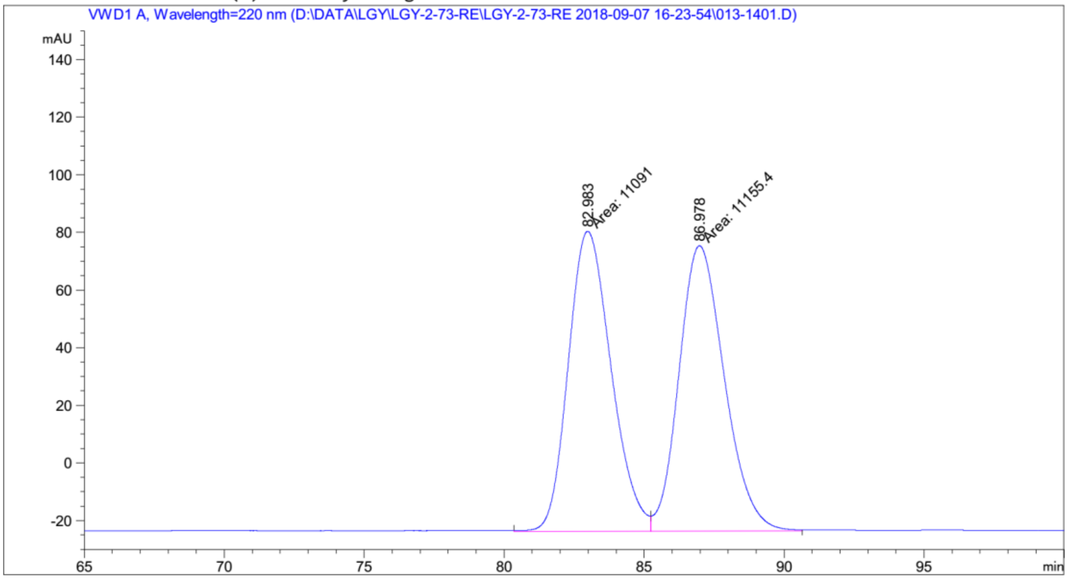

===
Area Percent Report
===

Sorted By : Retention Time
Multiplier : 1.0000
Dilution : 1.0000
Use Multiplier & Dilution Factor with ISTDs

Signal 1: VWD1 A, Wavelength=220 nm

Peak #	RetTime [min]	Sig	Type	Area [mAU*s]	Height [mAU]	Area %
1	82.983	1	MF	1.10910e4	104.12796	49.8553
2	86.978	1	FM	1.11554e4	99.01752	50.1447
Totals :				2.22464e4	203.14548	

===

HPLC of **2w**

Data File D:\DATA\LGY\LGY-2-73-RE\LGY-2-73-RE 2018-09-07 16-23-54\044-3701.D
Sample Name: LGY-2-73-11

===

Acq. Operator : Seq. Line : 37
Acq. Instrument : Instrument 1 Location : Vial 44
Injection Date : 9/9/2018 3:13:50 AM Inj : 1
Inj Volume : 5.000 µl
Acq. Method : D:\DATA\LGY\LGY-2-73-RE\LGY-2-73-RE 2018-09-07 16-23-54\VWD-AD(1-2)-95-5-0.3ML-5UL-220NM-100MIN.M
Last changed : 9/7/2018 3:24:25 PM
Analysis Method : D:\METHOD\LG\VWD-AD(1-2)-99-1-0.2ML-2UL-220NM-10MIN.M
Last changed : 9/10/2018 10:35:32 PM
(modified after loading)
Additional Info : Peak(s) manually integrated

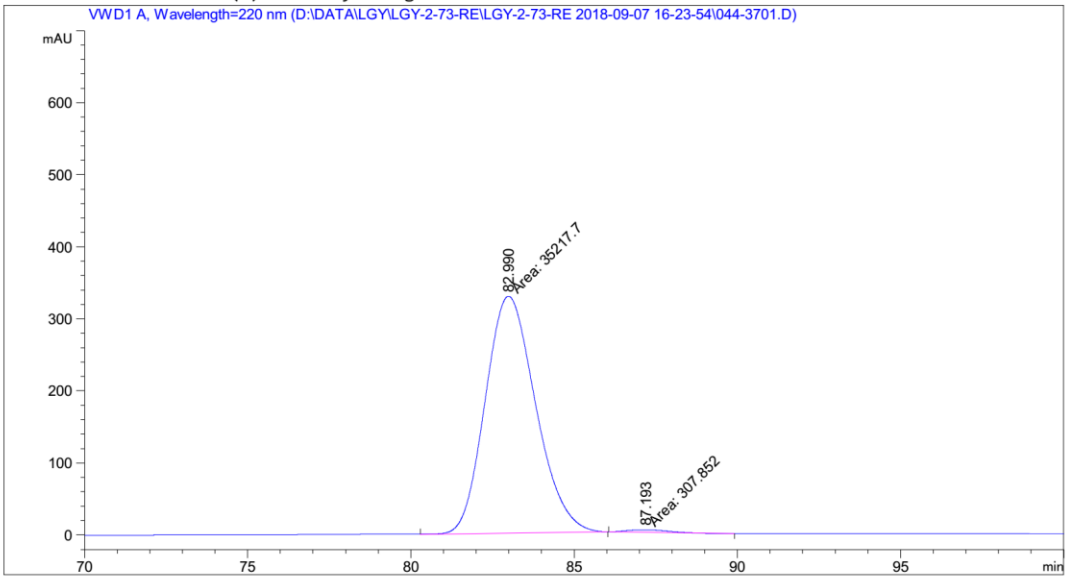

===

Area Percent Report

===

Sorted By : Retention Time
Multiplier : 1.0000
Dilution : 1.0000
Use Multiplier & Dilution Factor with ISTDs

Signal 1: VWD1 A, Wavelength=220 nm

Peak #	RetTime [min]	Sig	Type	Area [mAU*s]	Height [mAU]	Area %
1	82.990	1	MM	3.52177e4	328.70041	99.1334
2	87.193	1	MM	307.85239	3.58854	0.8666

Totals : 3.55255e4 332.28895

===

Instrument 1 9/10/2018 10:35:36 PM Page 1 of 1

HPLC of racemic-**2x**

Data File D:\DATA\LGY\LGY-2-73-RE\LGY-2-73-RE 2018-09-07 16-23-54\015-1601.D
Sample Name: LGY-2-64

===

Acq. Operator   :   Seq. Line : 16
Acq. Instrument : Instrument 1   Location : Vial 15
Injection Date : 9/8/2018 7:57:49 AM   Inj : 1
                                                 Inj Volume : 5.000 µl
Acq. Method   : D:\DATA\LGY\LGY-2-73-RE\LGY-2-73-RE 2018-09-07 16-23-54\VWD-AD(1-2)-95-5-0.3ML-5UL-220NM-90MIN.M
Last changed   : 6/14/2018 9:07:28 PM
Analysis Method : D:\METHOD\LG\VWD-AD(1-2)-99-1-0.2ML-2UL-220NM-10MIN.M
Last changed   : 9/10/2018 10:17:07 PM
                  (modified after loading)
Additional Info : Peak(s) manually integrated

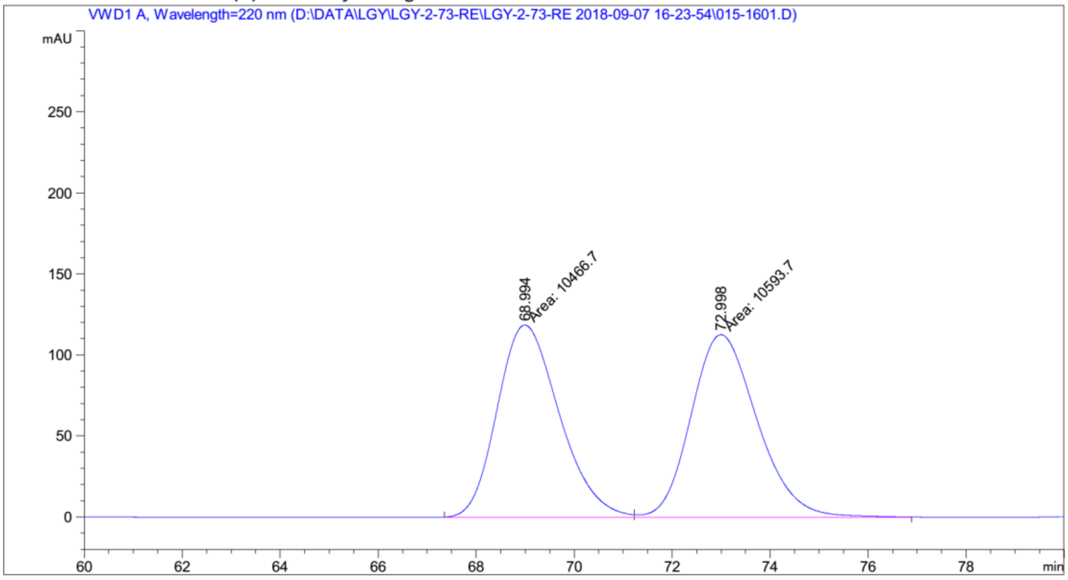

===

Area Percent Report

===

Sorted By : Retention Time
Multiplier : 1.0000
Dilution : 1.0000
Use Multiplier & Dilution Factor with ISTDs

Signal 1: VWD1 A, Wavelength=220 nm

Peak #	RetTime [min]	Sig	Type	Area [mAU*s]	Height [mAU]	Area %
1	68.994	1	MF	1.04667e4	118.64645	49.6986
2	72.998	1	FM	1.05937e4	112.75227	50.3014

Totals : 2.10604e4 231.39873

===

HPLC of **2x**

Data File D:\DATA\LGY\LGY-2-73-RE\LGY-2-73-RE 2018-09-07 16-23-54\046-3901.D
Sample Name: LGY-2-73-12

```
=====================================================================
Acq. Operator   :                                 Seq. Line :  39
Acq. Instrument : Instrument 1                     Location : Vial 46
Injection Date  : 9/9/2018 6:25:37 AM                   Inj :   1
                                                 Inj Volume : 5.000 µl
Acq. Method     : D:\DATA\LGY\LGY-2-73-RE\LGY-2-73-RE 2018-09-07 16-23-54\VWD-AD(1-2)-95-5-0.
                  3ML-5UL-220NM-90MIN.M
Last changed    : 6/14/2018 9:07:28 PM
Analysis Method : D:\METHOD\LG\VWD-AD(1-2)-99-1-0.2ML-2UL-220NM-10MIN.M
Last changed    : 9/10/2018 10:38:13 PM
                  (modified after loading)
Additional Info : Peak(s) manually integrated
```

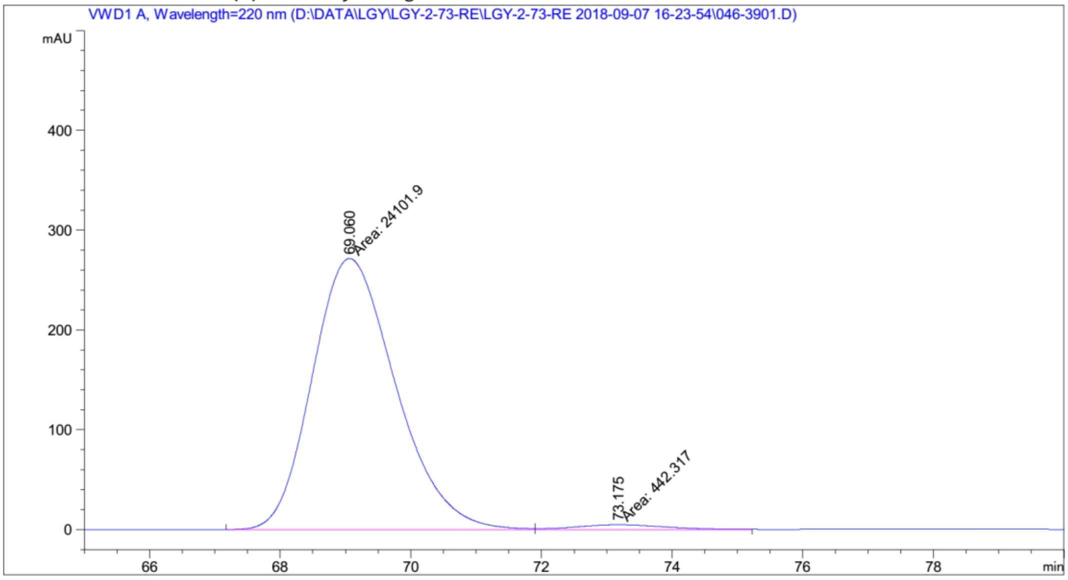


```
=====================================================================
                         Area Percent Report
=====================================================================

Sorted By             :      Retention Time
Multiplier            :      1.0000
Dilution              :      1.0000
Use Multiplier & Dilution Factor with ISTDs

Signal 1: VWD1 A, Wavelength=220 nm
```

Peak #	RetTime [min]	Sig	Type	Area [mAU*s]	Height [mAU]	Area %
1	69.060	1	MF	2.41019e4	271.55933	98.1979
2	73.175	1	FM	442.31656	4.69764	1.8021

Totals : 2.45442e4 276.25696

===

HPLC of racemic-**4a**

Data File D:\DATA\LGY\LGY-2-155-(2)\LGY-2-155-(2) 2018-11-26 09-15-18\001-0201.D
Sample Name: LGY-2-155

===

Acq. Operator	:	Seq. Line :	2
Acq. Instrument	: Instrument 2	Location :	Vial 1
Injection Date	: 11/26/2018 9:28:20 AM	Inj :	1
		Inj Volume :	5.000 µl

Acq. Method : D:\DATA\LGY\LGY-2-155-(2)\LGY-2-155-(2) 2018-11-26 09-15-18\DAD-OD(1-2)-90-10-1ML-5UL-ALL-80MIN.M
Last changed : 5/26/2018 3:40:32 PM
Analysis Method : D:\METHOD\TL\DAD-OJ(1-6)-80-20-1ML-3UL-ALL-10MIN.M
Last changed : 12/4/2018 9:40:39 AM
(modified after loading)
Additional Info : Peak(s) manually integrated

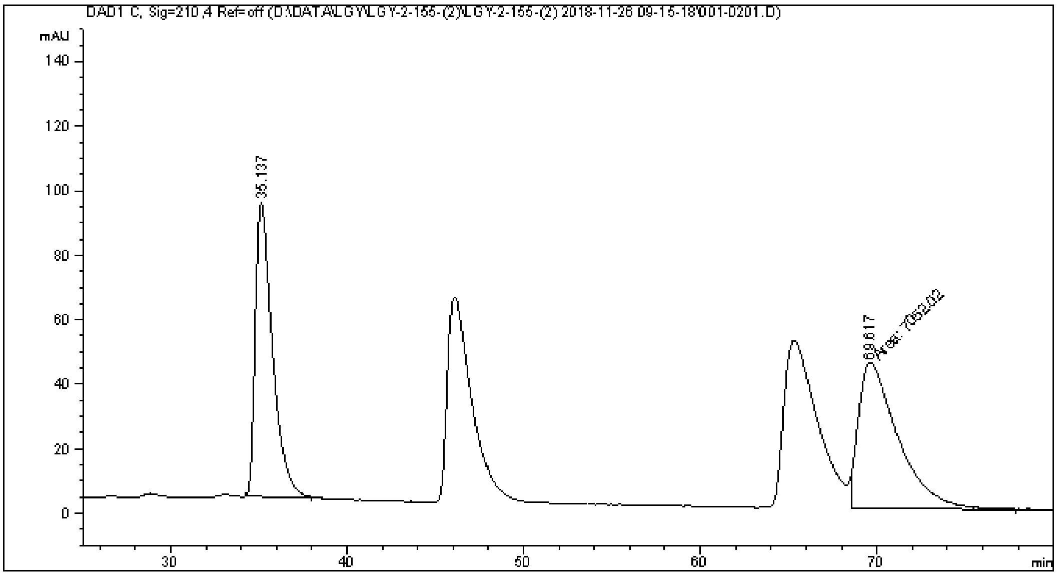

===

Area Percent Report

===

Sorted By : Signal
Multiplier : 1.0000
Dilution : 1.0000
Use Multiplier & Dilution Factor with ISTDs

Signal 1: DAD1 C, Sig=210,4 Ref=off

Peak #	RetTime [min]	Type	Width [min]	Area [mAU*s]	Height [mAU]	Area %
1	35.137	BB	0.8992	6142.91992	91.04142	46.5551
2	69.617	FM	2.6029	7052.02148	45.15573	53.4449

Totals : 1.31949e4 136.19715

HPLC of **4a**

Data File D:\DATA\LYH\LYH-3-606\LYH-3-606 2018-12-03 15-33-42\011-0601.D
Sample Name: LGY-3-14-1

===

Acq. Operator	:	Seq. Line :	6
Acq. Instrument	: Instrument 2	Location :	Vial 11
Injection Date	: 12/3/2018 5:16:10 PM	Inj :	1
		Inj Volume :	5.000 µl

Acq. Method : D:\DATA\LYH\LYH-3-606\LYH-3-606 2018-12-03 15-33-42\DAD-OD(1-2)-90-10-1ML-5UL-ALL-80MIN.M
Last changed : 5/26/2018 3:40:32 PM
Analysis Method : D:\METHOD\TL\DAD-OJ(1-6)-80-20-1ML-3UL-ALL-10MIN.M
Last changed : 12/3/2018 8:08:37 PM
(modified after loading)
Additional Info : Peak(s) manually integrated

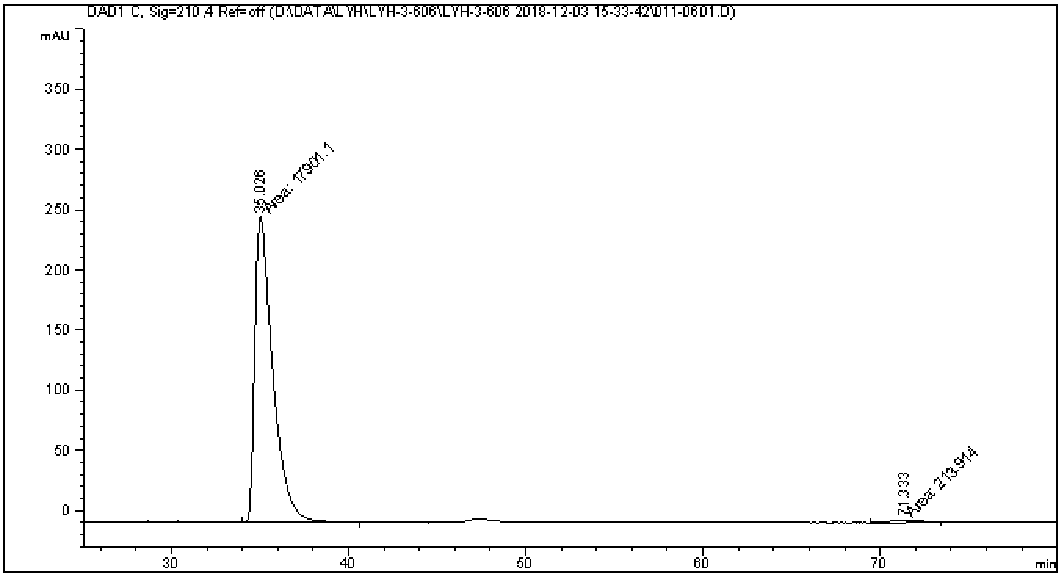

===
Area Percent Report
===

Sorted By : Signal
Multiplier : 1.0000
Dilution : 1.0000
Use Multiplier & Dilution Factor with ISTDs

Signal 1: DAD1 C, Sig=210,4 Ref=off

Peak #	RetTime [min]	Type	Width [min]	Area [mAU*s]	Height [mAU]	Area %
1	35.026	MM	1.1786	1.79011e4	253.14673	98.8191
2	71.333	MM	1.9375	213.91446	1.84013	1.1809
Totals :				1.81150e4	254.98685	
